# Identification of Prognostic DNA Methylation Signatures in Lung Adenocarcinoma

**DOI:** 10.1155/2022/8802303

**Published:** 2022-06-29

**Authors:** Pengli Wang, Gaoran Xu, Erji Gao, Yong Xu, Leilei Liang, Gening Jiang, Liang Duan

**Affiliations:** ^1^Department of Thoracic Surgery, Shanghai Pulmonary Hospital, School of Medicine, Tongji University, Shanghai 200433, China; ^2^Department of Thyroid and Breast Surgery, Zhongnan Hospital of Wuhan University, Wuhan 430071, China

## Abstract

**Background:**

Increasing evidence exists of a link between DNA methylation and tumor immunotherapy. However, the impact of DNA methylation on the characteristics of the lung adenocarcinoma microenvironment and its effect on immunotherapy remain unclear.

**Method:**

This study collected TCGA-LUAD related data sets (LUAD) to explore the characteristics and regulation of 20 DNA methylation-related genes. We further identified two DNA methylation subtypes by analysing the expression profiles of these 20 DNA methylation-related genes. Subsequently, the differences in immune cell infiltration (ICI) and the expression of immune-related signaling factors among different DNA methylation subtypes were explored, and the differentially expressed genes (DEGs) among different LUAD DNA methylation subtypes were identified. Using univariate Cox to screen differentially expressed genes meaningful for survival, a DNA methylation score (DMS) was constructed based on the weight of the first and second dimensions after dimensionality reduction by principal component analysis (PCA). Our study found that DMS can better evaluate the prognosis of lung adenocarcinoma.

**Results:**

Based on DMS, LUAD samples were divided into two groups with high and low scores. The differences in clinical characteristics, tumor mutation load, and tumor immune cell infiltration between different DMS groups of LUAD were deeply explored, and the prediction ability of DMS for the benefit of immunotherapy was evaluated.

**Conclusions:**

DMS is a valuable tool for predicting survival, clinicopathological features, and immunotherapeutic efficacy, which may help to promote personalized LUAD immunotherapy in the future.

## 1. Introduction

The incidence rate of lung cancer is second in the world [1]. Importantly, the mortality of lung cancer accounts for approximately 25% of all cancer mortality and is the main cause of cancer-related death [2]. Lung adenocarcinoma (LUAD), which accounts for more than 40% of lung cancer diagnoses, is the most common histological subtype of lung cancer [3]. Despite new advances in treatment options, such as molecular targeted drugs and immune checkpoint inhibitors, the average 5-year relative survival rate of lung cancer patients is only 17% [4]. Therefore, it is urgent to find biomarkers related to the prognosis of LUAD.

As one of the most abundant and well-studied epigenetic modifications, DNA methylation plays an important role in normal development and cell biology [5, 6]. DNA methylation consists of the addition of a methyl at position 5 of cytosine to form 5-methylcytosine (5mC), and it is the main form of DNA modification in many eukaryotes [7]. DNA methylation patterns often change in cancer, including DNA hypomethylation of reverse transcription elements, centromeres, and oncogenes and DNA hypermethylation associated with inhibiting key gene regulatory elements [8]. Moreover, 5mC modification is abolished in many cancers, including acute myeloid leukemia (AML), glioma, and melanoma [9–11]. As DNA methylation-related genes are closely related to the occurrence and development of cancer, we aimed to establish a prediction model based on DNA methylation-related differentially expressed genes to provide accurate clinical guidance for patients with LUAD. Although some studies have focused on the development of prognostic models according to gene characteristics, few studies have delved into DNA methylation-related genes.

With advancements in high-throughput sequencing technology, the generation of large-scale omics data has become possible [12–14]. The characteristics of these DNA methylation-related genes can explain the etiology of cancer and have diagnostic and prognostic value. However, no prognostic features associated with DNA methylation have been established in LUAD.

In this study, DNA methylation-related genes were considered to be closely related to the progression of LUAD. By analysing the relationship between DNA methylation-related gene expression and LUAD, we established a DNA methylation score (DMS) that can better evaluate the prognosis of LUAD. This, together with the good predictive ability of the DMS, is a major improvement compared to previous studies. In the future diagnosis and treatment of LUAD, gene diagnosis and treatment will become an effective means. By detecting the expression of DNA methylation-related genes in LUAD, the model gene was transformed into a DNA methylation score, which can be used to predict the prognosis of patients.

## 2. Materials and Method

### 2.1. Data Acquisition and Preprocessing

**Figure 1:**
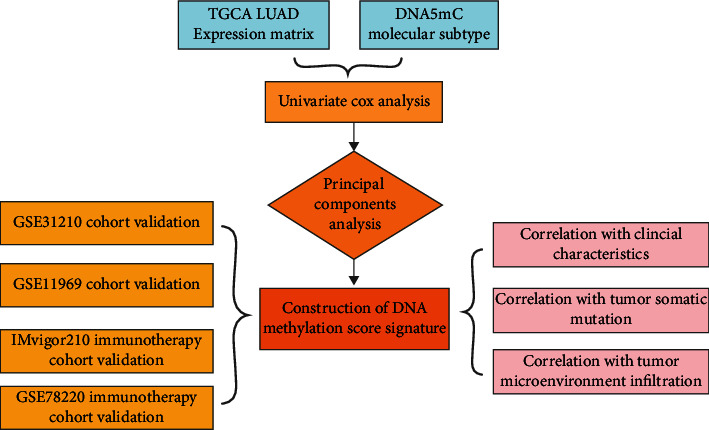
Flow chart.

First, using the TCGA database (https://portal.gdc.cancer.gLUAD/), the expression profile data of LUAD and clinical follow-up information data were downloaded. The RNA SEQ data of TCGA-LUAD were processed using the following steps: (1) samples without clinical follow-up information were removed; (2) the samples with unknown survival time, less than 30 days, and no survival state were removed; (3) the probe was converted to gene symbols; (4) one probe corresponded to multiple genes, and the probe was removed; and (5) the expression of multiple gene symbols was taken as the median value. The analysis flow chart is shown in Figure 1.

### 2.2. Consistent Clustering of Tumor DNA Methylation-Related Gene Expression Profiles

Using the consusclusterplus package in R, the PAM method based on Euclid and ward connecting rod was used for unsupervised clustering and repeated 1000 times to ensure the stability of classification.

### 2.3. Differentially Expressed Genes among Tumor DNA Methylation Subtypes

According to the expression of DNA methylation-related genes and the results of consistent clustering, tumor samples were divided into DNA methylation-1 and DNA methylation-2 groups. Using the limma package (R software), the differential gene expression between DNA methylation subtypes of TCGA-LUAD tumor samples was analysed. The screening threshold of gene differential expression was adjusted (*P* < 0.05 and |log2 (fold change)| > 1), and lncRNAs in differentially expressed genes were extracted by using the annotation file (∗.GTF) of the genome in assembly.

### 2.4. Dimensionality Reduction of Gene Characteristics and Construction of DNA Methylation-Related Score (DMS) Model

First, to reduce noise or redundant genes, the size of the DNA methylation subtype-related differentially expressed genes (DEGs) set was reduced by using a single factor Cox algorithm. After reduction, principal component analysis (PCA) was further used to reduce the dimension of variables to reduce the number of genes in the risk model. Finally, the weight values of the first dimension and the second dimension after PCA dimensionality reduction were used to construct the tumor DNA methylation score (DMS) model. The calculation formula was as follows:

(1) $$\begin{array}{c} \text{DMS}=\sum \text{P}\text{C}1\left(i\right)+\sum \text{P}\text{C}2\left(i\right). \end{array}$$

### 2.5. Gene Set Enrichment Analysis (GSEA)

First, determine the purpose of the analysis; that is, select one or more functional gene sets in MSigDB for analysis (gene matrix transposition file format ∗.GMT), and then sort based on the correlation degree between gene expression data and phenotype (which can also be understood as the change in expression). Finally, we judged whether the genes in each gene set were enriched in the upper or lower part of the gene list after phenotypic correlation ranking to judge the influence of the synergistic change of genes in this gene set on the phenotypic change.

### 2.6. Statistical Analysis and Hypothesis Testing

All statistical comparisons involved in this study and the hypothesis test of the significance of differences between groups were based on the statistical analysis method in R3.6.

## 3. Results

### 3.1. Molecular Characteristics of DNA Methylation-Related Genes in Lung Adenocarcinoma

**Figure 2:**
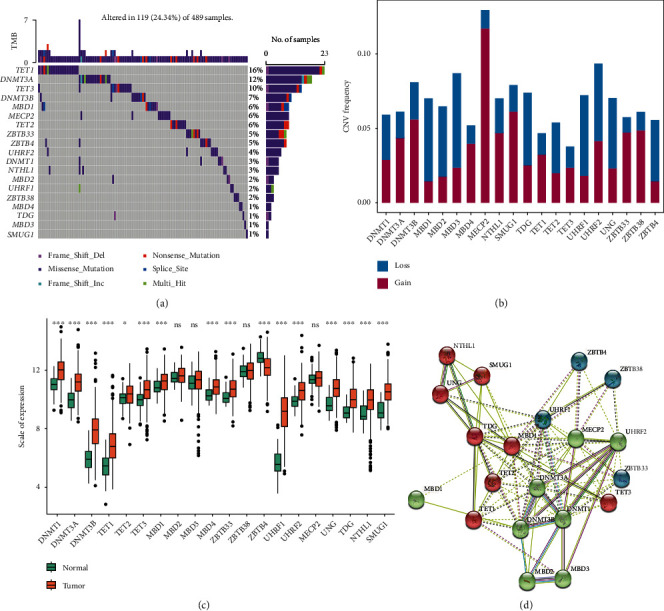
Multiomic characteristics of 20 DNA methylation-related genes in the TCGA-LUAD data set. (a) The mutation of 20 DNA methylation-related genes in LUAD and the overall mutation rate of DNA methylation-related genes in varying degrees in the genome. (b) The copy number variation of DNA methylation-related genes was counted and dominated by copy number variation. (c) At the transcriptome level, DNMT1, DNMT3A, DNMT3B, TET1, TET2, TET3, MBD1, MBD4, ZBTB33, UHRF1, UHRF2, UNG, TDG, NTHL1, and SMUG1 were significantly overexpressed in tumor tissues, while ZBTB4 was significantly downregulated in tumor tissues. (d) There were certain interactions between genes at the protein level.

After pretreatment, 489 tumor samples were included in our TCGA-LUAD data set. After a systematic review of published articles about DNA methylation and the TCGA-LUAD data set, the mutations of 20 DNA methylation-related genes (writers: *DNMT1*, *DNMT3A*, and *DNMT3B*; erasers: *TET1*, *TET2*, and *TET3*; and readers: *MBD1*, *MBD2*, *MBD3*, *MBD4*, *ZBTB33*, *ZBTB38*, *ZBTB4*, *UHRF1*, *UHRF2*, *MECP2*, *TDG*, *NTHL1*, and *SMUG1*) were counted. First, we analysed the mutations of 20 DNA methylation-related genes in LUAD (Figure 2(a)) and found that the overall mutation rate of 19 DNA methylation-related genes varied to varying degrees in the genome. DNMT3A (12%) had the highest mutation frequency among writer genes; TET1 (16%) had a higher mutation frequency than other eraser genes; and MBD1 (6%) had a high mutation frequency of reader genes. On the other hand, MBD3, MBD4, TDG, and SMUG1 exhibited extremely low mutation rates in LUAD patients (1%).

Then, the copy number variation of 20 DNA methylation-related genes was analysed (Figure 2(b)). There was a certain frequency of copy number variation in the TCGA-LUAD data set: MECP2, DNMT3A, DNMT3B, MBD4, SMUG1, ZBTB33, and ZBTB38 showed a relatively high frequency of amplification, among which the copy number variation of the *MECP2* gene was more prominent (>10%). MBD1, MBD2, MBD3, UHRF1, and ZBTB4 were mainly copy number deletions. Overall, the 20 methylation-related genes were dominated by gain copy number variation.

At the transcriptome level, the expression differences of 20 DNA methylation-related genes in normal tissues and tumor tissues were compared (Figure 2(c)). Most genes had significant expression differences, including *DNMT1*, *DNMT3A*, *DNMT3B*, *TET1*, *TET2*, *TET3*, *MBD1*, *MBD4*, *ZBTB33*, *UHRF1*, *UHRF2*, *UNG*, *TDG*, *NTHL1*, and *SMUG1*, which were significantly overexpressed in tumor tissues, while *ZBTB4* was significantly downregulated in tumor tissues. The level of protein regulation was based on the STRING database (https://www.string-db.org/). A network diagram of protein-level interactions was drawn (Figure 2(d)). It was found that there are certain interactions between genes.

In conclusion, the above results revealed that crosstalk among these DNA methylation regulators might play crucial roles in LUAD.

### 3.2. Correlation between DNA Methylation-Related Genes and Immune Cell Infiltration (ICI) in Lung Adenocarcinoma

**Figure 3:**
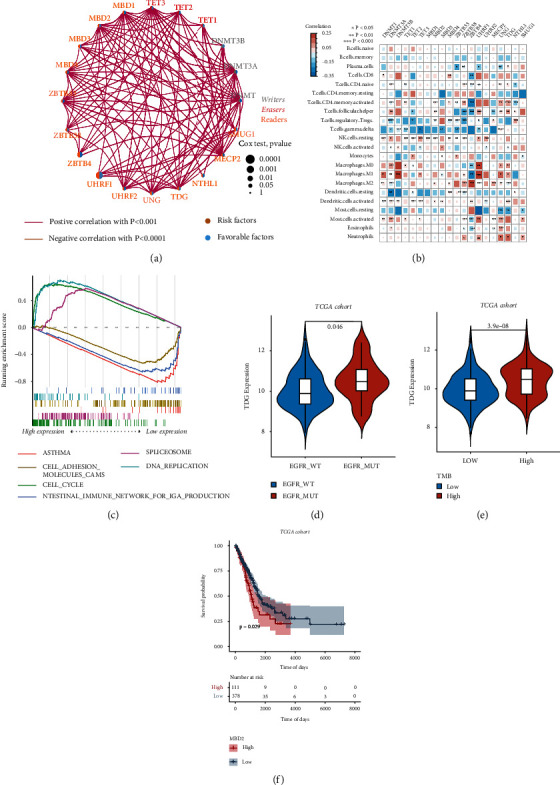
The relationship between DNA methylation genes and tumor immune cell infiltration in the TCGA-LUAD data set. (a) Gene coexpression network showing that there was a significant positive correlation between most genes. (b) Correlation analysis between the expression profiles of 20 DNA methylation genes and the infiltration of 22 kinds of immune cells. (c) Gene set enrichment analysis (GSEA) was carried out based on the high and low expression states of the TDG gene. (d) TDG gene expression was significantly higher after EGFR mutation. (e) The expression of the TDG gene in the TMB high evaluation group was significantly higher than that in the TMB low evaluation group. (f) The OS of the MBD2 low expression group was better than that of the high expression group.

To explore the relationship between the expression of DNA methylation-related genes and the tumor immune microenvironment, cibersort was used to evaluate the infiltration status of 22 immune cells in the TCGA-LUAD data set (B.cells.naive, B.cells.memory, Plasma.cells, T.cells. CD8, T.cells.CD4.naive, T.cells.CD4.memory.resting, T.cells.CD4.memory.activated, T.cells.follicular.helper, T.cells.regulatory. Tregs, T. cells.gamma.delta, NK. cells.resting, NK. cells.activated, monocytes, macrophages. M0, macrophages. M1, Macrophages. M2, Dendritic.cells.resting, Dendritic.cells.activated, Mast.cells.resting, Mast.cells.activated, Eosinophils, Neutrophils) (Table S1). First, by analysing the coexpression of DNA methylation-related genes in the TCGA-LUAD data set (Figure 3(a)), a significant positive correlation between most genes was found.

DNA methylation has been reported to play significant roles in the immune system and tumor microenvironment. Therefore, we also investigated the relationship between DNA methylation regulators and tumor immunology.

Then, the correlation analysis between the expression profiles of 20 DNA methylation genes and the infiltration of 22 kinds of immune cells (Figure 3(b)) showed great differences between different genes and the infiltration of immune cells. Among these genes, the *TDG* gene showed a significant correlation with the infiltration of most immune cells, and the *MBD2* gene had a strong correlation with T cell infiltration. Considering the relatively higher correlation between *TDG* and immune cells, we thoroughly analysed the role of *TDG* in immunotherapy. Gene set enrichment analysis (GSEA) was carried out based on the high and low expression states of the TDG gene. As shown in Figure 3(c), the main enrichment pathways of samples in the high expression state were CELL CYCLE, SPLICEOSOME, and DNA REPLICATION, which are tumor progression-related pathways, and the main enrichment pathways of samples in the low expression state were ASTHMA, CELL ADHESION MOLECULES CAMS, and INTESTINAL IMMUNE NETWORK FOR IgA PRODUCTION, which are tumor immune pathway-related pathways. Previous studies have found that EGFR mutations are associated with the diagnosis and treatment of LUAD, so this mutated gene was selected for grouping.

Furthermore, the effects of EGFR gene mutation status and tumor mutation load (TMB) on TDG gene expression in the TCGA-LUAD data set were observed. As shown in Figures 3(d) and 3(e), *TDG* gene expression was significantly higher after *EGFR* mutation. Similarly, TDG gene expression was higher in the TMB high evaluation group than in the TMB low evaluation group.

For the *MBD2* gene, the samples in the TCGA-LUAD data set were divided into the high expression and low expression groups by using the optimal density gradient method. The results showed that there was a significant difference in the survival curve between the two groups. The overall survival time (OS) of the *MBD2* low expression group was better than that of the high expression group (Figure 3(f)), suggesting that DNA methylation-related genes are closely related to the tumor immune microenvironment.

### 3.3. Identification and Functional Enrichment Analysis of DNA Methylation Subtypes in Lung Adenocarcinoma

The expression profiles of 20 DNA methylation-related genes in TCGA-LUAD sample data were clustered by consensus clustering (consumusclusterplus). The optimal number of clusters was determined according to the cumulative distribution function (CDF), and the CDF delta area curve was observed (Supplement Fig. 1A-B). Relatively stable clustering results were observed using a cluster of 2 (Supplement Fig. 1C). Further analysis of the prognostic features of these two DNA methylation subtypes revealed significant prognostic differences among them, as shown in Supplement Fig. 1D. Among the two DNA methylation subtypes, the prognosis of DNA methylation-2 was significantly better than that of DNA methylation-1, with a median survival time of 872 days. DNA methylation-1 was associated with a poor prognosis, with a median survival time of 656 days. These results suggest that these two DNA methylation subtypes may have the potential for a more accurate classification of patient prognosis.

**Figure 4:**
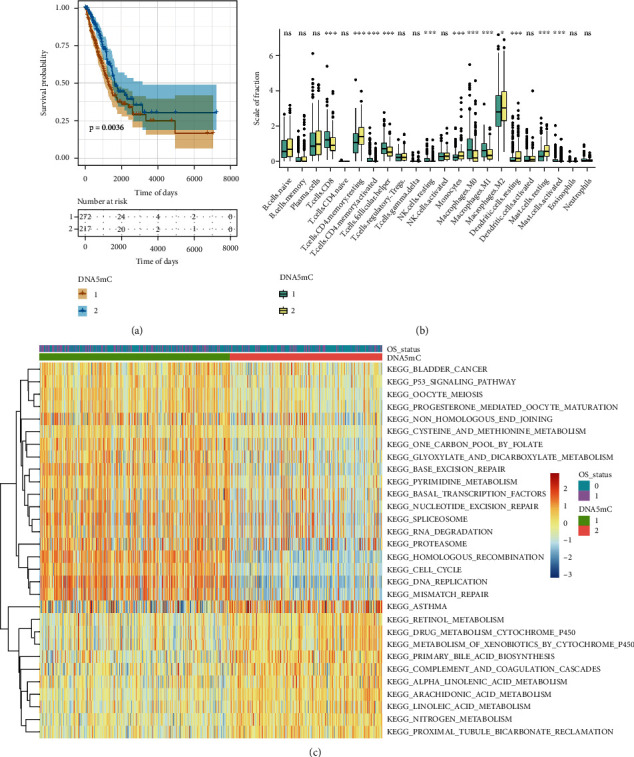
Identification and functional enrichment analysis of DNA methylation subtypes in the TCGA-LUAD data set. (a) The prognosis of the second dimension group was significantly better than that of the first dimension group; (b) Differences in immune cell infiltration among 22 tumor DNA methylation subtypes; (c) KEGG enrichment analysis of gene sets with different expressions among tumor DNA methylation subtypes.

To further explore the relationship between tumor DNA methylation subtypes and tumor immune cells, first, the principal component analysis (PCA) algorithm was used to visualize the expression profile related to DNA methylation. The samples had a good aggregation form in the space of the first and second dimensions (Supplement Fig. 2A). There was a significant difference in overall survival (OS) between the two groups (Figure 4(a)). The prognosis of the second dimension group was significantly better than that of the first dimension group, indicating that the classification method of DNA methylation subtypes is scientific and reasonable.

Then, the differences in immune cell infiltration among DNA methylation subtypes were compared (Figure 4(b)). CD8-positive middle T cells (T cell CD8), activated CD4-positive memory T cells (T cells CD4 memory activated), helper follicular T cells (T cells follicular helper), resting NK cells (NK cells), M0 macrophages, M1 macrophages, and activated mast cells significantly infiltrated at high levels in DNA methylation-1 subtypes. In the DNA methylation-2 subtype, the cells with significantly high levels of infiltration include resting CD4-positive memory T cells (resting memory CD4 T cells), monocytes, M2 macrophages (M2 macrophages), resting dendritic cells (resting dendritic cells), and resting mast cells (resting mast cells).

Furthermore, the genes were sequenced according to the status of DNA methylation subtypes, and the sequenced gene set was used for KEGG enrichment analysis (Figure 4(c)). The pathways with high enrichment scores in DNA methylation-1 subtypes were KEGG HOMOLOGOUS RECOMBINATION, KEGG CELL CYCLE, KEGG DNA REPLICATION, and KEGG MISMATCH REPAIR. Pathways with high enrichment scores in DNA methylation-2 subtypes included KEGG ASTHMA, KEGG RETINOL METABOLISM, KEGG DRUG METABOLISM CYTOCHROME P450, and KEGG METABOLISM OF XENOBIOTICS BY CYTOCHROME P450.

### 3.4. Expression of Immune-Related Factors among LUAD DNA Methylation Subtypes

**Figure 5:**
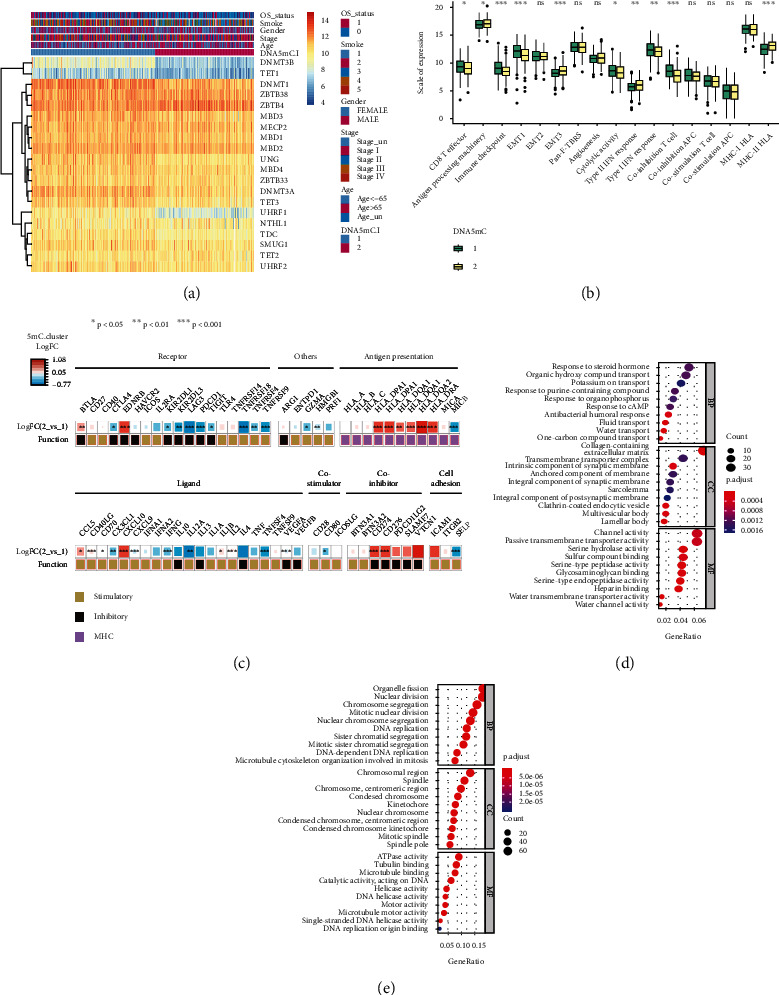
Differences in the expression of immune-related factors among tumor DNA methylation subtypes. (a) the role of each gene in the classification of DNA methylation subtypes; (b) the expression differences of various immune-related factors among DNA methylation subtypes; (c) differential expression of immune-related factors among tumor DNA methylation subtypes; (d) GO enrichment analysis of downregulated gene sets between tumor DNA methylation subtypes; (e) GO enrichment analysis of upregulated gene sets among tumor DNA methylation subtypes.

Immune-related signaling factors play an important role in the formation of the tumor immune microenvironment. It is worth further exploring the relationship between tumor DNA methylation subtypes and immune signaling factors in tumors. First, we observed the role of each gene in the classification of DNA methylation subtypes through a heatmap (Figure 5(a)). *DNMT3B*, *DNMT1*, *DNMT3A*, and *UHRF1* play a major role in the classification process. Then, by analysing the expression differences of various immune-related factors among DNA methylation subtypes (Figure 5(b)), it was found that there were significant differences in most categories. Among them, CD8 T effector, Immune checkpoint, EMT1, cytolytic activity, type I IFN response, and coinhibition T cell have high levels of activation signals in DNA methylation-1 subtypes, while antigen processing machinery, EMT3, type II IFN response, and MHC-II HLA have a high level of activation signal in DNA methylation-2 subtype. Further analysis of differential expression among subtypes of immune-related factors (Figure 5(c)) showed that most immune signaling factors were significantly differentially expressed.

To reveal the potential biological characteristics of different DNA methylation states, the differential gene expression among DNA methylation subtypes in TCGA-LUAD tumors was analysed by the limma package of R software. The screening threshold of gene differential expression was adjusted *P* < 0.05 and |log2 (fold change)| > 1, and 1396 differentially expressed genes (Table S2) were identified, of which 659 genes were highly expressed in the subtype of DNA methylation-1 and 737 genes were highly expressed in the subtype of DNA methylation-2 (Supplement Fig. 2B). Subsequently, GO functional enrichment analysis was carried out for the highly expressed genes in different DNA methylation subtypes, and the first 10 pathways enriched in the three functional classifications (BP, CC, and MF) are displayed with a bubble diagram (Figures 5(d) and 5(e)). It can be seen from the figure that most of the enriched pathways are related to nuclear division, chromosome recombination, synaptic tissue ion channels, and transmembrane transport.

Using the 1396 differentially expressed genes (DEGs) related to tumor DNA methylation subtypes, the expression profiles of differentially expressed genes (DEGs) were clustered by the consusclusterplus package in R.

Finally, the tumor samples of TCGA-LUAD were divided into two differential gene subtypes (DEG.cluster), the optimal number of clusters is determined according to the CDF, and the CDF delta area curve is observed (Supplement Fig. 3A-B). When the cluster is selected as 2 (Supplement Figure 3C), it has relatively stable clustering results. The prognostic signature among clusters was further analysed. The prognosis of C1 was significantly better than that of C2 (Supplement Fig. 3D). The results above might indicate that a relationship exists between the DEG.cluster and prognosis in LUAD patients.

### 3.5. Construction of a DNA Methylation Score (DMS) and Identification of Differential Gene Subtypes in LUAD

Based on the differentially expressed genes among DNA methylation subtypes, the principal component analysis (PCA) algorithm was used to reduce the dimension of the expression profile of differentially expressed genes. Finally, the weight of each sample in the first and second dimensions is summed as the DNA methylation score (DMS) of each sample. Then, the survminer package in R was used to calculate the optimal density gradient threshold of the tumor DNA methylation score (DMS) related to survival, and the score value of 4.75 was selected as the critical point (Supplement Fig. 4A). The tumor samples in TCGA-LUAD were divided into two groups with high and low DMS scores, and there were significant differences in survival between the two groups (Supplement Fig. 4B). The group with low DMS had a good prognosis.

**Figure 6:**
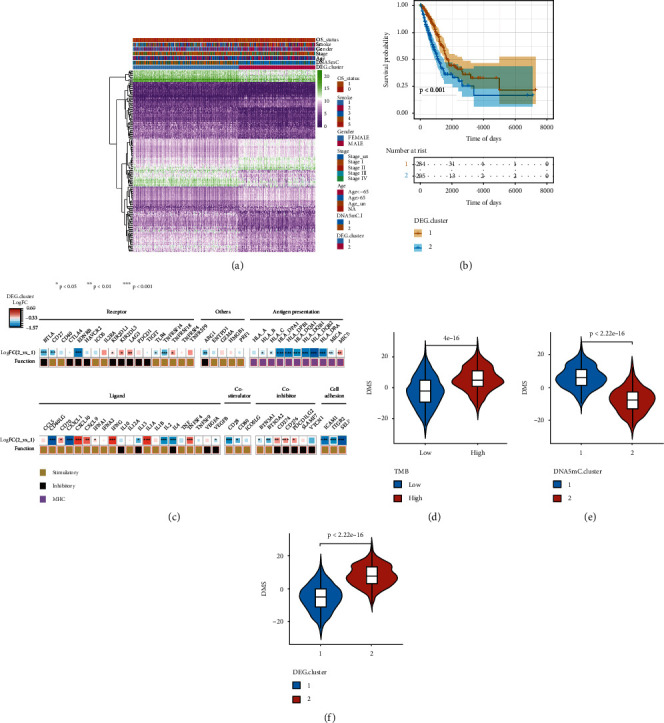
Identification and characteristic analysis of differential gene subtypes. (a) The role of 1396 differentially expressed genes in the grouping of differential gene subtypes is shown by heatmap; (b) cluster 1 group had prolonged survival time. (c) In line with the characteristics of immune cell infiltration and immune signatures, many stimulatory immunomodulators or immune checkpoint molecules were generally unregulated in DNA methylation regulator cluster 1; (d–f) the difference in DNA methylation score (DMS) between high and low groups of tumor mutation load (TMB), between DNA methylation subtypes, and between different gene subtypes (deg. cluster).

Subsequently, the molecular characteristics of different gene subtypes were explored to understand the influence of tumor DNA methylation subtypes on genome-wide expression profiles. The role of 1396 differentially expressed genes in the grouping of differential gene subtypes is shown by heatmap (Figure 6(a)), the survival of differential gene subtypes has significant differences (Figure 6(b)), and the cluster 1 group had prolonged survival time. By observing immune-related signaling factors, it was found that there were significant differences in the expression of most immune-related factors among different gene subtypes. In line with the characteristics of immune cell infiltration and immune signatures, many stimulatory immunomodulators or immune checkpoint molecules were generally unregulated in DNA methylation regulator cluster 1, indicating a relatively hot tumor immune microenvironment (Figure 6(c)). We further analysed the difference in DMS between the tumor mutation load (TMB) groups, DNA methylation subtypes, and different gene subtypes (Figures 6(d)–6(f)). There were significant differences in DMS between these groups. The relationship of the DNA methylation regulator pattern, ACRG molecular subtype, gene cluster, and DMS group is summarized in the Sankey diagram (Supplement Fig. 5A). These results may provide new ideas for the study of tumor DNA methylation status and the mechanism of gene mutation in immune checkpoints.

### 3.6. Characteristics of the DNA Methylation Score (DMS) in LUAD in the Validation Data Set

First, based on the methylation spectrum (DNA methylation-Illumina human methylation 450) of all loci in each sample of the TCGA-LUAD data set, the hypervariable loci were screened by standard variance (Supplement Fig. 6A), the methylation spectrum was standardized by using the range of gene size, and the standardized methylation spectrum of 72 genes was uniformly clustered. Finally, three independent methylation subtypes (meth.cluster) with significant survival differences were identified (Supplement Fig. 6B-C). In the three main meth.cluster subtypes, the prognosis of meth.cluster-3 was significantly better than that of meth.cluster-1/2, with a median survival of 1346 days, and meth.cluster-1/2 was associated with poor prognosis, with a median survival time of 796 days (Supplement Fig. 6D).

**Figure 7:**
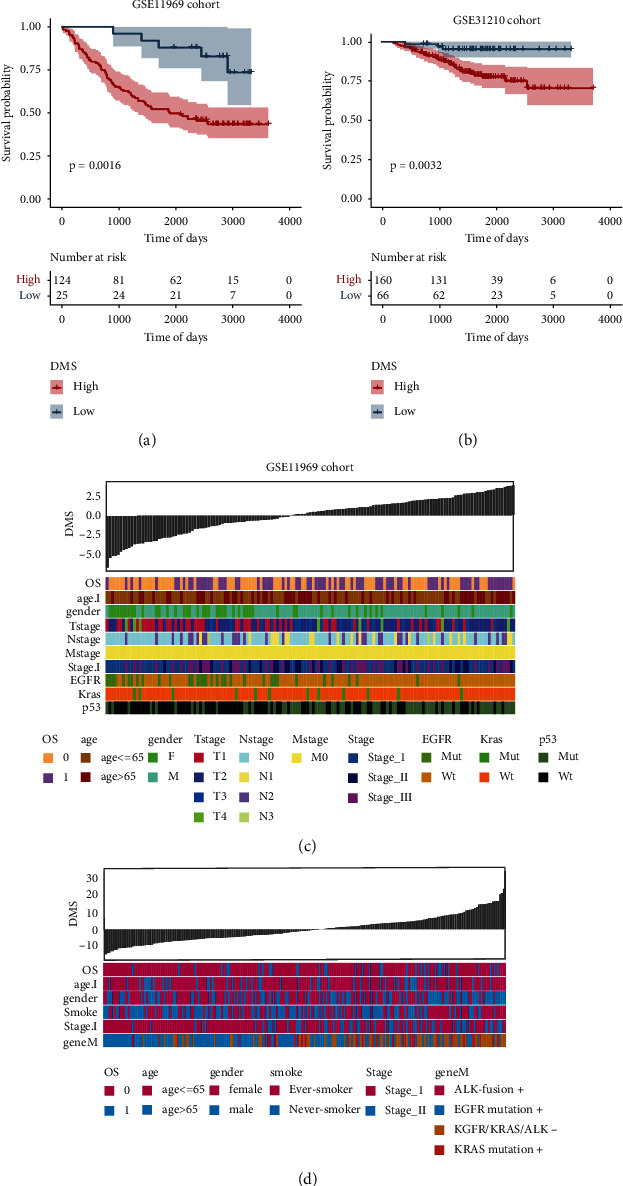
Characteristics of the tumor DNA methylation score in the external data set. (a, b) Survival curves between the high and low DMS groups in the GSE11969 and GSE31210 data sets, respectively. (c, d) The relationship between the distribution of DMS in the GSE11969 and GSE31210 data sets and the distribution of clinical special diagnoses.

To further evaluate the robustness of the DNA methylation score (DMS) based on differential gene construction to predict the overall survival of LUAD tumors, GSE11969 and GSE31210 in the GEO database were selected for analysis. First, the DNA methylation score (DMS) of the GSE11969 and GSE31210 data sets was calculated by using the differentially expressed genes screened in the early stage, and the optimal density gradient threshold of tumor DMS related to survival was calculated by using the survminer package in R. The tumor samples in the two data sets were divided into two groups with high and low DMS scores. There was a significant difference in survival between the two groups with high and low scores (Figures 7(a) and 7(b)). The heatmap was further used to show the relationship between clinical features and DMS in the two GEO data sets. It was found that DMS has a certain correlation with other clinical features (OS, age, sex, somke, stage, and Mut gene) (Figures 7(c) and 7(d)). Then, the differences in DMS between different methylation subtypes (meth.cluster) were compared. The results showed that the meth.cluster with good prognostic correlation cluster 3 had a trend of lower DMS, which is in good agreement with the previous change trend of DMS between DNA methylation subtypes and differential gene subtypes (Supplement Fig. 5B). In the TCGA-LUAD data set, the conversion between various subtypes is shown in Supplement Fig. 5C.

### 3.7. To Evaluate the Predictive Ability of the LUAD DNA Methylation Score (DMS) for the Benefit of Immunotherapy

**Figure 8:**
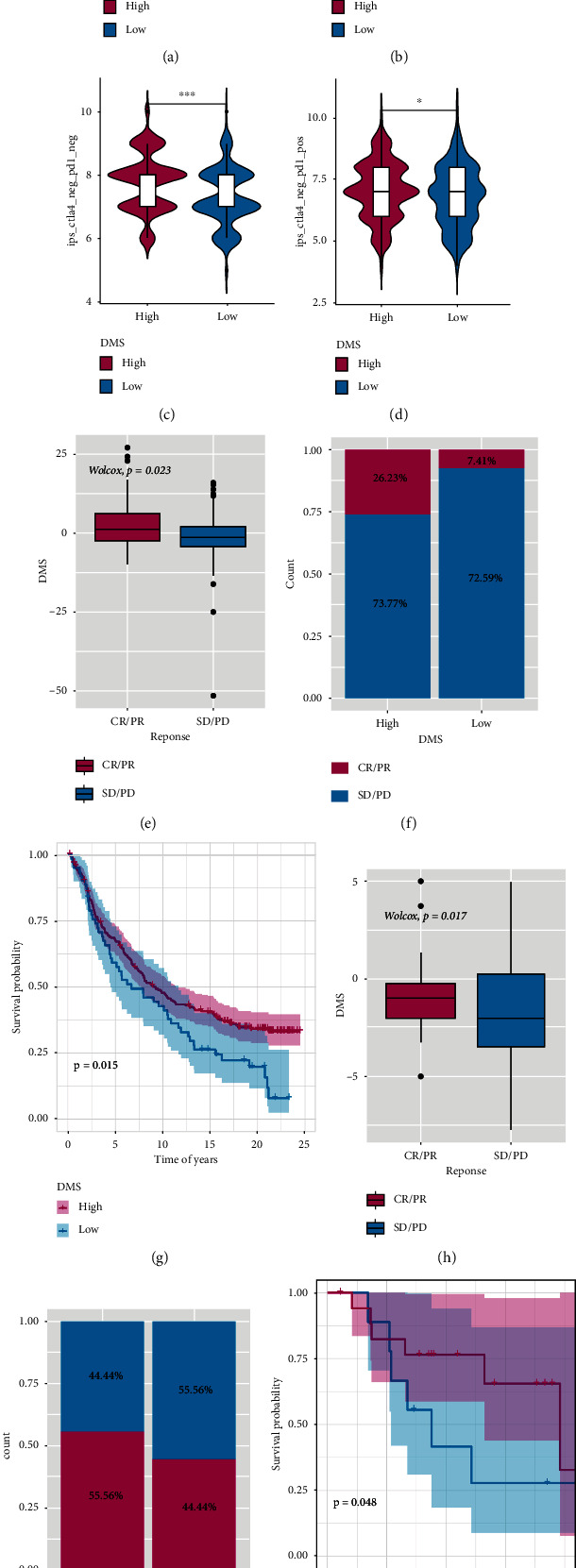
The role of the tumor DNA methylation score (DMS) in the prediction of immunotherapeutic benefits. (a–d) In the high DMS group, the IPS scores of the four types were significantly higher than those in the low DMS group. (e, f) The high DMS group was more likely to benefit from immunotherapy in the imvigor210 cohort. (g) The high DMS group lived significantly longer than the low DMS group. (h, i) In GSE78220, the objective response rate to anti-PD-L1 treatment in the high DMS group was higher than that in the low DMS group. (j) The patients with high DMS had better survival in the GSE78220 data.

To explore the predictive ability of the tumor DNA methylation score (DMS) for the benefit of immunotherapy, this study was based on the IPS score of TCGA-LUAD samples in the TCIA database and the imvigor210 data set of the immunotherapy cohort (http://researchpub.gene.com/IMvigor210CoreBiologies) and GSE78220 data sets for relevant evaluation and analysis. The Immunophenoscore (IPS) score can determine the immunogenicity of tumors and predict the response of various types of tumors to immunotherapy. In the high DNA methylation score (DMS) group, the IPS scores of the four types (ips_ctla4_neg_pd1_neg, ips_ctla4_neg_pd1_pos, ips_ctla4_pos_pd1_neg and ips_ctla4_pos_pd1_pos) were significantly higher than those in the low DNA methylation score (DMS) group (Figures 8(a)–8(d)), suggesting that patients in the high DMS group are more likely to benefit from immunotherapy. Patients receiving anti-PD-L1 immunotherapy in the imvigor210 cohort were assigned a high or low risk score (Figures 8(e) and 8(f)). The results showed that the high DMS group was more likely to benefit from immunotherapy. It is worth noting that patients in the high DMS group lived significantly longer than those in the low DMS group (Figure 8(g)). In GSE78220, the objective response rate to anti-PD-L1 treatment in the high DMS group was higher than that in the low DMS group (Figures 8(h) and 8(i)). Similarly, the patients with high DMS had better survival in the GSE78220 data (Figure 8(j)). Overall, these data suggest that the DNA methylation score of DNA methylation-related differentially expressed genes may be related to the response to immunotherapy.

Above all, the results of these four immunotherapy cohorts confirmed that DMS had the ability to efficiently predict the efficacy of immunotherapy and might achieve better predictive value when combined with TMB.

## 4. Discussion

The incidence rate and mortality rate of lung adenocarcinoma are still high. Therefore, it is urgent to identify new prognostic indicators for a more accurate prediction of prognosis in patients with lung adenocarcinoma. Although there have been some studies examining the relationship between DNA methylation and tumor formation, the relationship between DNA methylation and the prognosis of patients with lung adenocarcinoma is still very limited. In this study, the DNA methylation score (DMS) was used to predict the prognosis of patients with lung adenocarcinoma.

Specifically, we collected a data set related to TCGA-LUAD, explored the characteristics of 20 DNA methylation-related genes in the genome, transcriptome, and regulatory network, and further identified two DNA methylation subtypes by using the expression profiles of 20 DNA methylation-related genes. Subsequently, we explored the differences in the expression of immune cell infiltration (ICI) and immune-related signaling factors among different DNA methylation subtypes and identified the differentially expressed genes among different DNA methylation subtypes of LUAD. We used univariate Cox to screen differentially expressed genes (DEGs) that are meaningful for survival and constructed a DNA methylation score (DMS) based on the weights of the first and second dimensions after dimensionality reduction by principal component analysis (PCA). It was found that DMS can better evaluate the prognosis of lung adenocarcinoma. Based on DMs, LUAD tumor samples were divided into two groups with high and low scores. The differences in clinical characteristics, tumor mutation load, and tumor immune cell infiltration between different DMS groups of LUAD tumors were deeply explored, and the prediction ability of DMS for the benefit of immunotherapy was further evaluated to provide data support for accurate immunotherapy in LUAD.

DNA methylation is a form of DNA chemical modification that can change genetic performance without changing the DNA sequence [15]. The so-called DNA methylation refers to the covalent bond of the cytosine 5-carbon position of CpG dinucleotide in the genome under the action of DNA methyltransferase [16, 17]. A large number of studies have shown that DNA methylation can cause changes in chromatin structure, DNA conformation, DNA stability, and the interaction mode between DNA and protein to control gene expression [18, 19]. Changes in DNA methylation in cancer are considered to be a promising goal to develop powerful diagnostic, prognostic, and predictive biomarkers [20]. DNA methylation at position 5 of cytosine (5mC) is an epigenetic modification that regulates gene expression and cell plasticity in development and disease [21]. Studies have shown that abnormal DNA 5mC plays an important role in a variety of cancers, such as liver cancer, clear cell renal cancer, and gastric cancer, and DNA 5mC is closely related to tumor immunity [22–25].

An increasing number of research results show that the methylation of tumor DNA is closely related to the tumor immune microenvironment and tumor immunotherapy response [26–29]. DNA methylation makes cytosine easier to deaminate, resulting in C>t conversion mutations [27]. Tumors often show an overall loss of DNA methylation and obtain focal DNA methylation at CpG-rich sites. Many hot tumor mutations have been found at methylated CpG sites [30–32]. Our current results show that patients in the high DMS group lived significantly longer than those in the low DMS group. The objective response rate to anti-PD-L1 treatment in the high DMS group was higher than that in the low DMS group. Higher DNA methylation in the imvigor210 cohort was associated with an objective response to anti-PD-L1 treatment.

Although our current findings suggest that DMS can be used as an effective prognostic tool for LUAD patients, the limitations associated with this study suggest that additional analysis is needed before the clinical application of this model. First, since all samples used in our study were obtained retrospectively, prospective samples need to be included to verify our findings. Second, the focus of our analysis was only related to the prognostic value and clinical significance of DMS. DMS can better evaluate the benefit of immunotherapy in patients with LUAD, which needs to be further studied with additional in vivo and in vitro experiments.

In conclusion, we identified two different subtypes of LUAD from the perspective of DNA methylation and constructed a separate DNA methylation spectrum scoring system. DMS is a valuable tool for predicting survival, clinicopathological features, and immunotherapeutic efficacy, which may help to promote personalized LUAD immunotherapy in the future.

## Data Availability

The TCGA-LUAD data used to support the findings of this study are available from the corresponding author upon request.

## Supplementary Materials

Supplementary Materials Supplement Fig1: consistent clustering of tumor DNA methylation-related gene expression profiles. A-B: the optimal number of clusters is determined, and the CDF delta area curve is observed; C: when the cluster is selected as 2 and it has relatively stable clustering results; D: the prognosis of DNA methylation-2 was significantly better than that of DNA methylation-1. Supplement Fig2: A: the samples had a good aggregation form in the space of the first and second dimensions; B: volcanic map of differential expression analysis between tumor DNA methylation subtypes. Supplement Fig3: consistent clustering of differentially expressed gene expression profiles among tumor DNA methylation subtypes. A-B: the optimal number of clusters is determined, and the CDF delta area curve is observed; C: when the cluster is selected as 2 and it has relatively stable clustering results; D: the prognosis of C1 was significantly better than that of C2. Supplement Fig4: the best gradient grouping of the tumor DNA methylation score (DMS). A: the score value of 4.75 was selected as the critical point; B: the group with low DMS had a good prognosis. Supplement Fig5: A: the relationship of the DNA methylation regulator pattern, ACRG molecular subtype, gene cluster, and DMS group is summarized in the Sankey diagram. B: the results showed that the meth.cluster with good prognostic correlation cluster 3 had a trend of lower DMS. C: dynamic flow diagram of tumor sample grouping and state transition. Supplement Fig6: consistent clustering of gene methylation profiles in tumors. A-B: the optimal number of clusters is determined, and the CDF delta area curve is observed; C: when the cluster is selected as 3 and it has relatively stable clustering results; D: the prognosis of C3 was significantly better than that of method.cluster-1/2.
