# Supplementary material for: Identification of Prognostic DNA Methylation Signatures in Lung Adenocarcinoma
Source: Oxid Med Cell Longev. 2022 Jun 29;2022:8802303. doi: 10.1155/2022/8802303 (PMC9259289; doi:10.1155/2022/8802303)
Supplement: Supplementary Materials — Supplement Fig1: consistent clustering of tumor DNA methylation-related gene expression profiles. A-B: the optimal number of clusters is determined, and the CDF delta area curve is observed; C: when the cluster is selected as 2 and it has relatively stable clustering results; D: the prognosis of DNA methylation-2 was significantly better than that of DNA methylation-1. Supplement Fig2: A: the samples had a good aggregation form in the space of the first and second dimensions; B: volcanic map of differential expression analysis between tumor DNA methylation subtypes. Supplement Fig3: consistent clustering of differentially expressed gene expression profiles among tumor DNA methylation subtypes. A-B: the optimal number of clusters is determined, and the CDF delta area curve is observed; C: when the cluster is selected as 2 and it has relatively stable clustering results; D: the prognosis of C1 was significantly better than that of C2. Supplement Fig4: the best gradient grouping of the tumor DNA methylation score (DMS). A: the score value of 4.75 was selected as the critical point; B: the group with low DMS had a good prognosis. Supplement Fig5: A: the relationship of the DNA methylation regulator pattern, ACRG molecular subtype, gene cluster, and DMS group is summarized in the Sankey diagram. B: the results showed that the meth.cluster with good prognostic correlation cluster 3 had a trend of lower DMS. C: dynamic flow diagram of tumor sample grouping and state transition. Supplement Fig6: consistent clustering of gene methylation profiles in tumors. A-B: the optimal number of clusters is determined, and the CDF delta area curve is observed; C: when the cluster is selected as 3 and it has relatively stable clustering results; D: the prognosis of C3 was significantly better than that of method.cluster-1/2. [file 8802303.f1.zip › Table S1.pdf]

SampleID	CancerType	B.cells.naive	B.cells.memory	Plasma.cells
TCGA.L9.A444.01A.21R.A24H.07	LUAD	0.081641166	0	0.106932741
TCGA.MP.A4T9.01A.11R.A24X.07	LUAD	0.124234872	0	0.040309028
TCGA.MP.A4TC.01A.11R.A24X.07	LUAD	0.073752564	0	0.036224925
TCGA.MP.A4TA.01A.21R.A24X.07	LUAD	0.006147896	0.004224218	0.100610141
TCGA.L4.A4E5.01A.11R.A24X.07	LUAD	0.012174087	0.016290164	0.103329989
TCGA.MP.A4T8.01A.11R.A24X.07	LUAD	0.004593947	0	0.050076689
TCGA.MP.A4T7.01A.11R.A24X.07	LUAD	0.009398914	0.02137618	0.043665151
TCGA.MP.A4SW.01A.21R.A24X.07	LUAD	0.190360925	0	0.16068361
TCGA.MP.A4SY.01A.21R.A24X.07	LUAD	0.198462587	0	0.042852125
TCGA.MP.A4SV.01A.11R.A24X.07	LUAD	0.024575474	0.005582394	0.147190212
TCGA.MN.A4N1.01A.11R.A24X.07	LUAD	0	0.042911669	0.097571552
TCGA.MN.A4N5.01A.11R.A24X.07	LUAD	0.111116438	0	0.137508148
TCGA.MN.A4N4.01A.12R.A24X.07	LUAD	0.075938377	0	0.115104812
TCGA.86.A4JF.01A.11R.A24X.07	LUAD	0.014217166	0	0.0343363
TCGA.86.A4P8.01A.11R.A24X.07	LUAD	0.086082172	0	0.032159067
TCGA.93.A4JN.01A.11R.A24X.07	LUAD	0.061686846	0.090504256	0.087995414
TCGA.93.A4JP.01A.11R.A24X.07	LUAD	0.012276069	0	0.027320754
TCGA.93.A4JQ.01A.11R.A24X.07	LUAD	0.100592488	0.14616647	0
TCGA.93.A4JO.01A.21R.A24X.07	LUAD	0.113455372	0	0.0778173
TCGA.97.A4LX.01A.11R.A24X.07	LUAD	0.091598332	0	0.128347149
TCGA.97.A4M0.01A.11R.A24X.07	LUAD	0.041872621	0	0.032043749
TCGA.97.A4M1.01A.11R.A24X.07	LUAD	0	0.01708593	0.045053348
TCGA.97.A4M3.01A.11R.A24X.07	LUAD	0.139606859	0	0.136763709
TCGA.97.A4M2.01A.12R.A24X.07	LUAD	0.070023946	0	0.095087874
TCGA.97.A4M5.01A.11R.A24X.07	LUAD	0.043747708	0	0.020029173
TCGA.97.A4M7.01A.11R.A24X.07	LUAD	0.013090534	0.043831109	0.009268675
TCGA.97.A4M6.01A.11R.A24X.07	LUAD	0.035183231	0.046508444	0.009084545
TCGA.86.A4P7.01A.11R.A24X.07	LUAD	0.124499576	0	0.13095815
TCGA.44.A4SU.01A.11R.A24X.07	LUAD	0.166099274	0	0.29434106
TCGA.53.A4EZ.01A.12R.A24X.07	LUAD	0.088734465	0.028438618	0.053748121
TCGA.44.A4SS.01A.11R.A24X.07	LUAD	0.036998666	0	0.007727303
TCGA.55.A48Z.01A.12R.A24X.07	LUAD	0.134679582	0	0.156051265
TCGA.55.A494.01A.11R.A24X.07	LUAD	0	0.060812973	0.032720123
TCGA.MP.A4TK.01A.11R.A24X.07	LUAD	0.052259052	0.030654276	0.033455016
TCGA.MP.A4TI.01A.21R.A24X.07	LUAD	0.062116521	0	0.027566841
TCGA.L9.A443.01A.12R.A24H.07	LUAD	0.147915678	0	0.14886776
TCGA.L4.A4E6.01A.11R.A24H.07	LUAD	0.073697695	0	0.027800674
TCGA.86.A4D0.01A.11R.A24H.07	LUAD	0.059842804	0	0.036924433
TCGA.62.A470.01A.11R.A24H.07	LUAD	0	0.012545217	0.102944786
TCGA.62.A472.01A.11R.A24H.07	LUAD	0.028387702	0	0.03279953
TCGA.86.A456.01A.11R.A24H.07	LUAD	0	0.012963428	0.057765765
TCGA.J2.A4AD.01A.11R.A24H.07	LUAD	0.185320716	0	0.161318437
TCGA.91.A4BD.01A.11R.A24H.07	LUAD	0	0.021114187	0.035294753
TCGA.J2.A4AE.01A.21R.A24H.07	LUAD	0.220427702	0	0.180793993
TCGA.J2.A4AG.01A.11R.A24H.07	LUAD	0.151370253	0	0.068867988
TCGA.44.A479.01A.31R.A24H.07	LUAD	0.050881728	0	0.159858595
TCGA.38.A44F.01A.11R.A24H.07	LUAD	0	0.100673721	0.043250286
TCGA.44.A47B.01A.11R.A24H.07	LUAD	0.007064784	0.014805061	0.021163234
TCGA.44.A47G.01A.21R.A24H.07	LUAD	0.024566113	0.075910548	0.068998899
TCGA.55.A4DF.01A.11R.A24H.07	LUAD	0.079353153	0	0.060334393
TCGA.55.A492.01A.11R.A24H.07	LUAD	0.072345574	0	0.37097768
TCGA.55.A4DG.01A.11R.A24H.07	LUAD	0.157361493	0.059781043	0.153070547
TCGA.62.A460.01A.11R.A24H.07	LUAD	0	0.033625875	0.520103212

TCGA.62.A46P.01A.11R.A24H.07	LUAD	0.212732407	0	0.217122854
TCGA.62.A46R.01A.11R.A24H.07	LUAD	0.063700884	0	0.05224133
TCGA.62.8397.01A.11R.2326.07	LUAD	0.006103165	0.008619909	0.050096828
TCGA.50.8457.01A.11R.2326.07	LUAD	0.082018463	0	0.247123644
TCGA.62.8395.01A.11R.2326.07	LUAD	0.159268271	0	0.209534097
TCGA.62.8399.01A.21R.2326.07	LUAD	0.164846341	0	0.136962736
TCGA.62.8394.01A.11R.2326.07	LUAD	0.029910794	0.008603874	0.049781606
TCGA.55.8302.01A.11R.2326.07	LUAD	0.010405971	0.043771346	0.021169558
TCGA.95.8494.01A.11R.2326.07	LUAD	0.006971971	0.011050961	0.027918229
TCGA.86.8358.01A.11R.2326.07	LUAD	0.140960365	0	0.232255338
TCGA.86.8359.01A.11R.2326.07	LUAD	0.149708543	0	0.126948279
TCGA.69.8453.01A.12R.2326.07	LUAD	0.045430002	0	0
TCGA.50.8460.01A.11R.2326.07	LUAD	0	0.05036951	0.186267128
TCGA.62.A46V.01A.11R.A24H.07	LUAD	0	0.065283492	0.000312606
TCGA.62.A46Y.01A.11R.A24H.07	LUAD	0.048260307	0	0.021113397
TCGA.62.A46U.01A.11R.A24H.07	LUAD	0.009658413	0.005108185	0.00374273
TCGA.97.8552.01A.11R.2403.07	LUAD	6.21E-05	0.088612928	0.013135938
TCGA.97.8176.01A.11R.2403.07	LUAD	0.063576522	0	0.018675067
TCGA.91.8499.01A.11R.2403.07	LUAD	0.044666607	0	0.016665034
TCGA.91.8497.01A.11R.2403.07	LUAD	0.078928519	0.029340009	0.231253842
TCGA.86.8673.01A.11R.2403.07	LUAD	0	0.048260233	0.106576461
TCGA.86.8674.01A.21R.2403.07	LUAD	0.094095732	0	0.201254425
TCGA.86.8672.01A.21R.2403.07	LUAD	0.006565446	0.016361069	0.048595484
TCGA.86.8669.01A.11R.2403.07	LUAD	0.122930893	0	0.138302981
TCGA.86.8671.01A.11R.2403.07	LUAD	0.065737906	0.027431125	0.056204312
TCGA.86.8668.01A.11R.2403.07	LUAD	0.006797269	0.064615229	0.133097536
TCGA.86.8585.01A.11R.2403.07	LUAD	0.0411838	0.049270278	0.013252345
TCGA.78.8662.01A.11R.2403.07	LUAD	0.000731618	0.036053443	0.169845668
TCGA.91.8496.01A.11R.2403.07	LUAD	0	0.034282349	0
TCGA.50.5045.01A.01R.1628.07	LUAD	0.038866182	0.03669639	0.062441606
TCGA.97.8547.01A.11R.2403.07	LUAD	0	0.015357568	0.01849789
TCGA.55.8508.01A.11R.2403.07	LUAD	0.035635378	0	0.35341764
TCGA.78.8660.01A.11R.2403.07	LUAD	0.027320264	0.018324906	0.023445101
TCGA.78.8648.01A.11R.2403.07	LUAD	0.015513653	0.009136287	0.150839745
TCGA.78.8640.01A.11R.2403.07	LUAD	0	0.0352437	0.098019919
TCGA.71.8520.01A.11R.2403.07	LUAD	0.187165805	0	0.085154979
TCGA.55.8620.01A.11R.2403.07	LUAD	0.014332533	0.045726922	0.060966797
TCGA.55.8616.01A.11R.2403.07	LUAD	0.124839981	0.061976901	0.083099788
TCGA.55.8615.01A.11R.2403.07	LUAD	0.007259859	0.035120641	0.096753628
TCGA.55.8512.01A.11R.2403.07	LUAD	0.055536115	0	0.078041377
TCGA.55.8513.01A.11R.2403.07	LUAD	0.03306983	0.052482092	0.043455543
TCGA.55.8614.01A.11R.2403.07	LUAD	0.012542339	0.019533054	0.041051016
TCGA.55.8514.01A.11R.2403.07	LUAD	0	0.044197227	0.190916183
TCGA.55.8619.01A.11R.2403.07	LUAD	0.051025294	0.055881	0.016923283
TCGA.55.8511.01A.11R.2403.07	LUAD	0.101957272	0	0.136351224
TCGA.55.8510.01A.11R.2403.07	LUAD	0.073665498	0	0.042706328
TCGA.55.8507.01A.11R.2403.07	LUAD	0.008489471	0.008900065	0.058527725
TCGA.55.8506.01A.11R.2403.07	LUAD	0.011835793	0.005078028	0.042670971
TCGA.55.8505.01A.11R.2403.07	LUAD	0	0.054837213	0.063293167
TCGA.78.8655.01A.11R.2403.07	LUAD	0.042929821	0	0.011085957
TCGA.55.8621.01A.11R.2403.07	LUAD	0.073212554	0	0.118448478
TCGA.86.8280.01A.11R.2287.07	LUAD	0.037572125	0.012574364	0.045468134
TCGA.86.8278.01A.11R.2287.07	LUAD	0.129463095	0	0.148467953
TCGA.69.8253.01A.11R.2287.07	LUAD	0.130731163	0	0.296530633

TCGA.93.8067.01A.11R.2287.07	LUAD	0.005517222	0.008048529	0.062645611
TCGA.97.8171.01A.11R.2287.07	LUAD	0.05459193	0	0.119446313
TCGA.69.8254.01A.11R.2287.07	LUAD	0	0.064311034	0.10513804
TCGA.69.8255.01A.11R.2287.07	LUAD	0.159339622	0	0.116146729
TCGA.55.8299.01A.11R.2287.07	LUAD	0.030121279	0	0.034868601
TCGA.97.8179.01A.11R.2287.07	LUAD	0	0.010915415	0.017947621
TCGA.86.8281.01A.11R.2287.07	LUAD	0.074523716	0	0.229414083
TCGA.55.8301.01A.11R.2287.07	LUAD	0.016588455	0.040567092	0.148000026
TCGA.97.8174.01A.11R.2287.07	LUAD	0.095925226	0	0.107824192
TCGA.83.5908.01A.21R.2287.07	LUAD	0.10062243	0	0.021148974
TCGA.86.8279.01A.11R.2287.07	LUAD	0.006714009	0.026644095	0.04887608
TCGA.97.8172.01A.11R.2287.07	LUAD	0.010419843	0.188228621	0.007769342
TCGA.97.8175.01A.11R.2287.07	LUAD	0	0.043433283	0.009282713
TCGA.73.4666.01A.01R.1206.07	LUAD	0.016342214	0	0.002723227
TCGA.75.5147.01A.01R.1628.07	LUAD	0.05280094	0	0.070096814
TCGA.80.5608.01A.31R.1949.07	LUAD	0.110975364	0	0.076020674
TCGA.75.7027.01A.11R.1949.07	LUAD	0.026233741	0	0.066437224
TCGA.91.6847.01A.11R.1949.07	LUAD	0.040371358	0	0.056489818
TCGA.67.6215.01A.11R.1755.07	LUAD	0	0.02847712	0.021712186
TCGA.44.2657.01A.01R.1107.07	LUAD	0.113737945	0.009326867	0.140668397
TCGA.71.6725.01A.11R.1858.07	LUAD	0.02528475	0	0.046816232
TCGA.55.6987.01A.11R.1949.07	LUAD	0.032750621	0.035959557	0.102475519
TCGA.55.6978.01A.11R.1949.07	LUAD	0.02566128	0	0.005816847
TCGA.50.6595.11A.01R.1858.07	LUAD	0.010798891	0	0.004131783
TCGA.44.2657.11A.01R.1758.07	LUAD	0.208819547	0.014117249	0.040466605
TCGA.55.6986.11A.01R.1949.07	LUAD	0.033700887	0	0.009496402
TCGA.44.2661.11A.01R.1758.07	LUAD	0.096237623	0	0.022535913
TCGA.97.8177.01A.11R.2287.07	LUAD	0.051688357	0	0.038226112
TCGA.99.8032.01A.11R.2241.07	LUAD	0.090419483	0	0.356912223
TCGA.99.8028.01A.11R.2241.07	LUAD	0.027411893	0	0.119168121
TCGA.99.8025.01A.11R.2241.07	LUAD	0.215528941	0	0.138506718
TCGA.55.8089.01A.11R.2241.07	LUAD	0.102803585	0	0.067065834
TCGA.55.8204.01A.11R.2241.07	LUAD	0.001388893	0	0.233785196
TCGA.55.8097.01A.11R.2241.07	LUAD	0.156504867	0	0.077915571
TCGA.86.8056.01A.11R.2241.07	LUAD	0.141018368	0	0.074957377
TCGA.86.8073.01A.11R.2241.07	LUAD	0.004508767	0.046409694	0.015557312
TCGA.86.6851.01A.11R.1949.07	LUAD	0.00435767	0.033433681	0.121677206
TCGA.86.8076.01A.31R.2241.07	LUAD	0.033937256	0.023580729	0.16292289
TCGA.44.8120.01A.11R.2241.07	LUAD	0.045399781	0	0.083945051
TCGA.55.8090.01A.11R.2241.07	LUAD	0.083568604	0	0.101701293
TCGA.55.7284.01B.11R.2241.07	LUAD	0.063979462	0	0.03936457
TCGA.44.8119.01A.11R.2241.07	LUAD	0.094696741	0	0.224505432
TCGA.55.8205.01A.11R.2241.07	LUAD	0.068619221	0	0.050773362
TCGA.55.8094.01A.11R.2241.07	LUAD	0.030054415	0.03882703	0.103824229
TCGA.55.8206.01A.11R.2241.07	LUAD	0.052918628	0	0.046052621
TCGA.55.7913.01B.11R.2241.07	LUAD	0.144200625	0	0.121681067
TCGA.86.8075.01A.11R.2241.07	LUAD	0.028226929	0	0.073962183
TCGA.55.8092.01A.11R.2241.07	LUAD	0	0.062087914	0.202600344
TCGA.86.8074.01A.11R.2241.07	LUAD	0.00649519	0.007964996	0.018165417
TCGA.95.8039.01A.11R.2241.07	LUAD	0.010221147	0.028315724	0.018205096
TCGA.J2.8194.01A.11R.2241.07	LUAD	0.081133418	0	0.093472156
TCGA.99.8033.01A.11R.2241.07	LUAD	0	0.015250728	0.059298724
TCGA.J2.8192.01A.11R.2241.07	LUAD	0.012048187	0.023935789	0.005819259
TCGA.86.8055.01A.11R.2241.07	LUAD	0.021947787	0	0.048872389

TCGA.55.8096.01A.11R.2241.07	LUAD	0.002260251	0.032146998	0.089371178
TCGA.44.8117.01A.11R.2241.07	LUAD	0.139869601	0	0.089152666
TCGA.86.8054.01A.11R.2241.07	LUAD	0.017483968	0.029273562	0.024715168
TCGA.05.4395.01A.01R.1206.07	LUAD	0.00522697	0.007221961	0.016393879
TCGA.05.4389.01A.01R.1206.07	LUAD	0.019085934	0	0.00145074
TCGA.38.4631.01A.01R.1755.07	LUAD	0.187668212	0	0.153146159
TCGA.44.6145.01A.11R.1755.07	LUAD	0.106946	0	0.159628192
TCGA.38.4625.01A.01R.1206.07	LUAD	0.061100438	0	0.08376879
TCGA.55.6983.01A.11R.1949.07	LUAD	0.043334645	0.177425361	0
TCGA.91.6831.11A.02R.1858.07	LUAD	0.018502222	0	0
TCGA.44.2655.11A.01R.1758.07	LUAD	0.065992268	0	0.007273778
TCGA.44.2665.11A.01R.1758.07	LUAD	0.022797754	0	0.000327497
TCGA.38.4632.01A.01R.1755.07	LUAD	0.035129523	0.002143845	0.020251853
TCGA.05.4382.01A.01R.1206.07	LUAD	0.035664884	0.006949578	0.046981351
TCGA.05.4384.01A.01R.1755.07	LUAD	0.080790717	0	0.085652947
TCGA.05.4390.01A.02R.1755.07	LUAD	0	0.016599956	0.046652188
TCGA.38.6178.01A.11R.1755.07	LUAD	0.050739804	0	0.124811422
TCGA.05.4425.01A.01R.1755.07	LUAD	0.016808264	0.014888604	0
TCGA.55.8091.01A.11R.2241.07	LUAD	0.127170367	0	0.05586199
TCGA.95.7562.01A.11R.2241.07	LUAD	0.204324627	0	0.135509214
TCGA.55.8208.01A.11R.2241.07	LUAD	0.041705453	0.032728581	0.119042919
TCGA.55.8085.01A.11R.2241.07	LUAD	0.082320448	0	0.12660938
TCGA.55.8203.01A.11R.2241.07	LUAD	0.12205436	0	0.187990664
TCGA.55.8207.01A.11R.2241.07	LUAD	0.037257394	0	0.033436296
TCGA.55.8087.01A.11R.2241.07	LUAD	0	0.037950367	0.046749534
TCGA.62.8398.01A.11R.2326.07	LUAD	0.094713177	0	0.135917494
TCGA.50.8459.01A.11R.2326.07	LUAD	0.065164068	0	0.115430746
TCGA.62.8402.01A.11R.2326.07	LUAD	0.02103292	0	0.027745379
TCGA.55.A493.01A.11R.A24H.07	LUAD	0.136944373	0	0.067813959
TCGA.91.A4BC.01A.11R.A24H.07	LUAD	0.116066161	0	0.164701897
TCGA.44.A47A.01A.21R.A24H.07	LUAD	0.040375579	0	0.02952564
TCGA.55.A48X.01A.11R.A24H.07	LUAD	0.057290178	0.217546402	0.040707728
TCGA.55.A491.01A.11R.A24H.07	LUAD	0.186034178	0	0.211054222
TCGA.62.A471.01A.12R.A24H.07	LUAD	0.203722524	0	0.173084741
TCGA.44.7670.01A.11R.2066.07	LUAD	0.007732609	0.075094144	0.009249341
TCGA.53.7626.01A.12R.2066.07	LUAD	0.085330715	0	0.090231275
TCGA.44.7671.01A.11R.2066.07	LUAD	0.181374786	0	0.159850096
TCGA.44.7660.01A.11R.2066.07	LUAD	0	0.071844029	0.047216927
TCGA.44.7662.01A.11R.2066.07	LUAD	0.066239071	0	0.096971552
TCGA.44.7661.01A.11R.2066.07	LUAD	0.051334177	0	0
TCGA.86.7953.01A.11R.2187.07	LUAD	0.045191948	0	0.029611432
TCGA.95.7948.01A.11R.2187.07	LUAD	0.146419378	0	0.189021857
TCGA.97.7941.01A.11R.2187.07	LUAD	0.125698001	0	0.028785996
TCGA.93.7347.01A.11R.2187.07	LUAD	0.057285869	0	0.069244594
TCGA.44.7672.01A.11R.2066.07	LUAD	0.027609246	0.036139466	0.070209809
TCGA.55.7576.01A.11R.2066.07	LUAD	0.022709119	0.005318644	0.035565839
TCGA.53.7624.01A.11R.2066.07	LUAD	0.118913453	0	0.127607587
TCGA.78.7162.01A.21R.2066.07	LUAD	0.107752136	0	0.127837498
TCGA.86.7954.01A.11R.2187.07	LUAD	0.069117754	0	0.021887601
TCGA.86.7955.01A.11R.2187.07	LUAD	0.196262466	0	0.115016104
TCGA.95.7944.01A.11R.2187.07	LUAD	0.023953901	0	0.001744306
TCGA.64.1679.01A.21R.2066.07	LUAD	0.129954189	0	0.051958739
TCGA.95.7947.01A.11R.2187.07	LUAD	0.138886342	0	0.156892049
TCGA.64.1681.01A.11R.2066.07	LUAD	0.004891552	0.046191431	0.03082882

TCGA.73.7499.01A.11R.2187.07	LUAD	0.089555555	0	0.146039342
TCGA.69.7980.01A.11R.2187.07	LUAD	0.025639117	0.000668794	0.009725576
TCGA.69.7979.01A.11R.2187.07	LUAD	0.081759492	0	0.103583327
TCGA.69.7978.01A.11R.2187.07	LUAD	0.035291459	0.006129628	0.049403588
TCGA.55.7994.01A.11R.2187.07	LUAD	0.05896365	0.004873569	0
TCGA.69.7973.01A.11R.2187.07	LUAD	0.089666456	0	0.063195164
TCGA.44.7667.01A.31R.2066.07	LUAD	0.007540635	0.005709063	0.003266178
TCGA.55.7728.01A.11R.2187.07	LUAD	0.008155363	0.049510677	0.008589137
TCGA.55.7995.01A.11R.2187.07	LUAD	0.033098416	0.048464937	0
TCGA.44.7669.01A.21R.2066.07	LUAD	0.072554572	0	0.039348613
TCGA.55.7725.01A.11R.2170.07	LUAD	0.045913361	0.042498609	0.011849186
TCGA.50.5066.02A.11R.2090.07	LUAD	0.035006087	0.001993008	0.122556723
TCGA.50.5946.02A.11R.2090.07	LUAD	0.143524721	0	0.216301251
TCGA.50.5933.01A.11R.1755.07	LUAD	0.024068136	0	0.011962048
TCGA.50.5946.01A.11R.1755.07	LUAD	0.151893396	0	0.196156545
TCGA.55.7727.01A.11R.2170.07	LUAD	0.133350852	0.013172097	0.059987385
TCGA.55.7815.01A.11R.2170.07	LUAD	0.018047246	0	0.034709915
TCGA.55.7726.01A.11R.2170.07	LUAD	0.106590854	0	0.073469583
TCGA.53.7813.01A.11R.2170.07	LUAD	0.113295174	0	0.373586137
TCGA.55.7724.01A.11R.2170.07	LUAD	0.071973527	0	0.039241795
TCGA.69.7764.01A.11R.2170.07	LUAD	0.270176517	0	0.207703413
TCGA.86.7714.01A.12R.2170.07	LUAD	0.053829827	0	0.010083568
TCGA.97.7937.01A.11R.2170.07	LUAD	0.011979431	0	0.102501346
TCGA.38.4626.11A.01R.1758.07	LUAD	0.010682383	0	0.003022348
TCGA.38.4627.11A.01R.1758.07	LUAD	0.033870779	0	0.001877426
TCGA.44.3396.11A.01R.1758.07	LUAD	0.027804256	0	0
TCGA.44.2668.11A.01R.1758.07	LUAD	0.055747083	0	0.006588492
TCGA.69.7765.01A.11R.2170.07	LUAD	0.041748424	0.005620009	0.193876865
TCGA.44.7659.01A.11R.2066.07	LUAD	0.070534111	0.040623483	0.122407638
TCGA.91.7771.01A.11R.2170.07	LUAD	0.093165749	0	0.139698077
TCGA.69.7763.01A.11R.2170.07	LUAD	0.136689598	0	0.18758575
TCGA.69.7760.01A.11R.2170.07	LUAD	0.031714458	0.001763604	0.040431055
TCGA.69.7761.01A.11R.2170.07	LUAD	0	0.056595306	0.129724589
TCGA.55.7910.01A.11R.2170.07	LUAD	0	0.068430654	0.20917161
TCGA.55.7911.01A.11R.2170.07	LUAD	0.04687249	0	0.000629456
TCGA.55.7903.01A.11R.2170.07	LUAD	0.090784644	0	0.025332544
TCGA.86.7701.01A.11R.2170.07	LUAD	0.14078284	0	0.213549719
TCGA.55.7907.01A.11R.2170.07	LUAD	0.124600011	0	0.124166699
TCGA.97.7938.01A.11R.2170.07	LUAD	0.107249026	0	0.034928531
TCGA.78.7167.01A.11R.2066.07	LUAD	0.08534912	0	0.145895834
TCGA.78.7163.01A.12R.2066.07	LUAD	0.25342605	0	0.152686101
TCGA.78.7535.01A.11R.2066.07	LUAD	0	0.041583521	0.031541988
TCGA.78.7539.01A.11R.2066.07	LUAD	0.071317708	0	0.036553037
TCGA.78.7537.01A.11R.2066.07	LUAD	0.092620461	0	0.273104595
TCGA.78.7542.01A.21R.2066.07	LUAD	0.054638826	0	0.08408836
TCGA.86.7713.01A.11R.2066.07	LUAD	0.046555113	0	0.138240438
TCGA.78.7540.01A.11R.2066.07	LUAD	0.006059199	0.055597834	0.010729813
TCGA.78.7633.01A.11R.2066.07	LUAD	0.012753456	0.000392963	0.014897085
TCGA.95.7567.01A.11R.2066.07	LUAD	0.075485101	0	0.186819393
TCGA.78.7166.01A.12R.2066.07	LUAD	0.009686096	0.108739213	0.040344041
TCGA.73.7498.01A.12R.2187.07	LUAD	0.012660955	0.013485546	0.125197022
TCGA.69.7974.01A.11R.2187.07	LUAD	0.018942691	0.004966985	0.024049868
TCGA.55.7283.01A.11R.2039.07	LUAD	0.018759427	0	0.003034836
TCGA.97.7547.01A.11R.2039.07	LUAD	0.068357384	0	0.08960717

TCGA.78.7156.01A.11R.2039.07	LUAD	0.084659861	0	0.055022577
TCGA.78.7147.01A.11R.2039.07	LUAD	0.070647819	0	0.033529938
TCGA.55.7281.01A.11R.2039.07	LUAD	0.031926371	0	0.033496414
TCGA.78.7148.01A.11R.2039.07	LUAD	0.064250292	0	0.058206763
TCGA.55.6642.01A.11R.1858.07	LUAD	0.0097417	0	0.214514894
TCGA.44.3396.01A.01R.1206.07	LUAD	0.019309335	0.01301755	0.077957975
TCGA.49.4486.01A.01R.1206.07	LUAD	0.147079534	0	0.272929603
TCGA.49.4494.01A.01R.1206.07	LUAD	0.025742219	0	0.038568103
TCGA.86.6562.01A.11R.1755.07	LUAD	0.015277088	0.016171571	0.001838137
TCGA.05.4396.01A.21R.1858.07	LUAD	0.008313749	0.040193487	0.038199207
TCGA.49.4501.01A.01R.1206.07	LUAD	0.127659956	0	0.115666759
TCGA.75.6211.01A.11R.1755.07	LUAD	0.028061628	0.021042848	0.146265057
TCGA.05.4415.01A.22R.1858.07	LUAD	0.067541399	0	0.081803459
TCGA.50.5939.01A.11R.1628.07	LUAD	0.000544251	0.07184807	0.094624988
TCGA.75.6207.01A.11R.1755.07	LUAD	0.002724204	0.0216876	0.005654449
TCGA.05.4405.01A.21R.1858.07	LUAD	0.109327601	0	0.176330998
TCGA.05.4410.01A.21R.1858.07	LUAD	0.091315639	0	0.32860742
TCGA.55.5899.01A.11R.1628.07	LUAD	0.00786647	0	0.010631342
TCGA.44.6774.01A.21R.1858.07	LUAD	0.032751651	0	0.05803827
TCGA.50.5055.01A.01R.1628.07	LUAD	0.031155945	0.156459344	0.101644143
TCGA.55.6970.01A.11R.1949.07	LUAD	0.017759879	0.018556864	0.06305927
TCGA.75.6214.01A.41R.1949.07	LUAD	0	0	0.014456265
TCGA.55.6981.01A.11R.1949.07	LUAD	0.071000171	0.034141442	0.017743761
TCGA.55.6980.01A.11R.1949.07	LUAD	0.092602686	0	0.046150983
TCGA.75.6206.01A.11R.1755.07	LUAD	0.002747608	0.001959292	0.013498072
TCGA.38.4628.01A.01R.1206.07	LUAD	0.013120938	0	0.001773137
TCGA.38.4630.01A.01R.1206.07	LUAD	0.040964503	0	0.071355769
TCGA.91.6830.01A.11R.1949.07	LUAD	0.042463637	0	0.012960687
TCGA.75.5125.01A.01R.1755.07	LUAD	0.016954924	0	0.005330627
TCGA.75.6212.01A.11R.1755.07	LUAD	0.129452695	0	0.144601945
TCGA.75.6203.01A.11R.1755.07	LUAD	0.023697577	0	0.029850715
TCGA.75.5126.01A.01R.1755.07	LUAD	0.029043624	0	0.001648661
TCGA.50.5066.01A.01R.1628.07	LUAD	0	0.095496075	0.032304424
TCGA.75.6205.01A.11R.1755.07	LUAD	0.025781904	0.003906356	0
TCGA.50.5936.01A.11R.1628.07	LUAD	0.016372073	0.012543386	0.023445869
TCGA.50.5068.01A.01R.1628.07	LUAD	0.011517198	0.078978567	0.039766205
TCGA.78.7145.01A.11R.2039.07	LUAD	0.111687198	0	0.028249464
TCGA.78.7154.01A.11R.2039.07	LUAD	0.056077158	0	0.007669466
TCGA.78.7220.01A.11R.2039.07	LUAD	0.148211823	0	0.043019253
TCGA.78.7153.01A.11R.2039.07	LUAD	0.034052002	0.031440249	0.146526981
TCGA.97.7546.01A.11R.2039.07	LUAD	0.052970463	0.004987409	0
TCGA.78.7155.01A.11R.2039.07	LUAD	0.054767287	0	0.026526129
TCGA.93.7348.01A.21R.2039.07	LUAD	0.233703268	0	0.186710414
TCGA.78.7146.01A.11R.2039.07	LUAD	0.010445891	0	0
TCGA.78.7536.01A.11R.2066.07	LUAD	0.039773048	0	0.058607102
TCGA.86.7711.01A.11R.2066.07	LUAD	0.01040578	0.004067895	0.024388599
TCGA.78.7150.01A.21R.2039.07	LUAD	0.090910672	0	0.04660339
TCGA.50.7109.01A.11R.2039.07	LUAD	0.125129858	0	0.211750987
TCGA.55.7573.01A.11R.2039.07	LUAD	0.184570004	0	0.082704432
TCGA.97.7554.01A.11R.2039.07	LUAD	0.027467469	0	0.088803038
TCGA.78.7160.01A.11R.2039.07	LUAD	0.079672008	0	0.032427636
TCGA.78.7143.01A.11R.2039.07	LUAD	0.004386132	0.066569463	0.01007961
TCGA.55.7227.01A.11R.2039.07	LUAD	0.012708851	0	0.008523695
TCGA.38.4625.11A.01R.1758.07	LUAD	0.045318664	0	0.016472055

TCGA.50.5939.11A.01R.1628.07	LUAD	0.010194803	0.001653317	0.002907528
TCGA.44.2662.11A.01R.1758.07	LUAD	0.052629856	0	0.011491003
TCGA.55.7570.01A.11R.2039.07	LUAD	0.171358902	0	0.309554616
TCGA.78.7149.01A.11R.2039.07	LUAD	0.16439885	0	0.341273161
TCGA.55.6985.11A.01R.1949.07	LUAD	0.088002308	0	0.029456646
TCGA.73.4676.11A.01R.1755.07	LUAD	0.020533787	0	0
TCGA.55.6984.11A.01R.1949.07	LUAD	0.010358761	0	0.011731549
TCGA.55.6972.11A.01R.1949.07	LUAD	0.05464172	0	0.048416033
TCGA.55.6968.11A.01R.1949.07	LUAD	0.042454042	0.055481485	0.020048778
TCGA.55.6980.11A.01R.1949.07	LUAD	0.108205058	0	0.069022334
TCGA.50.5936.11A.01R.1628.07	LUAD	0.024094315	0	0.00149296
TCGA.78.7158.01A.11R.2039.07	LUAD	0.040248186	0	0.096084938
TCGA.97.7552.01A.11R.2039.07	LUAD	0.09275999	0.028980016	0.16019539
TCGA.97.7553.01A.21R.2039.07	LUAD	0.084471566	0	0.046999517
TCGA.78.7159.01A.11R.2039.07	LUAD	0.12204731	0	0.26928555
TCGA.75.7030.01A.11R.1949.07	LUAD	0.222542497	0	0.134836081
TCGA.55.6982.01A.11R.1949.07	LUAD	0.022269086	0.027348161	0.055992098
TCGA.38.4627.01A.01R.1206.07	LUAD	0.050151047	0	0.026192593
TCGA.38.4629.01A.02R.1206.07	LUAD	0.023433865	0	0.04192272
TCGA.44.5644.01A.21R.2039.07	LUAD	0.215005421	0	0.307580858
TCGA.55.6972.01A.11R.1949.07	LUAD	0.090868824	0	0.178100593
TCGA.55.6971.01A.11R.1949.07	LUAD	0.026450398	0.044100139	0.106610916
TCGA.91.6849.01A.11R.1949.07	LUAD	0.085469043	0	0.212769899
TCGA.75.7031.01A.11R.1949.07	LUAD	0.099358623	0	0.124640979
TCGA.55.6984.01A.11R.1949.07	LUAD	0.066671402	0.09750309	0.054576201
TCGA.78.7152.01A.11R.2039.07	LUAD	0.065272573	0.047835959	0.105211806
TCGA.55.7574.01A.11R.2039.07	LUAD	0.15876943	0.03107552	0.115172488
TCGA.91.6848.01A.11R.1949.07	LUAD	0.04851333	0	0.061503848
TCGA.95.7043.01A.11R.1949.07	LUAD	0.135582184	0	0.222879439
TCGA.38.7271.01A.11R.2039.07	LUAD	0	0.221645334	0.022960328
TCGA.55.6982.11A.01R.1949.07	LUAD	0.042720801	0	0.013743962
TCGA.55.6975.11A.01R.1949.07	LUAD	0.127576746	0	0.053033269
TCGA.55.6979.11A.01R.1949.07	LUAD	0.073442465	0	0.015941248
TCGA.55.6978.11A.01R.1949.07	LUAD	0.011161646	0	0.002611326
TCGA.49.6761.11A.01R.1949.07	LUAD	0.017689432	0	0.002458473
TCGA.91.6847.11A.01R.1949.07	LUAD	0.073058526	0	0.030334951
TCGA.91.6836.11A.01R.1858.07	LUAD	0.030637965	0	0.006116341
TCGA.55.6983.11A.01R.1949.07	LUAD	0.010136133	0	0.001151157
TCGA.91.6840.01A.11R.1949.07	LUAD	0.174205917	0	0.108228909
TCGA.99.7458.01A.11R.2039.07	LUAD	0.093324634	0	0.019767739
TCGA.78.7161.01A.11R.2039.07	LUAD	0.151281362	0	0.198172664
TCGA.44.5645.01A.01R.1628.07	LUAD	0.114176099	0	0.046245327
TCGA.50.6593.01A.11R.1755.07	LUAD	0.024634516	0.018353552	0.027424917
TCGA.44.5643.01A.01R.1628.07	LUAD	0.122327181	0	0.073689809
TCGA.67.6216.01A.11R.1755.07	LUAD	0.072531118	0.034935748	0.075061042
TCGA.50.6594.01A.11R.1755.07	LUAD	0.008165884	0.022166764	0.030748693
TCGA.35.5375.01A.01R.1628.07	LUAD	0.012864296	0.048442271	0.004911222
TCGA.50.6592.01A.11R.1755.07	LUAD	0.160937675	0	0.101740682
TCGA.50.5944.01A.11R.1755.07	LUAD	0.118416941	0	0.039391373
TCGA.55.6543.01A.11R.1755.07	LUAD	0.104969532	0	0.111087253
TCGA.05.4430.01A.02R.1206.07	LUAD	0.019262553	0	0.026437955
TCGA.05.4434.01A.01R.1206.07	LUAD	0.047321231	0.011596793	0
TCGA.50.6591.01A.11R.1755.07	LUAD	0	0.015169023	0.002098158
TCGA.05.4432.01A.01R.1206.07	LUAD	0.08585473	0	0.191082019

TCGA.44.5645.11A.01R.1628.07	LUAD	0.056081403	0	0.044579501
TCGA.50.5930.11A.01R.1755.07	LUAD	0.105711611	0	0.023986303
TCGA.44.6144.11A.01R.1755.07	LUAD	0.008485863	0	0.00310539
TCGA.38.4632.11A.01R.1755.07	LUAD	0.039400241	0	0.019519642
TCGA.05.5420.01A.01R.1628.07	LUAD	0.03166352	0	0.0132263
TCGA.05.5423.01A.01R.1628.07	LUAD	0.027563676	0.022996306	0.01904199
TCGA.50.5931.01A.11R.1755.07	LUAD	0.031274775	0.02731141	0.098251454
TCGA.50.5930.01A.11R.1755.07	LUAD	0.03559909	0	0.26162299
TCGA.44.6148.01A.11R.1755.07	LUAD	0.136797642	0	0.232572993
TCGA.05.4398.01A.01R.1206.07	LUAD	0.061250351	0	0.115557376
TCGA.05.4402.01A.01R.1206.07	LUAD	0.047627639	0	0.067753545
TCGA.05.4397.01A.01R.1206.07	LUAD	0.012524432	0	0.049434936
TCGA.73.4676.01A.01R.1755.07	LUAD	0.018229148	0	0.002114246
TCGA.67.3770.01A.01R.0946.07	LUAD	0.089624557	0	0.030920784
TCGA.50.5049.01A.01R.1628.07	LUAD	0.041822522	0	0.146882755
TCGA.05.4403.01A.01R.1206.07	LUAD	0.108240848	0	0.088967777
TCGA.50.5932.01A.11R.1755.07	LUAD	0.039023611	0	0.121899577
TCGA.44.6146.01A.11R.1755.07	LUAD	0.014143948	0	0.029930998
TCGA.44.2655.01A.01R.0946.07	LUAD	0.130127158	0.006328619	0.144012878
TCGA.44.2659.01A.01R.0946.07	LUAD	0.103353799	0	0.153690311
TCGA.44.2656.01A.02R.0946.07	LUAD	0.067142279	0	0.138913289
TCGA.49.4510.01A.01R.1206.07	LUAD	0.17747931	0	0.367164554
TCGA.73.4658.01A.01R.1755.07	LUAD	0.069731813	0	0.076166287
TCGA.49.6767.01A.11R.1858.07	LUAD	0.014774488	0.010711377	0
TCGA.91.6829.01A.21R.1858.07	LUAD	0.181741926	0	0.031222626
TCGA.67.3772.01A.01R.0946.07	LUAD	0.021841169	0.008546675	0.127794615
TCGA.73.4662.01A.01R.1206.07	LUAD	0.064236754	0.018494163	0.148323211
TCGA.73.4668.01A.01R.1206.07	LUAD	0.148129367	0	0.258846813
TCGA.67.3771.01A.01R.0946.07	LUAD	0.104918591	0	0.166237602
TCGA.50.6595.01A.12R.1858.07	LUAD	0.022349675	0	0.002537983
TCGA.49.4514.01A.21R.1858.07	LUAD	0.069685137	0.012363149	0.020650066
TCGA.38.4625.01A.01R.1206.07	LUAD	0.077603909	0	0.069399756
TCGA.38.4626.01A.01R.1206.07	LUAD	0.024846876	0	0.009742482
TCGA.67.4679.01B.01R.1755.07	LUAD	0.006620795	0.005203754	0.028529742
TCGA.67.6217.01A.11R.1755.07	LUAD	0	0.069152359	0.086420368
TCGA.75.5122.01A.01R.1755.07	LUAD	0.006933489	0.000414142	0.001900503
TCGA.91.6828.11A.01R.1858.07	LUAD	0.010704438	0	0.006650209
TCGA.91.6829.11A.01R.1858.07	LUAD	0.025136527	0	0.011980527
TCGA.49.4490.11A.01R.1858.07	LUAD	0.039967264	0	1.67E-05
TCGA.50.6597.01A.11R.1858.07	LUAD	0	0.046057744	0.117378116
TCGA.44.6778.01A.11R.1858.07	LUAD	0.036166133	0.034063001	0.005115206
TCGA.64.5815.01A.01R.1628.07	LUAD	0.048773583	0	0.073861264
TCGA.75.5146.01A.01R.1628.07	LUAD	0.09000821	0.011836285	0.204260325
TCGA.49.4512.01A.21R.1858.07	LUAD	0.010990833	0.058078439	0.018882949
TCGA.49.4487.01A.21R.1858.07	LUAD	0.090395083	0.087741448	0.042064307
TCGA.44.6779.01A.11R.1858.07	LUAD	0.020970076	0	0.004098381
TCGA.73.4675.01A.01R.1206.07	LUAD	0.012244829	0.036434225	0.011829044
TCGA.64.5774.01A.01R.1628.07	LUAD	0.144839712	0	0.280782497
TCGA.05.4424.01A.22R.1858.07	LUAD	0.078512447	0	0.076981804
TCGA.49.4507.01A.01R.1206.07	LUAD	0.03916716	0	0.084291912
TCGA.64.5775.01A.01R.1628.07	LUAD	0.029167784	0	0.038467461
TCGA.91.6836.01A.21R.1858.07	LUAD	0.022770551	0.008010242	0.080749132
TCGA.73.4670.01A.01R.1206.07	LUAD	0.098574057	0	0.172091831
TCGA.44.6775.01A.11R.1858.07	LUAD	0.127956179	0	0.065674871

TCGA.91.6828.01A.11R.1858.07	LUAD	0.078161253	0	0.07292912
TCGA.44.6776.01A.11R.1858.07	LUAD	0.031697887	0	0.133450057
TCGA.64.5778.01A.01R.1628.07	LUAD	0.060081627	0.016152539	0.107329025
TCGA.73.4677.01A.01R.1206.07	LUAD	0.043114976	0	0.202647761
TCGA.80.5611.01A.01R.1628.07	LUAD	0	0.027960458	0.004524519
TCGA.44.6777.01A.11R.1858.07	LUAD	0.049214385	0	0.090089889
TCGA.49.6742.01A.11R.1858.07	LUAD	0.068037784	0	0.021643691
TCGA.50.5051.01A.21R.1858.07	LUAD	0.176271207	0	0.218555925
TCGA.64.1676.01A.01R.0946.07	LUAD	0.056857595	0	0.081956628
TCGA.44.2668.01A.01R.0946.07	LUAD	0.021300691	0	0.024861284
TCGA.50.5072.01A.21R.1858.07	LUAD	0.1484488	0	0.182640386
TCGA.91.6835.01A.11R.1858.07	LUAD	0.041899724	0.083956521	0
TCGA.55.1592.01A.01R.0946.07	LUAD	0.128440242	0	0.12818322
TCGA.49.6761.01A.31R.1949.07	LUAD	0.059223185	0	0.086764954
TCGA.64.1677.01A.01R.0946.07	LUAD	0.044953417	0.059243362	0.162916429
TCGA.50.6590.01A.12R.1858.07	LUAD	0.007719673	0.025922453	0.074827957
TCGA.73.4659.01A.01R.1206.07	LUAD	0.038301005	0	0.054910084
TCGA.49.4506.01A.01R.1206.07	LUAD	0.026178996	0.028551456	0.016598128
TCGA.05.4417.01A.22R.1858.07	LUAD	0.047513612	0	0.124540777
TCGA.05.4427.01A.21R.1858.07	LUAD	0.039302594	0	0.13279197
TCGA.64.5781.01A.01R.1628.07	LUAD	0.131756254	0	0.162073689
TCGA.49.6744.01A.11R.1858.07	LUAD	0.067627589	0	0.131618325
TCGA.49.6743.01A.11R.1858.07	LUAD	0.11500068	0	0.146309873
TCGA.55.1596.01A.01R.0946.07	LUAD	0.050081232	0	0.070910784
TCGA.55.1594.01A.01R.0946.07	LUAD	0.083034211	0	0.024886247
TCGA.49.4505.01A.01R.1206.07	LUAD	0.0571005	0	0.163685051
TCGA.50.5932.11A.01R.1755.07	LUAD	0.018931237	0	0.006171597
TCGA.50.5933.11A.01R.1755.07	LUAD	0.098780471	0	0.024522982
TCGA.44.6777.11A.01R.1858.07	LUAD	0.010609565	0.0081401	0.027902246
TCGA.44.6145.11A.01R.1858.07	LUAD	0.034681112	0	0.008135743
TCGA.49.4512.11A.01R.1858.07	LUAD	0.034103648	0	0.06460925
TCGA.49.6742.11A.01R.1858.07	LUAD	0.08695517	0	0.08283587
TCGA.91.6835.11A.01R.1858.07	LUAD	0.034276794	0.006753187	0.022525914
TCGA.44.6778.11A.01R.1858.07	LUAD	0.032855179	0	0.006426808
TCGA.44.6776.11A.01R.1858.07	LUAD	0.008564141	0	0
TCGA.64.1678.01A.01R.0946.07	LUAD	0.104379279	0	0.376157006
TCGA.49.6743.11A.01R.1858.07	LUAD	0.062890142	0	0.012909765
TCGA.44.6148.11A.01R.1858.07	LUAD	0.109834187	0	0.048389809
TCGA.91.6849.11A.01R.1949.07	LUAD	0.109091308	0	0.056054327
TCGA.44.6147.11A.01R.1858.07	LUAD	0.044149259	0	0.019200287
TCGA.44.6146.11A.01R.1858.07	LUAD	0.03698078	0	0.002305639
TCGA.50.5931.11A.01R.1858.07	LUAD	0.00945976	0.01496091	0.00035298
TCGA.50.5935.11A.01R.1858.07	LUAD	0.003778765	0	0.001474571
TCGA.55.6969.11A.01R.1949.07	LUAD	0	0.121127884	0.016759311
TCGA.55.6981.11A.01R.1949.07	LUAD	0	0.011000735	0.009650024
TCGA.49.6745.11A.01R.1858.07	LUAD	0.000669798	0	0
TCGA.05.5715.01A.01R.1628.07	LUAD	0.052487443	0	0.034191058
TCGA.05.4422.01A.01R.1206.07	LUAD	0.108384325	0	0.292359167
TCGA.67.3774.01A.01R.0946.07	LUAD	0.153652494	0	0.145822306
TCGA.05.5429.01A.01R.1628.07	LUAD	0.243427467	0	0.18700361
TCGA.05.4420.01A.01R.1206.07	LUAD	0.226656115	0	0.236620553
TCGA.05.5425.01A.02R.1628.07	LUAD	0.085262056	0	0.066312009
TCGA.50.5935.01A.11R.1755.07	LUAD	0.022949536	0.017534167	0.05923151
TCGA.05.5428.01A.01R.1628.07	LUAD	0	0.019023379	0.00961232

TCGA.50.5942.01A.21R.1755.07	LUAD	0.175677672	0	0.258613824
TCGA.50.5941.01A.11R.1755.07	LUAD	0.102679978	0	0.080645826
TCGA.05.4426.01A.01R.1206.07	LUAD	0.144135694	0	0.037837739
TCGA.80.5607.01A.31R.1949.07	LUAD	0	0.026098107	0.01874691
TCGA.55.1595.01A.01R.0946.07	LUAD	0.134892544	0	0.146715589
TCGA.44.2665.01A.01R.0946.07	LUAD	0	0.010206878	0.006467153
TCGA.55.6975.01A.11R.1949.07	LUAD	0.185363778	0	0.238791813
TCGA.05.4433.01A.22R.1858.07	LUAD	0.012198895	0.023583553	0
TCGA.67.3773.01A.01R.0946.07	LUAD	0.089828424	0	0.07698607
TCGA.55.6986.01A.11R.1949.07	LUAD	0.073866361	0	0.070836605
TCGA.55.6985.01A.11R.1949.07	LUAD	0.195902655	0	0.097868568
TCGA.95.7039.01A.11R.1949.07	LUAD	0.135184677	0	0.05721194
TCGA.64.5779.01A.01R.1628.07	LUAD	0.080329112	0	0.322823822
TCGA.44.2662.01A.01R.0946.07	LUAD	0.036766009	0	0.083483551
TCGA.44.2666.01A.01R.0946.07	LUAD	0.092649742	0	0.078159335
TCGA.49.4490.01A.21R.1858.07	LUAD	0.08821162	0	0.029670535
TCGA.50.5044.01A.21R.1858.07	LUAD	0.016102831	0	0.104058874
TCGA.05.4418.01A.01R.1206.07	LUAD	0.167300749	0	0.13578661
TCGA.49.4488.01A.01R.1755.07	LUAD	0.025172721	0.004117717	0.051188478
TCGA.91.6831.01A.11R.1858.07	LUAD	0	0.003540496	0.077052273
TCGA.55.6712.01A.11R.1858.07	LUAD	0.135589128	0	0.115352298
TCGA.05.4244.01A.01R.1107.07	LUAD	0	0.020772396	0.023529688
TCGA.05.4250.01A.01R.1107.07	LUAD	0.076331739	0	0.0223519
TCGA.44.3398.01A.01R.1107.07	LUAD	0	0.069870077	0.01708457
TCGA.44.4112.01A.01R.1107.07	LUAD	0.036838678	0	0.111896165
TCGA.44.3918.01A.01R.1107.07	LUAD	0.021548315	0	0.088089168
TCGA.05.4249.01A.01R.1107.07	LUAD	0.010003222	0.030142849	0.022884049
TCGA.44.2661.01A.01R.1107.07	LUAD	0.061671332	0	0.111119572
TCGA.35.4122.01A.01R.1107.07	LUAD	0.012683943	0.003817292	0.048291912
TCGA.35.4123.01A.01R.1107.07	LUAD	0.029835513	0	0.019668539
TCGA.44.3919.01A.02R.1107.07	LUAD	0.05886553	0	0.03277569
TCGA.50.6673.01A.11R.1949.07	LUAD	0.035111714	0	0.055298797
TCGA.55.6968.01A.11R.1949.07	LUAD	0.046310797	0	0.177491187
TCGA.55.6979.01A.11R.1949.07	LUAD	0.071921458	0	0.115659456
TCGA.55.6969.01A.11R.1949.07	LUAD	0.035522497	0	0.159450689
TCGA.75.7025.01A.12R.1949.07	LUAD	0.122094619	0	0.067920523
TCGA.55.6971.11A.01R.1949.07	LUAD	0.02043571	0	0.017823025
TCGA.55.6970.11A.01R.1949.07	LUAD	0.079456526	0	0.019570762
TCGA.64.1680.01A.02R.0946.07	LUAD	0.113221937	0	0.081238113
TCGA.49.6745.01A.11R.1858.07	LUAD	0.008527541	0.002252237	0.000766891
TCGA.35.3615.01A.01R.0946.07	LUAD	0.191795914	0	0.155804768
TCGA.44.6147.01A.11R.1755.07	LUAD	0.10834511	0.042623189	0.18762529
TCGA.49.6744.11A.01R.1858.07	LUAD	0.061695561	0	0.023454232
TCGA.55.7914.01A.11R.2170.07	LUAD	0.093034517	0	0.26528121
TCGA.MP.A4T6.01A.32R.A262.07	LUAD	0.179134879	0	0.084646797
TCGA.MP.A4TD.01A.32R.A262.07	LUAD	0.061853236	0.069246294	0.215400311
TCGA.MP.A4T4.01A.11R.A262.07	LUAD	0.058305213	0	0.035354595
TCGA.NJ.A55A.01A.11R.A262.07	LUAD	0.10359899	0.070739504	0.06915928
TCGA.NJ.A55O.01A.11R.A262.07	LUAD	0.132235076	0	0.046347699
TCGA.NJ.A4YI.01A.11R.A262.07	LUAD	0.004067132	0.006321769	0.044817769
TCGA.MP.A4TJ.01A.51R.A262.07	LUAD	0.077658443	0.002957554	0.098200866
TCGA.MP.A5C7.01A.11R.A262.07	LUAD	0.033492547	0.000311978	0.135246611
TCGA.O1.A52J.01A.11R.A262.07	LUAD	0.006729966	0	0
TCGA.69.A59K.01A.11R.A262.07	LUAD	0	0.102128474	0.135944307

TCGA.MP.A4TF.01A.11R.A262.07	LUAD	0.088006225	0	0.341358877
TCGA.MP.A4TH.01A.31R.A262.07	LUAD	0.083241388	0.125366141	0.130324751
TCGA.NJ.A4YQ.01A.11R.A262.07	LUAD	0.021229321	0.116806534	0.037479714
TCGA.NJ.A4YF.01A.12R.A262.07	LUAD	0.175497395	0	0.139163559
TCGA.95.A4VP.01A.21R.A262.07	LUAD	0.112533799	0.028971404	0.097790968
TCGA.95.A4VK.01A.11R.A262.07	LUAD	0.17580695	0.087324876	0
TCGA.95.A4VN.01A.11R.A262.07	LUAD	0.026165477	0.028321108	1.33E-05
TCGA.NJ.A55R.01A.11R.A262.07	LUAD	0	0.200783452	0.193833109
TCGA.62.A46S.01A.11R.A24H.07	LUAD	0.155437156	0	0.193333456
TCGA.NJ.A4YP.01A.11R.A262.07	LUAD	0.020849896	0	0.182043219
TCGA.44.6775.01C.02R.A277.07	LUAD	0.031542086	0.077138987	0.024736304
TCGA.44.3918.01A.01R.A278.07	LUAD	0.015034793	0	0.038270266
TCGA.44.2656.01A.02R.A278.07	LUAD	0.076816985	0	0.131175702
TCGA.44.4112.01A.01R.A278.07	LUAD	0.015756979	0	0.089227504
TCGA.44.2665.01B.06R.A277.07	LUAD	0.005898713	0.021110898	0
TCGA.44.2668.01A.01R.A278.07	LUAD	0.006966767	0	0.05173781
TCGA.44.2666.01A.01R.A278.07	LUAD	0.06679054	0	0.051461579
TCGA.44.6775.01A.11R.A278.07	LUAD	0.12475759	0	0.093045158
TCGA.44.6147.01A.11R.A278.07	LUAD	0.244237421	0	0.153482214
TCGA.44.2665.01A.01R.A278.07	LUAD	0	0.051587209	0.003393307
TCGA.44.2662.01A.01R.A278.07	LUAD	0.027899539	0	0.058411384
TCGA.44.6146.01A.11R.A278.07	LUAD	0	0.006801064	0.023638848
TCGA.44.5645.01A.01R.A278.07	LUAD	0.029700789	0.058226878	0.04681963
TCGA.44.3917.01A.01R.A278.07	LUAD	0.012667054	0.01799474	0.143876389
TCGA.44.6147.01B.06R.A277.07	LUAD	0.156912156	0.142895574	0
TCGA.44.2666.01B.02R.A277.07	LUAD	0.007788634	0.007499392	0.115047828
TCGA.44.4112.01B.06R.A277.07	LUAD	0.039433897	0.039403189	0.071943482
TCGA.44.3918.01B.02R.A277.07	LUAD	0.021481411	0.013881709	0.01438733
TCGA.44.3917.01B.02R.A277.07	LUAD	0.048935323	0.065732493	0.044103307
TCGA.44.2668.01B.02R.A277.07	LUAD	0.033248819	0.010070013	0.010378191
TCGA.44.6146.01B.04R.A277.07	LUAD	0	0.044238924	0.003137119
TCGA.44.2662.01B.02R.A277.07	LUAD	0	0.018817217	0.007436652
TCGA.44.5645.01B.04R.A277.07	LUAD	0.065405128	0.037182886	0
TCGA.44.2656.01B.06R.A277.07	LUAD	0.043287161	0	0.015199241
TCGA.49.AARQ.01A.11R.A41B.07	LUAD	0.02009978	0	0.151427407
TCGA.49.AARN.01A.21R.A41B.07	LUAD	0.030860595	0.116411458	0.186890598
TCGA.49.AARO.01A.12R.A41B.07	LUAD	0.0879268	0.026522426	0.067651232
TCGA.49.AAR4.01A.12R.A41B.07	LUAD	0.019777492	0.014536302	0.048356787
TCGA.55.A57B.01A.12R.A39D.07	LUAD	0.013196134	0.048293378	0.027758847
TCGA.99.AA5R.01A.11R.A39D.07	LUAD	0.069770807	0.084446561	0.087509371
TCGA.S2.AA1A.01A.12R.A39D.07	LUAD	0.121343877	0	0.204055707
TCGA.49.AARR.01A.11R.A41B.07	LUAD	0.169214558	0	0.108425619
TCGA.49.AAR3.01A.11R.A41B.07	LUAD	0.007474791	0.018157237	0.076670969
TCGA.73.A9RS.01A.11R.A41B.07	LUAD	0.215590846	0	0.227867515
TCGA.L9.A743.01A.43R.A39D.07	LUAD	0.040040302	0.052029336	0.131406657
TCGA.L9.A7SV.01A.11R.A39D.07	LUAD	0	0.07966518	0.163609366
TCGA.L9.A8F4.01A.11R.A39D.07	LUAD	0.093731973	0	0.155069153
TCGA.NJ.A7XG.01A.12R.A39D.07	LUAD	0	0.046254957	0.100072059
TCGA.49.AAR9.01A.21R.A41B.07	LUAD	0.012179898	0.016626896	0.022578477
TCGA.49.AARE.01A.11R.A41B.07	LUAD	0.136739809	0	0.163441103
TCGA.49.AAQV.01A.11R.A39D.07	LUAD	0.097201313	0.036565327	0.051945281
TCGA.49.AAR2.01A.11R.A39D.07	LUAD	0.116851391	0	0.312110243
TCGA.4B.A93V.01A.11R.A39D.07	LUAD	0.065689136	0	0.243792691
TCGA.49.AAR0.01A.21R.A39D.07	LUAD	0.005390951	0.098034049	0.052742518

TCGA.55.A48Y.01A.11R.A24H.07	LUAD	0.056263226	0	0.175039523
TCGA.L9.A5IP.01A.21R.A39D.07	LUAD	0.045480041	0	0.274803333
TCGA.L9.A50W.01A.12R.A39D.07	LUAD	0.108527605	0	0.186824172
TCGA.55.A490.01A.11R.A466.07	LUAD	0.07133997	0	0.191183613
TCGA.NJ.A4YG.01A.22R.A262.07	LUAD	0.02436013	0	0.056581265
TCGA.MP.A4TE.01A.22R.A466.07	LUAD	0.205856041	0	0.395535637
TCGA.55.7816.01A.11R.2170.07	LUAD	0.006280673	0.000505808	0.002958153
TCGA.44.3398.11B.01R.1758.07	LUAD	0	0.003649079	0.00470831

T.cells.CD8	T.cells.CD4.naive	T.cells.CD4.memory.resting
0.200428807	0	0.11595608
0.131142559	0	0.017014491
0.089592995	0	0.131088508
0.164437171	0	0.163521795
0.153431712	0	0.164744295
0.037827104	0	0
0.084768775	0	0.090937715
0.082046931	0	0.104335581
0.033047714	0	0.077687534
0.145479695	0	0.178141824
0.043350079	0	0.008483015
0.159460169	0	0.018069434
0.056052808	0	0.045523881
0.098916328	0	0.097024176
0.113944401	0	0.222017761
0.040883329	0	0.199112615
0.028237213	0	0.074493773
0.048696787	0	0.172661572
0.139879265	0	0.075349689
0.110311583	0	0.078022048
0.10554492	0	0.175484643
0.059060754	0	0.115462363
0.062251087	0	0.099601999
0.094214728	0	0.09292655
0.050974814	0	0.159317742
0.054959693	0	0.18686552
0.050731651	0	0.13953008
0.102325471	0	0.117818647
0.048700698	0	0.065310988
0.203262758	0	0
0.176332389	0	0.019258137
0.095637643	0	0.01485918
0.19966511	0	0
0.108424401	0	0.051184008
0.101708481	0	0.100645479
0.141999174	0	0.111040015
0.050376831	0	0.093220047
0.099548078	0	0.209770023
0.040568285	0	0.059049266
0.16134801	0	0.071589148
0.058627453	0	0.10521827
0.092039852	0	0.034908387
0.052119573	0	0.097250909
0.101720113	0	0.068764713
0.105728936	0	0.149064287
0.113004446	0	0.165628824
0.066532011	0	0.114535541
0.169273527	0	0.068325617
0.170289909	0	0.107903619
0.110648387	0	0.175387993
0.059589514	0	0.023035944
0.158974451	0	0.141523524
0.047470893	0	0

0.111140861	0	0.03010421
0.08834427	0	0.135687179
0.070924275	0	0.13964226
0.063183612	0	0.179043819
0.060226354	0	0.087964407
0.12445231	0	0.0672011
0.04116469	0	0.1608785
0.113937537	0	0
0.024775883	0	0.105210907
0.106875817	0	0.027639017
0.210363057	0	0.087407814
0.090537509	0	0.047359144
0.061118648	0	0.080441406
0.021981503	0	0.094115597
0.087603871	0	0.096260302
0.068414994	0	0.038826494
0.047893555	0	0.156166933
0.067942798	0	0.066385638
0.139198539	0	0.121088722
0.052202417	0	0.115297879
0.174539255	0	0
0.047058669	0	0
0.246032748	0	0
0.117640266	0	0.146287803
0.110677301	0	0.132387298
0.062927806	0	0.142085148
0.136701014	0	0.131892054
0.112438567	0	0.128429293
0.056416459	0	0.105621578
0.127854171	0	0.095211425
0.015761811	0	0.01034064
0.060334719	0	0.04715088
0.129362768	0	0.170088386
0.116732912	0	0.019845247
0.172480989	0	0.053814808
0.050878046	0	0.02146026
0.130178523	0	0.137050797
0.093503137	0	0.196785394
0.022648574	0	0
0.147978036	0	0.099377267
0.033975525	0	0.055519974
0.141757816	0	0.096537778
0.023066487	0	0.149544169
0.144070996	0	0.124755713
0.110230168	0	0.203653762
0.120169947	0	0.107570659
0.1163198	0	0.126035349
0.111267168	0	0
0.030914485	0	0.089369555
0.143246552	0	0.041743791
0.093068552	0	0.084316442
0.050137641	0	0.203278429
0.057475993	0	0.046233767
0.055642659	0	0.094806541

0.051741376	0	0.089336158
0.04441252	0	0.092525826
0.080331445	0	0.188173891
0.249823944	0	0
0.195294747	0	0
0.065906084	0	0.017234493
0.071252146	0	0.073180604
0.113087991	0	0.05567471
0.087583586	0	0.140336124
0.269422912	0	0.02867286
0.03540922	0	0.121694545
0.070569827	0	0.187996872
0.012079837	0	0.083402882
0.224911756	0	0.057194737
0.059887553	0	0.144082329
0.161122628	0	0.023639671
0.052057983	0	0.178040425
0.268317793	0	0
0.049158444	0	0.126870637
0.15787448	0	0.092614247
0.033034422	0	0.126993746
0.180968821	0	0.101247729
0.172300865	0	0.166416477
0.08860384	0	0.250985382
0.096534109	0	0.137104934
0.067039804	0	0.21784885
0.049190056	0	0.200003919
0.073181445	0	0.124616194
0.083619609	0	0.077590079
0.147523001	0	0.119634192
0.047025211	0	0.021712362
0.153536206	0	0.092196886
0.081780252	0	0.094689025
0.081249505	0	0.118510218
0.048603897	0	0.168304385
0.11560287	0	0.058530979
0.165626326	0	0.105283127
0.173763592	0	0.106443083
0.051813261	0	0.173715984
0.056710874	0	0.18724196
0.049681492	0	0.107842087
0.042975716	0	0.002247733
0.133213561	0	0.103260139
0.106886979	0	0
0.0547325	0	0.099546515
0.127122048	0	0
0.006854397	0	0.155941399
0.212286382	0	0.081970897
0.088545869	0	0.142348365
0.028439423	0	0.164242462
0.04348128	0	0.179295316
0.164935099	0	0
0.058101456	0	0.253239205
0.036464684	0	0.107043371

0.047886031	0	0.061656984
0.075717008	0	0.058813479
0.157235528	0	0.055437314
0.107104486	0	0.093375638
0.168626387	0	0.090483838
0.052857842	0	0
0.176424933	0	0.111258139
0.095985214	0	0.046519897
0.094369033	0	0.23551036
0.089091411	0	0.175538825
0.052142532	0	0.210632787
0.030895263	0	0.105107344
0.09510058	0	0.068521285
0.058021687	0	0.118319139
0.067194567	0	0.080134478
0.059996273	0	0.148737676
0.037414637	0	0.19967471
0.052170452	0	0.16235529
0.035671185	0	0.198103754
0.046961888	0	0.121695412
0.103606064	0	0.07657444
0.151977037	0	0.03745
0.112387613	0	0.114986139
0.058764747	0	0.153241727
0.027881131	0	0.072167636
0.036366375	0	0.152416514
0.051509314	0	0.118858674
0.132583225	0	0.107143498
0.125910449	0	0.078477538
0.287610435	0	0
0.041351918	0	0.140802058
0.075248946	0	0.207135574
0.066098326	0	0.094571939
0.162000765	0	0
0.167059775	0	0.005735842
0.109202222	0	0.211994635
0.051349345	0	0.060906399
0.105744035	0	0
0.107362284	0	0.008615199
0.076469168	0	0.129676384
0.04754408	0	0.2507669
0.084199155	0	0.10432778
0.062271435	0	0.065005664
0.149576346	0	0.10697593
0.081305376	0	0.135189358
0.085800585	0	0.154996613
0.090820262	0	0.059885909
0.111427279	0	0.132987583
0.069108523	0	0.179157494
0.116844678	0	0.039248228
0.286399813	0	0
0.021751267	0	0.079419008
0.075803945	0	0.166382841
0.024323639	0	0.178473401

0.196603187	0	0.030524188
0.165944096	0	0.158597926
0.080157355	0	0.020858495
0.147150957	0	0.153213619
0.235475589	0	0.012147462
0.107939697	0	0.201584223
0.053086876	0	0.060407796
0.044751688	0	0.056828723
0.126426428	0	0.117476282
0.067487099	0	0.182900829
0.060629227	0	0.22687892
0.111767156	0	0.185351378
0.114582746	0	0.1216692
0.042218087	0	0.122525829
0.128089852	0	0
0.070509552	0	0.134159255
0.037530066	0	0.338772962
0.062833845	0	0.16962741
0.221554313	0	0
0.077298601	0	0.185205495
0.136989565	0	0.13395438
0.03935629	0	0.156864344
0.054478479	0	0.064319375
0.048393767	0	0.050664704
0.037285853	0	0.224847586
0.114637216	0	0.189383978
0.102739734	0	0.094720836
0.074952681	0	0.136460648
0.068991732	0	0.11746452
0.098945836	0	0.26366834
0.066290475	0	0.153676039
0.086925983	0	0.193379522
0.129388473	0	0.245578449
0.024768811	0	0.128395158
0.180352652	0	0.048558155
0.11811665	0	0.001845686
0.118390532	0	0.143805044
0.108573575	0	0.112254405
0.107910999	0	0.184384518
0.092806473	0	0.112168677
0.117708271	0	0.147875787
0.028559669	0	0.02978599
0.167503487	0	0.141580448
0.090563143	0	0.081126145
0.135967931	0	0
0.077264103	0	0.256563286
0.035920105	0	0.050737112
0.024780309	0	0.183063281
0.134996248	0	0.05190656
0.103794616	0	0.095917695
0.087567135	0	0.209309035
0.08818938	0	0.069235179
0.11931523	0	0.196544843
0.058211706	0	0.185711743

0.144070142	0	0.159009608
0.125523061	0	0.122556003
0.0680585	0	0.07373163
0.158599452	0	0.021274946
0.09205135	0	0.146535956
0.12020909	0	0.161099801
0.091293391	0	0.082595179
0.152213352	0	0
0.062323818	0	0.146583035
0.115542411	0	0.193072478
0.068796576	0	0.172606079
0.078415202	0	0.063912788
0.203173579	0	0.084827707
0.040695151	0	0.156863223
0.12907765	0	0.182669032
0.111412632	0	0.091712408
0.109386663	0	0.119721993
0.129825082	0	0.153405289
0.049960409	0	0.15834951
0.113645602	0	0.074450041
0.073424103	0	0.227083199
0.104340523	0	0.082876917
0.033018497	0	0.092714909
0.044238485	0	0.228115694
0.040589951	0	0.078810279
0.062649358	0	0.019378291
0.102375796	0	0.205606167
0.130099321	0	0.133694748
0.130850591	0	0.085984225
0.010181129	0	0.127656256
0.045247415	0	0.144598453
0.086377357	0	0.07469811
0.183361691	0	0.043472054
0.087289283	0	0.176487428
0.045929781	0	0.153484652
0.121980445	0	0.116603227
0.027053953	0	0.164480553
0.10559295	0	0.243492576
0.216340519	0	0
0.196176093	0	0.079711046
0.130518403	0	0.203917507
0.202163075	0	0.08502494
0.081772767	0	0.110719948
0.163873413	0	0.07116911
0.114859969	0	0.163406773
0.057777605	0	0.163969758
0.135446443	0	0.0874187
0.123312104	0	0.074107822
0.048724417	0	0.206540062
0.098810248	0	0.131435807
0.137572486	0	0.154133691
0.040056557	0	0.100500715
0.052151948	0	0.158258911
0.043507054	0	0.085133528

0.0525763	0	0.199951046
0.047293489	0	0.153142611
0.19468373	0	0
0.058966524	0	0.102385717
0.078104754	0	0.17395238
0.1582918	0	0.101156867
0.108731624	0	0.087686
0.096829416	0	0.196152355
0.100726435	0	0.032848797
0.079568926	0	0.251637392
0.089714219	0	0.205456132
0.023163227	0	0.183354917
0.21466222	0	0.112943702
0.098854082	0	0.172070461
0.089118263	0	0.100544039
0.108264646	0	0.260354344
0.06498297	0	0.091800037
0.078741114	0	0.132079474
0.146893086	0	0.070155999
0.113470498	0	0.010278357
0.135870254	0	0.091162266
0.15243823	0	0.107076559
0.076764216	0	0.043478923
0.143371197	0	0.101946677
0.082254336	0	0.274444881
0.117536289	0	0.199884712
0.133509564	0	0.193753694
0.073001306	0	0.187222568
0.124661785	0	0.107791713
0.137591959	0	0.203309848
0.105547346	0	0.246986535
0.090406034	0	0.159460769
0.052853133	0	0.224243579
0.047279299	0	0.160862847
0.074994632	0	0.12439205
0.040280148	0	0.127501093
0.15311743	0	0.125491117
0.103140994	0	0.137358773
0.127505948	0	0.092054468
0.126780131	0	0.22860991
0.085401477	0	0.118994905
0.033814402	0	0.251570036
0.024673015	0	0.090329585
0.119038067	0	0.119836189
0.068746812	0	0.084616388
0.041661583	0	0.13451695
0.191787026	0	0.051710766
0.157052446	0	0.094781451
0.036778495	0	0.110683284
0.03302126	0	0.123785447
0.056105709	0	0.117971663
0.080318439	0	0.156172227
0.04602497	0	0.392844293
0.098592235	0	0.127573252

0.049712998	0	0.213383301
0.143585002	0	0.087819739
0.049570422	0	0.174407947
0.077134375	0	0.099752739
0.08740074	0	0.11555968
0.035461741	0	0.101173901
0.099750644	0	0.015773121
0.101497248	0	0.1442894
0.038776627	0	0.062769339
0.082545662	0	0.062328756
0.055048077	0	0.126356634
0.08006124	0	0.063014513
0.074944243	0	0.08721979
0.065063259	0	0.163833985
0.174521363	0	0.144109099
0.030265953	0	0.094712739
0.038361154	0	0.126442951
0.130827689	0	0.289496224
0.037985035	0	0.14729184
0.167428824	0	0.171100389
0.165390525	0	0.105490768
0.054779922	0	0.038799039
0.067171937	0	0.143851535
0.167443322	0	0.043710299
0.103279362	0	0.080321281
0.05283189	0	0.155221466
0.100011939	0	0.173147215
0.090600069	0	0.039044482
0.107987613	0	0.057752281
0.098034798	0	0.085157676
0.19005041	0	0.031455317
0.108321324	0	0.028154514
0.022644177	0	0.080851484
0.072245572	0	0.190671149
0.135196873	0	0.174468977
0.250075496	0	0.071893708
0.04960195	0	0.174849416
0.061220895	0	0.026146364
0.157893262	0	0.153712458
0.131993419	0	0.119414394
0.077522869	0	0.226984602
0.026533059	0	0.083884969
0.062512238	0	0.203190646
0.031537446	0	0.037654551
0.134397848	0	0.165925513
0.174736314	0	0.131094955
0.057021986	0	0.045099071
0.081730478	0	0.08304878
0.055661864	0	0.16449732
0.300274806	0	0
0.048483827	0	0.061138299
0.223887684	0	0.131129064
0.085554072	0	0.06971101
0.041620935	0	0.095793583

0.075159269	0	0.229228969
0.094172427	0	0.14050068
0.23154764	0	0.054526341
0.092266102	0	0.196183042
0.046419074	0	0.042581615
0.040584984	0	0.138785026
0.090590654	0	0.08954799
0.070592956	0	0.012733819
0.083581113	0	0.03431812
0.091150059	0	0.132552396
0.067155835	0	0.057214725
0.110561831	0	0.205329806
0.041686698	0	0.065386816
0.152977084	0	0.107178317
0.088219518	0	0.110336244
0.127079049	0	0.133015003
0.022006147	0	0.102983821
0.327300576	0	0
0.10625985	0	0.154962311
0.085055356	0	0.077247857
0.237075329	0	0
0.0703855	0	0.152575417
0.056823252	0	0.15256976
0.089501291	0	0.090046472
0.096644058	0	0.199945344
0.100067077	0	0.080677688
0.07050023	0	0.141561771
0.056398857	0	0.236004837
0.089526805	0	0.188715914
0.073890519	0	0.220848712
0.106037826	0	0.070152814
0.037843133	0	0.050854956
0.108554824	0	0.080653809
0.083081583	0	0.205266383
0.085947149	0	0.170411466
0.098985768	0	0.067805646
0.129596037	0	0.107197703
0.063154307	0	0.241870908
0.019886284	0	0.162862944
0.032964781	0	0.125957601
0.160317279	0	0.258010294
0.057633747	0	0.172362719
0.050144688	0	0.186603176
0.051782912	0	0.168099646
0.030506991	0	0.09737348
0.081405257	0	0.171050708
0.025967905	0	0.076775745
0.17073073	0	0.091028812
0.090211095	0	0.112575978
0.058628733	0	0.17131789
0.077009901	0	0.110402367
0.148957115	0	0.107889034
0.07614406	0	0.24800988
0.048941248	0	0

0.076528196	0	0.119545232
0.14240695	0	0.107596715
0.078870521	0	0.109661267
0.157337802	0	0.140637609
0.100525826	0	0.118436761
0.028348226	0	0.175976529
0.080889256	0	0.109475802
0.131225025	0	0.108656384
0.066762007	0	0.101452583
0.057390106	0	0.181989059
0.090320485	0	0.117349459
0.17098605	0	0
0.155303915	0	0.121061676
0.008431413	0	0.111410209
0.078035975	0	0.180878266
0.094885384	0	0.121977408
0.140846192	0	0.057170777
0.119391198	0	0.020397067
0.198133554	0	0.050898371
0.069702164	0	0.178402834
0.13971844	0	0.158569571
0.04556041	0	0.142351836
0.240335662	0	0
0.059658446	0	0.037100554
0.083649371	0	0.103277971
0.165211891	0	0.100931699
0.030955439	0	0.099147088
0.096222528	0	0.097651129
0.074717809	0	0.052948613
0.337148168	0	0
0.104833852	0	0.133768596
0.026431293	0	0.147698717
0.087598226	0	0.107980103
0.132855816	0	0.083224474
0.080023603	0	0.175153746
0.064181519	0	0.139079987
0.093468783	0	0.149158124
0.089488314	0	0.234117542
0.067903804	0	0.165460324
0.139594091	0	0.189216241
0.07631648	0	0.060568514
0.089984531	0	0.122493575
0.080595421	0	0.11133955
0.113523077	0	0.167119839
0.118998236	0	0.007746316
0.082070346	0	0.089823541
0.212210105	0	0.122354678
0.081637662	0	0.176819061
0.072305809	0	0.158291299
0.035460481	0	0.063964512
0.106907859	0	0.238698409
0.04529497	0	0.065248711
0.039353812	0	0.092226145
0.160418107	0	0

0.094157085	0.006638614	0
0.098623747	0	0.158574477
0.142704944	0	0.096985098
0.05700234	0	0.024869864
0.148729438	0	0.086820308
0.106830811	0	0.173430648
0.154995988	0	0.111495117
0.043850167	0	0.053907161
0.079838177	0	0.166104416
0.14793295	0	0
0	0	0.244834241
0	0	0.307316253
0.123808377	0	0.051659231
0.006609157	0	0.175158621
0.005576271	0	0.357131944
0.080883202	0.012981357	0.025803609
0.055215664	0	0.110910755
0.027514181	0	0.098535773
0.05234359	0	0.205081718
0.031761913	0.016227397	0.140694119
0	0	0.131749703
0.090022805	0	0.279484461
0.005951757	0.001803622	0.249699847
0.090227702	0	0.053850427
0	0.051626911	0.228270172
0	0	0.295998196
0.031112331	0	0.257397134
0.065714644	0	0.233631616
0	0	0.365987701
0.130444917	0	0.177196599
0	0.009701891	0.467600631
0	0	0.287785331
0	0	0.425391834
0.130784023	0	0.22286984
0.151160828	0	0
0.063536785	0	0.117108497
0.108574713	0	0
0.458553892	0	0
0.021610124	0	0.184941432
0.158598523	0	0.138556641
0.070130466	0	0.128458668
0.111683448	0	0.042053052
0.220134724	0	0.003296489
0.189533725	0	0
0.119313776	0	0.070191979
0.185734803	0	0
0.133743512	0	0.144644061
0.029148948	0	0.18771414
0.164886481	0	0
0.102438294	0	0.010633272
0.082230064	0	0.087284986
0.117809551	0	0
0.072793226	0	0.059752432
0.127126088	0	0

0.040348314	0	0.150040827
0.148702277	0	0
0.022715841	0	0.07051103
0.067710212	0	0.198843032
0.100856793	0	0.019251589
0.062079989	0	0.006016343
0.118532959	0	0.059109223
0.060081873	0	0.153215901

T.cells.CD4.memory.activated	T.cells.follicular.helper	T.cells.regulatory..Tregs.
0.031438076	0.107587171	0.011263517
0	0.06463333	0.018790416
0.01919159	0.022292959	0.042743799
0	0.114428555	0
0.004025086	0.07081507	0.037547214
0	0.089088408	0.053512175
0	0.128966167	0.024428269
0	0.049416896	0.066361121
0.002529179	0.004716308	0.008005593
0.012606776	0.059063202	0.011292572
0	0.045223599	0.050658933
0	0.179654495	0.013873865
0	0.039808607	0.024226944
0.012700218	0.080675198	0
0	0.028556427	0.013881703
0	0.098267278	0.0085851
0	0.071001777	0
0	0.09622878	0.012852085
0	0.074236643	0.042519652
0.000996191	0.072493594	0.030522122
0	0.057612995	0.060811478
0	0.036539233	0
0	0.107069338	0
0	0.069597869	0.03695527
0	0.029951366	0.011063203
0	0.060985203	0.062912184
0	0.089584995	0.087912916
0	0.087316385	0
0	0.101053229	0.020283603
0	0.078576833	0.045890464
0.02725991	0.172096231	0
0	0.0456358	0.063170762
0	0.202395526	0
0	0.074273366	0.028008464
0.031679681	0.057260899	0.050894986
0	0.066962201	0.012137463
0	0.027536474	0.010732466
0	0.109335335	0
0	0.038241512	0
0	0.128678614	0.026065804
0	0.05150825	0.001871334
0	0.049989203	0
0	0.029468572	0.018453843
0	0.083267643	0.011268991
0	0.054443146	0.070235614
0	0.059284885	0.02310928
0	0.119777673	0.049747387
0	0.024772427	0.080450136
0.028067625	0.035371387	0.021171989
0.023530276	0.050663526	0.003642853
0	0.095724294	0.035253756
0	0.058564027	0.008940189
0	0.07629829	0

0	0.043280667	0.028075446
0	0.02522493	0.038613963
0	0	0.038395854
0	0.035608118	0.025786035
0	0.071577621	0.025656467
0.008308205	0.044409209	0
0	0.061176842	0.062968558
0	0.138830376	0.034147827
0	0.022156659	0.024269536
0.027092901	0.085724939	0
0.013205886	0.087397859	0.078342496
0	0.105622438	0.030342202
0	0.076596024	0.055144103
0	0.009098445	0.021955775
0	0.084504447	0.01151512
0	0.070868918	0.045492701
0	0.03539286	0.00706678
0	0.055912848	0.003253339
0.01175588	0.079033434	0.001713721
0	0.036116369	0.051281468
0	0.110077286	0.073132633
0	0.179987003	0.017557107
0.054305557	0.068939619	0.038791903
0.012327182	0.034208829	0.057205962
0	0.079321329	0.075641894
0	0.055937075	0.0401744
0.024567229	0.054230677	0.063303496
0	0.102476432	0
0	0	0.021954338
0	0.081474158	0.053040075
0	0.026614494	0.052295114
0.046395331	0	0.040549232
0.01405262	0.091048269	0.045893367
0	0.056830178	0.028897484
0.02821388	0.032830154	0.02011741
0	0.032309555	0.030790504
0.003390036	0.092237575	0.041688284
0	0.036700973	0.010872113
0	0.131940497	0.011939562
0	0.037083272	0
0	0.034600005	0.01895429
0	0.103670656	0.021138025
0	0.077002493	0.002678381
0	0.068198019	0.029413023
0	0.095684052	0.014246188
0	0.065977663	0.052707018
0	0.040199335	0.01464679
0	0.040703387	0.015821042
0	0.040642431	0.024186458
0	0.056365251	0.078847505
0	0.042319609	0.020689104
0	0.071203575	0.027246989
0	0.025803013	0.00300231
0	0.062100684	0.005189704

0	0.07686796	0.022715985
0	0.055130456	0
0	0.026412018	0.01011692
0.082585385	0.083926671	0
0.037655733	0.114903117	0.016052224
0	0.087015566	0.007103698
0	0.083186701	0.009003701
0	0.069986976	0.055713588
0	0.0098794	0.001071974
0.135116892	0.096651468	0
0	0.030258529	0.057936725
0	0.056730691	0.006268877
0	0.052967304	0.031583373
0.101873871	0.032165437	0.016032478
0	0.036257292	0.044762347
0	0.123969656	0.020842775
0	0.061718985	0.019314865
0	0.06619185	0
0.005262246	0	0.007534708
0.004159927	0.054600862	0.030650612
0	0	0
0.035591081	0.079014187	0.026211214
0.038670461	0.048448657	0.018456639
0	0	0
0	0.080172448	0.028049297
0	0.01097569	0
0	0.02285291	0.025809783
0	0.067027462	0.03880088
0	0.071654113	0.020608861
0.002970394	0.038450588	0.072215443
0	0.020033146	0.030453288
0.022297845	0.046609147	0
0.003048117	0.018848182	0
0	0.075213831	0.050362531
0	0.022108103	0
0	0.027876933	0
0	0.100979902	0.041142113
0	0.095692277	0.070603587
0	0.024818164	0
0	0.027296531	0.011771011
0	0.025968154	0.014084889
0	0.078576482	0
0.04616536	0.042778979	0
0	0.173554054	0.002052852
0	0.015117909	0.035306242
0	0.106884158	0.031803954
0	0.042310616	0.004403584
0	0.110669521	0.024540592
0	0.045775477	0.012342897
0	0.047934059	0.046957054
0	0.042704589	0
0.045492099	0.158367312	0.007641083
0.011711411	0	0.012278026
0	0.030216104	0.00530244

0.003541285	0.003638554	0.020772157
0	0.114719209	0
0.001595818	0.026660773	0.018821159
0	0.074697703	0
0.009358502	0.056618338	0.043893606
0.033452911	0.068611267	0
0.022029871	0.017953061	0.023726101
0.007734047	0.051812144	0
0	0.041185393	0.046166969
0	0.01095564	0
0	0.004177833	0
0	0.016350422	0
0.051939402	0.060731776	0.019810988
0.021034238	0.028701712	0.014830886
0	0.055867618	0.011322396
0	0.036532316	0.001542166
0	0.006773435	0.003218809
0	0.014315407	0.050522155
0.001456759	0	0.017262328
0	0.06803084	0.022997829
0	0.02493825	0.067645629
0.015410936	0.071966999	0.051814708
0	0.054405049	0.063220802
0	0.012095442	0.001266442
0	0.0249821	0.01312365
0	0.037086053	0.04567909
0	0.004882772	0.044068964
0.002131211	0.049720454	0.078479317
0.072258931	0.08355792	0.022081244
0.077198371	0.080437502	0.019189451
0	0.068230354	0.043318819
0	0.079260991	0.001100201
0.000205587	0.036042768	0.020303442
0	0.156290845	0.010255311
0.005533812	0.135237355	0.012705368
0	0.067224933	0.02316465
0	0.074729594	0.011439542
0	0.11166048	0.017592234
0.001551898	0.04425069	0.029598462
0.069836668	0.048445828	0.025600522
0	0.081172977	0.012083832
0	0.044876177	0
0	0.0814074	0.036121123
0	0.052448954	0.088218895
0.011413167	0.060663	0.077076068
0	0.02395587	0.042155343
0	0.066825111	0.030581377
0	0.030593499	0.013631736
0	0.105251023	0.020147338
0	0.117914104	0
0.08953207	0.08589465	0.011035857
0.011224821	0.019515763	0.020382463
0	0.038869402	0.009616629
0	0.011270668	0.019170238

0.035328354	0.046477172	0.055245258
0.003442376	0.089789238	0.023254289
0	0.042402475	0
0.000876568	0.019909898	0.060424379
0.024196846	0.056974758	0.016890346
0.001682084	0.013931032	0.013022734
0.032625733	0.020020223	0
0	0.065945137	0.045319842
0	0.078038526	0.046595106
0	0.045440921	0
0	0.094680397	0
0	0.018105472	0
0.01456991	0.02571991	0.015533983
0	0.00658623	0
0	0.048390265	0.012712031
0.047819895	0.075428487	0
0.063439145	0	0
0	0.022575225	0
0.013026544	0.094832587	0.000854528
0.003227392	0.010427567	0
0	0.024385468	0.015258281
0	0	0.01264361
0	0.059009766	0
0	0.03112645	0
0	0	0
0	0.012453535	0
0.012692535	0.002091956	0
0.028074953	0.003510177	0.003532704
0	0.067334843	0.007524219
0	0.010418777	0
0	0.054541075	0.015625348
0	0.031640146	0.011843595
0	0.056726119	0.006334129
0	0.121144606	0
0.162089066	0.120907885	0
0.020101493	0.100879113	0.015166732
0	0.100218273	0.015661954
0.008692881	0.067811392	0.005938806
0	0.066336511	0
0	0.086869551	0
0	0.080198537	0.028153277
0	0.030388019	0.011276846
0.000982078	0.065610162	0.048677775
0	0.035068621	0.053974874
0	0.162443939	0.042198218
0.007756692	0.030693049	0.012303142
0	0.04574336	0.02451872
0	0.053085991	0
0.014937477	0.029048679	0.081917994
0	0.172934697	0.034953693
0	0.037815365	0.009320162
0.002582044	0.052620157	0.01287508
0	0.035330775	0.001142467
0	0.037829772	0.056946614

0	0.070677731	0.01409102
0	0.040173826	0.058826635
0	0.042788946	0.045521616
0	0.08789945	0.049998412
0.110999954	0	0.003646568
0	0.028895852	0.001758579
0	0.064159457	0
0.012922582	0.09044312	0.018382059
0.008075745	0.024243233	0.010792468
0	0.046740956	0.015698771
0	0.035047698	0.043341324
0.021128673	0.119406546	0.033371711
0.045665989	0.080564398	0.005158084
0	0.035907049	0.01221324
0	0.023077068	0.003794512
0	0.035440165	0.019045498
0.022214457	0.013076752	0.013375879
0	0.044100595	0.023297675
0	0.053028416	0.026453745
0	0.068195856	0.092346027
0	0.038072452	0
0	0.084805564	0.009741206
0	0.057037365	0.009540621
0	0.036959397	0.043797652
0	0.069093658	0
0	0.027845812	0.002360108
0	0.045737311	0
0	0.057303744	0.007721338
0	0.092088589	0.013655645
0	0.017452356	0.007554501
0	0.001241886	0
0.006345171	0.022994423	0.006203987
0.085700023	0.116225603	0
0.006149829	0.023710697	0.02105832
0	0.085515842	0.022298296
0.005867679	0.092118706	0.012569041
0.008789259	0.020378283	0.009636235
0	0.048014306	0
0.013321268	0.133165147	0.000337854
0	0.064490986	0.080090804
0.00780329	0.034452528	0.022065096
0.026081277	0.064840142	0
0	0.045501218	0.056683304
0.022790829	0.056970763	0.017956988
0.045131084	0.070217165	0.000538354
0	0.013150891	0
0	0.061581072	0.026147711
0.002481933	0.081016283	0.013769388
0	0.08122953	0.002683849
0	0.044964497	0.043446973
0.002901259	0.023665869	0.013985392
0	0.089960031	0.039211052
0	0.028076708	0.059553069
0	0	0

0	0.010092386	0
0	0.000994175	0
0	0.10924256	0
0	0.045051079	0.00551728
0	0.013077883	0
0	0	0
0	0.020124917	0
0	0	0
0	0.061187867	0.0209728
0	0.019529379	0
0	0.013512918	0
0	0.016711814	0.014129779
0	0.047031414	0.01028161
0	0.030944136	0
0.016946136	0.014575804	0.033850606
0	0	0.017810623
0.009934446	0.033317079	0.016401966
0	0.003373554	0.016527116
0.015747765	0.045358896	0
0	0.06437311	0.002128601
0	0.037905866	0
0.008981054	0.035590576	0.052615763
0.003654456	0.048401778	0.003425547
0.002282513	0.061189605	0.017275076
0	0.07200744	0.074601756
0	0.053484452	0.046521364
0.015756194	0.030347997	0
0.012287505	0.025100925	0
0	0.065956132	0
0	0.004983156	0.057038278
0	0.029070888	0
0	0.024051816	0
0	0.049010363	0.004412826
0	0.009102128	0
0	0	0
0	0.006311765	0
0.010264135	0	0
0	0.022586591	0
0	0.080708475	0.015718608
0	0.051046636	0.052384851
0	0.011978859	0.039127903
0	0.034524323	0.057761234
0	0.039919278	0.02950385
0.044435203	0.062419501	0.047235744
0	0.014855264	0.045143218
0.003734419	0.03453535	0.038394273
0.037498386	0.134551088	0.01577776
0.020504725	0.028203081	0.013641388
0	0.014457264	0.014813959
0	0.01318793	0.020945205
0	0.024621445	0.026823253
0.032545206	0.028834134	0.047616554
0	0.015298157	0.014743543
0	0.052656555	0.029030892

0	0	0
0	0	0.013779441
0	0	0
0.007191036	0.009774235	0
0.052405836	0.055070684	0.014134764
0	0.034145704	0.035759256
0	0.235189482	0
0	0.053823598	0.006287833
0	0.05607573	0
0.008978987	0.073495967	0.009747801
0	0.021450073	0.024925168
0	0.018370719	0.046137015
0.050016268	0.004852696	0
0	0.019817447	0
0.011461891	0.067377232	0.028156263
0	0.019345007	0.034306239
0	0.044084114	0
0	0.037049069	0
0	0.074186568	0.008866077
0	0.017327947	0.056762024
0.027881617	0.039136785	0
0	0.029358861	0.011404718
0.004703133	0.043622985	0.009955456
0.027125183	0.060281795	0.047028857
0	0.013133974	0.006854329
0	0.027732827	0.029119996
0	0.073344577	0.054314635
0	0.035265848	0.020534418
0.024498677	0.012796591	0.015227258
0.01916881	0.021848193	0.013843359
0	0.129058207	0.104473537
0.0020758	0.078546828	0
0	0.007910506	0
0	0.00727882	0
0	0.021708606	0.011755007
0.020437951	0.045618229	0
0	0	0
0	0.019730208	0.009963674
0	0.003318902	0
0	0.070027867	0.130410151
0.001188307	0.065549728	0
0	0.020826443	0.032040111
0	0.063333719	0
0	0.09820352	0.027678635
0.002938873	0.045305494	0.055233759
0	0.04835653	0.028975999
0	0.034594345	0.046291113
0	0.084307576	0.02108841
0.000290879	0.026871573	0.008112736
0.039679379	0.108801124	0.063563552
0	0.009438303	0
0.058504767	0.048006169	0.024712815
0.012656461	0.073608894	0
0.007023357	0.03012006	0.033865772

0.002881677	0.012494262	0
0	0.044686398	0
0.038098755	0.060450431	0.047242893
0	0.023492866	0
0	0.061609178	0.023620788
0	0.022117463	0.019002033
0	0.098914743	0
0	0.084362418	0.014937671
0	0.051029323	0.009883236
0.086507882	0	0
0.006715254	0.06777816	0.017553944
0	0.046413422	0.026410796
0	0.044924891	0.00284634
0	0.085197102	0.025167138
0	0.099543786	0.032222834
0.019987589	0.024988642	0.038332958
0	0	0
0.058079697	0.116916308	0.026666817
0.059622162	0.055258783	0.01639995
0	0.041562948	0.000441267
0.039144637	0.133476396	0
0	0.020522714	0.035574367
0	0.026187237	0.005544302
0	0.05496054	0.069584826
0	0.062804166	0
0	0.040661909	0.03819863
0	0	0
0	0	0
0	0.004828833	0
0	0	0
0	0.043099765	0.004686165
0	0.013612307	0
0	0.086107661	0
0	0.02459259	0
0	0	0
0.06501995	0.00743477	0.00925954
0	0.041388213	0.014376756
0	0.000931811	0
0	0	0
0	0	0
0	0.002859044	0
0	0	0
0	0	0
0	0.003472292	0
0	0.02713132	0
0	0.006563091	0
0	0.01359388	0.023485768
0	0.061481912	0.022338542
0	0.07289322	0.018381798
0	0.049899466	0.032461828
0	0	0.025326932
0.005106662	0.039857344	0.022029727
0	0.020094571	0.061125296
0	0.034579046	0

0	0.030306134	0.014754136
0.010993461	0.06008444	0.047849791
0	0.003944523	0.059589621
0	0.056424623	0.00197791
0	0.060452896	0.014984443
0	0	0.008819094
0	0.048952699	0.022597844
0	0.063326716	0.039184188
0	0.071486311	0.015282414
0	0.025176455	0.014230841
0.007203824	0.050805437	0.016698583
0	0.14192561	0.004440072
0.030917533	0.066157983	0.024236016
0.016633157	0.009821738	0.012641647
0	0.023757816	0
0	0.031867276	0.128355275
0.025248977	0.038408293	0.024762202
0.015981577	0.092060174	0.048395011
0	0.045086307	0.046322036
0	0.037431759	0.036242752
0.012292975	0	0.028655079
0	0.062373704	0.006689693
0.105364703	0.059165011	0
0	0.038999186	0
0.024404408	0.021809404	0
0.042492715	0.05700732	0
0	0.031013708	0.031005553
0	0.045858038	0
0.028246178	0.086757603	0
0.061705458	0.089587821	0.026374239
0	0.071813834	0.036615774
0	0.056227695	0.029482154
0.013780786	0.065680054	0.024548191
0.007701764	0.070906309	0.026897444
6.20E-05	0.094858229	0.021046906
0	0.056219318	0.024175895
0	0	0
0.017728211	0.029167547	0
0	0.045252079	0
0.012565966	0.042160654	0.044092036
0.013865277	0.056374382	0.009722824
0.007549014	0	0.018340163
0	0	0
0.006623338	0.037196153	0.027501513
0	0.110682037	0.081435432
0	0.084925721	0.031530375
0.007905105	0.077404198	0.006488178
0	0.079669051	0
0	0.034603029	0.008337935
0	0.040792963	0.029350735
0.018272424	0.092749749	0
0	0.093003937	0
0	0.020346986	0.029579935
0.019276269	0.126354609	0

0.029178689	0.048960142	0
0	0.063858813	0.034184752
0	0.113428496	0.044574985
0	0.063837502	0.030197032
0	0.123315227	0.01421943
0	0.136594955	0
0.039611877	0.090855528	0.015204521
0	0.089178482	0.019091875
0	0.016468503	0.019029036
0	0.095772396	0.040205347
0.030582034	0.035438383	0
0.084840239	0.03196189	0
0.091307578	0	0
0.015265965	0.036152126	0
0.021970366	0.046735061	0
0.096881142	0	0
0	0.021667328	0.010028142
0.002576614	0	0.010852767
0.002506983	0	0
0.011546174	0.030144543	0
0.000269557	0.006210418	0
0	0.03201382	0
0.009438533	0	0.012961533
0.106985659	0.036901852	0
0.047162021	0.001803239	0
0	0	0
0	0.051497845	0
0.069879452	0.134390268	0
0.000555243	0.039231965	0
0.076887175	0	0
0.011502931	0.003366507	0
0.055002924	0	0
0.015694331	0.034037147	0
0.039808076	0.01873225	0
0	0.173252586	0.031450952
0	0.071656775	0.066759628
0	0.114730834	0.026259464
0.032126175	0.141521587	0.022297888
0	0.02588553	0.046674414
0	0.072226992	0.019114455
0	0.042887535	0.060450503
0	0.099471803	0.026420104
0.029358978	0.07353319	0.042023565
0.005693329	0.097725685	0.007339138
0.030501782	0.06966397	0.020086513
0	0.040881497	0.011844794
0	0.06927203	0.058029169
0	0.057170937	0.025751675
0	0.115838638	0.032193159
0.009109784	0.09830719	0.047887611
0	0.129507524	0.068164374
0.018592387	0.050118828	0.042981096
0.019557496	0.092009752	0.028582433
0	0.195002187	0.035513122

0	0.036993911	0.046337637
0.014282987	0.110463681	0.020958556
0	0.100227938	0.01264459
0.017284951	0	0.03407495
0	0.074084201	0.049978874
0	0.037917095	0.039630597
0.009637224	0.026005003	0.009745578
0	0.008041044	0

T.cells.gamma.delta	NK.cells.resting	NK.cells.activated	Monocytes	Macrophages.M0
0	0.021876178	0.02039608	0.012785826	0.007724846
0	0	0.040235161	0.049461935	0.076907611
0	0.025133579	0	0.001750919	0.097792633
0	0.029339679	0.049245662	0.036828759	0.035505144
0	0	0.008381032	0.025301574	0.084858007
0	0	0.016366478	0	0.318922426
0	0	0.045809673	0.01838688	0.067504831
0	0.038881176	0	0.031164954	0.010589478
0	0.002794481	0	0	0.167668354
0	0.003799512	0.023502842	0.006472887	0.025295156
0	0.023821117	0	0.099101197	0.231796296
0	0	0.031222305	0.013657177	0.018845366
0	0	0.019218854	0.001927651	0.348757476
0	0	0.076707208	0.058776393	0.008727715
0	0.045067695	0.017849673	0.04250248	0.051250681
0	0.017154809	0.005881376	0.035591966	0.002559078
0	0.008647329	0.010719859	0	0.064263182
0	0.001742437	0.023105921	0	0.125004824
0	0.009192401	0.031053826	0.043444344	0.027342825
0	0.025830839	0	0.012549704	0
0	0	0.067999258	0.069879278	0
0	0.005677725	0.029359446	0.079182508	0
0	0	0.054954701	0.061774781	0
0	0.018466379	0.032001732	0.05679262	0.105277669
0	0.031071095	0.015662421	0.024050624	0.049943383
0	0	0.055496672	0.074046715	0
0	0	0.040543027	0.027089302	0.044559265
0	0.017727316	0.013220138	0.004257435	0.073630558
0	0.00491381	0.016219899	0.017863619	0.046547346
0	0.023167781	0	0.066473925	0.01161566
0	0.049743153	0.021217132	0.033536999	0.025015589
0	0.02338491	0	0	0.160010245
0	0	0.05849702	0.002345844	0
0	0.010818706	0.000324066	0	0.249993021
0	0	0.02767077	0.003168726	0.14836032
0	0	0.017079844	0.020241759	0.02600254
0	0.029267746	0.003690431	0.013834682	0.115609325
0	0	0.064997207	0.021227907	0.021706312
0	0.009670109	0.007367131	0.013955154	0.128399756
0	0	0.070715942	0.019036503	0.011903229
0	0	0.023694909	0	0.148190524
0	0.001337202	0.017565923	0.010559956	0.095939406
0	0.013625784	0.044196669	0.283674087	0
0	0	0.0082911	0.022164766	0
0	0	0.000920263	0.032072524	0
0	0	0.024023418	0.002587817	0.083778929
0	0	0.055213991	0.018049665	0.036573377
0	0	0.065718988	0	0.122054513
0	0	0	0.02009858	0
0	0.020955117	0.010184918	0.010218694	0.096845685
0	0	0.053391275	0.016224407	0.006172536
0	0	0	0.034713376	0
0	0.028765653	0.021313635	0.006491502	0.061500048

0	0	0.039503206	0	0.018125179
0	0.000832313	0.017399496	0.10649673	0.026201668
0	0	0.051373991	0.146204342	0
0	0	0.014759781	0.057782187	0
0	0	0.040407689	0.030153636	0
0	0	0.032822827	0.077439711	0
0	0.003873366	0.014751484	0.021190875	0.087660868
0	0.006678686	0.033367234	0.059620345	0.059321574
0	0.030628155	0.015329604	0.249400942	0
0	0.005762119	0.021547598	0.00849936	0.066295814
0	0	0.064846794	0.02630292	0.051122776
0	0	0.023102519	0.035247629	0.146312993
0	0	0.03642143	0.079337572	0
0	0.014103569	0.00407552	0.032160622	0
0	0	0.045313677	0.029434284	0.03506774
0	0	0.032449729	0.004500357	0.317948148
0	0	0.04567205	0.074905447	0
0	0.021112596	0.001357368	0.042204238	0.164312228
0	0	0	0.009146991	0.093859473
0	0	0.064746984	0.006704998	0
0	0	0.074717124	0	0.152543568
0	0	0.037590686	0	0.288735473
0	0	0.050152559	0.017208088	0.044597603
0	0.004706185	0	0.068654578	0
0	0	0.020937366	0	0.081000111
0	0	0.044301371	0.012177399	0
0	0.018369807	0.027885627	0.013041673	0.141324207
0	0	0.021879786	0.007922577	0.059971938
0	0	0.047996153	0.124047388	0.016184766
0	0	0.053007796	0.025857213	0
0	0	0.01139328	0.046061577	0.347293594
0	0.103310382	0	0	0.168327045
0	0.001220566	0.086660761	0	0.038266464
0	0.008094385	0.017237378	0.000382398	0.276198992
0	0	0.041911358	0.017669844	0.155431838
0	0	0	0.003412195	0.227314287
0	0	0.023028995	0.005475826	0.075199167
0	0.002283584	0	0.022129876	0
0	0	0.025290064	0.004819976	0.356650736
0	0.036145558	0.038045368	0.115787184	0.054277968
0	0	0.014799077	0.013127839	0.064093644
0	0	0.064944095	0.057592295	0.085168938
0	0.022304383	0.00485112	0.026549991	0.017131102
0	0.000601352	0.033533589	0.024912116	0.044374655
0	0	0.027049296	0.027341942	0.01107122
0	0	0.035095181	0.036630292	0.045779735
0	0.04204814	0	0.027785037	0.186690135
0	0.045543611	0.025801799	0	0.171588057
0	0	0.038960191	0	0.248658117
0	0	0.044972368	0.033468624	0.076322447
0	0.060661052	0.005409346	0.045776401	0.029924778
0	0	0.027623276	0.038299188	0
0	0.018560323	0.032473665	0.018475673	0.024527485
0	0.043094529	0.029376919	0.038444576	0.017941537

0	0.017932408	0.034795899	0.011908264	0.036989131
0	0.009265892	0.011790522	0.019390258	0.082597493
0	0.000677813	0	0.024748076	0
0	0.011573909	0.053665796	0.027222705	0
0	0.045464702	0.034572113	0.04831697	0.004623304
0	0.002969962	0.017302834	0.019174952	0.079912721
0	0	0.056522026	0	0.016576985
0	0.008323027	0.007595657	0	0.13202769
0	0.020594442	0	0.033577404	0
0	0	0.055057707	0.010604244	0.01746688
0	0	0.092708384	0	0.11678919
0	0	0.006656675	0.024798962	0
0	0.007102507	0.013204904	0.030259458	0.06422049
0	0.036957878	0.001589662	0.019292565	0.04514615
0	0	0	0.040697565	0.028288444
0	0	0.081905297	0.024102365	0.007474981
0	0.007685	0.005760201	0.00102752	0.12866323
0	0.106354961	0.090436348	0.024086573	0.116713562
0	0	0	0.030466929	0.046342539
0	0	0.00214494	0	0
0	0	0.052407454	0.036831166	0
0	0.04832554	0.013426211	0.02050704	0.091178224
0	0.004720094	0.046847919	0.013773331	0
0	0.09344931	0.016706437	0.049563159	0.055990694
0	0	0.03712628	0.041209632	0.0323732
0	0.058094737	0	0.057071125	0.025098498
0	0	0.02889795	0.060420414	0.071552417
0	0	0.026777634	0.048758335	0
0	0	0	0	0.031828338
0	0	0.018880602	0	0.04236342
0	0.007061572	0	0	0.154546777
0	0	0.077329421	0.026672125	0.024819503
0	0.020191435	0	0.019298495	0.054622535
0	0	0.030442659	0.020875	0
0	0	0.022595922	0.028487888	0.056566607
0	0	0.000921694	0.031162524	0
0	0.030523321	0.032271375	0.010927762	0.018805371
0	0	0.034030695	0.014192705	0.067526633
0	0	0.009279854	0.020595051	0.037132642
0	0	0.021952476	0.072711667	0.008561695
0	0.00435456	0.016810222	0.00438658	0.270694199
0	0	0.068009519	0.010975848	0.161968781
0	0	0.050517651	0.054553646	0
0.01364519	0	0.008120407	0.012478939	0.101880034
0	0	0.057329731	0.306533474	0
0	0.010593799	0	0.037808317	0.213701938
0	0.02094996	0	0.010189772	0.070838636
0	0.009337447	0.027705255	0.026790499	0
0	0	0.068315264	0.042591086	0.007763203
0	0	0.035586245	0.026309239	0
0	0.013694074	0	0.109679021	0
0	0	0.11006674	0.018626589	0.049984613
0	0	0	0.032757787	0.032762808
0	0.008315878	0	0.008456629	0.153073275

0	0.046222445	0	0.034942917	0.20233477
0	0	0.009974563	0	0.157189013
0	0	0	0.089447814	0
0	0	0.113847195	0.020192385	0
0	0	0.085534897	0	0.011152434
0.028194423	0.00254874	0.002347845	0.028535641	0.127677493
0	0	0.036306944	0.014978366	0.028834742
0	0	0.037303164	0.018630299	0.129871703
0	0	0.054464378	0	0.038613608
0	0.016672575	0.010136798	0.112029734	0.05572633
0	0.014333721	0.019457102	0.064336731	0.087699262
0	0.013494075	0.005716345	0.07847544	0.10868836
0	0.006674939	0	0.029066951	0
0	0.006439349	0	0.013897708	0.048447116
0	0.014709247	0.047948456	0.050182228	0
0	0.038483429	0	0.023352552	0.182565721
0	0.048418023	0	0.019769822	0
0	0.014903543	0	0.029414379	0.13527472
0	0.013917398	0.036719858	0.030981803	0
0	0.017458819	0	0.016197827	0.073752418
0	0	0.027577597	0.023130798	0.03510151
0	0.010214548	0.011035782	0.044166065	0.079663738
0	0	0.04314972	0.029470131	0.036153146
0	0	0	0.055637454	0.036205548
0	0	0.025726691	0.0265245	0
0	0.005880107	0.030742862	0.041375728	0.065081351
0	0.011109243	0	0.103480422	0.070900029
0	0.016484849	0.008438432	0	0.140111345
0	0.014646632	0.03006911	0.018880508	0.016121311
0	0	0.035184706	0.01604164	0.012253381
0	0	0.094424732	0	0.010193669
0	0	0.029548211	0.015959211	0.000773888
0	0	0.04520961	0.019315434	0.080766704
0	0.010505232	0.052898844	0.01387241	0.054102223
0	0	0.038264265	0.047024574	0.208475187
0	0.000356589	0.004587843	0.055669592	0
0	0	0	0	0.005057311
0.029470255	0	0	0	0.010318601
0	0.021785644	0.020299176	0.005874705	0.104091853
0	0	0.017777179	0.071342542	0
0	0	0.031791225	0.084559179	0.103969643
0	0	0.053030601	0.027024215	0
0	0	0.015720896	0.00251861	0.015960624
0	0	0.010551903	0	0.112884848
0	0.014133358	0.056863294	0.048252201	0
0	0	0.016720878	0.032731329	0.072708977
0	0	0.035259514	0	0.127169835
0	0.001119098	0.031534299	0.015569683	0.003941505
0	0.005170069	0.029459392	0.029161397	0.006637903
0	0.004592598	0.057884804	0.022289738	0.012938051
0	0	0.125930794	0.022316787	0.033391477
0	0	0	0.007196498	0.156884698
0	0.02306297	0.000670403	0.008842477	0.026247413
0	0	0.031096279	0.038446076	0

0.002374421	0	0.04677228	0.019716391	0.019267743
0	0.026538078	0.020451321	0.034493493	0
0	0.073523583	0.013171676	0.024528287	0.093619351
0	0.004187222	0.020861398	0.025595976	0
0	0	0.039206315	0.018442161	0.018651037
0	0	0.036221849	0.051793561	0
0	0.013002948	0.033810406	0.001224882	0.365623724
0	0	0.023423444	0	0.388676536
0	0.018377183	0.018344419	0.038579475	0.240791174
0	0.012554675	0.029000901	0.01511308	0.036424876
0	0.014827326	0	0.001819485	0.067140998
0	0	0.022540826	0.009371783	0.054695564
0	0.008917783	0.006557047	0	0.015801354
0	0	0.009435976	0.034211186	0.116340368
0	0	0.033269305	0.03086608	0
0.005865339	0	0	0.000363142	0.061205443
0	0.009471811	0	0.012106648	0.040594262
0	0	0.004759761	0.017047958	0
0	0	0.027283669	0.038691824	0
0	0	0.012422489	0.000876471	0.055747296
0	0	0.01582513	0.025946796	0.011893587
0	0.014568458	0.025938001	0.033104669	0
0	0.0298433	0.002334936	0.000953707	0.095420945
0	0	0.059895437	0.087151605	0.204958548
0	0.03784136	0.005619732	0.090615793	0.040615822
0	0.048931655	0	0.106016729	0.028149038
0	0	0.000978641	0.194408232	0.05785628
0	0.030065965	0	0.003634885	0.063913915
0	0	0.032918872	0.014962956	0.03557596
0	0	0.024810482	0.017763671	0.02041768
0	0.00793999	0	0.023506997	0.030241404
0	0	0.000798949	0.068146913	0.022882752
0	0	0	0.005192466	0.068652018
0	0.084361145	0	0.014238245	0.063912906
0	0.028029181	0	0.015026347	0.00289877
0	0.005451329	0.024304884	0.020425503	0
0	0.008265499	0.009320347	0.020831225	0
0.012215584	0	0.010221039	0	0.062003309
0	0	0.000497513	0.018258283	0.035044426
0	0	0.047583001	0.06860729	0
0	0	0.064639873	0.021272007	0.000269197
0	0.01770455	0.019564143	0.013926672	0.030169674
0	0	0.044734581	0.033310638	0
0	0.00405472	0.031116631	0.012497129	0
0	0	0.02090816	0.002568905	0.044984166
0	0.003626681	0.000615133	0.058557675	0
0	0	0.07427669	0	0.29151943
0	0.000309636	0.03544933	0.109877134	0
0	0	0.036493418	0.012830173	0
0	0.102601162	0.029050719	0.018845734	0.141826835
0	0.000554841	0.015718561	0.069105316	0
0	0.00224075	0.043638636	0.032536608	0
0	0	0.010391145	0.047828495	0.027224683
0	0	0.031729943	0.067045805	0.001932352

0	0	0.013379573	0.060523176	0.0038405
0	0	0.049163608	0.021774781	0.032503951
0	0	0.030810188	0.074414648	0
0	0	0.068846362	0.022467754	0.023394009
0	0.072712852	0	0	0.086665385
0	0.011735432	0	0.036988474	0.067110003
0	0	0.030445544	0.014331912	0
0	0	0.067789488	0.024418957	0.044103621
0	0	0	0.024419956	0.042337318
0	0	0.007784461	0.065744604	0
0	0	0.039501803	0.029447463	0.018461173
0.01320065	0.013849854	0.009229348	0.025129469	0
0	0.024928933	0.048904853	0.026816579	0.040857977
0	0	0.04184018	0.01868831	0.049109605
0	0	0	0.089823925	0.002756879
0	0.002771198	0	0.008978269	0.111386873
0	0.017418954	0.011122521	0.001152756	0.004424029
0	0.026627353	0.01271143	0	0.152307759
0	0	0.018427377	0.064342096	0.061088316
0	0	0.019316179	0.012105314	0.057055496
0	0	0.019286355	0.037614128	0.03665538
0	0	0.012272044	0	0.158718974
0	0	0.064393269	0.056731571	0.019171021
0	0	0.031705969	0.083482895	0
0	0	0.031796622	0.000156161	0.174144079
0	0	0.024790608	0.033697127	0.060680987
0	0	0.030943769	0.010731893	0.046028044
0	0	0.030036421	0.048683465	0.101664703
0	0.024018587	0.007224654	0.01690324	0
0	0.003342974	0.005032646	0.008773609	0
0	0.024183109	0	0.020429575	0.094324116
0	0.0237419	0	0.028764705	0.10485333
0.041769057	0	0.001581028	0	0.002473366
0	0.044644329	0	0.022285018	0
0	0	0	0	0.227129575
0.022170761	0	0.0130485	0.027517991	0.01014062
0	0	0	0.059983957	0.020768975
0	0	0.030958798	0.035845383	0.009238047
0	0	0.041001883	0.020382662	0.059397569
0	0	0.039999323	0.031133548	0
0	0.013503081	0.008813065	0.074312674	0
0.00425064	0	0.047410076	0.017810076	0
0	0.003084809	0	0.027306525	0.081755072
0	0	0.051500565	0.059036147	0.094473162
0	0.022321288	0	0.024192039	0
0	0.058142309	0	0.026181404	0.190959878
0	0	0.052170303	0.00471168	0.053610788
0	0.005857125	0.01197548	0.015485757	0.047395515
0	0	0.031291224	0.025432967	0
0	0	0.033643865	0.039381186	0
0	0	0.025746794	0.015821168	0.07183295
0	0	0.011668337	0.047400645	0.019984167
0	0	0.033382454	0.051473361	0.005241133
0	0.013595134	0	0.106460812	0.207415071

0	0	0	0.164985701	0.058910157
0	0.022663986	0.033554342	0.219560638	0.012344048
0	0	0.028167416	0.020577067	0.08989065
0	0	0.048956527	0.000640715	0.025838127
0	0	0.039003231	0.063248388	0.202686404
0	0.02212879	0.037127448	0.085063068	0.134668214
0	0	0.045363782	0.027817188	0.076400703
0	0.100939503	0.001685218	0.058545784	0.064387404
0	0	0.053337064	0.166273776	0.176245773
0	0.050613131	0.021965988	0.0414916	0
0	0.068499167	0.014949542	0.11340213	0.183330169
0	0	0.034871168	0.05132382	0.003424107
0	0.012263532	0.022237447	0.020694881	0.032265186
0	0.012647081	0.004518846	0.045735444	0.05844112
0	0	0.060435712	0	0.049880656
0	0	0.001992775	0.022580899	0.015177255
0	0	0	0.013171651	0.057269256
0	0	0.00330207	0.027196199	0
0	0	0.032413586	0.028392043	0.021881971
0	0.011937179	0.033351069	0	0.036711506
0	0.069985786	0.076880059	0.061800188	0.005178427
0	0	0.077946956	0.029740314	0.047112472
0	0.012405394	0	0	0.229118537
0	0	0.029846466	0.016648493	0
0	0	0.030399766	0.024791542	0.026616833
0	0	0.044009811	0.003612632	0.003085099
0	0.057645302	0	0.034443771	0.016091174
0	0	0.04698575	0.053607567	0.030009254
0	0	0.036321556	0.004619739	0.020149445
0.004217329	0	0.030935196	0.017256928	0.032373585
0	0.057064692	0.021469135	0.078478309	0.010247924
0	0.008309969	0.029191464	0.106400181	0.010996601
0	0	0.02331191	0.027054211	0.145178255
0	0.067848867	0.033191699	0.122319918	0.018327101
0	0.094824607	0.028514125	0.075951119	0.175091302
0	0.062258259	0	0.033878962	0.086558561
0	0.063703735	0	0.055155194	0.118344884
0	0	0.02628002	0.014682646	0.119132353
0	0	0.061071332	0.033487444	0.043965413
0	0	0.046722169	0.03099515	0.011255881
0	0	0.065572474	0.027568822	0.01157589
0	0	0.006651583	0.049842937	0.000413492
0	0	0.011757285	0.065226986	0.128756506
0	0.025022829	0	0.015277149	0.010333123
0	0	0.035360207	0.044286906	0
0	0.01004529	0.025584448	0.10188149	0.036905514
0.024448292	0	0.011737437	0.000467363	0.007067975
0	0.009223976	0.059545795	0	0.093609458
0	0	0.019890265	0.032125084	0.025454928
0	0	0.014551459	0.027249292	0.108353226
0	0	0.046533426	0.021540051	0.008493523
0	0.019736551	0	0.010003395	0.046311527
0	0	0	0	0.466930592
0	0	0.033552104	0.014174332	0.082456689

0	0.044415474	0.030808769	0.077574949	0.137115198
0	0.019378728	0.029195613	0.077574997	0.047562777
0	0.024484733	0.020813881	0.071780221	0.137862208
0	0.01520811	0.005869129	0.053137191	0.067026987
0	0.017042933	0.017965324	0.009587156	0.052433161
0.006676788	0	0.026541653	0.028231112	0.01134873
0	0	0.048979697	0	0.237852566
0	0.003753995	0.003727539	0.022080518	0.056088142
0	0	0.025759791	0.059123466	0
0	0.004754702	0	0.064644729	0
0	0	0.035637919	0.021868904	0.029864074
0	0	0.03418421	0.035147223	0.10053743
0	0	0.011095594	0.092531653	0.098518208
0	0	0.006856514	0.01558152	0.018008795
0	0	0.015135598	0.022221313	0.06346644
0	0	0.016537353	0.030371446	0.103274058
0	0	0.038054037	0.043587381	0.038974079
0	0.010134043	0.01453711	0.056548688	0
0	0	0.035869086	0.01462957	0.00442769
0	0	0.009504658	0.026308343	0
0	0	0.018581478	0.031823963	0.027663326
0	0	0.013858727	0.014230775	0
0	0	0.014951602	0.024200564	0
0	0	0.111910495	0	0.252026699
0	0	0.046908357	0.015648633	0.072555588
0.000141709	0	0.030353975	0.04940473	0.018275243
0	0	0	0.081899304	0
0	0.012688116	0	0	0.17190037
0	0.009448245	0.004845221	0.035079497	0.070519521
0	0.044901377	0.007841939	0	0.154660087
0	0	0.087945248	0.006484284	0.048893146
0	0.001764878	0.035567662	0.02818289	0.070071236
0	0.006588444	0.006956755	0.107012033	0.186808086
0	0	0.017482658	0.142691116	0
0	0.002027156	0	0.017685182	0
0	0	0.00647062	0.050639716	0
0	0.09181805	0	0.066794472	0.094286062
0	0.008439981	0.015877179	0.086698956	0.389617229
0	0.092939626	0.001893265	0.017759389	0.012895895
0.007493903	0	0.051557352	0.006143555	0
0	0	0.025905011	0.009609989	0.110776647
0	0.015358466	0	0.006162707	0.231554468
0	0	0.013122869	0.010503415	0
0	0	0	0.081369699	0.032082145
0	0	0.010709837	0	0.159511959
0	0	0.087649313	0.014573586	0
0	0	0.041732714	0.04530266	0.134590277
0	0	0.066323367	0	0.059202488
0	0	0.012265243	0.015260583	0.066743921
0	0.016345683	0.110801052	0.021437072	0.025686699
0	0	0.047267062	0.024308469	0.046071701
0	0	0.03094905	0.006587511	0.077193424
0	0.01393328	0	0.035327272	0.018517936
0	0.003902641	0	0.02073163	0.123213399

0	0.005418532	0.002156012	0.037125274	0.013018409
0	0	0.053377613	0.033855462	0
0.003587141	0	0.046959788	0	0.031129443
0	0	0.025470959	0.016211968	0
0	0.018316163	0.014781857	0.161703883	0.2386012
0	0.011323207	0.010727949	0.025151824	0
0	0	0.056703964	0.01803071	0.0410122
0	0.027659097	0.014494088	0	0.08346372
0.002208926	0	0.040659698	0.115289919	0.035500611
0	0.020847704	0	0.061500305	0.227275396
0.002578348	0	0	0.029855945	0.056151321
0	0	0	0	0.094936004
0	0.00248406	0.004284586	0.068824763	0.12497915
0	0	0.067602448	0.012454405	0.035425103
0.014483885	0	0.058624101	0.003862425	0.040530426
0	0	0.072406671	0.004820312	0.119609417
0	0.005130775	0.007752298	0.019867281	0.12107773
0	0	0.072351823	0.022454851	0.027363293
0	0	0.022582777	0.00723745	0.002565356
0	0	0.017494377	0.045820775	0.038672665
0.020684629	0	0.134898627	0.006023203	0.002605923
0	0	0.019493571	0.041170251	0
0	0.017385642	0.015761306	0.020936443	0.107480307
0	0.014252276	0	0.015574857	0.077185776
0	0.029698644	0.031731781	0.027089655	0.074932418
0	0	0.045259371	0.025074672	0.002519344
0	0.057590363	0.017944269	0.047602712	0.008652955
0	0.065423483	0.003697145	0.150230571	0.031743818
0	0.021318498	0.027016571	0.119595581	0.103836353
0	0.058292732	0.006212982	0.053989837	0.14636173
0	0	0.054958839	0.027276099	0.006080278
0	0	0.019935108	0.015896532	0.114847176
0	0	0.016282857	0.047883159	0.030260727
0	0	0.052628823	0.105736599	0
0	0.054754621	0.034361979	0.056740761	0.073554643
0	0.018661638	0.029881063	0.000559699	0
0	0	0.035306417	0.043348384	0.043656864
0	0.045119913	0.002195273	0.088752893	0
0	0.051327228	0.032369971	0.080548056	0.074958334
0	0.084396632	0	0.136033933	0.089798148
0	0.015703231	0.030229827	0.074680084	0
0	0.026421012	0.060347755	0.029726426	0.071869094
0	0.07276454	0.001990956	0.052074259	0.134204691
0	0.018219518	0.005356552	0.03889964	0.126747311
0	0.017005738	0.019410782	0.03994647	0.160623847
0	0.063018632	0	0.064132087	0.027163991
0	0	0.013043451	0.006338289	0.186874651
0	0	0.026699879	0.009558821	0
0	0	0.051137814	0.031122948	0
0	0	0.016102502	0.032479301	0
0	0	0	0.022367119	0.010518359
0	0	0.023298332	0.000498678	0.062801772
0	0.015453215	0	0.03891863	0.034224066
0.021987946	0	0.01518402	0.009174749	0

0	0	0.018394402	0.038900274	0
0	0	0.044093124	0.028442686	0.023295731
0	0	0.009743228	0.052418476	0.088079049
0	0	0.04998095	0.027702776	0
0	0	0.054028233	0.019344531	0
0	0	0.011195287	0.049121375	0.080663192
0	0	0.02101257	0.024779964	0.008694251
0	0	0.036113755	0.035981061	0.137132219
0.007949977	0	0.02069449	0.000210011	0.01665791
0	0	0.025778974	0.016812633	0
0	0	0.036327133	0.014191618	0.06000629
0.00667598	0	0.008672683	0	0.09207145
0	0	0	0.029284071	0.064649178
0	0.0542627	0	0.15724476	0.136393341
0	0.026860324	0.017270664	0.029124196	0
0	0	0.012697067	0.022321316	0.091856743
0	0	0.098523787	0.015486656	0
0	0	0.039253038	0	0.002968017
0	0	0.069443955	0.026184259	0.06562013
0	0.004425249	0.033973628	0.020684945	0.111712576
0	0	0.006356078	0	0.102673477
0	0.018686361	0	0.110700863	0.087319617
0	0	0.030560895	0.0115354	0.011580307
0	0	0.024742216	0	0
0	0	0.01827059	0.015452716	0.031217191
0	0.004358417	0.055209145	0	0.04835349
0	0	0.035997025	0.034004909	0
0.021097318	0	0.041914518	0.022312228	0
0.044596493	0	0.032734608	0.033007133	0
0	0	0.025685386	0.029929471	0.014141689
0	0	0.023070264	0.00804041	0
0	0	0.014201548	0.032273039	0.093678397
0	0	0.032913083	0	0.15146488
0	0	0.02926691	0.023147785	0.019722148
0.002449478	0	0.002091897	0	0.169650979
0	0	0.043145932	0.061146048	0
0	0.03484424	0.002519217	0.045452979	0.109575318
0	0.011918419	0.002423103	0.078901364	0.052061581
0	0	0.01872996	0.004918533	0
0	0	0	0.070850922	0.028586786
0	0	0	0.001642514	0.000563558
0	0.030466307	0	0.020725821	0.062115846
0	0.022052249	0.004576416	0.016130333	0.104958814
0	0.008387451	0.008255817	0.015817495	0.002447842
0	0	0	0.026247127	0.137822369
0	0.010357594	0.011000245	0.041112782	0.059315936
0	0.012254919	0.024628951	0.043681533	0.052466908
0	0	0.03900009	0.009832467	0
0	0	0	0.017899564	0
0	0	0.034794075	0.092554274	0.054031335
0	0	0.025519582	0.004120492	0.057003841
0	0.028626819	0.159641407	0.034718698	0.046367603
0	0	0.028728926	0.216184017	0.010674429
0	0	0	0.043194177	0

0	0.013974659	0.036109977	0	0.084065582
0	0	0.040886849	0.009584745	0
0	0	0.025126476	0	0.132872348
0	0.023260015	0	0	0.254884605
0	0.032167091	0.070218442	0.044384623	0.013090643
0	0	0.022520978	0.013452065	0.051165512
0	0.028413865	0.02996366	0.03814354	0.013785172
0	0	0.000638593	0	0.162952045
0	0	0.044924	0.058987338	0
0	0	0.038544482	0.004959589	0.066402087
0	0.024389948	0	0.009763636	0.024896571
0.059287592	0	0.007384932	0	0
0.010131923	0	0.00119737	0.00543338	0.056018953
0.003498934	0	0.021586757	0.01323907	0.025649196
0	0.020114854	0	0.050730817	0
0	0	0.042359623	0.064978989	0.220519128
0	0	0.03296137	0.026770584	0
0	0	0.005564208	0.009964761	0.126782442
0	0	0.008635491	0.013633684	0.100705146
0	0	0.023571928	0.021996652	0.007003684
0	0.00261995	0.004025063	0.133089675	0.179347872
0	0	0	0.069004864	0
0	0	0.023812159	0.059839041	0
0.011011365	0.01223227	0	0	0.013123552
0.036028047	0	0.021964567	0	0
0	0	0.063798923	0.009998194	0
0	0	0.030863832	0.006395816	0.059358335
0	0	0.024865587	0.016583232	0
0	0.008287656	0.035085863	0.006471233	0.007574161
0	0	0	0.082658758	0.013381393
0	0.09489962	0	0.032487369	0
0	0.069190953	0	0.049986574	0.123332662
0	0.002855604	0.002719183	0.04199133	0
0	0	0.045022415	0.01543589	0.006287043
0	0	0.081582051	0.004993825	0.070956886
0	0	0.06470289	0.004894404	0
0	0	0.036900804	0.035353484	0.079753183
0	0	0.0072007	0.024888155	0.05457152
0	0	0.041634864	0.045451462	0
0	0	0.068639086	0.04069448	0.042390077
0	0	0.05366309	0.046525789	0.038680686
0	0.042966418	0.00523581	0.031491745	0.033759767
0	0	0.053426334	0.003177379	0.103977071
0	0	0	0.016410375	0.012879395
0	0.011127827	0.01133079	0.045290527	0
0.042620138	0	0.063280945	0.027889835	0.013589507
0	0	0.022970488	0.003554278	0.072494464
0	0	0.087949535	0.009531694	0
0.031969125	0	0	0.003942219	0.374330142
0	0.03615316	0	0	0.145871176
0	0	0.040202063	0.00657304	0.151439748
0	0	0.001600296	0.013810198	0.054038127
0	0	0.021669725	0.010747983	0.230528002
0	0	0.049637494	0.02897694	0.042035208

0	0	0.0436591	0.009975061	0.120538818
0	0.075167794	0.032831651	0.028749384	0.109778485
0	0	0.053426453	0.00604275	0
0	0	0	0.011801843	0.111652379
0	0	0.068849848	0.033963431	0.020029199
0	0.057947586	0	0.00160649	0.033554771
0	0	0.013972229	0.158023654	0
0	0.074860645	0.039332341	0.117968054	0.114516362

Macrophages.M1	Macrophages.M2	Dendritic.cells.resting	Dendritic.cells.activated
0.101227335	0.16029829	0.000387289	0
0.027880925	0.280185996	0.000762887	0.014585384
0.057163607	0.321196186	0.000856408	0
0.041725318	0.20384904	0	0.045829722
0.032635819	0.204045467	0.046068514	0.001131023
0	0.261553271	0	0.145109778
0.012932118	0.357477703	0	0.077265038
0.010556995	0.214697026	0	0.011816364
0	0.433273124	0	0.00380486
0.104026629	0.203965929	0.007652371	0.009484755
0	0.273072483	0.009370009	0.060211217
0.092282301	0.165649376	0	0.047737865
0.026834278	0.200436064	0	0
0.103978208	0.275960802	0	0.048820738
0.022396456	0.269196228	0	0.026507175
0.015764294	0.245320409	0	0.0159788
0.041862467	0.429506917	0	0.097181511
0.080128299	0.178060675	0	0
0.059669434	0.266811197	0.006509664	0
0.116637222	0.233497835	0.046181801	0
0.034394245	0.187016917	0.078628894	0.00970048
0.016926279	0.390383603	0	0.027973425
0	0.263467249	0	0.047054444
0.028085425	0.199384328	0.017323548	0.00805974
0.024435151	0.390685476	0	0.044596151
0.04828127	0.208648054	0.088994807	0.004233751
0.069570053	0.246088119	0.039768836	0.010024587
0.014839304	0.273926636	0	0
0.068806474	0.101171338	0.017733677	0
0.015232901	0.22132575	0.083803564	0.003341008
0.055715856	0.320366907	0.005208261	0.004296091
0.049844738	0.22220767	0.01458244	0
0	0.276680529	0	0.101610646
0.027945643	0.316427588	0	0
0.102579845	0.248966554	0.008319975	0
0.07237138	0.225658409	0.000104087	0
0.018517855	0.482411661	0.005968606	0
0.056652412	0.246279424	0.059727203	0
0.018393505	0.502810844	0.016057573	0.016972784
0.061539609	0.221007721	0.045453296	0.000841682
0.047731381	0.363171007	0	0.022406585
0	0.222974691	0	0.0589097
0.004409393	0.271050312	0.005746092	0.024774349
0.042543583	0.220846357	0	0.021966905
0.007524034	0.254234261	0.005827275	0.035909103
0.108372295	0.161636589	0.019462283	0
0.07177455	0.204529689	0.050715148	0
0.040929819	0.315109768	0.016961236	0
0.08171613	0.261248048	0.044710833	0.013123039
0.145656768	0.18983433	0	0
0.005320572	0.1724921	0	0.044459197
0.025290593	0.166188687	0	0.011131534
0	0.086208384	0	0.108433296

0	0.237164118	0	0.002189123
0.015256632	0.238070799	0.002227917	0.05151543
0	0.265760258	0.03004079	0.019884004
0.037828495	0.188204635	0.017570967	0
0.015338655	0.23919348	0.024641019	0
0	0.218402352	0	0.063796605
0.044045418	0.308127121	0	0.090309821
0.117018236	0.30599405	0	0.044488851
0	0.198853659	0.171142049	0.088865298
0.054177395	0.218858295	0	0
0.011388519	0.047212794	0	0
0.050142469	0.35857025	0.021460624	0.000304314
0	0.135076468	0.076569631	0.047682291
0	0.392449859	0.087246458	0.090374696
0.036554727	0.312855719	0.091777601	0.037258945
0.124314626	0.23149338	0.019889788	0
0.009783494	0.392757361	0.042697873	0.010126133
0.014654072	0.434290456	0	0
0.103268464	0.284439435	0.092479215	0
0.003197893	0.209382623	0.024334879	0.006579747
0.022066945	0.208582213	0	0.021131627
0.031337798	0.046566581	0	0.04328124
0.062946758	0.303024295	0.015627571	0
0.018976622	0.19160189	0.051613771	0.021754999
0.118025969	0.18610269	0	0
0.058611004	0.29201388	0.012859896	0
0.065534121	0.202780207	0.003159213	0
0.098950943	0.208147282	0	0
0.016372213	0.391675462	0.02946675	0
0.051771176	0.247212274	0.056002327	0.010667938
0.0052007	0.304638791	0.034474902	0.062844535
0.011686438	0.045662169	0	0.004620035
0.065001383	0.260129731	0.012397615	0
0.018362746	0.260950224	0	0
0.063031915	0.210137824	0.003016863	0
0.011684693	0.2728879	0	0.023695156
0.091825624	0.271407362	0	0
0.030336163	0.319769519	0	0.003384045
0.005048751	0.265884347	0	0.028074266
0	0.225700556	0	0.048188484
0.008304295	0.530179828	0.029217445	0.016618367
0.068788706	0.226394154	0.002043381	0.015015856
0.037293323	0.339558054	0	0.022436026
0.023197492	0.313953692	0	0.004091286
0.077706355	0.179041534	0	0
0.066730467	0.246307284	0.015617137	0
0.021186316	0.186002106	0	0.009661318
0.057967555	0.34399189	0.009241933	0
0.020294809	0.319167956	0	0
0.07242751	0.335894636	0.011959142	0
0.055987908	0.294542244	0.018680968	0
0.091226701	0.269469726	0.064616125	0.005907748
0.028814133	0.273570048	0.000676618	0.027470078
0	0.113252489	0	0.053269553

0.063940822	0.429276252	0.033085215	0.045129993
0.008350436	0.452736549	0.007713062	0.01546447
0.094220414	0.332062273	0.023516678	0.004819421
0.044118555	0.142549874	0	0.001004207
0.085033247	0.150949264	0	0.137350907
0.022861668	0.469036158	0.050175609	0.069152148
0.013048133	0.262422002	0.0196345	0.035007016
0.176439775	0.144660333	0	0
0.043730948	0.365439802	0	0.040100481
0.09062532	0.150327388	0.020008411	0.001110472
0.012095831	0.23890789	0.162706201	0.017728819
0.018201192	0.300941927	0.002463554	0.002886504
0.029185099	0.348041263	0.185666459	0.056026663
0.097834526	0.326592946	0.003034805	0
0.036959093	0.33285579	0.053512516	0.01937757
0.051826681	0.209458718	0.016570103	0.03457471
0.043505918	0.392376967	0	0.008269052
0.06724017	0.045854831	0.068302914	0
0	0.475431081	0.046766245	0.026173947
0.092672691	0.209361628	0.008006093	0
0.001626844	0.518031133	0.013567916	0.033215787
0.117915175	0.108349139	0	0
0.083906435	0.319339221	0.017815202	0.003725848
0.049992176	0.250837956	0	0.024766344
0.010431433	0.201661289	0.00329514	0.015103392
0.016341905	0.36065281	0	0.054866413
0.028192507	0.299012956	0.00398246	0.006250846
0.010907664	0.342995002	0.081498163	0.024213728
0.012160898	0.16443756	0	0.016927853
0.109429249	0.273241611	0	0
0.001778185	0.328624211	0	0.013270533
0.139005144	0.159650646	0.031765042	0
0.129312194	0.201728518	0.043751221	0
0.004666891	0.256987526	0	0.014197275
0.030927343	0.318393914	0.020752102	0.003286411
0.033352328	0.554472985	0.007765315	0
0.094155648	0.206159531	0.009554827	0
0.040924729	0.135696044	0.018877105	0.000183645
0.009123168	0.405043718	0.052322406	0.017294376
0.0292711	0.277649044	0.056822987	0
0.045340303	0.302524642	0.003202351	0
0.045148733	0.247435693	0.00044386	0
0.082594867	0.328870116	0.023009211	0.001593155
0	0.037281768	0	0.154372093
0	0.132145037	0.035880862	0.017428701
0.007043438	0.085919341	0.046922761	0
0.046594543	0.341289604	0.064298788	0.070629189
0.103487417	0.133857133	0	0.001906189
0.075451518	0.344251658	0.067114315	0
0.028114356	0.331924408	0.12099698	0.028056427
0.033495916	0.290774435	0.03249888	0.007145664
0.04230266	0.110595258	0	0.152824768
0.003529592	0.395027149	0.025413105	0.040007378
0.059243316	0.418237801	0.003939846	0.035744192

0.025882087	0.406480261	0.005435907	0
0.04734585	0.280511892	0	0
0.001721321	0.435855775	0.019366916	0.003344139
0.075849397	0.412987681	0.022819356	0.005579916
0.092059091	0.227874968	0.067985174	0
0.053676344	0.107331179	0	0
0.113422023	0.134618039	0	0.00271821
0.067705919	0.29287782	0	0.069778507
0.013367985	0.203072227	0.004102934	0.009635723
0.023844292	0.282964507	0	0.090870966
0.006093029	0.269996689	0	0.046310068
0.015377945	0.462591132	0	0.016099685
0.049742528	0.331254914	0.133694888	0.017699066
0.054159893	0.500164546	0.003204707	0
0.01035869	0.445234237	0	0.003356029
0.044809738	0.379015929	0	0
0.029989928	0.331523313	0	0.057345072
0.046462624	0.399190413	0.033240544	0
0.083531736	0.29128213	0.018904172	0
0.061082686	0.202082899	0.003833182	0
0.148316148	0.239382643	0.026337603	0
0.051345007	0.214238847	0.001236949	0.024897367
0.026265175	0.111762846	0.021398516	0.009380772
0.011101666	0.451986509	0.059924206	0.005076394
0.007942778	0.316151691	0.220659335	0
0.091880479	0.192729652	0.021238211	0
0.013287273	0.281359339	0.073044592	0
0.275876354	0.122273886	0.001553616	0
0.073003418	0.223407918	0.022883107	0
0.032407616	0.105983448	0.002080929	0.004411327
0.004591833	0.243696943	0.140660181	0.055834106
0.040312213	0.172874599	0.005122833	0
0.078250172	0.117627506	0.001506047	0
0.012605626	0.076642989	0	0.052492965
0.057990257	0.137983018	0.003542753	0.02395638
0.048262316	0.235659427	0.007771942	0.008843182
0	0.301464125	0	0.077624127
0.114690282	0.436014352	0.004205001	0
0.05247939	0.368374997	0.006401362	0
0.042894567	0.334940724	0.025557779	0.075759125
0.012520786	0.240125744	0.015101384	0.027533111
0.017949941	0.235642274	0.021315552	0.009104934
0.029033414	0.44050246	0.014080275	0.006722707
0.050191419	0.264609188	0	0
0.066535505	0.237579847	0.027755965	0
0.057642765	0.348336819	0.035803187	0.003264371
0.157734411	0.140344588	0	0.044857952
0.057647452	0.262117342	0.005594052	0.027720023
0.146325829	0.254291802	0.001780309	0.000360403
0.029836739	0.133022494	0	0.12000985
0.122861155	0.157267338	0.006593679	0.013739628
0.010947529	0.308576802	0.114415611	0.003164855
0.095432161	0.223986975	0.005040753	0
0.090006896	0.307454054	0.14639453	0.005728677

0.112935739	0.125384616	0.017147459	0
0.103392536	0.267575134	0.008057732	0
0.112160813	0.323373071	0.011960828	0.000463793
0.080651546	0.315565229	0.044179836	0.001687167
0.09898374	0.35463091	0.016897912	0.009753473
0.014190695	0.31299874	0	0.0260498
0.05375447	0.27836603	0.00082959	0
0.021571366	0.243298789	0.016406396	0
0.107441748	0.102364019	0.00675855	0
0.077838133	0.346494369	0	0
0.104840659	0.191987436	0.027694069	0.002512061
0.066994256	0.341634865	0	0
0.074974604	0.220497936	0.000621486	0
0.034526423	0.542153148	0.00437972	0
0.001643716	0.273399488	0	0.02221543
0.094887618	0.24977769	0.014300518	0.001919515
0.040012503	0.311558584	0.073435231	0
0.055222178	0.207829617	0.208631985	0.003354453
0.023692976	0.025646475	0.022840354	0.01603109
0.070324044	0.34455948	0.042033607	0.008448878
0.060960588	0.0678691	0	0.014264813
0	0.338125619	0.15658382	0.046620968
0.03499302	0.357538987	0.045637504	0.010587544
0	0.331708663	0	0.035165859
0.007662572	0.364267937	0	0.075073657
0.017923862	0.312160331	0	0.052286614
0	0.372567777	0.000404018	0.035812478
0.053335223	0.254294786	0.080844385	0.007555535
0.014720881	0.313928975	0.025870877	0.019195927
0.065360348	0.22909252	0.013173011	0.000978542
0.014141839	0.235527229	0	0.015970717
0	0.162292504	0	0.339872864
0.098017203	0.136795712	0.037684613	0
0.015407674	0.122209432	0	0.061165651
0.062380099	0.199837893	0	0.105773824
0.077843145	0.376875613	0.01796463	0.075980307
0.033513696	0.128742634	0.008277911	0.01632806
0.072919188	0.262417819	0	0
0.032288312	0.33892813	0.002691036	0.008469972
0.03833795	0.264929202	0	0.002333786
0.005320867	0.067241793	0.001901434	0.00659309
0	0.516467233	0.216762402	0.001843833
0.081552958	0.225200799	0.047770906	0.00355562
0.002983079	0.214236866	0.052036166	0.011544386
0.157444862	0.173454991	0.011327655	0.096307085
0.052949203	0.25687274	0	0.039039578
0.000289902	0.300078187	0.019156791	0.023836809
0	0.411682918	0	0.114327825
0.037828099	0.28541716	0.035919403	0.015237315
0.031352795	0.053239599	0.00183238	0.020606279
0.018494771	0.309581463	0.005512209	0.006412105
0.044240632	0.497619076	0.038485886	0.026720303
0.061153698	0.348602177	0.035100125	0.006532566
0.031030235	0.288572324	0	0.006027561

0.114541652	0.145657089	0	0
0.147414321	0.210595127	0.010839624	0.002778194
0.084083805	0.23917628	0.245058878	0
0.063508381	0.295996372	0.032908138	0
0.03746531	0.167035926	0.016906664	0
0.09650879	0.278331035	0.011387974	0.011404994
0.018536686	0.219403209	0	0.036942944
0.058523508	0.297067713	0.109062837	0.005440689
0.045357405	0.482430724	0.013386391	0.009992841
0.044728616	0.347948296	0	0.013205059
0.024870469	0.221527395	0.010247872	0.038489289
0.027946474	0.248831543	0.056017081	0.002941753
0.09564033	0.12411715	0	0.031221514
0.027709344	0.395395067	0	0
0	0.374528024	0.018629759	0.092924795
0	0.288009943	0	0.008183935
0.087882355	0.153651771	0	0
0.062488091	0.355857443	0.008408695	0
0.031346812	0.384360296	0.022617389	0.003425877
0.033724124	0.150570532	0.063982186	0.000232213
0.042599066	0.33503325	0	0.060323762
0.03544745	0.356168512	0	0.093294409
0	0.302739516	0.069087428	0.053652075
0.03008664	0.200871711	0.109142922	0.025909243
0.025344541	0.479414736	0	0.015083816
0.004669457	0.586574332	0.036197058	0.017269714
0.07931036	0.320262232	0	0.046684155
0.060523567	0.206329031	0	0.039755921
0.128223948	0.379528314	0.009377876	0.017159353
0.0074147	0.314871173	0.109994379	0.006251361
0.01216308	0.513886539	0.031098142	0
0.049711305	0.467926453	0	0.03652722
0.083870715	0.263354471	0.031160409	0
0.08440291	0.407957935	0.024702302	0
0	0.253953851	0	0.118730394
0.084101733	0.191132564	0.113462344	0
0.004073358	0.311167031	0.0944711	0.044681912
0.111652398	0.301630811	0	0.004286557
0.040079363	0.226182279	0	0
0	0.186232965	0.00268872	0.025392443
0.022108458	0.300737654	0.012544238	0.018308273
0.085931024	0.367423264	0	0
0.018342839	0.08995379	0	0.01238351
0.081532455	0.289280537	0.012047932	0.022256771
0.063238135	0.32843284	0.012874491	0
0.031210475	0.206716371	0	0.005333156
0.044203294	0.295482476	0	0.071968353
0.074931016	0.204729456	0	0.007948237
0.039603767	0.16226114	0.08292994	0
0.05236023	0.386353015	0.009612579	0.009516132
0.074037024	0.32392747	0.010441389	0.011305334
0.002602493	0.347157375	0.079133755	0.087092895
0.047235535	0.324630945	0.122576451	0.01711447
0	0.359168063	0	0.034339282

0.002083368	0.397438509	0	0.0242187
0.028474465	0.244925807	0	0.048936383
0.006585299	0.058384924	0	0.001350541
0.038433062	0.122136532	0	0
0	0.213672406	0.003058115	0.041309164
0	0.305006589	0.020272849	0.078672101
0.011585085	0.483972877	0.004037311	0.011554511
0.022734134	0.254506951	0	0.062508
0.01806227	0.150308338	0.00857879	0
0.041366888	0.214862652	0	0.00447421
0.004242683	0.17333812	0.0005087	0.039321657
0.030407335	0.324207905	0.067014842	0.006812452
0.093178081	0.103259553	0.027371034	0
0.032657458	0.289832216	0.04185015	0.013782752
0.036047124	0.128508977	0	0.011874773
0.016430847	0.113775871	0.024315063	0.017004908
0.120874894	0.418590341	0.022974433	0.00474757
0.01602579	0.534071137	0.002338827	0.005315669
0.061675927	0.450326951	0.018694341	0
0	0.090176847	0	0.072926578
0.023421754	0.123589296	0.002132928	0.018029739
0.061458816	0.163694906	0.005938633	0.001422716
0	0.239030212	0	0.042050756
0.084006409	0.252177417	0.015149123	0.040681722
0	0.107708076	0.002911984	0.03524048
0.042691618	0.212453069	0.024602465	0
0.018597369	0.163061737	0	0.02053918
0.107456526	0.306776992	0.032655114	0
0.03295383	0.219152747	0.003816225	0.003254317
0.029455322	0.190735021	0	0.009817949
0.046272221	0.236901018	0.001555338	0.02700766
0	0.22735006	0	0.034054953
0.05674429	0.27499209	0.00519626	0
0.016420657	0.306210206	0	0.089573648
0.005090592	0.221192253	0.017870881	0.067583562
0	0.389692633	0	0.050198682
0	0.341374372	0	0.053048663
0.043826182	0.430076205	0	0.013144734
0.092947774	0.114557451	0	0
0.055649215	0.174308339	0.044081693	0
0	0.16736785	0	0.056990183
0.025906515	0.150146071	0.158986187	0.010605946
0.017117007	0.285350226	0.133855675	0.004507068
0.067240377	0.222398962	0.033150933	0.010490313
0.014676318	0.279505466	0.103144121	0.028383913
0	0.322871412	0.048987418	0.055731149
0.153788934	0.196214428	0.080902388	0
0.072249176	0.173212616	0	0
0.016455015	0.439578304	0.004771739	0
0.027659839	0.345051824	0	0.009733302
0.046208283	0.399165727	0.088222796	0.020337401
0.140506149	0.281174449	0.043583947	0
0	0	0.002871858	0
0.083229176	0.168695584	0	0.006462479

0.007457321	0.204055535	0	0.027194307
0.002922893	0.322026632	0.004722837	0.020134673
0.042058784	0.321268429	0.031799106	0.043692251
0.027132552	0.440036737	0	0.049843609
0.162095316	0.195611018	0.140423412	0
0	0.375682858	0.128058925	0.059523904
0.041353981	0.037319212	0	0.006643099
0.047679487	0.246185438	0	0.001069455
0.002698236	0.26002237	0	0.010001226
0.070940376	0.337780798	0.036952678	0
0.034255216	0.37544571	0.014706208	0.029511039
0.086141332	0.336466259	0	0.049396606
0.009053761	0.400134671	0.016950379	0.020388936
0.046564105	0.39857713	0.005263541	0.047243566
0.053824608	0.208263361	0.01282038	0
0.038827793	0.379043268	0.003347839	0
0.013288491	0.403220363	0	0.03903452
0.062318531	0.233213336	0.010501868	0.02895696
0.009838389	0.241183404	0.086048251	0.010485395
0.066587281	0.147166869	0.020545667	0
0.075647711	0.214250356	0.018843115	0
0.00673759	0.219560497	0.004964237	0.015811105
0.042081925	0.290273478	0.166806253	0.001209461
0.067449279	0.16429255	0	0
0.000392926	0.310998691	0.001605509	0.000883753
0.021033104	0.251153035	0.098690935	0.038825639
0.013256965	0.212712279	0.020998426	0.030091577
0.004192833	0.125202895	0	0.023227216
0.094151931	0.250497781	0.011624721	0
0.145818171	0.353728638	0	0.007245815
0.08241534	0.147958981	0	0.02392924
0.075131398	0.306282721	0	0.084486617
0.000573635	0.393505644	0	0.064337041
0.014803274	0.202904091	0.131003768	0.006053811
0.047391994	0.314668176	0.081243631	0.007992475
0.030664308	0.400787557	0.053935278	0
0.019593117	0.322374213	0	0.051438389
0.006828376	0.216333876	0.01681021	0
0.008867561	0.334307768	0.027021406	0.060771649
0.094216318	0.104622964	0.084727619	0.005401047
0.082397593	0.258997505	0.012866654	0
0.003535135	0.374093086	0.012369253	0.040570257
0.026131985	0.231366211	0.028315024	0.022355414
0.018879427	0.509147058	0.008418483	0.019830138
0.06437821	0.141326457	0	0
0.112044632	0.23745127	0.012282197	0
0	0.345071371	0.07008951	0.02651372
0	0.125338488	0.000738739	0.008679955
0.048461531	0.397625482	0	0.003213734
0.060618408	0.097275491	0.016536837	0
0.023020298	0.610537197	0	0
0.010453879	0.120481484	0.026719585	0.032338756
0	0.274447229	0	0.082580856
0.014220319	0.357098688	0.042055616	0.011353091

0.043145573	0.371903748	0.027592734	0.004192594
0.013258609	0.287630429	0.038810216	0.053418824
0.131311709	0.122799254	0.014542457	0
0.011312955	0.330358908	0.001151309	0.023113769
0.050559325	0.272639505	0.01252241	0
0.022976426	0.438546962	0.058777776	0.007871212
0.006617504	0.423965562	0	0.046972473
0	0.192849762	0.016971029	0.050935885
0.028016033	0.369150474	0.028309366	0.019616357
0.086256759	0.15417495	0.005038337	0
0.039406495	0.238991834	0	0.027664373
0.088178114	0.275255206	0.002378402	0
0.041476461	0.286137796	0.014052402	0
0.110948959	0.22928076	0	0.013406117
0.144974123	0.100655722	0.003857478	0
0.118401253	0.202792773	0	0
0	0.522789066	0.005411613	0.020372604
0.076610734	0.165359056	0.015634109	0
0.05807959	0.254301859	0.044249511	0
0.055673169	0.417569867	0.005803446	0.016445122
0.034560909	0.061868796	0.008613461	0
0.025481022	0.255087481	0.071551212	0.067840721
0.063304478	0.22529626	0	0
0.09891887	0.243563144	0	0.083426748
0.100865234	0.200139322	0.027773223	0
0.079268055	0.211616734	0.052920509	0.023310682
0	0.379240032	0	0.06442226
0.010760119	0.133420445	0	0.040935859
0.017740497	0.266716591	0	0.027702799
0.005841534	0.21879261	0	0.044629526
0.041865217	0.356244482	0.046494158	0
0.005901072	0.483532522	0	0.000756123
0.076866444	0.357107403	0	0.010107534
0.013850941	0.173586158	0	0.059702855
0.002566109	0.315640411	0	0.143598887
0.005806478	0.158663844	0	0.031060964
0.011561786	0.303910751	0.000478652	0.023449051
0.013385441	0.233447221	0	0.019226129
0.028474194	0.273283012	0	0.051810619
0.000103141	0.21590951	0	0.054395075
0.044540542	0.255149393	0	0
0	0.299157714	0	0.070358754
0.071069654	0.256723214	0	0.033849165
0.003998508	0.385382144	0	0.018414361
0.025810304	0.392883293	0.004173229	0.017665343
0.053001778	0.371143568	0.005093315	0.039949681
0.005039674	0.464144623	0.022978998	0.007581024
0.041131409	0.166255402	0.006619836	0
0.045087322	0.197439633	0.002851805	0.01312033
0.006692655	0.137288431	0.01092759	0.005967903
0.000684654	0.184819655	0	0.004077525
0.141799191	0.234231063	0.011134604	0.006392695
0.044946199	0.240627946	0.042263805	0.056246047
0.005941549	0.548485369	0.097629923	0.018374081

0.017820652	0.116041291	0.010517745	0.004634716
0.107096579	0.153754777	0.032748331	0.0002349
0.003934075	0.36221053	0	0
0.105512741	0.266169167	0.044077816	0.042369872
0.061501124	0.206426169	0.010557873	0
0	0.337103892	0.137499572	0.079680297
0	0.16814904	0	0.066420941
0.058492704	0.269698621	0.00520966	0
0.075641271	0.255146068	0.095262786	0
0.018600226	0.393703056	0.078621184	0
0.093406245	0.176442437	0	0
0.120784068	0.240931764	0.00100546	0
0.065571151	0.036362701	0.003302844	0
0.032510207	0.304879074	0.01106457	0
0	0.222971351	0.000386396	0.053430095
0.05518973	0.284908572	0.005555412	0.014109426
0.025252084	0.316763043	0.028586731	0.024980525
0.06056086	0.248634128	0	0.011995225
0.074202779	0.228106683	0.089504044	0
0.076819739	0.287733336	0	0.00474646
0.066844497	0.205241208	0	0
0.015301975	0.374714811	0.042521414	0.012422145
0.059122861	0.352205325	0.011476348	0.000610942
0.004740774	0.30465396	0.280954705	0.042193155
0.018883411	0.389034069	0.021675233	0.058703422
0.091982577	0.26393116	0.012552566	0
0.017159271	0.285952394	0.215164152	0.000452625
0.056094166	0.26219239	0.067340698	0
0.059748596	0.409363762	0.040285723	0.019907096
0.041713145	0.237500185	0.020142426	0
0.124739916	0.244404241	0.073922658	0
0.016650727	0.394582894	0.022987126	0.051609903
0.097840022	0.184141487	0.007790682	0
0.104572968	0.2880792	0.009236186	0
0.03637732	0.207152202	0	0
0	0.161234634	0.095728398	0.088430384
0.016287586	0.334772448	0	0.01803459
0.071698716	0.19818402	0.001083674	0.032554779
0.042195598	0.382193842	0.003876819	0
0.065581621	0.330362853	0.016397287	0
0.025201458	0.303193613	0.033046851	0.005577736
0.0519159	0.196377617	0	0.005843332
0.004732041	0.338045262	0	0.016865538
0.074377587	0.128056683	0	0
0.054631835	0.136257852	0.006184227	0
0.020476414	0.19882359	0	0
0.107753505	0.175384754	0.009118871	0
0.050757674	0.148233133	0.048720758	0.011179936
0.008836642	0.453679963	0	0.024792626
0.010075114	0.437821497	0.114819604	0
0.107282569	0.148647372	0.005750126	0.001416962
0.007933167	0.181771195	0.010443415	0.046342711
0	0.411269827	0.009774393	0.04551436
0.085340772	0.262787331	0.003403105	0.020350644

0.079637442	0.100923928	0.009591192	0.028028075
0.016564403	0.186515242	0.01241596	0.007784587
0.051646263	0.214507115	0	0
0	0.183069718	0	0
0.018511025	0.151362577	0	0.019507865
0.062553708	0.142506699	0	0.010422023
0.090886422	0.306494373	0.006382505	0.010491952
0.012906098	0.156253038	0	0.00085975
0.026592218	0.168363404	0.001667573	0.006880022
0.038958278	0.22394818	0	0.107435924
0.013323778	0.291990862	0	0.09504375
0.117604513	0.256616835	0.023620578	0.000736955
0.087665203	0.203339145	0.04349986	0.02604926
0	0.354429293	0.003091537	0.138248268
0.028965615	0.316874874	0	0.086344158
0.038333966	0.15033787	0	0.110079947
0	0.166748587	0	0.154861936
0	0.363532167	0.071263256	0.032944745
0.03462749	0.097551721	0	0.000750378
0	0.354722817	0.125944694	0.064337831
0.004164068	0.334011545	0	0.036109834
0	0.228326732	0	0.196886568
0.006136123	0.173937422	0.142510385	0.068428094
0.089556157	0.193147905	0.174482642	0
0.02776603	0.113978804	0	0.102941599
0	0.060852587	0	0.147293905
0	0.194208102	0	0.133795141
0.10699704	0.1901952	0	0.082806819
0.097639187	0.272497204	0	0
0.135646533	0.122337015	0	0.022939886
0	0.103023509	0	0.148626755
0.028818564	0.185999098	0	0.030996396
0.009564791	0.186829469	0.016528385	0.072095597
0.099897717	0.196522253	0	0.091912984
0.170604405	0.09213748	0	0
0.019056085	0.155941981	0.048452113	0.003683111
0.0174374	0.299948123	0.032086375	0.050805975
0.053668914	0.089798904	0.006275325	0
0.008714713	0.215957485	0.206734869	0.029459399
0.016635961	0.162354265	0.011197964	0.002331197
0.047987431	0.08566615	0.016527393	0
0.01471757	0.231030084	0	0.005127114
0.087926179	0.274532513	0.002347261	0
0.021821233	0.183058809	0	0.004672955
0.03703529	0.209741798	0.04860328	0.066384151
0.068229548	0.189595396	0.00748277	0.009115947
0.045082878	0.1953997	0	0
0.01034404	0.209779477	0.028106788	0.050436564
0.028047701	0.140997897	0	0.043063518
0.102974123	0.124134558	0	0
0.037574722	0.179899276	0	0.004786138
0.051858484	0.150056828	0.008632333	0
0.018323428	0.133173687	0	0
0.018203483	0.252304833	0.002555296	0.007078162

0.049557088	0.230734559	0	0
0.019906995	0.062412568	0.002670029	0.001932757
0.036133259	0.162323805	0.146759048	0
0.001656358	0.221981773	0.044573249	0.006233333
0.023706395	0.356368902	0.080725156	0.017547917
0	0.055436913	0	0.010090744
0.089320245	0.435935777	0.010619974	0
0.026294902	0.16867322	0	0.076305886

Mast.cells.resting	Mast.cells.activated	Eosinophils	Neutrophils	P.value
0.020056598	0	0	0	0.012
0.105868044	0	0	0.007987361	0.008
0	0.07243534	0	0.008783989	0.044
0.004306901	0	0	0	0.03
0.031566598	0	0	0.00365435	0.022
0.022949724	0	0	0	0.046
0.010674761	0	0	0.006407825	0.022
0.001476485	0.02425092	0	0.003361538	0.002
0.020283582	0	0	0.004874561	0.008
0.031867772	0	0	0	0.012
0.014428835	0	0	0	0.018
0.010923062	0	0	0	0.004
0.046170249	0	0	0	0.016
0.082799579	0	0	0.00635997	0.14
0.02858808	0	0	0	0.008
0.07471443	0	0	0	0.006
0.119681391	0	0	0.01480776	0.018
0.014759659	0	0	0	0.008
0.032718388	0	0	0	0.01
0.047750068	0	0	0.005261513	0.006
0.079010522	0	0	0	0.042
0.177295387	0	0	0	0.02
0	0.027455832	0	0	0.004
0.075802323	0	0	0	0.012
0.097288329	0	0	0.007183364	0.114
0.085970515	0	0	0.002415299	0.136
0.057660297	0	0	0.006160652	0.042
0.026794161	0.011344972	0	0.001341252	0.004
0.030954983	0	0	0	0.002
0.073482704	0	0	0.002905449	0.008
0.039244111	0	0	0.005983266	0.132
0	0.019935767	0	0	0.004
0.061110093	0	0	0.004162136	0.574
0.013536246	0.002696148	0	0	0.052
0.029060922	0	0	0	0.02
0.009619691	0	0	0	0.004
0.041132417	0	0	0.00620309	0.006
0	0.013988862	0	0	0.084
0.025715649	0	0	0.007308428	0.018
0.11915892	0	0	0.001474292	0.086
0	0.106851095	0	0	0.068
0.066791384	0	0	0.002345143	0.002
0.098821477	0	0	0	0.13
0.001122252	0.014637129	0.00102302	0.001161733	0.002
0	0.058041811	0	0.005760505	0.008
0.028370911	0	0	0	0.008
0.068626961	0	0	0	0.02
0.049549397	0	0	0.003821494	0.114
0.044907655	0	0	0.001915625	0.01
0.022743907	0	0	0	0.02
0.044146369	0	0	0.000866784	0.002
0.017613289	0.001648965	0	0.005198283	0.002
0.009789214	0	0	0	0.002

0	0.045984051	0	0.014577877	0.002
0.138186459	0	0	0	0.024
0.172954325	0	0	0	0.098
0.051090244	0	0	0	0.002
0.032381841	0	0	0.003656462	0.002
0.034537161	0.007229987	0.000369895	0.019221561	0.004
0.015556184	0	0	0	0.032
0.011248409	0	0	0	0.094
0.007895777	0.015530372	0	0	0.02
0.00431104	0	0	0	0.002
0	0.017332272	0	0.028419991	0.004
0.000916674	0.037482124	0	0.00716911	0.044
0.114975789	0	0	0	0.004
0.161587538	0	0	0.005254321	0.018
0.05491704	0	0	0.007562823	0.074
0.015263469	0	0	0.012028066	0.01
0.075726501	0	0	0	0.012
0.025282243	0	0	0.021040588	0.052
0.001230609	0	0.001453875	0	0.13
0.090632372	0	0	0	0.002
0.00255684	0	0	0.005815814	0.018
0.012535286	0	0	0	0.004
0	0.016894134	0	0.009957166	0.088
0	0.005997929	0	0.00779011	0.004
0.043489898	0	0	0.003042801	0.02
0.068481227	0	0	0.00592076	0.008
0.008871622	0	0	0.004632627	0.056
0	0.053152453	0	0	0.016
0.142462833	0	0	0.013519712	0.004
0.054142427	0	0	0.005754843	0.022
0.049225103	0	0	0	0.01
0	0.060670213	0	0.022240537	0.002
0.016787802	0	0	0	0.12
0.02097837	0	0	0	0.018
0.052149417	0	0	0.015930081	0.02
0.047882599	0	0	0.00536402	0.004
0.005044709	0	0	0.002446849	0.042
0.014318526	0	0	0	0.004
0.008569099	0	0	0	0.022
0.008758603	0.01407587	0	0.041004342	0.018
0.047771085	0	0	0.00383116	0.036
0.039748047	0	0	0.004073844	0.086
0.028839248	0	0	0.013631812	0.004
0.04326368	0	0	0.02180481	0.042
0.012951353	0	0	0.002715633	0.006
0.076690015	0	0	0.014352776	0.036
0	0.126829556	0	0.026678857	0.076
0.037879383	0.060606383	0	0.020002998	0.132
0.04928986	0	0	0.020385759	0.052
0.050736398	0	0	0	0.114
0.050059756	0	0	0.006902808	0.008
0.055375979	0	0	0	0.064
0.149741235	0	0	0.015244611	0.004
0.04714182	0	0	0.012477193	0

0.006196032	0.000345624	0	0.003527518	0.092
0.026584274	0	0	0	0.01
0.00885073	0.023647444	0	0.012973802	0.01
0.028042602	0	0	0	0.004
0.064793793	0	0	0	0.044
0.056771505	0	0	0.006519566	0.052
0.054290569	0	0	0.001937818	0.004
0.030385669	0	0	0.00094901	0.01
0.053361831	0	0	0.00057459	0.008
0	0.002013308	0	0.001150733	0.01
0	0.022674036	0	0.008856447	0.086
0.116067112	0	0	0	0.008
0.008042976	0.010315632	0	0.015185158	0.028
0.018307748	0	0	0	0.02
0.054251546	0.010599616	0	0.015570584	0.034
0.040169204	0	0	0.017347172	0.012
0.008908889	0	0	0	0.086
0.04963982	0	0	0	0.16
0.125903896	0	0	0.009900022	0.024
0.084181311	0	0	0	0.004
0.091302338	0	0	0.020888212	0.112
0.005688567	0	0	0.000391374	0.018
0.025240964	0	0	0.00885976	0.146
0.104014837	0	0	0.000159192	0.02
0.042955187	0	0	0.010580258	0.002
0.026485415	0.031709658	0.004330723	0.026287082	0.024
0.07922156	0	0	0.005838687	0.014
0.048332766	0.013474086	0	0.009502173	0.032
0.055093867	0	0	0.018747117	0.002
0.028711486	0	0	0	0.02
0.005264376	0.008835595	0	0.007359084	0.002
0.056248617	0	0	0	0.018
0.063560688	0	0	0.033995248	0.006
0.107682322	0	0	0.005391804	0.006
0.057670914	0	0	0.006326769	0.006
0.096442778	0	0	0.00739582	0.044
0.025102139	0	0	0	0.012
0.016462273	0	0	0.005162757	0.004
0.069516544	0	0	0	0.026
0.050340538	0	0	0.014400221	0.008
0.050684261	0	0	0.001082227	0.022
0	0.007262214	0	0.015753247	0.004
0.004393855	0	0	0.009656876	0.02
0.208396849	0	0	0.008625161	0.02
0.147007779	0	0	0	0.044
0	0.054678335	0	0.01164022	0.004
0	0.046090641	0	0.017419757	0.066
0.00276041	0	0	0	0.004
0	0.046497211	0	0.026377534	0.19
0.082782965	0	0	0.001914416	0.01
0	0.03747831	0.003314101	0.031832839	0.01
0	0.052426458	0	0.012187867	0.052
0.090695585	0	0	0.002673263	0.138
0.033259265	0.003071374	0	0.026811648	0.14

0.008737223	0.008690951	0	0	0.044
0	0.026706718	0	0	0.008
0.105833897	0	0	0.013206846	0.07
0.030787509	0	0	0.013915926	0.15
0.119109509	0	0	0.006766582	0.172
0	0.074680619	0	0.079271325	0.004
0.035966501	0	0	0.015188877	0.004
0.01310617	0.012715101	0	0.011090788	0.018
0.035521928	0	0	0.003229457	0.01
0.039344125	0.045349755	0	0.028972818	0.008
0.151554198	0	0	0	0.018
0.107180066	0	0	0.016898671	0.01
0.05617527	0	0	0.022062191	0.086
0	0.043183203	0	0	0.092
0.030681024	0	0	0.016567368	0.01
0.004291327	0.006047374	0	0.011373357	0.144
0.088597591	0	0	0.001723434	0.01
0.003456967	0.026996638	0	0	0.024
0.079177166	0	0	0.009959355	0.014
0.013272093	0.005178369	0	0.007621898	0.002
0.026017938	0	0	0.007894428	0.01
0	0.025652188	0	0	0.006
0.0563428	0	0	0.011032264	0.004
0.079224564	0	0	0.004781611	0.044
0.17132186	0	0	0.008818727	0.012
0.034736059	0	0	0.014156848	0.004
0.046904564	0	0	0	0.01
0.016425516	0	0	0	0.132
0	0.005530145	0	0.008413438	0.008
0.039106413	0	0	0.007326725	0.004
0.084389511	0	0	0.002604658	0.022
0.056507611	0	0	0.000611415	0.004
0.042174356	0	0	0.00083971	0.002
0	0.010510752	0	0.011014772	0.002
0.045339523	0	0.019075794	0	0.054
0	0.051700681	0	0	0.014
0.076204675	0	0	0	0.002
0.048654348	0	0	0.002589456	0.04
0	0.055714159	0	0.010389556	0.02
0.030365336	0	0	0	0.006
0	0.015413301	0	0.002614458	0.078
0.064350476	0	0	0.002737661	0.002
0.076171394	0	0	0	0.012
0.038012051	0	0	0	0.02
0.049274341	0	0	0	0.024
0.057998906	0	0	0.004290754	0.084
0	0	0	0	0.006
0.070526814	0	0	0	0.004
0.057002761	0	0	0.005140403	0.02
0.034140146	0	0	0	0.004
0.019338544	0	0	0	0.014
0	0.057435133	0	0.007172625	0.014
0.025961426	0	0	0.004304216	0.004
0.065723739	0	0	0	0.048

0.056628294	0	0	0	0.006
0.062430294	0	0	0	0.13
0.009285169	0	0	0.009152283	0.014
0.015637237	0.012127325	0	0.007106968	0.062
0.033912231	0	0	0	0.044
0.057296453	0	0	0.010427513	0.02
0	0.070551553	0	0.000179892	0.39
0.027522902	0	0	0	0.112
0.017243738	0	0	0	0.018
0.06814749	0	0	0.006694442	0.038
0.103888866	0	0	0.0028394	0.032
0.018708417	0	0	0.011274465	0.018
0.020624794	0	0	0.000103275	0.002
0.02744673	0.016073885	0	0.008072233	0.14
0.094314204	0	0	0.007049687	0.002
0.035447887	0	0	0.001805326	0.01
0.014478396	0	0	0.00584323	0.15
0	0.049234781	0	0.018822351	0.014
0	0.020087484	0.008576844	0	0.002
0.047799281	0	0	0.030414077	0.052
0.006661367	0.008110994	0	0	0.002
0.106482404	0	0	0.005798423	0.012
0.109702579	0.001937673	0	0.018761407	0.024
0	0.108663275	0	0.028566962	0.008
0.073349998	0	0	0.007071483	0.032
0.061860213	0	0	0.028392572	0.056
0.042338545	0	0	0.021053393	0.018
0.018578845	0	0	0	0.004
0.045923383	0	0	0.002021623	0.004
0.020103994	0	0	0.002402973	0.006
0.045812347	0	0	0.012451192	0.004
0	0.000234957	0.001934579	0.006138119	0.11
0.016437886	0	0	0.012873036	0.01
0	0.079157368	0.003211667	0.004425072	0.004
0.016755185	0	0	0.009888996	0.142
0.014600629	0.011390899	0	0.0029362	0.02
0.031109921	0	0	0.011202345	0.004
0.023798661	0	0	0.004386632	0.004
0.052268414	0.007100097	0	0.003644233	0.022
0.055119117	0	0	0	0.008
0.052713716	0	0	0	0.002
0	0.01042546	0	0	0.018
0.031649802	0	0	0	0.03
0.042938312	0	0	0.002134873	0.002
0.013666903	0	0	0	0.018
0	0.009026202	9.92E-05	0.00983779	0.01
0.061536049	0	0	0	0.098
0.039380072	0	0	0	0.136
0	0	0	0.001162982	0.004
0	0.027619181	0	0.006655265	0.022
0.079265514	0	0	0	0.008
0	0.031697618	0	0.009359107	0.102
0.088114068	0	0	0.000925464	0.058
0.076997391	0	0	0	0.01

0.13452707	0	0	0	0.022
0.073673111	0	0	0	0.034
0.030932724	0	0	0	0.042
0.049882505	0	0	0.002767162	0.03
0.041723441	0	0	0	0.006
0	0.035874304	0.001528303	0.026882511	0.044
0.018851216	0	0	0.003431324	0.002
0.049068969	0	0	0.006252784	0.104
0.074499837	0	0	0.022270435	0.24
0	0.047443338	0	0.015384567	0.08
0.051877497	0	0	0.002458647	0.004
0.071516441	0	0	0.019732934	0.006
0	0.02757382	0	0.01120423	0.02
0.040669154	0	0	0.013892366	0.018
0.052652103	0	0	0	0.144
0.03740048	0	0	0	0.004
0.023074566	0	0	0.003574244	0.002
0.012472776	0	0	0	0.254
0.017324224	0	0	0.018485611	0.082
0.025116998	0	0	0	0.004
0.025247421	0	0	0.00528487	0.05
0	0.008858748	0	0.039019387	0.044
0.116440799	0	0	0.002587554	0.042
0.026935724	0	0	0	0.018
0.05608168	0	0	0.011279504	0.106
0.093211554	0	0	0.015781519	0.114
0	0	0	0	0.042
0.093170449	0	0.013083125	0.022509842	0.136
0.052141237	0	0	0.02055819	0.188
0.099950513	0	0	0.007469763	0.004
0.052657933	0	0	0.006621459	0.03
0.013545605	0.001313702	0.005732024	0.040572422	0.01
0	0.019231084	0	0	0.028
0.06925037	0	0	0.002373318	0.192
0	0.029827137	0	0.010769144	0.142
0.05902442	0	0	0	0.022
0.045310393	0.042330413	0	0.006937915	0.044
0.045541549	0	0	0	0.04
0.049817006	0	0	0.008743376	0.012
0.073909248	0	0	0.008155591	0.008
0.092957863	0	0	0	0.126
0	0.01777207	0	0	0.064
0	0.04468463	0	0.007397907	0.002
0	0.038945196	0	0.007720241	0.11
0	0.032397955	0	0.024009758	0.048
0	0.172767154	0	0.034928725	0.17
0	0.025040357	0	0.004704762	0.024
0	0.000109038	0	0	0.002
0.052028668	0	0	0	0.004
0.023279913	0	0	0.010925048	0.042
0.021805106	0	0	0.000724425	0.02
0.054196774	0	0	0	0.004
0.078012653	0	0	0.001059816	0.194
0.068434203	0	0	0.020156134	0.018

0.02325694	0.032747955	0.010090111	0.00889318	0.042
0	0.088214167	0	0.035775031	0.022
0.008909344	0.001294952	0	0	0.002
0.042915294	0	0	0.003487134	0.002
0.032832366	0.021595954	0	0	0.022
0.015070082	0.02044541	0	0.001562994	0.02
0.084359479	0	0	0.016276215	0.008
0.033610566	0	0	0.005042915	0.02
0.093473787	0	0	0	0.018
0.085699588	0	0	0.011562855	0.012
0	0.060840499	0	0.007296787	0.042
0.106141186	0	0	0.002104323	0.02
0.021875943	0	0	0	0.004
0.039469691	0.001982384	0	0.025743096	0.02
0.018452273	9.36E-05	0	0.04833915	0.002
0.039415792	0	0	0.005498398	0.004
0.040326013	0	0	0	0.056
0.080802655	0	0	0.023882755	0.092
0.030162296	0	0	0.012940552	0.1
0	0.017965977	0	0.024093999	0.002
0.085074021	0	0	0	0.004
0.055138186	0	0	0.023683365	0.01
0	0.000457759	0	0.002973481	0.004
0	0.008991647	0	0.002434055	0.004
0	0.03042463	0	0.019847581	0.01
0.02858075	0	0	0.005217401	0.008
0.011236579	0	0	0	0.004
0.010896944	0	0	0.003982371	0.046
0.022860888	0	0	0	0.002
0.037679767	0	0	0	0.004
0.082934171	0	0	0	0.02
0.101685896	0.001105093	0	0.026377148	0.01
0.011116351	0.017912484	0.001685323	0.016905212	0.024
0.094085431	0	0	0.021005227	0.042
0.094346971	0	0	0	0.004
0.052837676	0	0	0.047088742	0.012
0.020452436	0.003038192	0.000729304	0.018526234	0.008
0.06737679	0	0	0.011107421	0.012
0.055548261	0	0	0	0.004
0.065073653	0	0	0	0.024
0.052793222	0	0	0.013174387	0.002
0.059355849	0	0	0	0.01
0.081429272	0	0	0.017161262	0.098
0.027104621	0	0	0	0.008
0.09474366	0	0	0.004009819	0.01
0.078076071	0	0	0.005993293	0.066
0.025751328	0	0	0.002079039	0.136
0	0.003847844	0	0.011449688	0.004
0.122293306	0	0	0.004890044	0.008
0.016983822	0.015298272	0	0.028122336	0.008
0.082502079	0	0	0.015774137	0.144
0.046266366	0	0	0.008013034	0.088
0	0.044019406	0	0	0.144
0.00518002	0	0	0.021459934	0.004

0.095772071	0	0	0.011849173	0.014
0.084973926	0	0	0.016624828	0.018
0.061334281	0	0	0.009336483	0.004
0.057491541	0	0	0.031481875	0.008
0.020736842	0	0	0.014643314	0.216
0.070367128	0	0.001750668	0.01567566	0.022
0	0.073271199	0.047029361	0	0.018
0.011294693	0	0.000244471	0.004756103	0.004
0.110643405	0	0	0.004759175	0.002
0	0.064015442	0	0.007006376	0.012
0.106344699	0	0	0.009205096	0.04
0.088584084	0	0	0	0.046
0.090811516	0	0	0.023138894	0.008
0.082626414	0	0	0.010018384	0.008
0	0.003416007	0.001708991	0.004812176	0.01
0.047762287	0	0	0.004997392	0.01
0.047626724	0	0	0.006402998	0.016
0.061782336	0	0	0.020559201	0.16
0.046179531	0	0	0.002540509	0.004
0.060223889	0	0	0	0.004
0.069234788	0	0	0	0.006
0.040801577	0	0	0.005049088	0.002
0	0.034458326	0	0.010815245	0.018
0.033245656	0	0	0	0.03
0.134453044	0	0	0	0.01
0.066268126	0	0	0.002764867	0.014
0	0.009168954	0	0	0.006
0.06982093	0	0	0.000546645	0.002
0.033786871	0	0	0.000627599	0.004
0.022863479	0	0	0	0.142
0.042222486	0	0	0.002415452	0.08
0.014193418	0.013150958	0	0.007066094	0.016
0.059329868	0	0	0.028892969	0.008
0.174511451	0	0	0	0.028
0.030289195	0	0	0	0.016
0	0.056197054	0	0.004031948	0.054
0.004178714	0.036953811	0	0.070757157	0.02
0.087026058	0	0	0.01818994	0.01
0.080114909	0	0	0.00851999	0.092
0.03055555	0	0	0	0.014
0	0.041917399	0	0.010939357	0.134
0.030437199	0	0	0	0.042
0.030304302	0	0	0.002759358	0.004
0.047246674	0	0	0	0.06
0	7.12E-05	0	0	0.01
0.115119543	0	0	0.012647205	0.1
0.088549256	0	0	0.004635877	0.092
0.022218945	0	0	0.021700565	0.002
0.033188976	0	0	0.012311907	0.018
0.012222739	0.003298086	0	0	0.042
0	0.038617607	0	0.02348199	0.042
0	0.073228355	0	0.024277531	0.042
0	0.024763612	0	0.038233491	0.004
0	0.012586016	0	0.012783843	0.016

0.012745475	0	0	0.0118471	0.02
0.075141397	0	0	0	0.01
0.034240957	0	0	0	0.01
0.023413854	0	0	0.011261532	0.004
0.024160025	0	0	0	0.098
0.050017316	0	0	0.014813548	0.02
0.028903766	0	0	0.009058961	0.024
0.029309304	0.006863119	0	0	0.002
0.043622601	0	0	0	0.02
0	0.062755202	0	0.025779035	0.126
0.045652324	0	0	0.012192255	0.002
0.024680176	0	0	0	0.004
0.02634905	0.018290938	0	0.001652588	0.004
0	0.009481565	0	0.004892863	0.02
0.035576249	0	0	0	0.004
0.019419699	0	0	0.010676548	0.048
0.052086329	0	0	0.027311247	0.024
0	0.019934156	0	0	0.1
0.035950174	0	0	0.010475838	0.01
0.026118588	0	0	0	0.012
0.007013096	0.006635283	0.005169874	0.008399893	0.004
0.038270101	0	0	0.002801728	0.008
0.04740046	0	0	0	0.004
0.037550069	0	0	0.004443115	0.02
0.034670885	0	0	0.005784811	0.068
0.075927508	0	0	0.003712268	0.004
0.178342172	0	0	0.009040403	0.03
0.036538867	0.042049852	0	0.069492692	0.022
0.073847744	0	0	0.012501904	0.142
0.098365842	0	0	0.029957121	0.114
0.128433866	0	0	0.015957593	0.024
0.061359184	0	0	0.025670849	0.01
0.122619686	0	0	0	0.03
0	0.182960923	0	0.059311156	0.044
0.043761489	0.010098345	0	0	0.02
0.023588472	0	0	0.002735883	0.002
0.146647149	0	0.013723223	0.009559106	0.028
0.102605569	0	0	0.031086538	0.012
0.012125122	0.034913036	0	0.012295562	0.012
0.08002193	0.033164102	0	0.083905602	0.044
0.06648119	0.023126429	0	0.029616271	0.08
0	0.187349129	0	0	0.044
0.133358913	0	0	0.001963408	0.01
0.01575014	0.015114387	0	0.010875395	0.022
0.134971774	0	0	0.01184667	0.012
0.108949742	0	0	0.007858352	0.004
0.063595496	0	0	0.003901996	0.024
0	0	0	0.003411165	0.002
0.04973977	0	0	0.015963489	0.004
0	0.047802624	0	0	0.002
0	0.030499472	0	0.071017347	0.002
0.024169893	0	0	0.020259826	0.02
0.022231071	0	0	0	0.048
0.126126954	0.020713899	0	0.024225517	0.022

0.118265726	0	0	0	0
0.058076711	0	0	0	0.01
0	0.026771928	0	0.02280335	0.008
0.060508143	0	0	0.002455574	0.14
0.066357821	0	0	0.005776191	0.004
0.069550617	0	0	0.005367888	0.042
0.024872042	0	0	0	0.002
0.07919722	0	0	0	0.018
0.105165339	0	0	0.001474338	0.01
0.039290124	0	0	0.003704376	0.02
0.043477267	0	0	0	0.004
0.016268102	0.003842143	0	0	0.01
0	0	0	0	0.002
0.000208922	0.001297597	0	0.022951105	0.022
0.184132556	0	0.011380466	0.00096282	0.012
0.018394235	0	0	0	0.024
0.074823111	0	0	0.008985917	0.022
0.037276345	0	0	0	0.004
0.025677669	0	0	0.000341297	0.08
0.037515842	0	0	0.020015949	0.086
0.024558486	0.004148764	0	0	0.004
0.037055087	0	0	0	0.136
0	0.008234812	0	0.011124095	0.024
0.10000412	0	0.007766716	0.012231521	0.066
0.049913295	0	0	0.014974076	0.02
0.048331538	0	0	0	0.042
0.144819961	0	0	0.011297756	0.128
0.116526084	0	0	0	0.01
0	0.05289324	0	0	0.126
0	0.066567961	0	0	0.066
0.087149234	0	0	0	0.074
0	0.020445635	0	0.003320362	0.088
0.002372366	8.81E-05	0	0	0.008
0.016254387	0.000553696	0	0	0.008
0	0.014578836	0	0.001581578	0.01
0.076642744	0	0	0	0.008
0.139569277	0	0	0.018058703	0.008
0.08164544	0	0	0	0.028
0.075008993	0	0	0	0.01
0.048329377	0	0	0.000715497	0.146
0.060509904	0	0	0.005816207	0.002
0.052251524	0	0	0.003342782	0.002
0.212053912	0	0	0.00350067	0.02
0.052377478	0	0	0	0.002
0.054047739	0	0	0.002165151	0.004
0.008450768	0.015612847	0	0	0.002
0.054688488	0	0	0	0.074
0.110652394	0	0	0	0.004
0.039345031	0	0	0.003325326	0.008
0.016629163	0	0	0.014499577	0.03
0.003287436	0.005558055	0	0.005968258	0.018
0.111556231	0	0	0	0.018
0.089617204	0	0	0	0.02
0.040802204	0	0	0	0.004

0	0.007743844	0.002781446	0.028844223	0.002
0.032074144	0	0	0	0.002
0.002638706	0	0	0	0.016
0	0.043213285	0	0.005004686	0.002
0.030564656	0	0	0.007812503	0.006
0.017390773	0	0	0	0.008
0.008775562	0	0	0	0.048
0.065746229	0	0	0	0.002
0.062374701	0	0	0	0.002
0.025952108	0	0	0.006995543	0.006
0.047341991	0	0.02479048	0.024186951	0.022
0.052829194	0	0	0.004495957	0.16
0.062226545	0	0.02419717	0.005473318	0.006
0.074757982	0	0	0.027328612	0.048
0.033476092	0	0	0.005070337	0.018
0	0.07906585	0	0.019070739	0.068
0.206150854	0	0.096432659	0	0.03
0.018617588	0	0	0.01404875	0.01
0.086444162	0	0	0	0.002
0.102299002	0	0	0.014768729	0.01
0.036327587	0	0	0.045763806	0.048
0.048186171	0	0	0.025634666	0.222
0.110659597	0	0	7.46E-05	0.01
0.004717614	0	0	0.03922467	0.008
0.047690262	0	0.020960618	0	0.004
0.29172234	0	0	0	0.03
0.078034641	0	0	0.006556254	0.02
0.010652867	0	0	0.014532825	0.088
0	0	0	0.007898663	0.02
0	0.09801216	0.015422702	0.07137584	0.032
0.050417181	0	0.015102285	0.015895277	0.17
0	0.006035451	0.063762198	0.072835981	0.052
0.081322788	0	0.008381528	0	0.02
0.074241106	0	0	0	0.082
0.0523338	0	0	0	0.018
0.050045078	0	0	0	0.002
0.00741962	0.00525546	0	0.003374108	0.01
0.00676426	0.01666794	0	0.002994159	0.07
0.074162608	0	0	0.009524742	0.012
0.013911876	0.011621742	0	0	0.004
0.083622704	0	0	0	0.004
0.073796334	0	0	0.004606576	0.004
0	0.00396332	0	0	0.044
0.017406996	0	0	0	0.002
0.023977902	0.011296607	0	0.001977512	0.004
0.073942473	0	0.022517803	0	0.004
0	0.006008295	0	0	0.004
0.156920214	0	0	0.000818971	0.018
0	0.013345848	0	0	0.074
0.022309922	0	0	0	0.002
0.026626146	0	0	0	0.01
0.059053776	0	0	0.002486461	0.002
0	0.00338001	0	0	0.004
0.083864755	0	0	0.001534914	0.012

0.029436818	0	0	0.01107512	0.006
0	0.021235312	0.002109672	0.028514478	0.004
0.093863509	0	0	0	0.002
0	0.013050382	0	0.008613956	0.004
0.069219859	0	0	0.004476443	0.044
0	0.047462708	0.01515055	0.031714536	0
0.059353499	0	0	0	0.11
0.131446337	0	0	0.020906046	0.042

Correlation	RMSE
0.251370102	0.989856398
0.288700357	0.962538554
0.158150311	1.020572661
0.182755367	1.016587399
0.201387263	1.002993388
0.154555956	1.044628174
0.192816826	1.007339231
0.421269811	0.90843381
0.29303868	0.97629148
0.254756742	0.98519993
0.223316501	1.000784196
0.345872414	0.945478326
0.241142858	1.001433456
0.088712016	1.046750516
0.29246212	0.963467918
0.318066797	0.952631727
0.235288038	0.990885628
0.300083108	0.963648106
0.268294469	0.974015013
0.312568329	0.956390731
0.170223192	1.011273854
0.21383958	0.994272984
0.360437766	0.935772674
0.256025146	0.975940111
0.099231933	1.040635317
0.090515165	1.039213748
0.166342458	1.013546159
0.335347921	0.94762989
0.467817351	0.891653365
0.280693164	0.965542272
0.091839471	1.049498226
0.386119545	0.925302252
-0.008246169	1.088878198
0.148730807	1.037494532
0.20311559	1.003507883
0.36925212	0.933902796
0.307399343	0.963280303
0.121428048	1.038320263
0.226320684	1.007543767
0.118999808	1.03398171
0.136393268	1.039314985
0.425898117	0.905133826
0.093317006	1.043917111
0.459611372	0.889177304
0.305477913	0.959193679
0.288325986	0.97056888
0.205129402	0.997177911
0.099459321	1.049393617
0.278669836	0.96788375
0.219429661	0.999441746
0.482899094	0.886107356
0.434214267	0.902653614
0.460183799	0.94538943

0.460710776	0.89086305
0.191944868	0.997784378
0.108392511	1.0313923
0.419315283	0.910427955
0.439508356	0.899629114
0.396643113	0.918252107
0.177708335	1.01232104
0.111126305	1.044153833
0.204042547	0.999049608
0.431008161	0.905910489
0.332503112	0.958690563
0.160212157	1.026959856
0.351751307	0.937871308
0.229873268	0.989065716
0.129874943	1.02558088
0.277912564	0.986086129
0.255092227	0.977856906
0.14690314	1.038961367
0.093346447	1.050717013
0.418914498	0.910398834
0.227017689	0.997706655
0.356537278	0.9455172
0.118137219	1.040230578
0.358999851	0.936108959
0.219794788	0.997905836
0.299447573	0.960642327
0.144071101	1.029538919
0.242226395	0.992028811
0.33805232	0.944087226
0.201434185	0.999020813
0.279183258	0.990946652
0.41630293	0.926012821
0.097667612	1.055025753
0.236550588	1.001334753
0.220346395	0.993744943
0.339784422	0.949213024
0.16513611	1.021944538
0.388427976	0.922550507
0.195585875	1.029696263
0.23016023	0.988176356
0.174540642	1.026518785
0.120510667	1.03480869
0.353174704	0.939617368
0.168098039	1.015635433
0.310751511	0.961769646
0.174666162	1.008650113
0.128995149	1.035922777
0.092077932	1.057362842
0.146467218	1.036953252
0.099967297	1.045160848
0.304480726	0.957339909
0.138807733	1.023458188
0.378940258	0.926183038
0.507282852	0.863013458

0.112084173	1.04390505
0.265000475	0.982248932
0.271354515	0.973667028
0.346829879	0.945441337
0.16175603	1.021918626
0.147696875	1.034471912
0.405046472	0.916560263
0.266734954	0.985942734
0.294289266	0.962820643
0.265269985	0.982602781
0.120705565	1.03620622
0.290342251	0.963520875
0.185554507	1.01268011
0.209624177	1.002870077
0.175748105	1.008093194
0.256890365	0.979871637
0.120537318	1.044019188
0.070499264	1.077378472
0.192167487	1.011886681
0.366105716	0.933351876
0.102378814	1.050122821
0.235216822	0.995568283
0.076408059	1.06054853
0.219124003	0.994464628
0.413372236	0.91002207
0.191472474	1.00511428
0.247658155	0.979752835
0.177033509	1.007572303
0.461702791	0.899645859
0.215313939	1.001206639
0.429008373	0.906338287
0.235041949	0.992016822
0.321733152	0.957837813
0.323057446	0.95041034
0.306934375	0.956976793
0.156329651	1.031134473
0.248945236	0.98783019
0.341790181	0.947298291
0.186261432	1.008370523
0.280432231	0.966468718
0.194326381	1.016788094
0.37613506	0.935542929
0.208424429	0.998754475
0.205242518	0.999657255
0.156503395	1.016434853
0.400945569	0.916757157
0.136931318	1.028571675
0.334883554	0.957811894
0.056101158	1.063512051
0.263057833	0.972885746
0.274691332	0.969495683
0.148099625	1.03379549
0.089138085	1.047939561
0.088482404	1.062773094

0.159731628	1.036413394
0.302728992	0.963074034
0.133578425	1.030598607
0.072864977	1.064728147
0.065192484	1.059834213
0.396895994	0.920483206
0.374022845	0.931674698
0.229793921	0.992318436
0.269463992	0.978600436
0.281838034	0.967106558
0.224663726	0.988028078
0.266832924	0.982856264
0.119907703	1.027758784
0.111857554	1.05001485
0.27391851	0.973805594
0.080002285	1.065288659
0.275188977	0.969058188
0.189097468	1.016807859
0.244409482	0.981535647
0.475631481	0.878837341
0.27547933	0.975142292
0.311252172	0.95634793
0.394218921	0.921743881
0.161776971	1.022183592
0.25078793	0.979996022
0.336916688	0.944851245
0.262205565	0.97549351
0.092032954	1.096634578
0.284741344	0.967447156
0.356322954	0.943050709
0.198417456	0.998524456
0.379725362	0.927332025
0.442614848	0.899226625
0.424752376	0.909893536
0.145908548	1.025169575
0.247112558	0.984109985
0.426567884	0.904721948
0.173244238	1.019193166
0.22119118	0.998454925
0.309667409	0.954319845
0.127158628	1.033268232
0.409592112	0.913725782
0.256736858	0.979869258
0.203321613	1.003324097
0.188077033	1.005386114
0.122467299	1.032920653
0.32048042	0.960580034
0.330366338	0.947612167
0.204045559	1.005127365
0.372163106	0.931658078
0.248698847	1.000418706
0.243329413	0.990319519
0.376365314	0.929757029
0.154006017	1.016765703

0.322351213	0.955396502
0.09344003	1.052423239
0.244913269	0.989202728
0.140688648	1.027653191
0.159428881	1.024166446
0.211696951	0.995607782
0.015031673	1.125361906
0.102595256	1.076293948
0.238740329	0.992803089
0.174173205	1.011448319
0.176315552	1.010668028
0.227817791	0.994177092
0.420715956	0.910790088
0.088134812	1.076511824
0.449618043	0.893050998
0.276159063	0.970337325
0.072965555	1.053972783
0.243209016	0.981661697
0.454886357	0.916122479
0.147410381	1.022481625
0.460160928	0.896148712
0.254298172	0.976816701
0.189412215	1.006393901
0.285726207	0.978751772
0.176042749	1.010801521
0.143780997	1.023185343
0.233878469	0.991977066
0.336522497	0.947078186
0.324484268	0.950265973
0.311675627	0.958859327
0.398590863	0.919351665
0.104050052	1.059807936
0.259687093	0.983124832
0.371056075	0.932341223
0.082364144	1.052196726
0.204849677	1.00145008
0.407424035	0.918867841
0.331341601	0.949047471
0.192334722	1.00539071
0.304095571	0.959771211
0.416266708	0.917699726
0.223736806	1.011367041
0.181046878	1.012518113
0.434371084	0.904759835
0.238424706	0.997967208
0.267789247	0.9763128
0.109546476	1.059391331
0.090718871	1.048803848
0.349303588	0.943033493
0.199331859	1.011870316
0.299368518	0.959828182
0.105813635	1.050898506
0.143198961	1.025402905
0.275393758	0.968812199

0.202046313	1.001898207
0.17565689	1.016755484
0.162439041	1.014605236
0.182907299	1.00856812
0.322631271	0.953821598
0.161260748	1.019061676
0.462347065	0.891010911
0.105285794	1.037248624
0.043297385	1.075501905
0.125437357	1.033163987
0.336559711	0.944754992
0.305961196	0.956634112
0.220090014	1.00064137
0.235253709	0.989269879
0.07916699	1.051717983
0.368888295	0.934315561
0.443469118	0.908429909
0.040149082	1.082311235
0.123561517	1.035478089
0.393081786	0.919997148
0.151988629	1.021242876
0.160704874	1.030258331
0.168435828	1.00775777
0.222832372	0.988657042
0.10510171	1.065049445
0.100137502	1.071977572
0.163824879	1.021387521
0.091167041	1.04064259
0.057835492	1.068722017
0.369920865	0.930867782
0.179496786	1.025202695
0.278336045	0.97832326
0.184215215	1.010247166
0.05431335	1.064004427
0.083761696	1.062084632
0.201674716	0.997203073
0.160206102	1.01344315
0.172582062	1.018270697
0.256537182	0.980112203
0.301453663	0.963803122
0.095763774	1.041721101
0.139603774	1.034273662
0.450706652	0.895395919
0.104323247	1.04783208
0.153195828	1.02027834
0.065611792	1.070076809
0.187714382	1.008466363
0.422683859	0.90900478
0.362649521	0.933424119
0.166329459	1.017197653
0.209303314	1.000123747
0.361207057	0.932771566
0.05310996	1.056306862
0.22788766	1.002132825

0.163869697	1.02502511
0.198776477	1.002781034
0.471339667	0.895467769
0.499007922	0.877476311
0.200835241	1.00331865
0.217807822	0.999259773
0.288642602	0.971012729
0.204745254	0.997757036
0.227921295	0.987714572
0.257823576	0.976990263
0.167970637	1.019563869
0.214393172	0.991552009
0.382984605	0.92833818
0.205583206	0.996565164
0.444573923	0.900330368
0.399080635	0.92128453
0.144568449	1.033289587
0.111969205	1.047854067
0.107260342	1.045294032
0.482478309	0.888532356
0.330657712	0.953151168
0.266902302	0.975543847
0.359605426	0.944910378
0.328290725	0.950611358
0.262016435	0.985871664
0.296160659	0.965580992
0.381230221	0.927688887
0.155346864	1.021598725
0.444913139	0.897360799
0.326784048	0.951407525
0.20437918	0.998942309
0.275777618	0.96717771
0.187674866	1.01003548
0.164370209	1.013612167
0.35955028	0.934391065
0.25249434	0.981186578
0.291335872	0.9663727
0.251664636	0.987847986
0.372504154	0.931110654
0.189764716	1.009124764
0.412619785	0.913114738
0.269526717	0.970352652
0.108780731	1.038666507
0.282998246	0.967173963
0.277713044	0.965841353
0.137117229	1.02161165
0.091164372	1.05781885
0.335996039	0.948681162
0.284615641	0.967880361
0.294828958	0.965802374
0.078153038	1.049602486
0.116702896	1.039386525
0.077214229	1.106494436
0.355041033	0.941305942

0.246588556	0.979273636
0.227571626	0.988146297
0.357539984	0.936048741
0.285653263	0.968964913
0.048965272	1.068250405
0.197348586	1.001239225
0.234740306	0.995274907
0.386665316	0.929156733
0.43616818	0.90171144
0.254874282	0.979748532
0.172826925	1.009724985
0.155082091	1.02198343
0.285950902	0.969007651
0.280050921	0.967737332
0.271762048	0.977396184
0.264537526	0.978787587
0.240348318	0.987187691
0.070664239	1.056891876
0.372225043	0.929565575
0.339727706	0.947337463
0.313786168	0.954432056
0.490129226	0.890429893
0.22875661	0.987775653
0.183526695	1.02387312
0.278857225	0.969566142
0.246588135	0.979014457
0.322793334	0.953426853
0.447370634	0.899001775
0.367755407	0.932869561
0.081979541	1.071159467
0.126763683	1.046281176
0.239569886	0.986805849
0.300459969	0.96997697
0.186083337	1.000905556
0.23954177	0.984989193
0.145648347	1.031427987
0.216473996	0.998793466
0.262548902	0.997453087
0.112785516	1.037431288
0.244088364	0.991494296
0.09135573	1.051196658
0.162650619	1.034274085
0.392500554	0.922772615
0.1414671	1.036933239
0.271340104	0.978213236
0.10750297	1.048181434
0.112414467	1.040496298
0.448841828	0.90150851
0.230523517	0.992058189
0.170559563	1.040297199
0.166630396	1.04228776
0.170946715	1.01817713
0.371089877	0.930649758
0.241791759	0.989865165

0.206660398	1.000680369
0.260068883	0.975255259
0.262596092	0.98551206
0.348566584	0.943572834
0.108111772	1.054664148
0.205476972	1.001319228
0.188127835	1.010107291
0.449836921	0.894725272
0.215416798	0.995355251
0.095601539	1.049089761
0.41943251	0.908362233
0.398457337	0.917090666
0.341904118	0.944821446
0.206689254	1.006828318
0.331043078	0.956033563
0.153675555	1.027738118
0.188003948	1.026170054
0.106316671	1.061206856
0.271906413	0.971385053
0.255264578	0.983689557
0.328823289	0.968923075
0.301681363	0.958254517
0.375028585	0.928393854
0.208790808	0.999582994
0.135957787	1.029101311
0.337871703	0.944184741
0.18160759	1.008280208
0.192389043	1.001715737
0.0850837	1.044748722
0.101241788	1.039150465
0.187608523	1.0029958
0.258232636	0.989449913
0.181037721	1.007649879
0.158993877	1.024540163
0.219458341	0.996446223
0.462502699	0.904536033
0.185291211	1.003567594
0.257156496	0.974548352
0.25747464	0.976274857
0.15928988	1.015998367
0.125317561	1.035517307
0.157658432	1.032150244
0.263228653	0.97569767
0.196853778	1.009120289
0.252878386	0.986941057
0.375138102	0.927160457
0.188725304	1.025816072
0.439616097	0.908124532
0.384964895	0.924584407
0.450197186	0.896683899
0.46524062	0.890377617
0.217307791	1.000070941
0.153022202	1.019857106
0.195325629	1.019526228

0.540786365	0.840518144
0.267123118	0.975582419
0.288803833	0.967809715
0.087077537	1.050375142
0.364619995	0.933999397
0.162684267	1.020294539
0.454508646	0.894380315
0.221812762	0.994276937
0.269856103	0.969438801
0.219044651	0.994325796
0.360277491	0.936328681
0.278274351	0.973874052
0.425143149	0.921239701
0.192941555	1.009302661
0.248735365	0.978243025
0.187470929	1.009467304
0.193374102	1.004132949
0.370898183	0.932139797
0.125550334	1.033223466
0.11885411	1.036342807
0.333399918	0.94838833
0.090829335	1.050065803
0.188772273	1.008561467
0.136404326	1.028138144
0.217926991	0.994469052
0.170001987	1.015782788
0.094073746	1.038227889
0.265236238	0.971940826
0.095784533	1.046593017
0.137499773	1.037238553
0.129406702	1.02950991
0.117263135	1.04106809
0.302247176	0.965878086
0.299252192	0.962898301
0.271832502	0.979392855
0.287677313	0.961685036
0.28445142	0.966561774
0.185354301	1.003629695
0.271578853	0.972694067
0.076356544	1.056475537
0.435898241	0.899957769
0.426577292	0.90478124
0.215497225	0.996392134
0.40803385	0.921183593
0.385325982	0.923384727
0.424755523	0.907364893
0.130143612	1.0350122
0.334935224	0.944839526
0.290187529	0.966962793
0.180693028	1.019197093
0.235875521	0.995325898
0.232771042	0.99287634
0.208509282	1.004390989
0.346283826	0.94124856

0.442947358	0.910203711
0.42151369	0.907773437
0.240714705	0.98993905
0.408919363	0.915975245
0.319795402	0.956217336
0.303918316	0.965321053
0.154383611	1.026068189
0.459720299	0.888847789
0.426457497	0.905626401
0.308882159	0.959817644
0.198724764	0.997122735
0.07060284	1.055816554
0.316515464	0.951717108
0.15367539	1.017753233
0.224207168	0.99422626
0.135312851	1.033994366
0.179351859	1.004016893
0.273878113	0.978038507
0.441337784	0.898445247
0.258746905	0.975305565
0.153711752	1.033355174
0.046230216	1.066750065
0.258291581	0.971597239
0.289222979	0.963417609
0.355548418	0.938081621
0.180483145	1.016459502
0.214943476	0.993018422
0.116031288	1.047115198
0.214223397	1.002269079
0.176140169	1.016255881
0.06593228	1.070396348
0.147915143	1.022451388
0.217131416	0.996420117
0.123212478	1.03826743
0.235675144	1.004280687
0.422757133	0.907418037
0.270954081	0.972310597
0.133971565	1.062722217
0.254058366	0.974757328
0.333350612	0.949189256
0.397577192	0.920482304
0.364011012	0.933072418
0.160820519	1.025318241
0.468678255	0.88775789
0.337175945	0.94334289
0.358412788	0.936789261
0.335474896	0.950333696
0.222788037	0.989459746
0.129268885	1.056261027
0.409305782	0.913988661
0.264776127	0.977638093
0.490850152	0.874794607
0.37944965	0.934872797
0.25344187	0.979343801

0.312983376	0.958224193
0.382956651	0.935139243
0.441612721	0.896473936
0.346844692	0.944536532
0.159015692	1.015845894
0.527484745	0.872954699
0.104103517	1.050603836
0.171040435	1.010462655
