# Supplementary material for: Identification of Prognostic DNA Methylation Signatures in Lung Adenocarcinoma
Source: Oxid Med Cell Longev. 2022 Jun 29;2022:8802303. doi: 10.1155/2022/8802303 (PMC9259289; doi:10.1155/2022/8802303)
Supplement: Supplementary Materials — Supplement Fig1: consistent clustering of tumor DNA methylation-related gene expression profiles. A-B: the optimal number of clusters is determined, and the CDF delta area curve is observed; C: when the cluster is selected as 2 and it has relatively stable clustering results; D: the prognosis of DNA methylation-2 was significantly better than that of DNA methylation-1. Supplement Fig2: A: the samples had a good aggregation form in the space of the first and second dimensions; B: volcanic map of differential expression analysis between tumor DNA methylation subtypes. Supplement Fig3: consistent clustering of differentially expressed gene expression profiles among tumor DNA methylation subtypes. A-B: the optimal number of clusters is determined, and the CDF delta area curve is observed; C: when the cluster is selected as 2 and it has relatively stable clustering results; D: the prognosis of C1 was significantly better than that of C2. Supplement Fig4: the best gradient grouping of the tumor DNA methylation score (DMS). A: the score value of 4.75 was selected as the critical point; B: the group with low DMS had a good prognosis. Supplement Fig5: A: the relationship of the DNA methylation regulator pattern, ACRG molecular subtype, gene cluster, and DMS group is summarized in the Sankey diagram. B: the results showed that the meth.cluster with good prognostic correlation cluster 3 had a trend of lower DMS. C: dynamic flow diagram of tumor sample grouping and state transition. Supplement Fig6: consistent clustering of gene methylation profiles in tumors. A-B: the optimal number of clusters is determined, and the CDF delta area curve is observed; C: when the cluster is selected as 3 and it has relatively stable clustering results; D: the prognosis of C3 was significantly better than that of method.cluster-1/2. [file 8802303.f1.zip › Table S2.pdf]

Gene	logFC	AveExpr	t	P.Value	adj.P.Val
KIF18B	-2.233752591	3.141406138	-23.90843835	2.15E-84	6.26E-80
TICRR	-1.928860853	2.029407429	-22.90125344	1.53E-79	2.23E-75
SPAG5	-1.718356158	4.591895164	-22.34484971	7.37E-77	7.17E-73
TPX2	-2.095353963	5.25185806	-21.82471424	2.39E-74	1.74E-70
MYBL2	-2.430464022	4.98365889	-21.80147881	3.09E-74	1.80E-70
RAD54L	-1.871653185	2.188530982	-21.65504074	1.57E-73	7.64E-70
UHRF1	-1.7981447	3.546828358	-21.62687603	2.15E-73	8.96E-70
GTSE1	-1.814513834	3.030145131	-21.58338531	3.48E-73	1.27E-69
ESPL1	-2.012957852	3.387151407	-21.55662075	4.69E-73	1.52E-69
CDCA5	-1.866424497	4.066207171	-21.36556907	3.91E-72	1.14E-68
EME1	-1.657617896	1.751066748	-21.16098061	3.79E-71	1.01E-67
CENPF	-1.902627016	5.205589875	-21.12188765	5.86E-71	1.42E-67
KIF4A	-2.028712894	3.781026007	-21.07212662	1.02E-70	2.28E-67
KIF2C	-1.93996644	4.04472289	-20.97324255	3.05E-70	6.35E-67
CDC6	-1.879145346	3.978726084	-20.84306045	1.29E-69	2.51E-66
HJURP	-2.034281434	3.421153158	-20.5751053	2.52E-68	4.60E-65
FOXM1	-1.973699757	4.588172895	-20.5403382	3.71E-68	6.36E-65
KIF14	-1.95871098	2.757961552	-20.41887269	1.42E-67	2.31E-64
KIFC1	-1.781337754	4.243508868	-20.36553022	2.57E-67	3.95E-64
TROAP	-2.073296012	3.219580999	-20.35407051	2.92E-67	4.26E-64
POLQ	-1.884424515	2.108543877	-20.27667913	6.88E-67	9.56E-64
KIF23	-1.755015477	3.775312549	-20.21301406	1.39E-66	1.85E-63
TOP2A	-1.877065331	6.420791053	-20.19741744	1.65E-66	2.10E-63
NCAPH	-1.803755626	3.354842485	-19.94277031	2.77E-65	3.36E-62
ASPM	-1.910457404	3.870942366	-19.82030545	1.07E-64	1.25E-61
CDC45	-1.834031402	2.934428887	-19.79598663	1.40E-64	1.57E-61
MCM10	-2.046631709	2.622327467	-19.76632021	1.94E-64	2.10E-61
MKI67	-1.83202296	5.758764342	-19.73829954	2.65E-64	2.76E-61
EXO1	-1.918267768	2.705642726	-19.73142064	2.86E-64	2.87E-61
FEN1	-1.126758447	4.885057761	-19.7267948	3.01E-64	2.92E-61
XRCC2	-1.653909681	2.333563404	-19.71893362	3.28E-64	3.09E-61
PKMYT1	-1.725052827	3.160409335	-19.61975858	9.80E-64	8.93E-61
PLK1	-1.673240876	4.295010593	-19.53471943	2.50E-63	2.21E-60
RECQL4	-1.593838951	4.234755858	-19.53132461	2.60E-63	2.23E-60
BUB1B	-1.751161151	3.474857722	-19.49057567	4.07E-63	3.40E-60
CENPE	-1.72359269	3.153110557	-19.45059155	6.33E-63	5.13E-60
HELLS	-1.346294215	3.63039402	-19.40736087	1.02E-62	8.04E-60
BRCA1	-1.409300707	3.730651388	-19.37803146	1.41E-62	1.08E-59
CHEK1	-1.448492071	3.734696491	-19.35602303	1.79E-62	1.34E-59
CDCA3	-1.713528321	3.06963127	-19.25712299	5.33E-62	3.89E-59
CENPI	-1.683104548	1.969968269	-19.14902549	1.75E-61	1.25E-58
CENPA	-2.037381516	2.077816956	-19.11308076	2.60E-61	1.81E-58
PLK4	-1.408403308	2.82628836	-19.06623066	4.35E-61	2.96E-58
NDC80	-1.776487541	3.092565057	-19.00416611	8.61E-61	5.71E-58
CDC20	-1.883001015	4.609680762	-18.97514098	1.18E-60	7.69E-58
MELK	-1.963772946	3.407853396	-18.96051053	1.39E-60	8.83E-58
CDCA8	-1.586459017	3.915191466	-18.94744063	1.61E-60	9.97E-58
AUNIP	-1.678261075	1.012308989	-18.91562254	2.28E-60	1.39E-57
STIL	-1.313127473	3.407825563	-18.90580544	2.54E-60	1.51E-57
BUB1	-1.693905063	4.160629446	-18.86650174	3.91E-60	2.28E-57
DNMT3B	-1.60088784	2.283676043	-18.7790166	1.02E-59	5.84E-57
E2F1	-1.489856337	4.152826475	-18.76126889	1.24E-59	6.96E-57
CLSPN	-1.796742739	2.380660085	-18.74945102	1.41E-59	7.77E-57

PRC1	-1.483640983	4.699980253	-18.73134575	1.72E-59	9.30E-57
FAM72A	-1.589623218	-0.265368129	-18.72884381	1.77E-59	9.38E-57
FANCI	-1.237206516	4.682661881	-18.72095123	1.93E-59	1.00E-56
KIF15	-1.613551785	2.616797909	-18.68595988	2.83E-59	1.45E-56
KNTC1	-1.186096854	4.609824108	-18.61068411	6.46E-59	3.25E-56
CHAF1A	-1.010014825	4.048655731	-18.5811564	8.92E-59	4.41E-56
KIF11	-1.484546154	4.365984968	-18.55186572	1.23E-58	5.98E-56
CKAP2L	-1.774878069	2.922439528	-18.53079482	1.55E-58	7.41E-56
CDT1	-1.617991978	3.672487452	-18.52735741	1.61E-58	7.57E-56
TACC3	-1.190116711	5.45686226	-18.49327137	2.33E-58	1.08E-55
UBE2C	-2.173851944	4.549435657	-18.49088341	2.40E-58	1.09E-55
C17orf53	-1.534441558	2.343638921	-18.45878451	3.40E-58	1.52E-55
DTL	-1.438390834	3.684364191	-18.4580541	3.43E-58	1.52E-55
E2F2	-1.484759429	2.467035113	-18.43004843	4.66E-58	2.03E-55
IQGAP3	-1.643301791	4.530754896	-18.41258349	5.64E-58	2.42E-55
PRR11	-1.700530369	3.671691189	-18.38497618	7.63E-58	3.23E-55
FAM72B	-1.839894096	-0.449883242	-18.36578348	9.41E-58	3.92E-55
ORC1	-1.617608366	2.444589509	-18.29895186	1.95E-57	8.02E-55
ZWINT	-1.402373145	4.67523366	-18.27985412	2.40E-57	9.75E-55
AURKB	-1.84630603	3.289397293	-18.23787959	3.80E-57	1.52E-54
DLGAP5	-1.870136413	3.51377542	-18.2049961	5.44E-57	2.15E-54
TIMELESS	-1.091817811	5.343542245	-18.2037547	5.52E-57	2.15E-54
ATAD5	-1.070985289	2.79195082	-18.19167003	6.29E-57	2.42E-54
C16orf59	-1.414703805	2.347225868	-18.18318428	6.90E-57	2.62E-54
CEP55	-1.7311927	4.064347551	-18.14255003	1.08E-56	4.02E-54
BRIP1	-1.481560681	2.563574229	-18.14116411	1.09E-56	4.03E-54
NEK2	-1.830466937	3.429703981	-18.06401512	2.53E-56	9.23E-54
NCAPG	-1.805939414	3.388443374	-18.04936072	2.97E-56	1.07E-53
NCAPG2	-1.225049137	4.671188777	-18.03402808	3.51E-56	1.25E-53
CDC25A	-1.554378874	2.03056995	-17.93756246	1.00E-55	3.52E-53
SGOL1	-1.681074176	1.722801698	-17.93468774	1.03E-55	3.59E-53
GINS1	-1.520133376	3.566424594	-17.86475592	2.21E-55	7.59E-53
MCM4	-1.2876932	6.209875186	-17.85396105	2.49E-55	8.44E-53
TTK	-1.833588283	2.845109886	-17.84156145	2.85E-55	9.55E-53
NUF2	-1.838503767	3.124339931	-17.77746663	5.71E-55	1.89E-52
INCENP	-1.087692957	4.402645694	-17.76273438	6.70E-55	2.20E-52
CTD_2510F5.4	-1.716222865	0.440620458	-17.75829404	7.03E-55	2.28E-52
BLM	-1.250019349	2.845119079	-17.75108847	7.60E-55	2.44E-52
CCNB2	-1.607164514	3.902855988	-17.74271325	8.33E-55	2.62E-52
SKA1	-1.812487015	2.01027475	-17.74231543	8.36E-55	2.62E-52
ORC6	-1.452525633	2.597908608	-17.72553119	1.00E-54	3.11E-52
BIRC5	-1.889600439	4.794047159	-17.71328586	1.15E-54	3.52E-52
DSCC1	-1.423928137	2.549768188	-17.62319375	3.04E-54	9.25E-52
ANLN	-1.879874476	4.941142786	-17.62125095	3.11E-54	9.35E-52
KPNA2	-1.286581562	6.470272164	-17.60478211	3.72E-54	1.11E-51
PSMC3IP	-1.035732674	1.927527709	-17.53635075	7.80E-54	2.30E-51
GSG2	-1.454099173	1.053775792	-17.48234223	1.40E-53	4.08E-51
SKA3	-1.636116189	2.375620746	-17.46837188	1.63E-53	4.70E-51
DEPDC1	-1.940446972	2.819978348	-17.44271783	2.15E-53	6.14E-51
WDHD1	-1.250230032	3.727516645	-17.37810613	4.32E-53	1.22E-50
MCM2	-1.284485713	5.479333404	-17.27636007	1.29E-52	3.63E-50
LMNB2	-1.030288049	6.083320992	-17.26700393	1.43E-52	3.98E-50
KIF20A	-1.508094894	3.916431667	-17.23088437	2.11E-52	5.82E-50
E2F8	-1.493592585	2.600057047	-17.21207301	2.59E-52	7.06E-50

TRIP13	-1.722087561	3.914122963	-17.18575799	3.44E-52	9.20E-50
DBF4B	-1.022401599	3.069867384	-17.18573534	3.44E-52	9.20E-50
CDCA2	-1.625730196	2.129598377	-17.14069386	5.58E-52	1.48E-49
ASF1B	-1.363666467	3.958048993	-17.12730168	6.45E-52	1.70E-49
SPC24	-1.463871269	2.743683435	-17.09165828	9.47E-52	2.47E-49
CASC5	-1.462623996	2.98956251	-17.06708718	1.23E-51	3.18E-49
EZH2	-1.234184373	4.110296816	-17.04444155	1.57E-51	4.03E-49
NUSAP1	-1.372730978	4.582862516	-16.98160361	3.09E-51	7.84E-49
ARHGAP11A	-1.40813683	3.758129181	-16.9217209	5.88E-51	1.47E-48
LMNB1	-1.179974608	5.266895983	-16.89662284	7.70E-51	1.90E-48
ATAD2	-1.209484313	5.29963025	-16.81311727	1.88E-50	4.62E-48
KIAA1524	-1.475785045	2.808352132	-16.77732026	2.76E-50	6.72E-48
ERCC6L	-1.477965841	1.792180494	-16.76496158	3.16E-50	7.61E-48
WDR62	-1.54745062	3.240801864	-16.75309789	3.58E-50	8.57E-48
MTBP	-1.08078649	2.356755256	-16.68301374	7.58E-50	1.80E-47
RRM2	-1.680391953	5.243083711	-16.66561472	9.13E-50	2.15E-47
TRAIP	-1.034686681	2.200186318	-16.63413875	1.28E-49	2.98E-47
RP11_424C20.2	-1.725722301	-1.196684946	-16.62986592	1.34E-49	3.10E-47
RACGAP1	-1.223939813	4.866555897	-16.61564318	1.56E-49	3.58E-47
FANCA	-1.093111707	3.766998556	-16.61408773	1.58E-49	3.61E-47
CCNA2	-1.572787415	3.88913623	-16.60752682	1.70E-49	3.84E-47
POLE2	-1.280105203	2.315922059	-16.58288191	2.21E-49	4.96E-47
MCM6	-1.03146901	5.596964664	-16.5634845	2.72E-49	6.06E-47
SAPCD2	-1.586975404	3.618006657	-16.51607125	4.51E-49	9.89E-47
MTFR2	-1.465165774	1.565719304	-16.50428873	5.11E-49	1.11E-46
PIF1	-1.593048441	1.570593666	-16.46520787	7.75E-49	1.68E-46
CDC25C	-1.598997926	1.703741799	-16.45360161	8.77E-49	1.88E-46
CCNF	-1.061336712	4.07176649	-16.4113146	1.38E-48	2.93E-46
FAM64A	-1.756717363	2.14251802	-16.38236979	1.87E-48	3.96E-46
RAD51AP1	-1.440998797	2.885943178	-16.37115386	2.11E-48	4.43E-46
AURKA	-1.509025272	4.13903678	-16.27045479	6.15E-48	1.28E-45
SHCBP1	-1.35350629	3.14303139	-16.13025059	2.72E-47	5.63E-45
TONSL	-1.15457611	4.274098341	-16.10694681	3.48E-47	7.15E-45
PSRC1	-1.16603577	2.673667023	-16.10361852	3.61E-47	7.36E-45
RAD51	-1.351624014	2.437963056	-16.08972763	4.18E-47	8.47E-45
CCDC150	-1.470741169	0.824299237	-16.03653101	7.33E-47	1.48E-44
TYMS	-1.197161165	4.281307465	-16.00440519	1.03E-46	2.06E-44
FAM83D	-1.590484528	3.420640954	-15.89911097	3.13E-46	6.17E-44
FAM72D	-1.832447259	-1.280094518	-15.83075579	6.43E-46	1.25E-43
UBE2T	-1.382077904	3.887804034	-15.80969172	8.02E-46	1.54E-43
SPC25	-1.407488858	1.889567052	-15.78896207	9.98E-46	1.90E-43
ARHGAP11B	-1.158413885	1.935507679	-15.74290463	1.62E-45	3.07E-43
CDK1	-1.474752961	4.664660235	-15.70244134	2.48E-45	4.66E-43
FANCB	-1.25405251	0.060748012	-15.59003367	8.05E-45	1.50E-42
CCNB1	-1.398969317	4.96891907	-15.52548256	1.58E-44	2.92E-42
DEPDC1B	-1.645394658	2.406493038	-15.47780977	2.61E-44	4.75E-42
DNA2	-1.140363951	2.846453075	-15.46620712	2.94E-44	5.33E-42
CDKN3	-1.628475858	2.660208808	-15.39728036	6.04E-44	1.09E-41
FAM111B	-1.447468208	3.1469157	-15.30632887	1.56E-43	2.77E-41
RFC4	-1.077557275	3.974254632	-15.29636818	1.73E-43	3.04E-41
RAD54B	-1.00296722	1.336454501	-15.2455933	2.93E-43	5.10E-41
ARHGEF39	-1.12277253	2.575142393	-15.12417729	1.03E-42	1.79E-40
CHAF1B	-1.002374053	3.530405915	-15.09917214	1.34E-42	2.30E-40
CENPU	-1.288109052	3.636799839	-15.05326478	2.16E-42	3.66E-40

CDC7	-1.110768173	3.110002787	-15.04336475	2.39E-42	4.03E-40
CCNE1	-1.586056951	2.543109129	-15.02840526	2.79E-42	4.68E-40
DDIAS	-1.391205327	2.274403309	-14.99554901	3.92E-42	6.50E-40
TK1	-1.376987291	5.548552362	-14.98829765	4.22E-42	6.96E-40
SGOL2	-1.133785931	3.092180815	-14.97899386	4.65E-42	7.62E-40
GINS4	-1.345737067	2.415618928	-14.902072	1.03E-41	1.67E-39
C5orf34	-1.111211214	1.684474061	-14.61600376	1.94E-40	3.08E-38
DIAPH3	-1.320091463	2.537642677	-14.58361209	2.71E-40	4.27E-38
PARPBP	-1.215524349	2.358716748	-14.47245971	8.43E-40	1.32E-37
POC1A	-1.001861458	3.070270828	-14.42591164	1.35E-39	2.10E-37
CENPM	-1.212164989	2.764419592	-14.35983124	2.65E-39	4.05E-37
ESCO2	-1.333261325	1.987697461	-14.24977881	8.11E-39	1.22E-36
OIP5	-1.355881091	0.948057805	-14.24494784	8.52E-39	1.27E-36
E2F7	-1.649076354	1.426074971	-14.14523657	2.34E-38	3.44E-36
CENPW	-1.302983476	2.455277981	-14.13246454	2.66E-38	3.90E-36
MAD2L1	-1.343447418	3.864107023	-14.06480283	5.26E-38	7.60E-36
CCNE2	-1.168630344	2.039034356	-14.05887283	5.58E-38	8.03E-36
CTSV	-2.019303733	2.117410862	-13.9760609	1.29E-37	1.84E-35
INMT	1.810481766	3.793509505	13.94969382	1.68E-37	2.37E-35
C16orf89	2.861343946	6.175670584	13.94361506	1.78E-37	2.51E-35
RP11_443B20.1	-1.026675515	0.088725465	-13.89143121	3.01E-37	4.22E-35
C1QTNF7	1.839651326	0.903705835	13.85549441	4.31E-37	5.99E-35
ZNF367	-1.056349997	2.366309834	-13.76435863	1.07E-36	1.48E-34
KIF24	-1.015500396	2.200363969	-13.74318582	1.32E-36	1.82E-34
GINS2	-1.306966374	3.168196861	-13.68835398	2.29E-36	3.14E-34
DDX12P	-1.245871532	1.305912207	-13.53981592	1.00E-35	1.35E-33
KIAA0101	-1.275879461	3.749997219	-13.51981	1.22E-35	1.64E-33
SFTA1P	2.401306661	2.804466689	13.47882151	1.83E-35	2.43E-33
TMPO_AS1	-1.019886457	1.030217752	-13.38063959	4.84E-35	6.30E-33
RP11_635O16.2	2.777650648	-2.633003573	13.3780586	4.96E-35	6.44E-33
CENPK	-1.155274185	2.404693713	-13.36260218	5.78E-35	7.43E-33
PBK	-1.638083274	2.62632171	-13.32719973	8.19E-35	1.04E-32
UBE2S	-1.199127378	3.924141843	-13.28698018	1.22E-34	1.53E-32
KIF18A	-1.063681381	2.546467465	-13.26841058	1.46E-34	1.83E-32
HMMR	-1.34283809	3.450498661	-13.24170091	1.90E-34	2.37E-32
ADH1B	2.791143694	3.778830037	13.21982222	2.35E-34	2.90E-32
GPR19	-1.35793273	-0.294419159	-13.20598968	2.70E-34	3.30E-32
CTSH	1.437630707	8.441333768	13.1415333	5.07E-34	6.08E-32
FCER1A	2.316923954	1.060735008	12.94908661	3.31E-33	3.89E-31
CDH24	-1.139011327	3.420505698	-12.94514808	3.44E-33	4.03E-31
ACRV1	-1.442665847	-2.505662627	-12.84590123	8.99E-33	1.05E-30
ECT2	-1.066539257	5.371385025	-12.62519003	7.54E-32	8.63E-30
SCN7A	1.705216115	3.148332301	12.50937597	2.29E-31	2.55E-29
MGP	1.225126166	7.261396956	12.49435043	2.64E-31	2.92E-29
RP4_694A7.2	-1.687466736	-4.364557514	-12.45005983	4.02E-31	4.40E-29
MND1	-1.106711293	1.358428052	-12.43766985	4.53E-31	4.91E-29
FAM72C	-1.729055992	-2.053095691	-12.37278492	8.39E-31	9.04E-29
CFAP221	1.858420339	3.011492495	12.34241016	1.12E-30	1.20E-28
CYBRD1	1.149078733	6.609304634	12.3177483	1.41E-30	1.51E-28
LINC00634	-1.694676575	-2.987797821	-12.22718276	3.33E-30	3.47E-28
RNASE1	1.469538811	8.450256125	12.20127229	4.25E-30	4.42E-28
ATP1A2	1.78598684	0.19039781	12.19890932	4.35E-30	4.50E-28
KIF3C	-1.22224504	3.893524655	-12.15458107	6.60E-30	6.74E-28
RDM1	-1.468305875	-0.789081419	-12.1299488	8.32E-30	8.46E-28

PLLP	1.209610515	4.460946595	12.11906306	9.22E-30	9.31E-28
SCGB3A2	3.329942087	5.492938257	12.11830769	9.29E-30	9.34E-28
CTD_2006C1.6	-1.181266856	-2.817543014	-12.04237377	1.89E-29	1.89E-27
STMN1	-1.008683798	6.92619882	-12.03164667	2.09E-29	2.08E-27
SHH	2.046515665	1.701412776	12.01017949	2.56E-29	2.52E-27
RP11_381E24.1	-1.183331163	-2.525674696	-11.99433082	2.97E-29	2.91E-27
EMCN	1.117873082	3.165858774	11.95972015	4.10E-29	3.99E-27
MAMDC2	1.704596479	2.369988021	11.92281438	5.78E-29	5.57E-27
ABCA8	1.607201148	2.179888565	11.89841582	7.25E-29	6.94E-27
NEIL3	-1.655057257	1.314393481	-11.87660269	8.89E-29	8.45E-27
MTL5	-1.740648097	1.50613369	-11.83577328	1.30E-28	1.22E-26
FOLR1	2.19482994	5.97955947	11.83298106	1.33E-28	1.24E-26
SLC22A3	1.885087607	4.059524965	11.83286907	1.33E-28	1.24E-26
ROBO2	1.751162953	1.888421199	11.81307836	1.60E-28	1.48E-26
TMEM163	1.623529535	4.392734605	11.78598414	2.06E-28	1.88E-26
OTC	1.691248639	-4.742334235	11.78175999	2.14E-28	1.93E-26
MAP6D1	-1.016396204	0.890806833	-11.77308992	2.32E-28	2.09E-26
CTD_2318O12.1	-1.698440374	-3.29168166	-11.76476759	2.51E-28	2.24E-26
HOPX	1.831008681	7.370217345	11.70713626	4.27E-28	3.74E-26
HMGA1	-1.054480764	7.664072136	-11.68884148	5.05E-28	4.39E-26
ADAMTS9_AS2	1.411097714	-0.822546675	11.68356127	5.30E-28	4.59E-26
C2orf48	-1.528142205	-1.406816025	-11.63944656	7.96E-28	6.81E-26
LINC00337	-1.674660591	-1.548587232	-11.57408867	1.45E-27	1.22E-25
ADAMTS8	1.836253872	1.11529792	11.55400431	1.74E-27	1.46E-25
ZNF695	-2.160877462	-1.074858689	-11.52956855	2.18E-27	1.82E-25
ZNF670	-1.389937768	-2.908287769	-11.49986454	2.86E-27	2.38E-25
PTTG1	-1.085853999	4.224587778	-11.46735714	3.84E-27	3.18E-25
CD1C	1.670825229	1.516789156	11.43298528	5.26E-27	4.31E-25
LINC00940	2.075470576	-4.278575421	11.41127612	6.41E-27	5.24E-25
SCN4B	1.318924383	1.935136629	11.39863214	7.19E-27	5.84E-25
MFAP4	1.401311433	5.777011725	11.36490946	9.76E-27	7.78E-25
IL33	1.328144549	3.944849398	11.32119866	1.45E-26	1.15E-24
C8orf34	1.660386587	0.700670159	11.31459095	1.54E-26	1.22E-24
CD300LG	2.142066748	-2.634377263	11.31203284	1.58E-26	1.24E-24
AC015849.15	-1.573954121	-3.094228966	-11.29292438	1.87E-26	1.47E-24
C7	1.917623491	5.279278746	11.27566928	2.19E-26	1.70E-24
GNG7	1.047077725	2.699140662	11.26925738	2.32E-26	1.80E-24
CYP4B1	2.453097782	4.711084156	11.26810858	2.34E-26	1.81E-24
FBXO43	-1.126464502	-0.815210869	-11.25106787	2.73E-26	2.11E-24
CD302	1.009367545	3.550934989	11.24031642	3.01E-26	2.31E-24
TMEM132A	-1.065779918	5.496468784	-11.21191046	3.89E-26	2.95E-24
LINC00968	1.456354726	-1.366064681	11.18403889	5.00E-26	3.77E-24
AC018647.3	1.233925351	-2.636356254	11.17581076	5.38E-26	4.04E-24
HIGD1B	1.096721036	0.920141738	11.15181484	6.67E-26	4.99E-24
CYB5A	1.028059472	6.930053126	11.14822629	6.89E-26	5.13E-24
GFRA1	1.822011895	1.097981025	11.12412343	8.55E-26	6.30E-24
NPC2	1.087708938	8.790325283	11.10138943	1.05E-25	7.69E-24
DLC1	1.2142798	5.714857967	11.07080623	1.38E-25	9.90E-24
AC079630.4	2.091529595	1.134041385	11.03657498	1.87E-25	1.34E-23
SUSD2	1.988401627	6.015805172	11.03459476	1.90E-25	1.36E-23
GPC2	-1.513754392	0.404466207	-11.00672214	2.44E-25	1.73E-23
PEBP4	2.527716671	2.337119687	10.99862692	2.62E-25	1.85E-23
C1orf116	1.595185551	7.065527703	10.96856703	3.43E-25	2.40E-23
CD1E	1.849165102	0.770992267	10.95661493	3.81E-25	2.66E-23

CHRDL1	1.783714806	3.174165446	10.95486238	3.87E-25	2.70E-23
RP11_564D11.3	-1.285523523	-0.533307112	-10.94030843	4.40E-25	3.06E-23
ART4	1.487811585	-0.543917143	10.92442482	5.07E-25	3.51E-23
RP11_85I17.2	-1.288367343	-2.984695679	-10.89120367	6.80E-25	4.68E-23
RSPO2	2.073999191	-1.173419202	10.88574136	7.14E-25	4.90E-23
FIGF	2.097391616	0.64073651	10.87073513	8.15E-25	5.57E-23
RCOR2	-1.565473384	1.455539501	-10.85386322	9.46E-25	6.44E-23
CD207	2.423377014	1.400500823	10.82742785	1.19E-24	8.07E-23
CACNA2D2	2.083620038	4.409546084	10.78858122	1.68E-24	1.12E-22
SDPR	1.201125347	4.723793952	10.74585164	2.45E-24	1.61E-22
HPGDS	1.428943813	1.331046437	10.72889053	2.84E-24	1.86E-22
AC015849.16	-1.525212951	-1.808360338	-10.72623481	2.91E-24	1.89E-22
SELENBP1	1.306438038	6.971701363	10.71425655	3.23E-24	2.09E-22
C2orf40	2.090441675	-0.633795469	10.70534766	3.49E-24	2.26E-22
CX3CR1	1.476878386	1.452278205	10.70323746	3.56E-24	2.29E-22
PGM5P4	1.669045003	-3.574881856	10.70318684	3.56E-24	2.29E-22
DRP2	-1.553601582	-0.835116999	-10.69840988	3.71E-24	2.38E-22
TYMSOS	-1.16002193	-0.822019769	-10.68991359	4.00E-24	2.56E-22
DDN	-1.541032549	-1.53051728	-10.66641072	4.91E-24	3.12E-22
FHL5	1.239866975	0.496222289	10.630291	6.73E-24	4.25E-22
CYP4Z2P	2.142542739	-2.546009632	10.6263751	6.96E-24	4.37E-22
MYOCD	1.646466917	-0.102141279	10.62302567	7.17E-24	4.49E-22
UCK2	-1.143548835	4.420240964	-10.62128273	7.28E-24	4.55E-22
LPAL2	1.329141982	0.053836027	10.56337828	1.20E-23	7.29E-22
RBPMS_AS1	1.199943942	0.748595458	10.5524898	1.32E-23	7.98E-22
NAPSA	2.307641364	9.337005359	10.54957748	1.36E-23	8.17E-22
SCTR	2.30494387	2.699735875	10.53829377	1.50E-23	8.99E-22
NFIX	1.015008845	5.850780575	10.53499463	1.54E-23	9.24E-22
RP1_186E20.1	1.753732322	-2.896585041	10.51630849	1.81E-23	1.08E-21
ZYG11A	-2.014920069	0.580256752	-10.5148136	1.84E-23	1.09E-21
CAV3	1.459690005	-4.606033294	10.50082312	2.07E-23	1.23E-21
AC004947.2	1.619230405	-3.065175114	10.47569694	2.58E-23	1.51E-21
GGTLC1	2.53499242	1.885529294	10.43971013	3.51E-23	2.01E-21
EIF4EBP1	-1.007891716	5.01163307	-10.43738838	3.58E-23	2.05E-21
RNU1_38P	1.382034375	-5.301438826	10.41441855	4.37E-23	2.48E-21
NR3C2	1.168111949	3.530246865	10.40882261	4.58E-23	2.59E-21
GPR116	1.349826549	7.148645132	10.37407265	6.18E-23	3.47E-21
HLF	1.680623226	3.210925854	10.36303021	6.79E-23	3.80E-21
ZNF724P	-1.014419747	0.05607733	-10.34127632	8.19E-23	4.57E-21
SEPP1	1.049375272	6.135105155	10.33715872	8.48E-23	4.72E-21
DUSP1	1.131496428	7.745593148	10.31048955	1.07E-22	5.87E-21
CST5	1.877424844	-2.661965072	10.29442119	1.22E-22	6.71E-21
ITIH5	1.315133119	3.371322037	10.26092266	1.63E-22	8.88E-21
LRRK2	1.687166783	5.331937219	10.2279348	2.16E-22	1.16E-20
RP11_664D7.4	2.231263598	0.939529385	10.22543206	2.20E-22	1.18E-20
C15orf59	1.380459824	0.207317688	10.21238162	2.46E-22	1.32E-20
ITGA9	1.014528254	4.74597234	10.20385941	2.65E-22	1.41E-20
RAB3B	-2.268032212	1.001322497	-10.16211123	3.77E-22	1.99E-20
RP11_480C22.1	1.502036184	-4.924197713	10.15661479	3.95E-22	2.07E-20
RNFT2	-1.116232887	2.11088785	-10.13041078	4.93E-22	2.57E-20
SFTPB	2.474132556	11.46842637	10.11140388	5.79E-22	3.00E-20
RP11_259K15.2	2.006439665	-0.143457217	10.09853139	6.46E-22	3.32E-20
FXYD1	1.143892898	-1.195639418	10.07569	7.83E-22	4.02E-20
HIST1H3B	-1.761698569	-3.864212032	-10.06123401	8.85E-22	4.50E-20

EDN3	1.822806091	-4.52215125	10.05005315	9.72E-22	4.93E-20
LEFTY2	1.700969854	-1.221384426	10.03187086	1.13E-21	5.72E-20
IGF2BP3	-2.339010866	1.966379252	-10.02647359	1.19E-21	5.98E-20
HPSE2	1.639551242	-1.279709846	10.01034955	1.36E-21	6.83E-20
FAM150B	1.342801056	-1.354181405	10.00785526	1.39E-21	6.96E-20
RP11_793H13.3	1.492763454	-3.168656961	9.949707256	2.26E-21	1.11E-19
TCF21	1.325016842	1.795342936	9.949480413	2.26E-21	1.11E-19
EDA2R	1.423574072	0.841039586	9.939281776	2.46E-21	1.21E-19
PGM5	1.176110274	2.667207819	9.938171817	2.49E-21	1.22E-19
SCGB3A1	2.832555669	4.83926076	9.930106134	2.66E-21	1.30E-19
RPL26P30	1.354300002	-1.761470238	9.921955555	2.85E-21	1.39E-19
TERT	-1.678034199	-1.410053616	-9.915867275	2.99E-21	1.45E-19
SPATA18	1.447402654	2.556995236	9.911388439	3.11E-21	1.50E-19
CASQ2	1.485158051	-1.094950453	9.91062473	3.13E-21	1.51E-19
GRIA1	1.953069303	-0.996980388	9.897909236	3.48E-21	1.66E-19
CYP2B7P	2.055505805	5.634604025	9.895461794	3.55E-21	1.69E-19
PRG4	1.839249005	1.467783937	9.873373906	4.27E-21	2.02E-19
HPDL	-1.467300847	-0.012632507	-9.87251469	4.30E-21	2.03E-19
ITGA8	1.052859772	3.518348197	9.870914962	4.35E-21	2.06E-19
SGCA	1.235481293	1.406213712	9.853835899	5.02E-21	2.35E-19
SLC7A5	-1.135128307	6.496489522	-9.844364315	5.43E-21	2.52E-19
SFTPD	2.106620013	6.025698951	9.831401355	6.05E-21	2.80E-19
FHL1	1.113262136	4.382931577	9.824103757	6.42E-21	2.97E-19
SLC26A9	1.990656654	4.141115517	9.805698787	7.48E-21	3.44E-19
AFF3	1.463310432	1.82261233	9.751757111	1.17E-20	5.31E-19
RP11_251M1.1	1.145632989	-2.479102822	9.746711414	1.22E-20	5.53E-19
MEX3A	-1.282038247	4.239600875	-9.739409294	1.29E-20	5.86E-19
PGR	1.084541195	0.734502076	9.713919002	1.60E-20	7.16E-19
TMEM100	1.570286136	1.720950412	9.694484246	1.87E-20	8.36E-19
ANGPTL5	1.28652614	-3.453662985	9.664929887	2.39E-20	1.06E-18
TBX4	1.199955187	2.662574086	9.655768739	2.58E-20	1.14E-18
SMCO3	1.022278402	-1.177275409	9.64340913	2.85E-20	1.25E-18
PLA2G1B	2.104342548	-0.056924606	9.617237951	3.53E-20	1.54E-18
SFTA2	1.709433003	6.330374156	9.608266081	3.80E-20	1.65E-18
CD1A	2.069290036	1.337583447	9.599862957	4.07E-20	1.76E-18
PLA2G4F	1.44574773	2.86498945	9.58679615	4.53E-20	1.94E-18
RP1_35C21.1	1.275114577	-4.487513559	9.564568592	5.43E-20	2.32E-18
RP11_88H9.2	1.298977883	-2.891311084	9.560160561	5.63E-20	2.39E-18
ASPA	1.113402021	-0.500918427	9.554204753	5.91E-20	2.50E-18
GUCA1A	-1.647335968	-3.047389219	-9.552839511	5.97E-20	2.53E-18
RP11_1099M24.8	1.226015834	-4.944577695	9.541483381	6.55E-20	2.76E-18
HSPB7	1.032925582	1.726065373	9.533920803	6.97E-20	2.93E-18
AC109642.1	1.149634045	0.804111057	9.49383348	9.65E-20	4.03E-18
RP11_93209.10	-1.016747011	-2.541712628	-9.487376455	1.02E-19	4.23E-18
PTCSC3	1.928917284	-1.036128366	9.472484039	1.15E-19	4.76E-18
SLC14A1	1.215195451	0.094021849	9.467821445	1.19E-19	4.91E-18
MYOC	1.542444347	-3.703049093	9.466970602	1.20E-19	4.93E-18
ST3GAL5	1.033683755	5.884047674	9.452610199	1.35E-19	5.49E-18
CLEC9A	1.260353091	-1.771159648	9.448568615	1.39E-19	5.65E-18
CLEC3B	1.158978447	2.397915392	9.443798522	1.45E-19	5.86E-18
RP11_373N22.3	1.062989724	-1.702294388	9.411943244	1.87E-19	7.53E-18
P2RY12	1.388731493	-0.268743171	9.407139922	1.95E-19	7.81E-18
ACSM1	1.182720874	-0.499158766	9.407010927	1.95E-19	7.81E-18
RP11_98G7.1	-1.627651566	-2.376963654	-9.40239889	2.02E-19	8.07E-18

RP11_213O5.4	1.359227644	-3.160078762	9.386953026	2.29E-19	9.07E-18
GAPT	1.054715851	0.934682853	9.378217163	2.46E-19	9.71E-18
GPRC5C	1.113559835	6.113249213	9.370688955	2.61E-19	1.03E-17
RP3_512B11.3	-1.257446251	-0.019098856	-9.36758415	2.67E-19	1.05E-17
RP11_357D18.1	2.026910515	-1.93059479	9.362346819	2.79E-19	1.10E-17
ELN	1.226208719	6.0407593	9.35132148	3.05E-19	1.19E-17
RP11_365O16.3	1.325111778	-3.104270505	9.334666902	3.48E-19	1.35E-17
ELANE	1.682079045	-2.948978805	9.33404648	3.50E-19	1.35E-17
OLFM1	1.280266591	2.662390054	9.331134965	3.58E-19	1.39E-17
ACKR1	1.513615634	2.714159396	9.323778491	3.80E-19	1.46E-17
CTD_2529O21.1	-1.160629944	-4.237817681	-9.319815805	3.92E-19	1.50E-17
UNC45B	1.084158718	-2.423848029	9.268314848	5.92E-19	2.23E-17
ENTPD3	1.156239659	3.298437847	9.248395734	6.94E-19	2.61E-17
C1QL4	-1.539153868	-4.212293179	-9.24462208	7.15E-19	2.68E-17
RP11_244M2.1	-1.533140224	-1.307312086	-9.238102266	7.53E-19	2.82E-17
CCL14	1.417407197	-0.533916359	9.235669291	7.68E-19	2.87E-17
MAOB	1.032740604	3.655009331	9.214224793	9.11E-19	3.38E-17
NTRK3	1.189692699	-0.099154278	9.19968985	1.02E-18	3.77E-17
RP11_95I16.2	1.510278437	-4.403689696	9.193416018	1.07E-18	3.96E-17
RP11_789C1.1	2.032524257	-3.132081367	9.191514064	1.09E-18	4.01E-17
MACROD2	1.544725645	3.037422101	9.18511439	1.15E-18	4.21E-17
RP11_121C6.5	-1.110629225	-3.931303658	-9.184714273	1.15E-18	4.22E-17
RP11_87E22.2	1.51442565	-3.605168554	9.181365118	1.18E-18	4.33E-17
TMEM132C	1.829696999	-2.913959158	9.174877946	1.25E-18	4.54E-17
FAM171A2	-1.22950002	1.702723021	-9.170988483	1.28E-18	4.68E-17
C19orf26	-1.086327943	0.432301088	-9.165691899	1.34E-18	4.87E-17
MMP28	1.733672781	3.24666027	9.158960554	1.41E-18	5.11E-17
HSD17B6	1.365815127	3.338967045	9.158144572	1.42E-18	5.13E-17
F11	2.144307735	-1.964177205	9.155248046	1.45E-18	5.25E-17
PLA2G10	1.71134754	0.918934692	9.150032906	1.52E-18	5.45E-17
AC090616.2	1.13040938	0.386954597	9.149454291	1.52E-18	5.47E-17
SCNN1B	1.42432486	5.137901554	9.147005599	1.55E-18	5.56E-17
B3GNT8	1.160588683	3.604197246	9.141720935	1.62E-18	5.78E-17
ABI3BP	1.070358515	4.153003237	9.127817277	1.81E-18	6.41E-17
LINC00261	2.166773685	2.25336694	9.127434006	1.81E-18	6.43E-17
CTB_51J22.1	1.244858975	0.043673592	9.091503014	2.41E-18	8.38E-17
RP11_354E11.2	1.123973513	-1.484446963	9.091283927	2.41E-18	8.38E-17
ZNF385B	1.834357582	2.173576158	9.089509751	2.44E-18	8.47E-17
SLC14A2_AS1	1.091650723	-4.870517656	9.085165176	2.53E-18	8.74E-17
VSIG2	1.774027968	3.23188011	9.083926345	2.55E-18	8.79E-17
CLEC4F	1.569346978	-1.714837418	9.083478596	2.56E-18	8.81E-17
SNTN	1.968079602	0.294720738	9.082965665	2.57E-18	8.83E-17
GKN2	2.720535749	0.026507843	9.078367002	2.67E-18	9.14E-17
AQP4	2.272977055	4.572023365	9.07091843	2.83E-18	9.67E-17
VIPR1	1.11604744	3.350395552	9.062556446	3.02E-18	1.03E-16
AC079630.2	2.079683507	-2.757204448	9.061505852	3.05E-18	1.04E-16
C5orf38	1.753141102	1.743607044	9.044027016	3.49E-18	1.18E-16
RGS13	1.174902356	-0.530729801	9.042275301	3.54E-18	1.20E-16
RP11_630A13.4	1.699239069	-0.304658733	9.040717995	3.59E-18	1.21E-16
PSAT1	-1.173230558	4.204848206	-9.015358733	4.37E-18	1.46E-16
HAGLR	1.462122777	4.582022952	9.00088666	4.90E-18	1.62E-16
CTSG	1.603394058	-0.829001917	8.994326085	5.16E-18	1.70E-16
ANGPT1	1.055989467	2.850351535	8.993726642	5.18E-18	1.71E-16
KCND3	1.107812253	2.165825095	8.993632143	5.18E-18	1.71E-16

RP11_332J15.4	-1.154605866	-4.092242299	-8.98736664	5.44E-18	1.78E-16
LCN6	1.305370495	-4.062117892	8.977276423	5.89E-18	1.93E-16
OGN	1.538139435	1.171189367	8.966404861	6.41E-18	2.09E-16
CEACAM8	1.772305234	-3.358253898	8.965623149	6.45E-18	2.09E-16
CD1B	1.579570905	-1.048692363	8.964821223	6.49E-18	2.10E-16
CA3	1.362415027	0.508685228	8.956684266	6.92E-18	2.24E-16
SFTPA1	2.677447845	8.940978261	8.948352585	7.38E-18	2.37E-16
TMEM132E	1.266999488	-1.040309838	8.943040616	7.69E-18	2.46E-16
CCDC150P1	-1.174338165	-1.467130206	-8.928672954	8.60E-18	2.74E-16
FADS2	-1.057891226	6.146542571	-8.920140881	9.19E-18	2.92E-16
RP5_836N17.4	-1.12714003	-3.930479553	-8.907407112	1.01E-17	3.21E-16
NEGR1	1.08113081	1.608556266	8.903228223	1.05E-17	3.31E-16
DSP	-1.227742695	7.938701834	-8.896785981	1.10E-17	3.47E-16
APCDD1L	-1.861041471	-0.532281958	-8.892532473	1.14E-17	3.57E-16
KLF15	1.266438989	2.108712808	8.88818896	1.18E-17	3.68E-16
TSLP	1.23040317	-1.529467333	8.870596467	1.35E-17	4.21E-16
SHOX2	-1.630404436	-0.355059318	-8.870173451	1.35E-17	4.22E-16
KREMEN2	-1.236619869	-0.39991822	-8.869829936	1.36E-17	4.23E-16
CTSE	2.204724202	6.059324984	8.828096062	1.88E-17	5.77E-16
MYOM2	1.022765663	0.241023111	8.82267913	1.96E-17	6.01E-16
CDH20	1.664848585	-2.526305228	8.82186643	1.97E-17	6.04E-16
MAST1	-1.108336211	0.509709747	-8.820024294	2.00E-17	6.11E-16
ZBTB16	1.703167514	0.820826185	8.814690407	2.08E-17	6.34E-16
CHIA	2.241278634	1.140337503	8.805630781	2.23E-17	6.79E-16
ADRA1A	1.552378545	-3.475048864	8.800003328	2.33E-17	7.07E-16
RP5_1120P11.1	-1.344801641	1.004182483	-8.787577335	2.56E-17	7.74E-16
RHBG	-1.508324962	-1.022389256	-8.78441253	2.63E-17	7.92E-16
LINC01224	-2.189089883	-1.185156407	-8.781183232	2.69E-17	8.11E-16
RP1_163G9.2	1.635859184	-3.252623626	8.7596029	3.18E-17	9.51E-16
GDF10	1.471884781	0.403031734	8.716556394	4.42E-17	1.30E-15
SYNPR_AS1	1.611964607	-1.124398054	8.712102241	4.57E-17	1.34E-15
LINC00694	1.321591284	-2.911996455	8.706271076	4.78E-17	1.40E-15
ADH1A	1.202989261	-4.157119922	8.704368388	4.85E-17	1.42E-15
RNF144A_AS1	-1.31320894	-0.05014541	-8.690900326	5.37E-17	1.56E-15
ADAMTSL3	1.016936605	2.081046531	8.687927754	5.50E-17	1.59E-15
KIAA2022	1.663004112	-0.06098497	8.686772853	5.55E-17	1.60E-15
RP11_485G7.6	-1.026884333	-3.072719767	-8.682915103	5.71E-17	1.65E-15
LUZP2	1.777802575	-1.518505926	8.675343762	6.05E-17	1.74E-15
SLPI	1.563604334	7.182424114	8.675308074	6.05E-17	1.74E-15
UBE2SP1	-1.078916094	-1.946973026	-8.67175441	6.22E-17	1.78E-15
CCL17	1.513594888	0.38553614	8.666523882	6.47E-17	1.85E-15
RPL13AP17	2.020748879	-2.226043179	8.663841265	6.60E-17	1.89E-15
BTNL9	1.216144395	1.652304006	8.653775846	7.13E-17	2.03E-15
MYADML2	-1.099921347	-3.551978966	-8.641057668	7.85E-17	2.23E-15
RP1_137D17.1	1.139078659	-1.216073051	8.632990101	8.35E-17	2.36E-15
AP003774.6	1.067213048	-4.340042699	8.619785901	9.23E-17	2.61E-15
THSD7B	1.276035637	-0.741096356	8.605680402	1.03E-16	2.88E-15
TMPRSS2	1.202826849	6.164681258	8.603348977	1.05E-16	2.93E-15
SLC2A1	-1.114647424	6.782139442	-8.59461956	1.12E-16	3.11E-15
RP4_782L23.1	1.142014299	-2.661591709	8.579308489	1.25E-16	3.46E-15
SLC16A7	1.045913531	3.899242219	8.572072679	1.32E-16	3.65E-15
IRX2	1.835631045	3.489357475	8.567758437	1.37E-16	3.76E-15
LHFPL3_AS2	2.012658343	0.499010515	8.547460744	1.60E-16	4.37E-15
GNG4	-2.072744871	1.022376649	-8.542492659	1.66E-16	4.52E-15

FOS	1.016602556	7.673910032	8.539719935	1.69E-16	4.61E-15
SFTPA2	2.474647211	9.400736495	8.527334521	1.86E-16	5.04E-15
RP11_320N7.2	1.86796822	-1.641376052	8.512596565	2.07E-16	5.60E-15
SNORA71C	-1.063134449	-2.854765338	-8.512181835	2.08E-16	5.61E-15
RP11_548P2.2	1.097484898	-1.791067976	8.507157982	2.16E-16	5.82E-15
LINC00982	1.566995741	0.307045013	8.500486367	2.27E-16	6.08E-15
PGM5P4_AS1	1.194403815	-4.260840001	8.491883244	2.42E-16	6.45E-15
FMO2	1.058452509	4.339684665	8.485005251	2.55E-16	6.78E-15
AC079467.1	1.232907271	-5.113876506	8.473776919	2.78E-16	7.32E-15
CD40LG	1.071470198	0.272311101	8.467676579	2.91E-16	7.63E-15
PLD4	1.024604623	1.944564878	8.462654794	3.02E-16	7.89E-15
PRDM16	1.47725626	2.850324329	8.460358827	3.07E-16	8.02E-15
AC013264.2	1.558343332	-1.9867228	8.45512233	3.19E-16	8.31E-15
RP11_20J15.3	1.398373305	-2.53226355	8.451565103	3.28E-16	8.51E-15
IL12RB2	-1.149380806	-0.510628408	-8.415890989	4.28E-16	1.10E-14
RP11_44N12.5	-1.375071861	-2.933759771	-8.403214211	4.70E-16	1.20E-14
AC114730.3	-1.486647538	-2.356923985	-8.399548835	4.83E-16	1.23E-14
PNMA2	1.156243998	4.189907156	8.386740586	5.32E-16	1.35E-14
FMO5	1.220343637	5.019777884	8.386136571	5.34E-16	1.35E-14
RP11_559N14.5	-1.335454217	-1.482700398	-8.361667415	6.41E-16	1.61E-14
CFD	1.006368482	3.905971416	8.358914913	6.54E-16	1.64E-14
GPR133	1.259513585	4.286303748	8.356117127	6.67E-16	1.67E-14
MMRN1	1.068347434	2.445646459	8.355605098	6.70E-16	1.67E-14
TMPOP2	-1.028587659	-3.401548347	-8.349484993	7.01E-16	1.75E-14
MAPK10	1.010819269	1.989319119	8.348550509	7.06E-16	1.76E-14
PGC	3.133019004	4.863841487	8.334012789	7.86E-16	1.95E-14
PCSK2	2.930090105	-0.46358381	8.330388417	8.08E-16	2.00E-14
CTC_480C2.1	-1.713466702	-4.745064651	-8.323518517	8.50E-16	2.10E-14
PIGR	2.122634986	7.687978625	8.320348314	8.70E-16	2.15E-14
ACOXL	1.201281831	1.452606135	8.309748123	9.41E-16	2.31E-14
CBX2	-1.106006374	3.485166876	-8.307061604	9.60E-16	2.35E-14
RAP1GAP	1.163173714	5.831975508	8.305803444	9.69E-16	2.37E-14
DES	1.384319162	2.34618177	8.303843552	9.83E-16	2.40E-14
RP11_302F12.1	1.473825881	-0.630100151	8.295529621	1.05E-15	2.54E-14
MC5R	1.260693437	-4.733738592	8.29410778	1.06E-15	2.56E-14
CELSR3	-1.031483538	3.524305704	-8.293269212	1.06E-15	2.58E-14
RP11_22B23.2	-1.081652517	-4.615217467	-8.289648557	1.09E-15	2.64E-14
LINC01314	1.369641083	-1.12440724	8.288014453	1.10E-15	2.67E-14
CTB_134H23.3	1.529637497	-1.484964817	8.283696014	1.14E-15	2.75E-14
CLU	1.184622285	7.799858785	8.277288225	1.20E-15	2.88E-14
GAL3ST2	-1.567272032	-2.977126883	-8.276439605	1.20E-15	2.90E-14
AGR3	1.773634823	4.661968524	8.269428398	1.27E-15	3.05E-14
PARM1	1.055415953	6.96151651	8.264962024	1.31E-15	3.14E-14
MALRD1	1.347635843	-0.277296995	8.26287635	1.33E-15	3.18E-14
ANKFN1	1.556140777	-0.081891103	8.262525973	1.33E-15	3.19E-14
GRIN2A	1.95638783	-1.721041536	8.25195274	1.44E-15	3.43E-14
CLIC5	1.240918702	4.059386848	8.251652883	1.44E-15	3.43E-14
RETNLB	-1.384501472	-4.848785193	-8.238810135	1.59E-15	3.76E-14
ATP13A4	1.60656192	3.868402276	8.231873154	1.67E-15	3.93E-14
S100B	1.261272431	2.338261518	8.222323798	1.79E-15	4.20E-14
RP11_551L14.1	-1.337547031	-1.589363134	-8.211670068	1.94E-15	4.51E-14
AC067959.1	1.390588823	-5.002183887	8.207763895	1.99E-15	4.63E-14
TMEM232	1.247238076	-0.524210599	8.203260755	2.06E-15	4.78E-14
AQP3	1.340382722	8.073711673	8.202113316	2.08E-15	4.82E-14

CTD_2008P7.8	-2.01065014	-3.618778625	-8.193939618	2.20E-15	5.10E-14
GGH	-1.031768761	4.152342634	-8.181884106	2.41E-15	5.55E-14
C1orf186	1.102278272	1.03040873	8.181144585	2.42E-15	5.57E-14
ADAM12	-1.203898736	3.977773562	-8.180826057	2.43E-15	5.58E-14
WFDC2	1.164106757	7.906648476	8.179910351	2.44E-15	5.61E-14
RP11_626G11.4	-1.150548688	-1.667173286	-8.173866373	2.55E-15	5.85E-14
NDNF	1.367828843	5.175074219	8.170660952	2.61E-15	5.99E-14
ST3GAL5_AS1	1.04152037	-1.502928347	8.166454375	2.69E-15	6.15E-14
BX842568.4	1.146271488	-4.831893677	8.149289771	3.05E-15	6.93E-14
AQP1	1.276684193	7.516265841	8.148357158	3.07E-15	6.97E-14
CLIC3	1.141362834	3.406400423	8.123830143	3.67E-15	8.25E-14
GATA5	1.175109457	-0.922107116	8.123519775	3.68E-15	8.26E-14
RP11_95I16.6	1.696178654	-4.149085357	8.123383898	3.69E-15	8.26E-14
C18orf63	1.337428413	-4.619003963	8.120091201	3.78E-15	8.44E-14
CDKL2	1.151559578	2.611674988	8.116480168	3.88E-15	8.66E-14
ESYT3	1.03328808	3.524114494	8.108104145	4.12E-15	9.16E-14
CPA3	1.257800329	3.7111298	8.104330563	4.23E-15	9.39E-14
C1orf132	1.106081132	2.248817397	8.097680533	4.44E-15	9.82E-14
RXFP2	1.326602323	-4.910750505	8.084658784	4.88E-15	1.07E-13
SEC14L6	1.271543562	1.706307219	8.081901311	4.98E-15	1.09E-13
WIF1	2.225047587	1.877587501	8.081865058	4.98E-15	1.09E-13
ENAM	1.50812879	-0.745821969	8.075746176	5.21E-15	1.14E-13
ALOX15B	1.461332796	4.761690241	8.069520151	5.45E-15	1.19E-13
PENK	1.686562247	-0.846584559	8.067481261	5.53E-15	1.20E-13
HPGD	1.510759638	5.42561356	8.067446173	5.53E-15	1.20E-13
CRTAC1	1.570308227	3.37986298	8.0607516	5.80E-15	1.26E-13
RP5_856G1.2	1.005278425	-5.157420163	8.055901056	6.01E-15	1.30E-13
RPLP0P2	-1.075464723	1.779949847	-8.055307456	6.04E-15	1.31E-13
FPGT_TNNI3K	1.028610111	-1.375665382	8.053539728	6.12E-15	1.32E-13
FENDRR	1.241850935	1.022135318	8.043978178	6.55E-15	1.41E-13
OR2B6	-1.287573886	-3.395835992	-8.033202776	7.08E-15	1.52E-13
RP11_655M14.4	1.189977805	-4.761002661	8.031821263	7.15E-15	1.53E-13
SPTLC3	1.082748822	3.339391443	8.028425809	7.33E-15	1.57E-13
FSD1	-1.157250795	-1.374188872	-8.026511631	7.43E-15	1.59E-13
LMO3	1.43429541	5.886961607	8.018903435	7.85E-15	1.67E-13
SOSTDC1	1.864017594	-0.857215864	8.018788496	7.86E-15	1.67E-13
SFTPC	3.01912708	6.119276134	8.004731527	8.69E-15	1.83E-13
GPR37L1	-1.015672723	-1.1550376	-8.001647355	8.89E-15	1.87E-13
RP5_1057J7.7	1.11604951	-1.254759571	7.999096882	9.05E-15	1.90E-13
SFTA3	1.558523743	5.67854165	7.993008663	9.46E-15	1.98E-13
RP13_514E23.1	1.078313543	-3.206037857	7.992807233	9.47E-15	1.98E-13
GGT6	1.767082438	2.273564206	7.98461468	1.00E-14	2.09E-13
PIFO	1.13421516	3.175248479	7.97081477	1.11E-14	2.30E-13
FLRT3	1.146694168	4.706359707	7.965305813	1.15E-14	2.39E-13
KCNA5	1.004664574	-1.299739248	7.953126196	1.26E-14	2.59E-13
GBX2	-1.45256402	-4.143121893	-7.948428085	1.30E-14	2.68E-13
GLOD5	1.0294147	-0.076039119	7.94616136	1.32E-14	2.71E-13
STRIP2	-1.177660704	2.560096372	-7.935897077	1.42E-14	2.91E-13
C14orf180	1.182811477	-4.68185804	7.933257186	1.45E-14	2.96E-13
FAM57B	-1.118566355	-2.424959881	-7.931128898	1.47E-14	3.00E-13
SLC8A3	1.053924947	-1.839768419	7.928829585	1.50E-14	3.05E-13
ARTN	-1.111175922	0.387529391	-7.927767572	1.51E-14	3.07E-13
CCR6	1.119131633	-2.233441457	7.92236163	1.57E-14	3.19E-13
MYH16	-1.135983946	-1.170754745	-7.915611066	1.65E-14	3.33E-13

HIST1H2BO	-1.317035566	-3.154479468	-7.911180602	1.70E-14	3.43E-13
SLC46A2	1.48830641	0.928794094	7.910728175	1.70E-14	3.44E-13
FAM107A	1.078093506	2.607327042	7.908629787	1.73E-14	3.49E-13
MGC27382	1.491336037	-3.028077656	7.901501095	1.82E-14	3.66E-13
FOXA2	1.660511231	3.695015628	7.89389924	1.92E-14	3.85E-13
RP11_528A4.2	1.580869837	-1.005005803	7.893103674	1.93E-14	3.87E-13
STC2	-1.082884839	3.496591176	-7.886969005	2.02E-14	4.03E-13
HLA_DQB2	1.258138579	5.190836692	7.880637353	2.11E-14	4.20E-13
ANKRD18B	-1.774765283	-1.441464669	-7.879120849	2.13E-14	4.25E-13
RP11_191L9.4	-1.690486623	-4.666776013	-7.867830784	2.31E-14	4.57E-13
MS4A2	1.203798117	1.255850608	7.864614161	2.36E-14	4.67E-13
UCN2	-1.425508387	-2.731314955	-7.848101011	2.66E-14	5.21E-13
ALPL	1.406593467	5.586727065	7.842804106	2.76E-14	5.39E-13
DUOXA1	1.276790325	2.844205529	7.83479419	2.92E-14	5.70E-13
AC005077.14	-1.410055989	0.82505267	-7.832269842	2.97E-14	5.78E-13
CMA1	1.187279051	-3.322113086	7.82992054	3.02E-14	5.87E-13
DPCR1	1.831471423	1.162819195	7.829222944	3.04E-14	5.89E-13
ATOH8	1.335258384	3.772222073	7.828068785	3.06E-14	5.94E-13
AC008268.1	2.290346898	-0.716907571	7.826100633	3.11E-14	6.01E-13
CHIAP2	2.03913614	-2.774589977	7.824555708	3.14E-14	6.07E-13
LINC01354	1.074434503	-3.008428888	7.814326014	3.38E-14	6.51E-13
ROS1	1.469199768	5.10652089	7.803993631	3.63E-14	6.98E-13
TMEM252	1.173365469	-3.692100363	7.800838698	3.71E-14	7.12E-13
PTCHD4	1.283896423	-0.29973758	7.798321281	3.78E-14	7.25E-13
FGF10	1.338388643	-2.808234311	7.796729154	3.82E-14	7.32E-13
CTD_2116N20.1	-1.006059096	-3.766562272	-7.796167774	3.84E-14	7.34E-13
AC003090.1	1.414580393	-2.21158771	7.795165736	3.86E-14	7.39E-13
RGS22	1.20961544	-0.231220642	7.794703301	3.88E-14	7.40E-13
CAPN8	1.384385415	5.610309345	7.785370895	4.14E-14	7.88E-13
LINC00892	1.031123679	-1.447648689	7.783474977	4.20E-14	7.98E-13
RP11_28001.2	1.761694181	-2.334999676	7.767083376	4.71E-14	8.90E-13
NT5DC4	-1.066789622	-2.304623732	-7.752224998	5.23E-14	9.81E-13
CADM3_AS1	1.278962369	-2.12829941	7.749401669	5.33E-14	9.99E-13
AC092071.1	1.715010685	-1.619335381	7.748100752	5.38E-14	1.01E-12
PTGDS	1.018668419	5.35738119	7.742413079	5.60E-14	1.04E-12
DNASE2B	1.319395844	-1.904070577	7.736268156	5.85E-14	1.09E-12
IGF2BP1	-2.572354229	-1.042992577	-7.733676382	5.95E-14	1.11E-12
LINC01550	1.186178283	0.005673052	7.732274883	6.01E-14	1.12E-12
OCA2	1.52418549	-1.577912461	7.731352762	6.05E-14	1.12E-12
GAL	-1.872653492	-1.251005819	-7.699307555	7.57E-14	1.39E-12
MAGEA3	-3.109949371	-2.850286661	-7.667889028	9.42E-14	1.71E-12
COL7A1	-1.282167206	3.573061457	-7.667280281	9.46E-14	1.71E-12
PGM5P3_AS1	1.207925382	-4.034897051	7.667004953	9.48E-14	1.71E-12
DLX2	-1.185658718	-4.855705155	-7.657843586	1.01E-13	1.82E-12
HMGCLL1	1.090884159	-0.654637271	7.653410428	1.04E-13	1.87E-12
FOXQ1	1.581160706	3.155578775	7.651247967	1.06E-13	1.90E-12
CDH18	-1.799360632	-4.315878784	-7.649978024	1.07E-13	1.91E-12
CNTN6	1.52485836	-1.057253166	7.638099835	1.16E-13	2.07E-12
LGI3	1.950052184	-0.41069174	7.608742971	1.42E-13	2.52E-12
CLDN18	2.273034093	3.032863183	7.60518647	1.46E-13	2.58E-12
RXRG	1.4624856	-1.233738362	7.603023726	1.48E-13	2.62E-12
MEOX1	1.020786716	-0.578127907	7.601216641	1.50E-13	2.65E-12
RP11_20J15.5	1.130218592	-4.182572772	7.598067075	1.53E-13	2.69E-12
BCHE	1.040582033	0.511814037	7.595093136	1.56E-13	2.74E-12

GLDC	-1.383296886	0.922565115	-7.594833116	1.56E-13	2.75E-12
CTD_3032H12.1	1.391419728	-3.743735889	7.591584857	1.60E-13	2.80E-12
LL22NC03_75H12.2	1.637784431	-1.53716159	7.590261408	1.61E-13	2.83E-12
GPR115	-1.758533681	0.977998971	-7.582338656	1.71E-13	2.97E-12
SHE	1.092431947	4.013692115	7.574412883	1.80E-13	3.13E-12
C1orf61	-1.814739012	-0.806546359	-7.570877331	1.85E-13	3.20E-12
GPD1	1.137236681	1.164243427	7.562815271	1.95E-13	3.37E-12
CYCSP6	-1.692039115	-4.330332484	-7.558924196	2.00E-13	3.46E-12
SYNGR4	-1.246044096	-2.42029214	-7.557710078	2.02E-13	3.48E-12
TYRP1	1.207995853	0.217201153	7.548038162	2.16E-13	3.71E-12
AC005592.1	1.297626076	-3.01137789	7.547379438	2.17E-13	3.72E-12
ZBTB7C	1.124691211	2.978280732	7.547303632	2.17E-13	3.72E-12
LINC01234	-1.966208563	-3.414960027	-7.544659313	2.21E-13	3.78E-12
LRRC36	1.165747944	0.556593504	7.543590896	2.23E-13	3.81E-12
FAM216B	1.829778269	0.089296247	7.540192926	2.28E-13	3.89E-12
NME5	1.005410728	1.044339958	7.540190119	2.28E-13	3.89E-12
RTN4RL1	1.314062079	1.02245171	7.538895629	2.30E-13	3.92E-12
SLC16A11	1.002602134	0.580478507	7.537470747	2.32E-13	3.96E-12
ABCA3	1.189407765	7.061268775	7.532281295	2.41E-13	4.09E-12
SV2A	-1.052206224	2.267603592	-7.531305592	2.42E-13	4.11E-12
MUSK	1.110822652	-2.513411499	7.528142571	2.48E-13	4.20E-12
RP11_643A5.3	1.388188834	-4.217144528	7.527814583	2.48E-13	4.20E-12
AR	1.031774222	2.33427407	7.525927213	2.51E-13	4.26E-12
SP8	-2.316413884	-2.152210322	-7.522378715	2.58E-13	4.35E-12
C2orf71	1.429496899	-3.587675057	7.518782599	2.64E-13	4.45E-12
MYH2	1.149872485	-3.697302028	7.51724147	2.67E-13	4.49E-12
FAM189A2	1.058113379	2.817475397	7.509188884	2.82E-13	4.73E-12
RN7SKP18	1.047840739	-4.843446992	7.509124115	2.82E-13	4.73E-12
HBB	1.357308918	3.915792421	7.507443412	2.85E-13	4.78E-12
MAGEE2	1.372370003	-3.258870733	7.50496355	2.90E-13	4.85E-12
DYNLRB2	1.362970547	-1.11221483	7.500454088	2.99E-13	4.99E-12
SCARA5	1.563294632	0.683256352	7.498782716	3.03E-13	5.05E-12
RP11_525G13.2	-1.042920865	-2.863761682	-7.495444532	3.10E-13	5.15E-12
MT1H	-1.357028379	-1.601382272	-7.490876985	3.20E-13	5.31E-12
B3GALT2	1.172969488	0.053692421	7.488528729	3.25E-13	5.38E-12
SOHLH1	-1.390588733	-4.938056025	-7.485551619	3.32E-13	5.49E-12
AC016735.2	1.2870692	-4.268096046	7.479296277	3.46E-13	5.71E-12
ANKRD29	1.12998178	3.14826465	7.476340823	3.53E-13	5.82E-12
COL11A1	-1.900769896	4.048970734	-7.473825573	3.59E-13	5.92E-12
AC013275.2	1.569142428	-1.121355495	7.470926064	3.66E-13	6.02E-12
RP11_476D10.1	1.737224976	-1.690841918	7.46744369	3.75E-13	6.16E-12
CNTFR	1.34349314	-2.577500172	7.46732715	3.76E-13	6.16E-12
SPINK5	1.548237189	2.720118312	7.461669142	3.90E-13	6.38E-12
HLA_DRB5	1.038835101	7.553857102	7.458823373	3.98E-13	6.49E-12
RP11_357P18.2	1.011216995	-0.746817879	7.454817835	4.09E-13	6.66E-12
KCNA4	1.272780667	-3.808150308	7.446424634	4.33E-13	7.02E-12
PCDH15	1.509214886	-2.601687738	7.446046239	4.34E-13	7.04E-12
FLJ39080	-1.596540066	-3.987964539	-7.440586625	4.51E-13	7.28E-12
ACADL	1.412886603	0.419243656	7.436651888	4.63E-13	7.47E-12
CTD_2006K23.2	1.213597184	-5.077100928	7.430146654	4.84E-13	7.78E-12
SLC22A31	1.503556677	5.654829857	7.426995527	4.94E-13	7.93E-12
CTD_2292M14.1	1.648846829	-3.707014039	7.422243213	5.10E-13	8.17E-12
PPY2	-1.426758228	-4.837519405	-7.421976964	5.11E-13	8.18E-12
AGRP	1.029504379	-1.987319348	7.420282105	5.17E-13	8.27E-12

RSPO1	1.150833984	-1.60549433	7.407264195	5.65E-13	9.00E-12
C9orf152	1.178960956	3.795953207	7.395996316	6.10E-13	9.67E-12
CTB_43E15.2	1.271438469	-2.948115321	7.394672172	6.15E-13	9.75E-12
CH25H	1.100969089	2.632377705	7.391887424	6.27E-13	9.92E-12
LINC00887	-1.322155889	-2.351668558	-7.387753666	6.45E-13	1.02E-11
RP5_1007F24.1	1.050579347	-2.674818605	7.387162495	6.47E-13	1.02E-11
PACSIN1	-1.010189257	0.57212059	-7.381662675	6.72E-13	1.06E-11
CTB_43E15.1	1.150815827	-4.293852748	7.380443837	6.78E-13	1.06E-11
C10orf107	1.331515878	-0.216703325	7.372646352	7.14E-13	1.12E-11
GJB1	1.950741832	2.343302746	7.36975258	7.28E-13	1.14E-11
OR51E1	-1.330557923	-1.547075012	-7.366219645	7.46E-13	1.16E-11
DUOX1	1.125721718	4.397152499	7.36238438	7.65E-13	1.19E-11
SOX8	1.085302139	-0.759784062	7.362035002	7.67E-13	1.19E-11
CNR1	1.144164529	1.474733826	7.359631956	7.80E-13	1.21E-11
YBX2	-1.700311908	0.856180162	-7.358874581	7.84E-13	1.22E-11
RP11_1069G10.2	-1.11311104	-4.258543499	-7.353627111	8.12E-13	1.26E-11
TMEM132D	1.921731072	-1.386398911	7.351969851	8.21E-13	1.27E-11
KLHL31	-1.012963052	-0.391252339	-7.351134058	8.26E-13	1.28E-11
COCH	-1.386382152	1.375955226	-7.342972712	8.72E-13	1.34E-11
RIC3	1.210558829	0.53659794	7.340842321	8.85E-13	1.36E-11
RASGRF1	1.354116448	2.649436014	7.340418113	8.88E-13	1.37E-11
PTPRN	-1.710126094	-0.672433597	-7.32889708	9.59E-13	1.47E-11
LL22NC03_N64E9.1	-1.052567873	-4.838863918	-7.32319206	9.97E-13	1.52E-11
NKAIN1	-1.406059554	-1.846611893	-7.322459093	1.00E-12	1.53E-11
LINC00665	-1.108011846	3.755531241	-7.320056277	1.02E-12	1.55E-11
RS1	1.115092968	-2.651344646	7.317779446	1.03E-12	1.57E-11
RP11_190J1.3	-1.655281305	-4.082980551	-7.317056894	1.04E-12	1.58E-11
BMP3	1.456884682	3.443142019	7.309600688	1.09E-12	1.65E-11
ANO5	1.17739526	1.221429911	7.30894824	1.10E-12	1.66E-11
GRIN2D	-1.17813036	1.708486455	-7.30578415	1.12E-12	1.69E-11
RP11_641D5.2	1.075281549	-3.655320215	7.302113497	1.15E-12	1.73E-11
AL589743.1	-1.378745446	-2.459263248	-7.297758122	1.18E-12	1.78E-11
CTD_2531D15.4	1.128701058	-4.221352793	7.294914101	1.20E-12	1.81E-11
SLC34A2	1.322061879	10.01219888	7.294791489	1.21E-12	1.81E-11
FCGBP	1.310500598	5.055718255	7.267060209	1.45E-12	2.16E-11
ERVV_2	-1.435934267	-4.96650503	-7.266386631	1.46E-12	2.17E-11
TPSAB1	1.050752631	3.140228466	7.265782089	1.46E-12	2.18E-11
STEAP1B	-1.152157145	-0.593905668	-7.263174917	1.49E-12	2.21E-11
RP11_203P23.2	1.027649396	-5.498882516	7.256059822	1.56E-12	2.31E-11
SLC16A4	1.090940274	4.310890934	7.255997968	1.56E-12	2.31E-11
SMCO2	-1.055857688	-2.287528128	-7.249780461	1.63E-12	2.40E-11
AC064834.3	1.036824062	-4.347290592	7.244740851	1.69E-12	2.48E-11
TPSB2	1.21940125	3.207252256	7.239930433	1.74E-12	2.56E-11
SEC14L3	1.500944309	-3.177416796	7.23751345	1.77E-12	2.60E-11
RP11_829H16.3	-1.110764488	-3.578993511	-7.232611566	1.83E-12	2.68E-11
LEMD1	1.3075397	0.849004072	7.22670007	1.90E-12	2.78E-11
RN7SL8P	1.341128011	-3.799019821	7.215254094	2.05E-12	2.99E-11
ADRB3	1.164834406	-3.280091228	7.200283614	2.26E-12	3.29E-11
C1orf105	-1.092857258	-4.087321346	-7.192192424	2.39E-12	3.46E-11
WBSCR17	1.073081525	1.472716236	7.191621554	2.40E-12	3.47E-11
NT5C1A	1.416625869	-3.720954239	7.191215951	2.41E-12	3.48E-11
SLC44A4	1.03361136	6.543756945	7.187872287	2.46E-12	3.56E-11
DUSP5P1	-1.334178517	-3.047890173	-7.172891566	2.72E-12	3.91E-11
LINC00332	1.201114727	-5.158846961	7.162186174	2.91E-12	4.19E-11

RP11_401P9.4	1.049432931	0.73860008	7.154464721	3.07E-12	4.40E-11
PLAC1	-1.44841037	-1.73189998	-7.148604255	3.19E-12	4.56E-11
FTCD	-1.305291298	-2.097291885	-7.144055161	3.28E-12	4.69E-11
GFY	-1.58021938	-4.216868079	-7.141053595	3.35E-12	4.77E-11
CASR	1.793388508	-3.144995236	7.140063089	3.37E-12	4.80E-11
RP5_831C21.1	1.187088359	-4.619164184	7.135420979	3.48E-12	4.95E-11
HOXD13	-1.71431568	-4.859638156	-7.128093985	3.65E-12	5.18E-11
C4BPA	1.590372491	6.202898358	7.110864967	4.09E-12	5.76E-11
ERBB4	1.287877998	0.405434493	7.108051211	4.16E-12	5.86E-11
HNF1B	1.116770169	4.276192157	7.102725869	4.31E-12	6.06E-11
AGER	1.339572896	4.460704109	7.100705311	4.37E-12	6.14E-11
LPL	1.14557534	4.599063312	7.098254241	4.44E-12	6.24E-11
LINC00355	-1.851013061	-4.088487283	-7.088860943	4.72E-12	6.61E-11
LINC00551	1.089071424	-2.732614959	7.088138773	4.74E-12	6.64E-11
LINC01305	-1.283350583	-4.791420032	-7.085429451	4.83E-12	6.74E-11
RP11_103J17.2	-1.63028227	-4.858315753	-7.085340234	4.83E-12	6.74E-11
PDE4C	1.007122533	2.199333168	7.082413326	4.93E-12	6.86E-11
C5orf49	1.2254451	1.171842221	7.078775093	5.04E-12	7.01E-11
RP11_417L19.2	-1.121323567	-4.678643276	-7.070783943	5.32E-12	7.37E-11
SYBU	1.048211873	3.817265197	7.070499826	5.33E-12	7.38E-11
HSD17B13	1.212168172	-0.512021554	7.06338036	5.58E-12	7.72E-11
CTD_2385L22.1	1.210352766	-4.976769463	7.062690489	5.60E-12	7.75E-11
PRSS12	1.257380949	2.627928085	7.062440698	5.61E-12	7.76E-11
NKX1_2	-2.03762041	-2.150544538	-7.060627101	5.68E-12	7.84E-11
CFAP57	1.105625992	1.093333611	7.052292658	6.00E-12	8.24E-11
SLC6A17	-1.358396184	-1.574891556	-7.051644218	6.02E-12	8.27E-11
KRT27	1.215901404	-3.65888403	7.05156622	6.03E-12	8.27E-11
CHRNA9	-1.989177716	-1.996021186	-7.041967772	6.42E-12	8.76E-11
KNDC1	1.328321608	2.447918897	7.02941213	6.96E-12	9.46E-11
C17orf50	1.133638831	-2.838618194	7.027967913	7.03E-12	9.53E-11
MYBPHL	1.676276185	-0.014801456	7.02369624	7.23E-12	9.79E-11
CTD_2162K18.4	-1.299692905	-2.545252499	-7.02060135	7.37E-12	9.98E-11
TBR1	-1.240509782	-4.564167877	-7.018469982	7.48E-12	1.01E-10
TNXB	1.001493124	3.637706341	7.01057617	7.87E-12	1.06E-10
CTD_2357A8.3	-1.214292146	-0.669647726	-7.010214215	7.89E-12	1.06E-10
SORCS2	1.16122692	3.523073107	7.007225667	8.04E-12	1.08E-10
RP11_402G3.3	-1.142424134	-3.956413453	-7.000199065	8.42E-12	1.13E-10
C12orf56	-1.948536028	-1.468275155	-6.99922978	8.47E-12	1.14E-10
ATP13A4_AS1	1.558973714	-2.885395986	6.99834477	8.52E-12	1.14E-10
AC005592.3	1.076807178	-4.045540082	6.993895995	8.77E-12	1.17E-10
DRD1	1.449738818	-1.376669063	6.99139234	8.91E-12	1.19E-10
ADAMTS20	-1.593271344	-4.202019817	-6.989420553	9.03E-12	1.20E-10
RP11_439M11.1	-1.004044266	-3.598256024	-6.976455454	9.82E-12	1.31E-10
TSPAN7	1.04133635	3.606677732	6.975705911	9.87E-12	1.31E-10
RP11_599J14.2	-1.0247189	-1.446942936	-6.97038008	1.02E-11	1.35E-10
RP11_805I24.3	1.031482511	-4.295832287	6.966010037	1.05E-11	1.39E-10
HAP1	-1.150023996	-1.117598408	-6.963465428	1.07E-11	1.41E-10
SYNDIG1L	1.190973393	-1.581143014	6.952136197	1.15E-11	1.51E-10
MAGEA6	-2.742634191	-3.292405003	-6.946998385	1.19E-11	1.56E-10
ADRA2A	1.211280217	2.609460965	6.944019539	1.21E-11	1.59E-10
FAM81B	1.290557509	0.578264595	6.942423845	1.22E-11	1.60E-10
IGJ	1.122067575	8.255950571	6.941110403	1.23E-11	1.61E-10
OMG	1.069499377	0.456967522	6.935952758	1.28E-11	1.66E-10
SEMA3A	-1.229218263	3.436499031	-6.934018556	1.29E-11	1.68E-10

AC005077.12	-1.04490225	-0.996972717	-6.93241314	1.31E-11	1.70E-10
PHGDH	-1.032472003	4.1652574	-6.929864868	1.33E-11	1.73E-10
P3H2	1.081983802	3.909962128	6.922470507	1.39E-11	1.81E-10
DIO3OS	1.258878629	-0.241750821	6.912732566	1.48E-11	1.91E-10
CYP4A11	1.073378632	-4.318480674	6.911605936	1.49E-11	1.93E-10
CRYM	1.486859151	3.062625201	6.908142018	1.53E-11	1.97E-10
CTD_3006G17.2	-1.243301911	-5.213046626	-6.903540217	1.57E-11	2.02E-10
HSPB3	1.011111995	-3.380217881	6.898603974	1.62E-11	2.09E-10
RP11_501C14.7	-1.116518962	-5.094671086	-6.89658097	1.64E-11	2.11E-10
CNTN3	1.177907691	0.59248738	6.888109807	1.73E-11	2.22E-10
SCGB1A1	2.530887551	3.127306625	6.88716791	1.75E-11	2.23E-10
RP11_539E17.5	-1.056229224	-2.646512883	-6.887046156	1.75E-11	2.23E-10
DUXAP10	-1.18560361	-2.137988111	-6.887019231	1.75E-11	2.23E-10
CLUL1	1.045265603	0.236737463	6.886059865	1.76E-11	2.24E-10
PLCXD3	1.270730013	0.155261048	6.884377358	1.78E-11	2.26E-10
PTPRT	1.699088649	-0.713530369	6.875663922	1.88E-11	2.38E-10
MYOZ1	1.003895553	0.096681271	6.874549397	1.89E-11	2.40E-10
VEPH1	1.111401727	2.809695591	6.867806284	1.98E-11	2.49E-10
ST8SIA6_AS1	-1.798740402	-3.71320707	-6.864427811	2.02E-11	2.54E-10
IRX5	1.129680034	3.262565096	6.854924802	2.15E-11	2.69E-10
CSAG2	-1.575202826	-4.983548617	-6.842474701	2.32E-11	2.90E-10
C1QL1	-1.174316816	-2.21218107	-6.839746301	2.36E-11	2.94E-10
KIAA0408	1.135483214	-2.84350635	6.83782433	2.39E-11	2.98E-10
CPAMD8	1.112287758	3.867778186	6.834753595	2.44E-11	3.03E-10
DPPA3P2	1.275668058	-3.289980774	6.830416282	2.51E-11	3.11E-10
IRX1	1.729397156	-1.372984231	6.826754156	2.57E-11	3.18E-10
LUCAT1	-1.353918016	0.628166789	-6.826062623	2.58E-11	3.19E-10
HOXA11_AS	-1.403123624	-3.951400894	-6.814328061	2.78E-11	3.41E-10
HOXD1	1.409046111	0.460925087	6.810259788	2.85E-11	3.50E-10
RP11_896J10.3	1.123596113	-1.734995119	6.80277787	2.99E-11	3.66E-10
ADAMTS7P3	1.196385786	-1.497321755	6.788122316	3.28E-11	3.99E-10
RN7SKP51	1.198359411	-3.171213557	6.78057788	3.44E-11	4.17E-10
TMEM130	1.180717072	2.764219853	6.774840563	3.57E-11	4.31E-10
CTA_384D8.31	-1.146456235	-2.525373238	-6.770956115	3.66E-11	4.42E-10
TNNC1	1.031046406	1.863109626	6.765921997	3.78E-11	4.55E-10
RP1_78O14.1	1.100965892	-0.864101502	6.76433866	3.81E-11	4.60E-10
IGSF9B	1.222976304	0.769197808	6.763786494	3.83E-11	4.61E-10
RP11_164O23.8	1.087850668	-3.261902336	6.763348808	3.84E-11	4.62E-10
LRRC52	1.383757495	-3.373804846	6.762767544	3.85E-11	4.63E-10
LINC01194	-1.791921041	-4.492609121	-6.755862523	4.02E-11	4.81E-10
LHFPL3	1.460021767	-2.085983717	6.754108698	4.07E-11	4.87E-10
RP11_219G17.4	-1.101843835	-1.948996342	-6.752841563	4.10E-11	4.90E-10
RP11_561N12.5	-1.117811946	-5.001381115	-6.752445539	4.11E-11	4.91E-10
RP11_336K24.5	1.219847735	-3.363138368	6.752229392	4.12E-11	4.91E-10
CYP4F26P	-1.334719919	-3.553462513	-6.750673803	4.16E-11	4.95E-10
LL22NC03_N14H11.1	-1.117887884	-3.036700597	-6.750632098	4.16E-11	4.95E-10
SP9	-1.362778426	-5.150404465	-6.746095094	4.28E-11	5.08E-10
FER1L4	-1.083178334	3.323702777	-6.737914794	4.51E-11	5.32E-10
TMEM145	-1.078812736	-0.913647184	-6.737511453	4.52E-11	5.33E-10
CALML3_AS1	-1.266005911	-1.834775444	-6.737004032	4.53E-11	5.35E-10
SNX18P7	-1.1969905	-4.266907364	-6.727590032	4.81E-11	5.65E-10
FOXI2	1.116725339	-3.935874612	6.723214003	4.94E-11	5.80E-10
PRMT8	1.598122753	-1.232522098	6.722725344	4.96E-11	5.81E-10
KIF6	1.018494478	-0.043313784	6.716488628	5.16E-11	6.03E-10

CTD_2561B21.11	1.092805629	-1.377848406	6.714084277	5.23E-11	6.11E-10
KCP	-1.042466938	1.059136788	-6.702417155	5.63E-11	6.55E-10
CTD_3035D6.2	-1.081047399	-2.939030611	-6.697674115	5.80E-11	6.72E-10
HIST1H3J	-1.068262522	-3.772728212	-6.695789551	5.87E-11	6.80E-10
APOD	1.220572263	4.505489191	6.693359668	5.96E-11	6.89E-10
ADCY2	1.092778617	-0.735106137	6.693287483	5.96E-11	6.89E-10
C6	1.5282319	-0.208778624	6.691208694	6.04E-11	6.97E-10
BAI3	1.01180577	-1.129919495	6.688854093	6.13E-11	7.07E-10
SHISA3	1.69324128	1.179977321	6.688233511	6.15E-11	7.09E-10
LANCL3	1.148353505	-0.147049999	6.681548824	6.42E-11	7.37E-10
C1orf87	1.676040243	-1.989108036	6.681257853	6.43E-11	7.38E-10
RP11_1C8.4	-1.423362946	-4.567751287	-6.673495727	6.75E-11	7.72E-10
NTRK2	1.102192171	1.17836759	6.667485368	7.01E-11	7.98E-10
BRINP3	1.029332104	-1.461271285	6.662328836	7.24E-11	8.23E-10
GPM6A	1.253183941	-0.213945783	6.656627983	7.50E-11	8.51E-10
HOXD11	-1.433804431	-4.922299475	-6.651568316	7.74E-11	8.76E-10
FCER2	1.156190493	-1.110507131	6.644829926	8.07E-11	9.11E-10
HOXB9	-2.364812955	-0.915072002	-6.642637744	8.18E-11	9.22E-10
PCMTD1P3	-1.08882831	-3.05870854	-6.641934741	8.22E-11	9.25E-10
TDRD12	-1.226078209	-1.943788649	-6.638094851	8.42E-11	9.46E-10
HHIP	1.559157462	1.843280235	6.626425327	9.05E-11	1.01E-09
MAGEC2	-2.08957993	-3.998574899	-6.607140337	1.02E-10	1.13E-09
CFTR	1.366559006	2.787654557	6.591005349	1.13E-10	1.24E-09
B4GALNT4	-1.478060188	2.05772232	-6.586113648	1.16E-10	1.28E-09
LINC01213	-1.08939009	-4.658542569	-6.582258486	1.19E-10	1.31E-09
CSAG1	-2.183859905	-3.234565448	-6.58220672	1.19E-10	1.31E-09
RP11_476K15.1	-1.473248582	-4.664647693	-6.571198218	1.27E-10	1.39E-09
AP001626.1	1.27600806	0.513816104	6.56782738	1.30E-10	1.42E-09
SERPINA1	1.095124967	8.962017292	6.560887723	1.36E-10	1.48E-09
RP11_215P8.4	-1.138264677	-4.749903929	-6.556842579	1.39E-10	1.51E-09
RIPPLY3	-1.263975737	-0.270007985	-6.551310874	1.44E-10	1.56E-09
CCL26	-1.091379749	-1.864190563	-6.545587155	1.49E-10	1.61E-09
LHX2	-1.272160828	-2.432374429	-6.545461951	1.49E-10	1.61E-09
CSAG3	-1.642986639	-4.632620874	-6.542427708	1.52E-10	1.64E-09
RP11_758M4.4	-1.471176516	-4.489685128	-6.540243873	1.54E-10	1.66E-09
LINC01446	-1.702706969	-4.411843302	-6.536425089	1.58E-10	1.70E-09
LSINCT5	1.147315873	-4.555362992	6.536338545	1.58E-10	1.70E-09
ADAM2	-1.339883117	-4.937390908	-6.536015716	1.58E-10	1.70E-09
AC133785.1	-1.440950671	-2.364132903	-6.533155176	1.61E-10	1.73E-09
SMC1B	-1.35765129	-0.635593602	-6.530935903	1.63E-10	1.75E-09
RP1_86C11.7	-1.028729992	-1.938320859	-6.530285783	1.64E-10	1.76E-09
CYP4X1	1.025594723	2.877758833	6.528551583	1.66E-10	1.78E-09
RP11_775D22.2	1.026024612	-2.262452655	6.527570361	1.67E-10	1.78E-09
POU4F1	-1.672182719	-2.386875537	-6.519023446	1.76E-10	1.88E-09
LHFPL3_AS1	1.078471385	-4.611445315	6.515666525	1.79E-10	1.91E-09
STRA6	-1.268233166	2.663398512	-6.509438794	1.86E-10	1.98E-09
AC092667.2	1.022403234	-4.820062885	6.504665273	1.92E-10	2.04E-09
ENPP7P11	1.065918537	-4.045002388	6.503961253	1.93E-10	2.04E-09
RP11_85G21.3	1.313376336	-3.258304494	6.499862836	1.98E-10	2.09E-09
GPR12	1.475721117	-4.113606911	6.492863042	2.06E-10	2.17E-09
HIST1H1D	-1.311437148	-2.826000888	-6.489500741	2.11E-10	2.22E-09
RSPO4	1.153242056	-0.060054761	6.486666238	2.14E-10	2.25E-09
GREB1L	-1.183255484	0.957866128	-6.486661125	2.14E-10	2.25E-09
HIST1H2BH	-1.321808063	-2.250518448	-6.484601995	2.17E-10	2.28E-09

KLK2	-1.142837499	-4.55811865	-6.4836334	2.18E-10	2.29E-09
RAD17P1	1.195019061	-2.651710829	6.479329002	2.24E-10	2.35E-09
CTA_280A3.2	-1.08098108	-5.203170486	-6.478994083	2.24E-10	2.35E-09
CHI3L2	1.022432966	3.112241899	6.477626485	2.26E-10	2.37E-09
RP11_10A14.4	-1.066353623	-1.678215155	-6.475855172	2.29E-10	2.39E-09
TRPM2_AS	-1.501808071	-1.589827422	-6.474527582	2.31E-10	2.41E-09
RP11_243M5.3	1.060024076	-4.00060654	6.464902366	2.45E-10	2.54E-09
ZSCAN4	1.325310361	-3.128226071	6.459921233	2.52E-10	2.61E-09
KCND2	-1.067250957	0.655851503	-6.458874177	2.54E-10	2.63E-09
RP11_863P13.3	-1.009744601	-2.336222918	-6.447596682	2.72E-10	2.80E-09
RP11_66N24.6	1.525900928	0.265233916	6.444299772	2.77E-10	2.85E-09
AZU1	1.112577662	-2.090278627	6.442326625	2.81E-10	2.89E-09
RP11_149I9.2	1.034102799	-4.274267433	6.441391418	2.82E-10	2.90E-09
RP11_206P5.2	1.384601726	-1.265669744	6.437601033	2.89E-10	2.96E-09
LINC00862	-1.089427676	-1.679115342	-6.434335307	2.95E-10	3.02E-09
RP11_144A16.8	1.018560272	-5.378306003	6.426786829	3.08E-10	3.15E-09
CCDC60	1.412433905	-1.827643839	6.425323003	3.11E-10	3.18E-09
GREM1	-1.28261827	4.684586223	-6.423956231	3.14E-10	3.20E-09
DNAH9	1.626512787	-0.153813967	6.421749406	3.18E-10	3.24E-09
HHIP_AS1	1.18653776	0.403599187	6.420263543	3.21E-10	3.27E-09
PPAPDC1A	-1.302021455	-0.106127369	-6.418837338	3.24E-10	3.29E-09
TCEAL2	1.099884807	-1.377976489	6.415965608	3.29E-10	3.34E-09
ODAM	1.262486432	-3.317225093	6.414569351	3.32E-10	3.37E-09
LINC01116	-1.184225563	-0.393157634	-6.414250161	3.33E-10	3.38E-09
HIST1H2BJ	-1.003936497	0.211304021	-6.411846709	3.38E-10	3.42E-09
FOXG1	-1.400547195	-4.834934139	-6.40286727	3.56E-10	3.60E-09
CHIT1	1.329804463	4.628447165	6.401710597	3.59E-10	3.62E-09
ERVV_1	-1.255639797	-4.103824283	-6.398347945	3.66E-10	3.69E-09
HOXC8	-1.435990166	-1.61235367	-6.393487754	3.77E-10	3.80E-09
EGLN3	-1.025638668	5.065349072	-6.391423918	3.82E-10	3.84E-09
PACRG	1.120841399	-0.987018723	6.379773437	4.10E-10	4.11E-09
RRAD	1.003846203	3.790838515	6.377118389	4.16E-10	4.17E-09
AC026471.6	1.123193397	-1.214990031	6.368288176	4.39E-10	4.38E-09
RP4_681L3.2	1.154326866	-2.937420867	6.366261741	4.45E-10	4.43E-09
ABO	1.013525487	4.464268296	6.36445214	4.49E-10	4.47E-09
DUXAP8	-1.15529278	0.779531887	-6.35907047	4.64E-10	4.60E-09
TEX19	-1.180328046	-3.962283383	-6.342791413	5.12E-10	5.05E-09
GPR158	-1.612139247	-1.12298773	-6.336839985	5.31E-10	5.22E-09
RP11_243M5.2	1.061788292	-2.950702803	6.336434964	5.32E-10	5.23E-09
RP11_88I21.2	1.063792141	-4.735677236	6.336420036	5.32E-10	5.23E-09
TRPA1	-1.166545025	-1.747806182	-6.326274749	5.65E-10	5.53E-09
PRAME	-2.358745886	1.039388844	-6.310532465	6.21E-10	6.04E-09
RP11_71E19.1	1.002179136	-3.764454574	6.308137989	6.30E-10	6.12E-09
PI16	1.170086464	-1.411151039	6.30660162	6.36E-10	6.17E-09
RP11_408B11.2	-1.356392161	-5.075188499	-6.303694329	6.47E-10	6.27E-09
COL6A5	1.075205422	1.284429857	6.292682673	6.91E-10	6.66E-09
HCN4	1.422002143	-1.684985307	6.291763607	6.95E-10	6.69E-09
NEU4	-1.022339189	-2.938577142	-6.275843632	7.64E-10	7.31E-09
AC069277.2	-1.372656693	-4.368221899	-6.27400678	7.72E-10	7.38E-09
AP000251.3	-1.170565172	-2.632559226	-6.269783385	7.92E-10	7.55E-09
AARD	1.166138663	-0.552461371	6.264354241	8.18E-10	7.78E-09
HOXC6	-1.274574497	0.174492221	-6.255170413	8.64E-10	8.16E-09
MMP12	-1.353636863	3.609114879	-6.252496146	8.77E-10	8.28E-09
MIR663AHG	1.592741511	-0.62504858	6.247906527	9.02E-10	8.50E-09

SPINK13	1.359680184	-1.638244715	6.245374129	9.15E-10	8.61E-09
FABP4	1.154509109	0.773803022	6.245153517	9.17E-10	8.62E-09
CA4	1.314246588	-0.53496007	6.243980457	9.23E-10	8.68E-09
ZNF280A	-1.346246007	-4.685731489	-6.241055197	9.39E-10	8.82E-09
DNASE1L3	1.037135389	0.166014092	6.233017186	9.85E-10	9.21E-09
KCNB1	1.176769198	-1.655900195	6.231474761	9.94E-10	9.29E-09
FOXD3	-1.293587978	-3.442539827	-6.230465528	1.00E-09	9.34E-09
COL6A6	1.067906051	1.815058335	6.22652989	1.02E-09	9.55E-09
SNTG2	1.023079402	-3.708250644	6.223347662	1.04E-09	9.72E-09
LINC00898	-1.135571877	-5.133400458	-6.222180935	1.05E-09	9.78E-09
SH2D5	-1.152086386	-2.562138072	-6.212349111	1.11E-09	1.03E-08
CTC_338M12.9	-1.067951388	-4.762340381	-6.209015882	1.13E-09	1.05E-08
SIM1	-1.328483608	-4.423691189	-6.207436035	1.15E-09	1.06E-08
HOXA10_AS	-1.083953333	-5.072437043	-6.203436401	1.17E-09	1.08E-08
IL5RA	1.022234422	-0.207897524	6.200432861	1.19E-09	1.10E-08
RP11_1260E13.2	1.06243072	-1.396967378	6.199889336	1.20E-09	1.10E-08
KCNMB2_AS1	-1.780612689	-1.298838517	-6.198850321	1.20E-09	1.11E-08
CXCL17	1.119691275	7.18315664	6.19462257	1.24E-09	1.14E-08
MS4A15	1.750502098	1.330586867	6.190981761	1.26E-09	1.16E-08
CTD_2591A6.2	-1.3727566	-4.724076375	-6.190861397	1.26E-09	1.16E-08
RP4_738P11.3	-1.091307774	-4.635744812	-6.184831515	1.31E-09	1.20E-08
RP11_476M19.3	1.03603286	-4.65259352	6.183166053	1.32E-09	1.21E-08
RP11_35N6.1	1.354931375	0.94509945	6.182534841	1.33E-09	1.21E-08
RP11_395E19.6	1.018281848	-5.152115538	6.1778923	1.36E-09	1.24E-08
SRGAP3_AS2	1.742504784	-2.03404952	6.177828937	1.36E-09	1.24E-08
RETN	1.197302156	-0.598296251	6.170630471	1.42E-09	1.29E-08
SLC9C2	1.046997737	-3.010333889	6.170012627	1.43E-09	1.30E-08
RP5_884M6.1	-1.746241342	-0.844279863	-6.168562272	1.44E-09	1.31E-08
CTC_490E21.13	1.059302024	-4.131435156	6.166765643	1.46E-09	1.32E-08
LRRC31	1.48066418	0.72468128	6.164961167	1.47E-09	1.33E-08
LINC01296	-1.177402387	-2.430569602	-6.162307812	1.49E-09	1.35E-08
EFCAB1	1.355182334	-0.028590539	6.160993259	1.51E-09	1.36E-08
MIR4697HG	1.220240347	1.508844389	6.158970095	1.52E-09	1.37E-08
TRHDE	1.274824215	-1.68595315	6.145022811	1.65E-09	1.48E-08
MAGEC1	-1.703591917	-4.093456718	-6.1397345	1.70E-09	1.52E-08
RP11_359M6.1	1.440452531	-2.13179545	6.135564754	1.75E-09	1.56E-08
KIR2DL4	-1.129330656	-1.745455453	-6.133621513	1.77E-09	1.57E-08
CAMK2N2	-1.014692244	-1.750262912	-6.122426055	1.89E-09	1.67E-08
TRIM63	1.168256072	-3.477710828	6.121236227	1.90E-09	1.68E-08
HIST1H1E	-1.047169268	-1.800851671	-6.120240527	1.91E-09	1.69E-08
ISM2	-1.151430252	-3.677434077	-6.117815555	1.94E-09	1.71E-08
RP11_483L5.1	1.226342098	-2.390762183	6.116028974	1.96E-09	1.73E-08
SSTR1	1.087042482	0.657030829	6.112804066	1.99E-09	1.76E-08
OFCC1	-1.286719145	-4.813809484	-6.109733502	2.03E-09	1.79E-08
FOSB	1.253461239	4.728956313	6.108982227	2.04E-09	1.80E-08
AGTR2	1.805785434	-0.540000725	6.103389174	2.11E-09	1.85E-08
SBSN	-1.476684308	-2.618014655	-6.101731587	2.13E-09	1.87E-08
RP1_27K12.4	-1.095513824	-2.662771932	-6.098383814	2.17E-09	1.90E-08
RP11_5407.18	-1.038317376	-2.284204146	-6.092331479	2.25E-09	1.97E-08
AQP7	1.189623051	1.024730394	6.0862985	2.33E-09	2.03E-08
VGF	-1.305956059	-0.991163702	-6.084875099	2.35E-09	2.04E-08
RP1_90L14.1	-1.201046459	-5.063415628	-6.083109332	2.37E-09	2.06E-08
LINC00525	-1.045320127	-2.520618101	-6.076244667	2.47E-09	2.14E-08
MYO18B	-1.294164606	-3.173511345	-6.067971487	2.59E-09	2.24E-08

CSTP1	1.171447266	-4.19309936	6.059509419	2.72E-09	2.35E-08
LRRC18	1.120326624	-2.431906235	6.058223791	2.74E-09	2.36E-08
CAPN6	1.536280435	0.099757283	6.055907869	2.78E-09	2.39E-08
DLX3	1.179272155	0.868525896	6.050579212	2.86E-09	2.46E-08
PPP1R1B	1.440036569	4.296023071	6.050313708	2.87E-09	2.46E-08
OVCH1	1.030325096	-3.085830015	6.047425733	2.91E-09	2.50E-08
ELFN1_AS1	-1.317213883	-2.503331616	-6.046999995	2.92E-09	2.51E-08
C2orf73	1.080931631	-2.751966691	6.044128828	2.97E-09	2.55E-08
NTNG1	1.231425608	-0.611728338	6.035432646	3.12E-09	2.67E-08
NAT8L	-1.204801185	0.381739873	-6.02924075	3.24E-09	2.76E-08
FAR2P1	-1.791183442	-3.469177042	-6.02309351	3.35E-09	2.85E-08
MKRN3	-1.486505621	-2.706495968	-6.022198618	3.37E-09	2.86E-08
TEKT1	1.587464473	-0.475157557	6.014267083	3.53E-09	2.98E-08
AC007128.1	-1.382962929	-1.779813034	-6.011363952	3.59E-09	3.03E-08
HPR	1.036191228	-3.38927038	6.009061353	3.64E-09	3.07E-08
AC012512.1	-1.186503545	-4.175877017	-6.008523049	3.65E-09	3.08E-08
C1orf158	1.484664672	-2.659027365	5.987242366	4.12E-09	3.45E-08
GLP1R	1.15461975	-2.97838906	5.984369659	4.19E-09	3.50E-08
RHOV	-1.068810018	3.578744321	-5.984221373	4.19E-09	3.50E-08
KLHDC7A	1.044676962	2.656574622	5.975872055	4.40E-09	3.66E-08
DSCR8	-1.400339757	-4.958978909	-5.974455913	4.43E-09	3.69E-08
RP13_463N16.6	-1.137508923	-2.243750464	-5.954855262	4.96E-09	4.09E-08
LINC01535	-1.018198761	-1.177688315	-5.953017351	5.01E-09	4.13E-08
NELL1	2.078989851	-1.304399065	5.951827507	5.04E-09	4.16E-08
NXF3	1.02945713	-1.471956431	5.949914994	5.10E-09	4.20E-08
CYP4Z1	1.041633412	-2.319861711	5.949024788	5.13E-09	4.21E-08
CRABP1	-1.652878376	-1.862708559	-5.94769199	5.16E-09	4.25E-08
C9orf135	1.479227858	-1.045463031	5.946113419	5.21E-09	4.28E-08
RNU6_403P	1.215219965	-1.478854086	5.94580506	5.22E-09	4.29E-08
RP11_108M12.3	-1.244768043	-3.601666281	-5.941120936	5.36E-09	4.39E-08
CHST8	1.032564818	-3.106093057	5.926531921	5.83E-09	4.73E-08
CPB2	1.534546155	-0.247217832	5.92257395	5.96E-09	4.83E-08
AC011298.2	-1.436403739	-3.920767793	-5.917335631	6.14E-09	4.95E-08
RP11_191N8.2	1.171264408	-1.927470488	5.906674688	6.52E-09	5.23E-08
RP11_328K4.1	-1.140842	-4.577913197	-5.905090963	6.58E-09	5.28E-08
CTC_499J9.1	-1.15267633	-4.556733131	-5.902782752	6.67E-09	5.34E-08
RP11_815M8.1	1.204952312	-0.07516837	5.898608771	6.82E-09	5.47E-08
RP11_308B16.2	-1.249856542	-5.293630329	-5.895468795	6.95E-09	5.55E-08
SYT1	-1.114353053	1.842993056	-5.895417138	6.95E-09	5.55E-08
DUSP27	1.018870265	-3.783483555	5.887335243	7.27E-09	5.79E-08
CCL19	1.02997571	3.519799133	5.884985366	7.37E-09	5.85E-08
CA10	1.865308343	-2.740221961	5.881930868	7.50E-09	5.95E-08
RP11_462L8.1	-1.023739438	-2.413789158	-5.88150419	7.52E-09	5.96E-08
KHDC1L	-1.249233992	-4.136305743	-5.879780905	7.59E-09	6.01E-08
MAGEA10	-1.709353981	-4.262562949	-5.879551606	7.60E-09	6.02E-08
LINC01105	1.306994415	-3.429020047	5.877864776	7.67E-09	6.07E-08
IL13RA2	1.111325225	-0.829573716	5.871665753	7.95E-09	6.27E-08
SCGB2A1	1.492178659	-1.29278399	5.867695637	8.13E-09	6.40E-08
CNTD2	-1.100882524	0.763659636	-5.859809577	8.50E-09	6.67E-08
TTC29	1.348203781	-1.982467664	5.856410895	8.66E-09	6.79E-08
MAGEA12	-2.100698984	-3.531293106	-5.848619997	9.05E-09	7.06E-08
SERPIND1	1.519290089	1.074865587	5.847384289	9.11E-09	7.10E-08
ALX1	-1.306832689	-4.181105431	-5.844499059	9.26E-09	7.21E-08
TRIM71	1.261715527	-1.799128653	5.836699811	9.67E-09	7.50E-08

PNCK	-1.135109796	-0.945575911	-5.836408006	9.69E-09	7.51E-08
NWD1	1.277296812	0.554626145	5.836231303	9.70E-09	7.52E-08
GAD1	-1.118313875	0.63724132	-5.82364822	1.04E-08	8.02E-08
RP11_238K6.1	1.398999957	-2.464003341	5.82134984	1.05E-08	8.12E-08
P3H2_AS1	1.017125577	-4.13318356	5.81516366	1.09E-08	8.38E-08
FAM83A_AS1	-1.092490364	0.734997118	-5.808206943	1.13E-08	8.69E-08
LINC00470	-1.373784973	-3.791593268	-5.805187429	1.15E-08	8.82E-08
SLC16A12	1.102525328	0.244983827	5.798416518	1.20E-08	9.14E-08
HIST1H1B	-1.115267786	-3.690549661	-5.793083142	1.23E-08	9.40E-08
CHRM1	1.070948943	-3.662757703	5.791962046	1.24E-08	9.45E-08
CASC9	-2.027391858	-1.258829587	-5.791480816	1.25E-08	9.47E-08
AC060834.2	1.020290783	-3.072847076	5.790735702	1.25E-08	9.51E-08
CPA4	-1.157396489	-2.743822673	-5.790305848	1.25E-08	9.53E-08
LECT1	1.811471	-3.384926445	5.787972059	1.27E-08	9.64E-08
GAS2L2	1.246284768	-0.418941644	5.786272324	1.28E-08	9.72E-08
TCAM1P	-1.498088161	-2.238019157	-5.784582123	1.29E-08	9.80E-08
EVPLL	1.075221325	-1.149753074	5.779668187	1.33E-08	1.01E-07
IYD	1.278274487	1.930772116	5.778422788	1.34E-08	1.01E-07
RIMS2	-1.534668325	-1.514808346	-5.776553466	1.35E-08	1.02E-07
LINC01468	-1.147418016	-4.601341284	-5.776101882	1.36E-08	1.02E-07
MAGEB2	-1.540064938	-4.707757495	-5.775287204	1.36E-08	1.03E-07
SCN1A	1.243154547	0.007280838	5.774220897	1.37E-08	1.03E-07
LINC01419	-1.414091662	-5.118411713	-5.766175866	1.43E-08	1.08E-07
PTCHD1	1.141447328	-1.193490193	5.758701152	1.49E-08	1.12E-07
C20orf85	1.797163709	-0.299516358	5.758303186	1.50E-08	1.12E-07
GGTLC2	1.009700066	-3.351598812	5.758029932	1.50E-08	1.12E-07
TEX15	-1.502089961	-3.499121397	-5.752138193	1.55E-08	1.16E-07
CTD_3010D24.3	-1.163810378	-0.457390302	-5.750808111	1.56E-08	1.17E-07
RP5_978I12.1	1.006088873	-5.048360386	5.745806272	1.61E-08	1.20E-07
RP4_539M6.14	1.124023149	-2.340803059	5.738299221	1.67E-08	1.24E-07
HMGCS2	1.70495918	-2.004907312	5.731809191	1.73E-08	1.28E-07
MSI1	-1.212067882	0.24224535	-5.724906306	1.80E-08	1.33E-07
RP11_1103G16.1	-1.125548368	-5.373700239	-5.72261087	1.83E-08	1.34E-07
RP4_755D9.1	1.023872536	0.086210027	5.717804463	1.87E-08	1.38E-07
AL109763.2	-1.279527897	-5.127052609	-5.717526453	1.88E-08	1.38E-07
ALDOB	1.154045541	-1.306727424	5.713173599	1.92E-08	1.41E-07
RP11_874J12.4	-1.174082656	-3.982719159	-5.710821118	1.95E-08	1.43E-07
PAX9	-1.034244024	2.599442902	-5.7107924	1.95E-08	1.43E-07
RORB	1.056094428	0.040308227	5.708382659	1.97E-08	1.44E-07
CXCL14	1.424217133	4.815574169	5.706828434	1.99E-08	1.45E-07
HBA2	1.017670437	1.63519555	5.705099226	2.01E-08	1.47E-07
FAM83A	-1.121658023	6.000322779	-5.700163704	2.07E-08	1.50E-07
HOXA11	-1.345112776	-3.853987014	-5.699869096	2.07E-08	1.51E-07
RP11_1C8.7	-1.173210285	-4.744905998	-5.693874184	2.14E-08	1.55E-07
FAM131C	-1.000383681	-2.847336965	-5.691099488	2.17E-08	1.58E-07
RP11_138J23.1	-1.308116835	-4.184607072	-5.691051282	2.17E-08	1.58E-07
IL17C	-1.106485621	-2.357740698	-5.684569025	2.25E-08	1.63E-07
MIR137HG	-1.165981525	-4.425953686	-5.675302566	2.37E-08	1.71E-07
CABYR	-1.087126989	2.230607953	-5.659363897	2.58E-08	1.85E-07
DNAI2	1.296700969	-1.30183983	5.636810787	2.92E-08	2.07E-07
RP11_85G21.2	1.092020602	-4.225398616	5.632281244	3.00E-08	2.12E-07
CCL7	-1.074101249	-1.195037981	-5.629930714	3.03E-08	2.15E-07
RP11_627G23.1	1.573408522	0.811863151	5.628788316	3.05E-08	2.16E-07
FCAMR	1.000236978	-2.326582772	5.626520354	3.09E-08	2.18E-07

C9orf24	1.18131358	-0.097631195	5.62036921	3.20E-08	2.25E-07
STMN2	1.162709122	-1.700352052	5.614199967	3.31E-08	2.32E-07
HOXC9	-1.350710912	-1.080709746	-5.61393614	3.31E-08	2.32E-07
SPOCK1	-1.103410342	2.382180167	-5.609907598	3.38E-08	2.37E-07
CTD_2544H17.1	1.070257607	-4.63546926	5.603298817	3.51E-08	2.45E-07
CTD_2589M5.4	1.236718348	-1.565507354	5.602932059	3.51E-08	2.45E-07
CDH19	1.053250703	-2.437257417	5.581382891	3.95E-08	2.73E-07
C11orf88	1.245271207	-1.170204108	5.57745863	4.03E-08	2.79E-07
HABP2	1.363027513	2.828725624	5.570652911	4.18E-08	2.88E-07
RP11_486A14.1	-1.163542044	-2.356838566	-5.565748021	4.30E-08	2.96E-07
DLL3	-1.537228551	-1.077028773	-5.563768775	4.34E-08	2.98E-07
IRX6	1.338357343	-0.032158204	5.560013879	4.43E-08	3.04E-07
POPDC3	-1.441349371	-1.494250041	-5.521355315	5.45E-08	3.69E-07
RP11_356K23.1	1.190897195	-2.128482918	5.520962962	5.47E-08	3.69E-07
CLGN	-1.065431199	1.163977149	-5.517570683	5.57E-08	3.75E-07
RP11_295M3.4	1.124681979	0.983226416	5.513642317	5.68E-08	3.83E-07
MUC21	1.579725878	4.114082169	5.51192336	5.74E-08	3.86E-07
CTD_2377D24.6	-1.243694924	-2.067295028	-5.511068985	5.76E-08	3.87E-07
RP4_666F24.3	1.109921375	-3.792555764	5.501560335	6.06E-08	4.06E-07
MST1L	1.08151932	1.692402284	5.489591949	6.46E-08	4.31E-07
SLC5A7	1.130822586	-3.966446929	5.487559448	6.53E-08	4.35E-07
ADGB	1.232165327	-2.558074214	5.486074194	6.59E-08	4.38E-07
LINC00664	-1.065710778	-1.49940613	-5.482261682	6.72E-08	4.46E-07
CAPN9	1.055281624	1.775774996	5.475676788	6.96E-08	4.61E-07
WT1_AS	-1.042380363	-3.513089787	-5.473135946	7.06E-08	4.66E-07
ABCC12	1.005747291	-3.997840742	5.471167227	7.13E-08	4.70E-07
AGBL1	1.057375578	-3.656653147	5.46486873	7.37E-08	4.85E-07
NKX2_1	1.032081085	6.675975164	5.463275319	7.44E-08	4.89E-07
AADAC	1.293708596	-0.192890575	5.445565704	8.17E-08	5.33E-07
RP11_161I6.2	-1.129476277	-3.030249532	-5.443583504	8.26E-08	5.38E-07
RP4_568B10.1	1.24856987	-3.169427787	5.442461359	8.30E-08	5.41E-07
C11orf97	1.215762573	-3.656907011	5.439793417	8.42E-08	5.48E-07
TUBA4B	1.129228079	-1.003312657	5.431630041	8.79E-08	5.71E-07
RP11_211G23.2	-1.323795412	-2.541695888	-5.416978195	9.50E-08	6.13E-07
CFAP46	1.065833354	-0.446745054	5.414646783	9.62E-08	6.19E-07
FGB	-2.697336716	0.921480714	-5.413179235	9.69E-08	6.23E-07
GSTA3	1.043882119	-3.703021424	5.409171195	9.90E-08	6.35E-07
NXPH4	-1.073534868	0.596937468	-5.406743411	1.00E-07	6.42E-07
TUBB2B	-1.238245952	1.599405145	-5.405203492	1.01E-07	6.47E-07
DMBT1	1.537675713	5.05501702	5.398509301	1.05E-07	6.68E-07
BAAT	1.230512256	0.079860784	5.398180468	1.05E-07	6.69E-07
CYP4F23P	-1.14072936	-3.788565934	-5.395541113	1.06E-07	6.77E-07
RPS16P9	-1.128642165	-4.422192137	-5.391614443	1.09E-07	6.90E-07
LINC01214	-1.19206569	-4.329203564	-5.378992079	1.16E-07	7.34E-07
CREG2	-1.028165898	-1.068629451	-5.375442523	1.18E-07	7.47E-07
RP11_89K21.1	-1.552200017	-2.117986062	-5.365657337	1.24E-07	7.83E-07
UCN3	1.501527245	-1.073755242	5.363497418	1.26E-07	7.91E-07
RP13_16H11.2	-1.1296943	-4.812924323	-5.356711514	1.30E-07	8.17E-07
KLF2P1	-1.15267279	-4.957379625	-5.348637724	1.36E-07	8.48E-07
RP11_424M24.5	1.050492403	-4.124633529	5.346930964	1.37E-07	8.54E-07
ORM1	1.229127367	1.254822908	5.341287924	1.41E-07	8.78E-07
CLDN2	1.544685071	2.324085428	5.330948745	1.49E-07	9.22E-07
RP11_357H14.17	-1.379607792	-2.280725774	-5.330129835	1.50E-07	9.25E-07
RP5_839B4.8	1.136037639	-1.965215999	5.326067598	1.53E-07	9.43E-07

RP11_76C10.6	1.319058228	1.332709115	5.317633994	1.60E-07	9.84E-07
RP11_663N22.1	-1.007135961	-3.382749812	-5.316837208	1.61E-07	9.87E-07
SLC5A12	-1.269137645	-2.209294454	-5.312066231	1.65E-07	1.01E-06
GABRA3	-1.767987481	-2.646339281	-5.299349744	1.76E-07	1.07E-06
GLB1L3	1.680693049	1.285089419	5.293622007	1.81E-07	1.10E-06
HCN1	1.28261025	-3.139703687	5.291300494	1.83E-07	1.11E-06
RP11_8L2.1	-1.334585357	-3.746254326	-5.289429807	1.85E-07	1.12E-06
PCSK1	-1.499702801	0.051631408	-5.276094884	1.98E-07	1.20E-06
CTD_2196E14.8	1.209178757	-3.618144929	5.274770422	2.00E-07	1.21E-06
MYEOV	-1.399173147	1.941230528	-5.274696645	2.00E-07	1.21E-06
LINC01571	1.003463346	-4.750041067	5.271624942	2.03E-07	1.22E-06
HTR2C	-1.309524676	-4.499474305	-5.268758691	2.06E-07	1.24E-06
THEG	-1.141752747	-3.510091017	-5.264590316	2.10E-07	1.27E-06
FAM133A	-1.695347537	-1.927666576	-5.260793737	2.14E-07	1.29E-06
HOXB_AS4	-1.077960548	-4.650486843	-5.254523023	2.22E-07	1.33E-06
PLA2G12B	1.407321807	-0.638319969	5.245411859	2.32E-07	1.39E-06
ORM2	1.179812326	0.321816005	5.243947043	2.34E-07	1.40E-06
WFDC12	1.134008192	-3.766447189	5.239892955	2.39E-07	1.42E-06
SLC1A7	1.341175162	2.17344202	5.239035284	2.40E-07	1.43E-06
AC009410.1	-1.156263791	-4.642727545	-5.233061197	2.47E-07	1.47E-06
ZDHHC11B	1.009017455	1.979798636	5.231643741	2.49E-07	1.48E-06
ASNSP1	-1.213307235	-4.862333276	-5.230025464	2.51E-07	1.49E-06
TRIM15	-1.471543058	-2.385556218	-5.224993106	2.58E-07	1.53E-06
RP11_81M19.3	1.173621222	-3.175068644	5.224618305	2.58E-07	1.53E-06
MAGEA9	-1.077373335	-5.576845684	-5.211183403	2.77E-07	1.63E-06
RP11_157E21.1	1.074786752	-3.897955711	5.207279023	2.82E-07	1.66E-06
RP11_169F17.1	-1.980710552	-0.462159922	-5.200890078	2.92E-07	1.71E-06
MROH9	1.022576198	-3.085569982	5.191359285	3.06E-07	1.79E-06
RP11_3B12.5	-1.155243093	-4.300588847	-5.184023281	3.18E-07	1.85E-06
RP11_109J4.1	1.051931921	-0.658015932	5.183966936	3.18E-07	1.85E-06
CYP2A6	1.073439327	-2.03853047	5.180863133	3.23E-07	1.88E-06
TEPP	1.082984008	-0.734973618	5.179822385	3.25E-07	1.89E-06
MEGF11	1.057687367	-0.550904358	5.174584237	3.33E-07	1.93E-06
ANKRD66	1.201528902	-2.245460901	5.164502447	3.51E-07	2.03E-06
IL36RN	-1.495364048	-1.225111166	-5.158819154	3.61E-07	2.08E-06
FOXD1	-1.142168254	-2.017443586	-5.155223353	3.68E-07	2.12E-06
F11_AS1	1.021457236	-3.44789403	5.14528482	3.87E-07	2.22E-06
LIN28B	-1.332504485	-4.898446596	-5.138341575	4.00E-07	2.29E-06
COX7B2	-1.324678577	-5.074268544	-5.136866304	4.03E-07	2.31E-06
RP11_76C10.5	1.317462108	0.469312201	5.136677918	4.04E-07	2.31E-06
DMBX1	-1.207030055	-1.188432736	-5.135643217	4.06E-07	2.32E-06
C11orf16	1.013851457	-0.358279973	5.131225046	4.15E-07	2.37E-06
PAX7	1.926285308	-2.396863705	5.105954941	4.71E-07	2.66E-06
VWDE	-1.016443772	0.747836173	-5.103384768	4.77E-07	2.69E-06
AC010091.1	-1.278385675	-4.236035351	-5.101180643	4.83E-07	2.72E-06
HOXC_AS2	-1.190038933	-2.488394786	-5.099567195	4.87E-07	2.74E-06
WT1	-1.124003656	-1.608164985	-5.093843906	5.01E-07	2.81E-06
ERN2	1.522463054	1.813258914	5.090684421	5.09E-07	2.85E-06
MS4A8	1.490330101	0.225258458	5.090166778	5.10E-07	2.86E-06
SLC6A4	1.024821269	-0.904719133	5.082183113	5.31E-07	2.96E-06
CTD_2291D10.4	-1.077105504	-2.175906252	-5.080729573	5.35E-07	2.98E-06
C6orf118	1.266668654	-2.103714435	5.080532036	5.35E-07	2.98E-06
LINC00648	-1.449485202	-3.431401267	-5.064839603	5.79E-07	3.21E-06
KRTAP3_1	-1.046706981	-4.519118775	-5.062608848	5.86E-07	3.24E-06

KIF1A	-1.874593142	0.747676206	-5.06191836	5.88E-07	3.25E-06
AC018890.6	-1.103101501	-2.756509948	-5.061340351	5.89E-07	3.26E-06
RP11_129M6.1	-1.295894787	-3.459446212	-5.053479341	6.13E-07	3.37E-06
TMEM212	1.211818369	-3.011889979	5.045676486	6.37E-07	3.50E-06
HHATL	1.187158468	-3.206370802	5.045333194	6.38E-07	3.50E-06
PIH1D3	1.159139027	-2.883855866	5.044634501	6.40E-07	3.51E-06
FOXD3_AS1	-1.198121357	-1.498978456	-5.042877055	6.46E-07	3.54E-06
HIST1H2BG	-1.109416963	-0.598376858	-5.03302208	6.78E-07	3.71E-06
KISS1R	-1.048209757	-0.737420157	-5.028594185	6.93E-07	3.79E-06
AQP2	1.079297692	-3.435872571	5.006853483	7.72E-07	4.19E-06
HP	1.408866066	3.425912348	5.005149422	7.79E-07	4.22E-06
LINC00942	-1.302748724	-0.487117632	-5.002343117	7.90E-07	4.27E-06
RP11_116N8.4	1.114946266	-3.492476197	4.994550576	8.21E-07	4.43E-06
LINC01564	-1.082945374	-2.112073453	-4.983189258	8.68E-07	4.67E-06
SUN3	-1.121644647	-2.932837845	-4.983127274	8.68E-07	4.67E-06
HTR1D	-1.079003634	0.13089787	-4.980239861	8.80E-07	4.73E-06
LINC01518	-1.044581082	-5.00398773	-4.976368767	8.97E-07	4.81E-06
TPSP2	-1.137787785	-3.840069617	-4.974681168	9.05E-07	4.84E-06
ARSE	1.046028115	3.605825021	4.967957635	9.35E-07	4.99E-06
TRIM51BP	1.082907607	-4.0698723	4.945077788	1.05E-06	5.53E-06
CALY	-1.095504519	-2.751723174	-4.940972856	1.07E-06	5.63E-06
AC012531.25	-1.002082582	-3.805536849	-4.939244489	1.08E-06	5.67E-06
RERGL	1.096058393	-2.20966498	4.938814975	1.08E-06	5.68E-06
TSPAN8	1.175569898	4.404402617	4.936542319	1.09E-06	5.74E-06
FAM3D	1.040211907	0.396880284	4.933874291	1.11E-06	5.81E-06
ARSEP1	1.07778165	-3.248940813	4.933021363	1.11E-06	5.83E-06
APOBEC4	1.173340334	-2.473334963	4.932225184	1.11E-06	5.85E-06
HIST1H2AD	-1.009194289	-2.435721778	-4.931010872	1.12E-06	5.88E-06
LINC00941	-1.015735794	-1.843659011	-4.916371603	1.20E-06	6.26E-06
LYPD6	-1.054903741	-0.265566002	-4.914135502	1.22E-06	6.32E-06
ZNF730	-1.004808924	-1.740368092	-4.911603593	1.23E-06	6.40E-06
ECEL1P2	1.042943894	-1.543294872	4.907965118	1.25E-06	6.50E-06
GABRQ	-1.047126648	-3.236654736	-4.906711611	1.26E-06	6.54E-06
TNNT1	-1.335642729	2.423768702	-4.898687632	1.31E-06	6.78E-06
PADI3	-1.397173207	-3.13988378	-4.898021566	1.32E-06	6.80E-06
DMRTC2	-1.129938737	-4.598241517	-4.896730477	1.32E-06	6.84E-06
STOML3	1.225961214	-1.718852737	4.896099903	1.33E-06	6.86E-06
FDCSP	1.187057089	0.236443163	4.872860227	1.49E-06	7.61E-06
LHX1	-1.207134421	-4.197292575	-4.861430805	1.57E-06	8.00E-06
HIST1H2AE	-1.09380173	-1.074794198	-4.851213801	1.65E-06	8.36E-06
GNGT1	-1.311986566	-2.313114145	-4.845692648	1.69E-06	8.56E-06
ADH1C	1.244737537	1.31493968	4.842354952	1.72E-06	8.69E-06
HOXD10	-1.135587613	-2.518066414	-4.8397313	1.74E-06	8.78E-06
RP4_594A5.1	-1.053537812	-3.407048083	-4.831985594	1.81E-06	9.08E-06
CATSPERD	1.038621237	-2.959623801	4.823398896	1.89E-06	9.44E-06
SLC6A15	-1.287623814	-3.496701189	-4.773867752	2.39E-06	1.17E-05
LY6K	-1.516482536	0.938615666	-4.77255731	2.40E-06	1.17E-05
RP4_547N15.3	1.053991256	-4.154700936	4.766532238	2.47E-06	1.21E-05
MSLN	1.448617356	6.354303961	4.765065112	2.49E-06	1.21E-05
C1orf141	1.092405167	-4.149890691	4.760515153	2.54E-06	1.24E-05
HOXB8	-1.288805961	-1.390738166	-4.757863717	2.58E-06	1.25E-05
RP11_102K13.5	-1.023617953	-1.006013095	-4.755922496	2.60E-06	1.26E-05
MAGEA1	-1.477786439	-4.276276333	-4.753270997	2.63E-06	1.28E-05
ABCC2	-1.20240544	0.97108641	-4.751913102	2.65E-06	1.28E-05

SYT5	-1.033540103	-1.921081658	-4.736564453	2.85E-06	1.37E-05
RP11_469H8.6	1.2628119	-0.751407216	4.729506583	2.94E-06	1.41E-05
SRD5A2	1.077110194	-0.541526376	4.726477694	2.99E-06	1.43E-05
COL2A1	-1.262656951	-2.251807905	-4.725741215	3.00E-06	1.44E-05
RP13_870H17.3	1.095879115	-0.942875392	4.725166988	3.01E-06	1.44E-05
FGF5	-1.005114512	-4.072924196	-4.716234872	3.13E-06	1.50E-05
AC008271.1	-1.030716473	-4.714947425	-4.709002684	3.24E-06	1.54E-05
HHIPL2	-1.333065517	0.500576304	-4.708548847	3.25E-06	1.55E-05
CHST9	1.412228366	-0.640036452	4.69134338	3.52E-06	1.67E-05
FAR2P4	-1.015981813	-4.839492304	-4.675721123	3.79E-06	1.78E-05
RP3_407E4.3	-1.16374914	-1.91139854	-4.673787806	3.82E-06	1.80E-05
IHH	1.027075822	-1.955852438	4.67051079	3.88E-06	1.82E-05
DRGX	1.234916949	-1.676884315	4.666698931	3.95E-06	1.85E-05
MYO16_AS1	1.039481483	-3.497984494	4.663340451	4.01E-06	1.88E-05
KCNH5	-1.009207189	-4.824202066	-4.659129205	4.09E-06	1.91E-05
NR0B2	1.185152505	-0.110464867	4.654168357	4.19E-06	1.95E-05
AQP5	1.434129388	3.251592341	4.65241147	4.22E-06	1.97E-05
CAPSL	1.150827068	-1.282089343	4.651249193	4.25E-06	1.98E-05
CDH7	-1.015109417	-3.809694407	-4.626528445	4.76E-06	2.20E-05
MAGEA4	-1.546242093	-4.404734841	-4.618931396	4.93E-06	2.27E-05
TMEM213	1.0850006	0.269917503	4.604782757	5.26E-06	2.41E-05
SOX2	-1.203307477	2.630020835	-4.602534241	5.32E-06	2.43E-05
HHLA2	1.421899628	1.269751282	4.570048435	6.17E-06	2.79E-05
AKAP14	1.027329452	-2.654290068	4.56311656	6.37E-06	2.87E-05
AC098973.2	-1.289250953	-4.366517689	-4.552827255	6.68E-06	3.00E-05
INA	-1.213258999	-1.48191249	-4.548377703	6.82E-06	3.05E-05
NETO1	-1.024283101	-1.180590454	-4.525654555	7.56E-06	3.35E-05
SP3P	-1.206656159	-3.295878379	-4.521025452	7.72E-06	3.42E-05
APOH	1.190248718	0.504342896	4.507298992	8.21E-06	3.61E-05
HOXA10	-1.18632756	-0.484841403	-4.506937837	8.23E-06	3.62E-05
DUSP13	-1.2307798	-1.34680697	-4.505567831	8.28E-06	3.64E-05
HMGA2	-1.445606243	0.413437738	-4.501949776	8.42E-06	3.69E-05
H19	-1.160940108	3.110637628	-4.500540846	8.47E-06	3.71E-05
MAGEA11	-1.0428453	-5.14117028	-4.499419629	8.51E-06	3.73E-05
TLR8_AS1	1.021244412	-3.112473465	4.497621132	8.58E-06	3.75E-05
MUC16	-1.391861938	3.770079377	-4.492288466	8.79E-06	3.84E-05
HSD17B2	1.061029954	0.028217613	4.48724909	8.99E-06	3.91E-05
C9orf171	1.003042998	-1.578344684	4.480292566	9.28E-06	4.03E-05
HOXC_AS3	-1.131239864	-4.016251869	-4.474529242	9.52E-06	4.13E-05
RP11_774D14.1	-1.007717541	-5.172105591	-4.474440116	9.53E-06	4.13E-05
PRAP1	-1.085303338	-2.105423169	-4.466915138	9.85E-06	4.26E-05
DPP10	1.192843348	-0.241447787	4.460384798	1.01E-05	4.37E-05
BRINP1	1.277950401	-0.221799834	4.45922981	1.02E-05	4.40E-05
ERICH3	1.185922037	-0.565787339	4.455665502	1.04E-05	4.46E-05
ALDH3A1	1.245103597	2.736378506	4.446770717	1.08E-05	4.62E-05
MORN5	1.044292672	-1.888597101	4.440143357	1.11E-05	4.75E-05
KRT83	-1.039452545	-2.965510424	-4.408218733	1.28E-05	5.41E-05
RP4_809F18.1	1.069451661	-3.825759032	4.404044607	1.30E-05	5.50E-05
LTF	1.019149579	5.065988468	4.40361601	1.31E-05	5.50E-05
LYPD8	-1.05860941	-3.141628356	-4.39748768	1.34E-05	5.64E-05
FAM83F	-1.040825157	1.580570212	-4.385359864	1.42E-05	5.91E-05
ZIC1	-1.271698639	-4.008570148	-4.375665935	1.48E-05	6.15E-05
IL37	1.360889608	-0.587012128	4.374423928	1.49E-05	6.18E-05
PIWIL3	-1.036050883	-4.887581428	-4.369814087	1.52E-05	6.29E-05

HOXC12	-1.331708439	-4.221144281	-4.338333782	1.74E-05	7.15E-05
IGHD	1.134881898	4.387074361	4.325052607	1.85E-05	7.54E-05
TMPRSS11E	-1.280330447	1.177782317	-4.318565895	1.90E-05	7.74E-05
LINC00858	-1.302575601	-2.400763078	-4.306410109	2.00E-05	8.10E-05
FSTL5	-1.121480018	-4.24502279	-4.299157996	2.07E-05	8.33E-05
ZIC4	-1.023233958	-4.782130843	-4.281165773	2.24E-05	8.94E-05
VIL1	-1.529424587	1.033821929	-4.266458119	2.38E-05	9.49E-05
SPAG6	1.10152794	0.558647015	4.256198344	2.49E-05	9.88E-05
CALML3	-1.087263462	-3.137040735	-4.179615171	3.46E-05	0.000133287
F2	-1.043213471	-3.971254337	-4.17738343	3.49E-05	0.000134432
HOXA13	-1.086302653	-4.192266297	-4.163839121	3.70E-05	0.000141602
CTAG2	-1.176546646	-4.856761521	-4.149350431	3.93E-05	0.000149333
LINC00668	-1.139344199	-3.86678131	-4.113331233	4.57E-05	0.000170879
CHP2	1.010911096	-3.715209684	4.09896523	4.85E-05	0.000180501
RP11_785D18.3	-1.074070287	-2.54114433	-4.066293682	5.56E-05	0.000204004
RP11_776H12.1	-1.101834046	-3.03295548	-4.063980679	5.62E-05	0.00020579
RP11_1038A11.3	-1.185430565	-2.690407586	-4.052533555	5.89E-05	0.00021473
C8B	1.028145121	-1.386825589	4.03688066	6.28E-05	0.000227563
CLDN8	1.07434482	-0.571440085	4.005126411	7.16E-05	0.00025573
CT83	-1.50749032	-1.487523787	-4.002955499	7.22E-05	0.000257617
RP11_116O18.1	1.346414941	-2.53423348	3.990624779	7.59E-05	0.00026964
DLX6	-1.095586788	-2.834791319	-3.961215924	8.56E-05	0.000300606
GSTA1	1.048983855	2.142235416	3.958958667	8.64E-05	0.000303117
ZIC2	-1.158399376	-1.708370909	-3.929077993	9.75E-05	0.000337661
SLC14A2	1.155180181	-1.582894281	3.912762832	0.000104092	0.000358275
CASP14	-1.018989971	-4.791747758	-3.909745827	0.000105361	0.0003623
TSPAN19	1.057856186	-2.27111354	3.895196311	0.000111687	0.000381806
LRRTM1	1.049935281	-3.098399915	3.887064064	0.000115378	0.000392951
TF	-1.046763609	1.159860438	-3.882204808	0.000117638	0.000400042
SPINK1	1.208982684	4.318659266	3.858632123	0.00012921	0.000435385
TM4SF4	1.23652027	0.188850294	3.817202895	0.000152197	0.000505086
UGT3A1	-1.037544405	-4.81423245	-3.815751368	0.000153069	0.000507748
C14orf105	1.072739075	-2.607920832	3.785583338	0.000172285	0.000565384
ALPPL2	1.029358507	-1.470111906	3.77598186	0.000178863	0.000584686
CALML5	-1.088227136	-3.893954925	-3.775975635	0.000178868	0.000584686
HOXC10	-1.420979717	-1.165837142	-3.757949328	0.000191864	0.000622289
CYP24A1	-1.042837713	4.134993898	-3.734003217	0.000210508	0.000676899
SERPINB5	-1.211858037	0.576602884	-3.651443279	0.00028872	0.000900311
NOVA1_AS1	-1.045969933	-3.444017172	-3.576681208	0.000382395	0.001156696
KLK6	-1.173605199	-1.230036812	-3.433543766	0.000646062	0.001853039
HOXB13	-1.162824782	-2.357467938	-3.309483983	0.001003264	0.002753104
FGL1	-1.16399313	1.606144148	-3.257165278	0.001203031	0.003233195
RP11_462G2.1	1.001912432	-0.940300063	3.254724739	0.001213194	0.003257574
CPS1	-1.066494957	2.689328419	-2.991752602	0.002913086	0.00707696
HOXC11	-1.08506379	-2.134529051	-2.989359211	0.002935589	0.007125694
PAEP	-1.096760539	1.526520288	-2.762631312	0.005948496	0.013312954
FGA	-1.016960921	3.455860476	-2.589484935	0.009896806	0.020752745

B	change
181.5503194	DOWN
170.315169	DOWN
164.4452383	DOWN
158.7063321	DOWN
158.4429857	DOWN
156.62774	DOWN
156.4585055	DOWN
155.9356898	DOWN
155.6672154	DOWN
153.603277	DOWN
151.1380231	DOWN
150.9365493	DOWN
150.3490896	DOWN
149.2719144	DOWN
147.8343502	DOWN
144.8521813	DOWN
144.5120899	DOWN
143.0873672	DOWN
142.5808132	DOWN
142.4072966	DOWN
141.465498	DOWN
140.8885641	DOWN
140.7204247	DOWN
137.8999113	DOWN
136.5745434	DOWN
136.2650985	DOWN
135.9148746	DOWN
135.6756804	DOWN
135.5407183	DOWN
135.557608	DOWN
135.3780413	DOWN
134.3480694	DOWN
133.4495109	DOWN
133.4118891	DOWN
132.9469876	DOWN
132.4954243	DOWN
132.0438	DOWN
131.7248198	DOWN
131.4837304	DOWN
130.3754533	DOWN
129.1205554	DOWN
128.7336806	DOWN
128.281458	DOWN
127.614089	DOWN
127.3230807	DOWN
127.1482921	DOWN
127.0190395	DOWN
126.4888649	DOWN
126.5561342	DOWN
126.137196	DOWN
125.1171644	DOWN
124.9893338	DOWN
124.8006314	DOWN

124.6593525 DOWN
124.3222877 DOWN
124.5452549 DOWN
124.1265476 DOWN
123.3440177 DOWN
123.0268878 DOWN
122.7070055 DOWN
122.4537805 DOWN
122.438423 DOWN
122.0479591 DOWN
122.0435845 DOWN
121.6444427 DOWN
121.6857912 DOWN
121.3405382 DOWN
121.1905192 DOWN
120.8909908 DOWN
120.377379 DOWN
119.9175475 DOWN
119.7448171 DOWN
119.2874677 DOWN
118.9356935 DOWN
118.9015082 DOWN
118.7761184 DOWN
118.6609538 DOWN
118.2628624 DOWN
118.2171416 DOWN
117.4079968 DOWN
117.2487784 DOWN
117.0774058 DOWN
115.9893331 DOWN
115.9372779 DOWN
115.2568915 DOWN
115.0877102 DOWN
114.9907125 DOWN
114.3075131 DOWN
114.1481931 DOWN
113.9371468 DOWN
114.0189336 DOWN
113.9401875 DOWN
113.8836776 DOWN
113.7342299 DOWN
113.6108406 DOWN
112.6318138 DOWN
112.6146382 DOWN
112.3881008 DOWN
111.672676 DOWN
111.033371 DOWN
110.9611873 DOWN
110.6996236 DOWN
110.0171821 DOWN
108.8825191 DOWN
108.761404 DOWN
108.4370505 DOWN
108.2255227 DOWN

107.9544309	DOWN
107.9548031	DOWN
107.4451102	DOWN
107.3268645	DOWN
106.9427984	DOWN
106.684954	DOWN
106.4373028	DOWN
105.7545064	DOWN
105.1339471	DOWN
104.8228745	DOWN
103.9311401	DOWN
103.5960073	DOWN
103.4335239	DOWN
103.3420036	DOWN
102.5869061	DOWN
102.3671448	DOWN
102.0646833	DOWN
101.7962935	DOWN
101.8448053	DOWN
101.8598648	DOWN
101.7900869	DOWN
101.5243387	DOWN
101.2625186	DOWN
100.8249763	DOWN
100.6651224	DOWN
100.2521419	DOWN
100.1355548	DOWN
99.70365592	DOWN
99.39927919	DOWN
99.29536722	DOWN
98.21666983	DOWN
96.75772028	DOWN
96.48649311	DOWN
96.4777846	DOWN
96.32908968	DOWN
95.71430541	DOWN
95.40839934	DOWN
94.33041394	DOWN
93.43602493	DOWN
93.38265838	DOWN
93.17250495	DOWN
92.69453995	DOWN
92.23411682	DOWN
91.03065491	DOWN
90.37576715	DOWN
89.94688706	DOWN
89.82628437	DOWN
89.11377852	DOWN
88.16830025	DOWN
88.03834753	DOWN
87.53121649	DOWN
86.29695658	DOWN
86.02001456	DOWN
85.54651342	DOWN

85.45887002 DOWN
85.31401187 DOWN
84.97750082 DOWN
84.79344917 DOWN
84.79885942 DOWN
84.02080384 DOWN
81.10629107 DOWN
80.77734783 DOWN
79.65379018 DOWN
79.16845867 DOWN
78.51056532 DOWN
77.41302306 DOWN
77.35492712 DOWN
76.36341967 DOWN
76.23263074 DOWN
75.50680687 DOWN
75.50162704 DOWN
74.67633676 DOWN
74.34310973 UP
74.16490854 UP
73.81413928 DOWN
73.47837279 UP
72.56937929 DOWN
72.36291993 DOWN
71.79424395 DOWN
70.36644997 DOWN
70.10386854 DOWN
69.73141199 UP
68.80892175 DOWN
68.72090689 UP
68.61878173 DOWN
68.2691468 DOWN
67.81242393 DOWN
67.69580278 DOWN
67.39910767 DOWN
67.15442291 UP
67.09437766 DOWN
66.17610425 UP
64.6303333 UP
64.52674807 DOWN
63.58866907 DOWN
61.33650366 DOWN
60.36332872 UP
59.99516326 UP
59.85349528 DOWN
59.76720566 DOWN
59.13390357 DOWN
58.79628902 UP
58.35123317 UP
57.77048657 DOWN
57.19497335 UP
57.54028699 UP
57.00056223 DOWN
56.8943052 DOWN

56.60564314 UP
56.55009911 UP
56.05964593 DOWN
55.67321371 DOWN
55.76039903 UP
55.62026177 DOWN
55.21984491 UP
54.92543023 UP
54.71057331 UP
54.55181032 DOWN
54.17315287 DOWN
53.86774302 UP
53.98650638 UP
53.93915334 UP
53.53019832 UP
53.67980098 UP
53.6102533 DOWN
53.52228537 DOWN
52.65227472 UP
52.48441269 DOWN
52.80349672 UP
52.39785625 DOWN
51.80636129 DOWN
51.60706371 UP
51.40877227 DOWN
51.12923354 DOWN
50.66061436 DOWN
50.50259095 UP
50.33702565 UP
50.17719146 UP
49.61461802 UP
49.34502197 UP
49.46671016 UP
49.45605086 UP
49.28257611 DOWN
48.84409788 UP
48.97541137 UP
48.81991773 UP
48.92047733 DOWN
48.65290248 UP
48.27617878 DOWN
48.32637482 UP
48.24651407 UP
48.01503634 UP
47.62307597 UP
47.76203855 UP
47.1443602 UP
46.99185805 UP
46.98712248 UP
46.6570359 UP
46.75402246 DOWN
46.59378241 UP
46.02618282 UP
46.29747115 UP

46.15312765	UP
46.1830031	DOWN
46.04183104	UP
45.75295561	DOWN
45.70867412	UP
45.5498773	UP
45.38974201	DOWN
45.14384903	UP
44.60247545	UP
44.20229194	UP
44.29554919	UP
44.32856816	DOWN
43.80485099	UP
44.14133986	UP
44.06801038	UP
44.12777325	UP
44.08715073	DOWN
44.01363819	DOWN
43.81414242	DOWN
43.47490203	UP
43.47062204	UP
43.42542134	UP
43.17064128	DOWN
42.91165984	UP
42.79994073	UP
42.30440407	UP
42.57076339	UP
42.30838539	UP
42.53073582	UP
42.49305718	DOWN
42.39921084	UP
42.18553031	UP
41.779633	UP
41.54275218	DOWN
41.66674451	UP
41.39936761	UP
40.87220324	UP
41.03664437	UP
41.02950692	DOWN
40.60371019	UP
40.31006242	UP
40.65632027	UP
40.15934973	UP
39.72626992	UP
40.01609314	UP
39.9354583	UP
39.5627307	UP
39.50294505	DOWN
39.50468912	UP
39.1796952	DOWN
38.54107459	UP
38.99277346	UP
38.8247034	UP
38.71433627	DOWN

38.62193655	UP
38.46154197	UP
38.33273185	DOWN
38.28469764	UP
38.26544891	UP
37.79644524	UP
37.67990975	UP
37.64454225	UP
37.5254256	UP
37.28665949	UP
37.56333072	UP
37.51311494	DOWN
37.311782	UP
37.46194438	UP
37.35522219	UP
36.93610625	UP
37.06992327	UP
37.13462847	DOWN
36.89732452	UP
36.91633727	UP
36.48171725	DOWN
36.38610662	UP
36.43559253	UP
36.30915968	UP
36.05649255	UP
36.14099626	UP
35.78517315	DOWN
35.80852052	UP
35.59678389	UP
35.48312143	UP
35.21660909	UP
35.29309309	UP
35.05223907	UP
34.54962739	UP
34.85217408	UP
34.64098867	UP
34.67666616	UP
34.6424303	UP
34.56241669	UP
34.58318924	DOWN
34.48996127	UP
34.30519563	UP
34.03397711	UP
34.06200815	DOWN
33.92070059	UP
33.85462187	UP
33.90126914	UP
33.32096979	UP
33.74510443	UP
33.53535919	UP
33.45434613	UP
33.38367839	UP
33.39000742	UP
33.38696897	DOWN

33.26838654 UP
33.1089049 UP
32.65522575 UP
33.07218906 DOWN
33.0638533 UP
32.50514402 UP
32.85674484 UP
32.85025875 UP
32.61808124 UP
32.55421054 UP
32.73782279 DOWN
32.33344286 UP
31.90790346 UP
32.14870247 DOWN
32.08599496 DOWN
32.04178496 UP
31.60569974 UP
31.74785664 UP
31.7511813 UP
31.73547779 UP
31.43488227 UP
31.68432949 DOWN
31.65956353 UP
31.60636294 UP
31.45232981 DOWN
31.47280116 DOWN
31.21113736 UP
31.19627426 UP
31.4443888 UP
31.31470725 UP
31.33815075 UP
30.95236199 UP
31.04307627 UP
30.88270173 UP
31.04905556 UP
30.90183411 UP
30.94361346 UP
30.76184923 UP
30.9096044 UP
30.62815245 UP
30.88674868 UP
30.82225054 UP
30.79411182 UP
30.41127005 UP
30.45206275 UP
30.72779765 UP
30.44197206 UP
30.54145107 UP
30.52020494 UP
30.03319317 DOWN
29.86077747 UP
30.1802297 UP
29.9679942 UP
30.02613642 UP

30.16203363 DOWN
30.08677876 UP
29.88244818 UP
29.99703467 UP
29.96017232 UP
29.8435401 UP
29.23990452 UP
29.79437801 UP
29.70062158 DOWN
29.14817138 DOWN
29.55323523 DOWN
29.37406331 UP
28.88704866 DOWN
29.40508191 DOWN
29.22108111 UP
29.25474329 UP
29.22907172 DOWN
29.22654441 DOWN
28.43564906 UP
28.83629292 UP
28.90099701 UP
28.81486801 DOWN
28.74417456 UP
28.65416952 UP
28.74198359 UP
28.54731787 DOWN
28.59821925 DOWN
28.57884444 DOWN
28.43722473 UP
28.0262276 UP
28.04828428 UP
28.03742929 UP
28.02354343 UP
27.8655703 DOWN
27.70884718 UP
27.82211088 UP
27.86450329 DOWN
27.78308077 UP
27.22233896 UP
27.77150201 DOWN
27.65166712 UP
27.70994043 UP
27.48373507 UP
27.55243553 DOWN
27.46146073 UP
27.39228374 UP
27.24375434 UP
26.73085112 UP
26.64874552 DOWN
27.09184903 UP
26.66918295 UP
26.67812482 UP
26.75699765 UP
26.71741865 DOWN

26.18894241	UP
26.04123016	UP
26.57892916	UP
26.59902688	DOWN
26.54484946	UP
26.42224062	UP
26.44887974	UP
25.98092508	UP
26.3067116	UP
26.18481965	UP
26.04415808	UP
25.94437848	UP
26.16564658	UP
26.14940982	UP
25.8465708	DOWN
25.80038214	DOWN
25.76950792	DOWN
25.2713714	UP
25.19566346	UP
25.48012458	DOWN
25.09542474	UP
25.0382327	UP
25.21638189	UP
25.41260773	DOWN
25.20487933	UP
24.84613963	UP
25.20487816	UP
25.21202147	DOWN
24.57169991	UP
24.96172267	UP
24.78819318	DOWN
24.55356143	UP
24.84624744	UP
24.96534266	UP
25.00483616	UP
24.68264317	DOWN
24.97066562	DOWN
24.92835464	UP
24.90746727	UP
24.25417508	UP
24.88138535	DOWN
24.37603402	UP
24.19892804	UP
24.71631691	UP
24.70442382	UP
24.68358528	UP
24.29978917	UP
24.60085463	DOWN
24.17652678	UP
24.25774122	UP
24.40046849	DOWN
24.38085058	UP
24.29794891	UP
23.69959244	UP

24.28684722	DOWN
23.81335002	DOWN
24.06256968	UP
23.82819429	DOWN
23.54573204	UP
24.13163601	DOWN
23.61936017	UP
24.06815872	UP
23.96556721	UP
23.33351846	UP
23.44620545	UP
23.74329557	UP
23.78828553	UP
23.76093626	UP
23.47360206	UP
23.32190891	UP
23.27516992	UP
23.37388135	UP
23.505878	UP
23.3055072	UP
23.28488392	UP
23.39630449	UP
22.92837663	UP
23.34093519	UP
22.86311475	UP
22.99847236	UP
23.29844114	UP
23.13129103	DOWN
23.26267857	UP
23.08519111	UP
23.15190205	DOWN
23.1349209	UP
22.774055	UP
23.07927092	DOWN
22.48834114	UP
22.99660123	UP
22.38316195	UP
22.89731488	DOWN
22.87492724	UP
22.31810872	UP
22.87092931	UP
22.56521878	UP
22.38377082	UP
22.19369486	UP
22.55449888	UP
22.55299571	DOWN
22.45788347	UP
22.22616525	DOWN
22.44538229	UP
22.4326428	DOWN
22.40086799	UP
22.3184517	DOWN
22.36527281	UP
22.29607454	DOWN

22.29768877 DOWN
22.15225473 UP
22.0050107 UP
22.22982971 UP
21.78975109 UP
22.12318482 UP
21.79072121 DOWN
21.56066494 UP
22.0516189 DOWN
21.98492372 DOWN
21.8107518 UP
21.85753838 DOWN
21.26909016 UP
21.46517163 UP
21.63213947 DOWN
21.73786816 UP
21.56674095 UP
21.32209588 UP
21.64416132 UP
21.69001581 UP
21.62920905 UP
21.03366151 UP
21.53740693 UP
21.43915027 UP
21.50429178 UP
21.50279161 DOWN
21.48262185 UP
21.41148798 UP
20.86830302 UP
21.38215457 UP
21.29015016 UP
21.19473026 DOWN
21.16716239 UP
21.14219439 UP
20.58822554 UP
21.07107952 UP
21.03893314 DOWN
20.97090733 UP
21.026964 UP
20.80923046 DOWN
20.60679614 DOWN
20.26951661 DOWN
20.62065541 UP
20.54297902 DOWN
20.4632123 UP
20.16773285 UP
20.49570821 DOWN
20.37338526 UP
20.14508883 UP
19.86681685 UP
20.14279641 UP
20.10659715 UP
20.15303962 UP
20.01023005 UP

20.00014453	DOWN
20.11177974	UP
20.06657723	UP
19.91477693	DOWN
19.55596636	UP
19.92373353	DOWN
19.74913508	UP
19.88162563	DOWN
19.87654844	DOWN
19.7082453	UP
19.81324612	UP
19.4821205	UP
19.78841603	DOWN
19.65932326	UP
19.65856407	UP
19.60612222	UP
19.59680139	UP
19.61781732	UP
19.05882357	UP
19.46901238	DOWN
19.67823905	UP
19.68054632	UP
19.40399604	UP
19.62996872	DOWN
19.62338779	UP
19.6141643	UP
19.24300944	UP
19.54652311	UP
19.11396088	UP
19.53062939	UP
19.44937399	UP
19.34800446	UP
19.46579918	DOWN
19.41222765	DOWN
19.31814131	UP
19.38187627	DOWN
19.35627719	UP
18.9870535	UP
18.92683281	DOWN
19.25166169	UP
19.24804811	UP
19.27226021	UP
18.93123742	UP
18.54403516	UP
19.1318307	UP
19.14165929	UP
19.13008763	UP
19.09598023	DOWN
18.9501236	UP
19.01700547	UP
18.42719711	UP
18.97989762	UP
18.96127225	DOWN
18.9473954	UP

18.84850192 UP
18.38088507 UP
18.79714672 UP
18.47779806 UP
18.74432931 DOWN
18.74515974 UP
18.5925393 DOWN
18.70137678 UP
18.56106787 UP
18.35415797 UP
18.5844256 DOWN
18.09703805 UP
18.51755213 UP
18.36915435 UP
18.42973876 DOWN
18.52126385 DOWN
18.47283364 UP
18.43990589 DOWN
18.28838866 DOWN
18.31053909 UP
18.13364357 UP
18.30912144 DOWN
18.31183088 DOWN
18.30519318 DOWN
17.91257831 DOWN
18.28861616 UP
18.28103173 DOWN
17.84676316 UP
18.0544808 UP
18.01713919 DOWN
18.19333415 UP
18.15492328 DOWN
18.14148961 UP
17.37621564 UP
17.4114304 UP
17.93715665 DOWN
17.59306891 UP
17.87332623 DOWN
17.86511 UP
17.40444544 UP
17.84119538 DOWN
17.813257 UP
17.41584891 UP
17.76936962 UP
17.73825317 DOWN
17.54246441 UP
17.62709376 UP
17.53168283 UP
17.47251936 DOWN
17.27036116 UP
17.47182471 UP
16.7999502 UP
17.35061875 DOWN
17.26536381 UP

17.08405785 UP
17.17428734 DOWN
17.15401104 DOWN
17.13937501 DOWN
17.13775503 UP
17.10376874 UP
17.04497467 DOWN
16.31922824 UP
16.804698 UP
16.412603 UP
16.382173 UP
16.35310824 UP
16.80516277 DOWN
16.80668111 UP
16.77497376 DOWN
16.77191716 DOWN
16.50267094 UP
16.56563028 UP
16.68448833 DOWN
16.25316919 UP
16.5696847 UP
16.63255842 UP
16.32950285 UP
16.61829216 DOWN
16.40321558 UP
16.55002346 DOWN
16.5792777 UP
16.49764185 DOWN
16.13712923 UP
16.42581902 UP
16.28837365 UP
16.3746656 DOWN
16.35418288 DOWN
15.89011961 UP
16.25174632 DOWN
15.88098064 UP
16.24841773 DOWN
16.21516929 DOWN
16.23550442 UP
16.21203569 UP
16.14989092 UP
16.17500087 DOWN
16.10269543 DOWN
15.67179218 UP
16.03066562 DOWN
16.03319156 UP
15.97512831 DOWN
15.9116771 UP
15.89927559 DOWN
15.57938418 UP
15.7412803 UP
15.1446981 UP
15.71018452 UP
15.46058999 DOWN

15.77452689	DOWN
15.3502481	DOWN
15.30225752	UP
15.60369009	UP
15.69191584	UP
15.30383475	UP
15.61802661	DOWN
15.618336	UP
15.57752378	DOWN
15.40069003	UP
15.16674983	UP
15.53907875	DOWN
15.52927891	DOWN
15.41095566	UP
15.40390062	UP
15.393241	UP
15.3475956	UP
15.07994329	UP
15.39515364	DOWN
14.94964683	UP
15.24202016	DOWN
15.23726262	DOWN
15.23525561	UP
14.75689612	UP
15.19274989	UP
15.11881209	UP
15.02988643	DOWN
15.08637664	DOWN
14.92293918	UP
14.9875829	UP
14.88771308	UP
14.88533771	UP
14.50629088	UP
14.81889162	DOWN
14.54300537	UP
14.7141526	UP
14.61673092	UP
14.78088471	UP
14.77547378	UP
14.71688098	DOWN
14.69849517	UP
14.69525454	DOWN
14.68801408	DOWN
14.71228576	UP
14.69808995	DOWN
14.6995681	DOWN
14.64496304	DOWN
14.24774477	DOWN
14.56293847	DOWN
14.59497117	DOWN
14.5506059	DOWN
14.53323864	UP
14.47332381	UP
14.37896286	UP

14.42901381 UP
14.23731263 DOWN
14.3758348 DOWN
14.36377695 DOWN
13.81765524 UP
14.27323552 UP
14.23038466 UP
14.26477933 UP
14.1186366 UP
14.17071224 UP
14.24940646 UP
14.21462528 DOWN
13.99690763 UP
14.11771913 UP
14.02216292 UP
14.07396386 DOWN
13.99590715 UP
13.99058429 DOWN
14.03851997 DOWN
13.99649018 DOWN
13.68688252 UP
13.81720599 DOWN
13.3784757 UP
13.45574466 DOWN
13.66333891 DOWN
13.66697548 DOWN
13.5953265 DOWN
13.4409291 UP
12.77139979 UP
13.50887618 DOWN
13.40509106 DOWN
13.43822638 DOWN
13.45058403 DOWN
13.42314821 DOWN
13.41384117 DOWN
13.39128532 DOWN
13.39634893 UP
13.3793213 DOWN
13.37503926 DOWN
13.30178604 DOWN
13.34915151 DOWN
12.9946782 UP
13.33705718 UP
13.28947879 DOWN
13.27202244 UP
12.93382324 DOWN
13.20214546 UP
13.21188583 UP
13.18901104 UP
13.14418474 UP
13.12249838 DOWN
12.9928992 UP
12.94417134 DOWN
13.0850108 DOWN

13.07716799 DOWN
13.05980168 UP
13.03480676 DOWN
12.66425158 UP
13.0171872 DOWN
13.00706814 DOWN
12.98108556 UP
12.95159102 UP
12.79873091 DOWN
12.86926048 DOWN
12.71878976 UP
12.82566652 UP
12.83850749 UP
12.76296853 UP
12.77202627 DOWN
12.7283818 UP
12.71425984 UP
12.20950759 DOWN
12.61104418 UP
12.57051155 UP
12.60962719 DOWN
12.64200512 UP
12.68632672 UP
12.59730995 DOWN
12.54843005 DOWN
12.59379877 DOWN
12.05104498 UP
12.58249148 DOWN
12.53042596 DOWN
11.97528998 DOWN
12.41163785 UP
11.99186889 UP
12.35519243 UP
12.40107213 UP
11.84659103 UP
12.20362125 DOWN
12.26062643 DOWN
12.18114409 DOWN
12.22860942 UP
12.21534735 UP
12.1426808 DOWN
11.91244457 DOWN
12.06699691 UP
12.00480495 UP
12.01038633 DOWN
11.75966968 UP
11.92954724 UP
11.87726614 DOWN
11.85491821 DOWN
11.83760432 DOWN
11.71847824 UP
11.63978439 DOWN
11.32242936 DOWN
11.62505404 UP

11.66045432	UP
11.5243886	UP
11.5991475	UP
11.65877831	DOWN
11.4969932	UP
11.58244893	UP
11.61526705	DOWN
11.33046947	UP
11.57949326	UP
11.54039166	DOWN
11.507145	DOWN
11.47468388	DOWN
11.47212728	DOWN
11.4349897	DOWN
11.33305356	UP
11.39086843	UP
11.39378155	DOWN
10.67923101	UP
11.16370921	UP
11.37075444	DOWN
11.33965385	DOWN
11.33572952	UP
11.15025836	UP
11.29378085	UP
11.28736562	UP
11.18372417	UP
11.27359393	UP
11.20109512	DOWN
11.25234645	UP
11.06558151	UP
11.21979555	DOWN
11.09496784	UP
10.97068955	UP
11.09034179	UP
11.09050511	DOWN
11.05316659	UP
11.03852151	DOWN
10.9752175	DOWN
10.9994759	UP
10.9647701	DOWN
10.9754037	DOWN
10.95280681	UP
10.77784372	UP
10.909244	DOWN
10.34617564	UP
10.79537852	UP
10.87823778	DOWN
10.86320112	DOWN
10.82215229	DOWN
10.59955662	UP
10.73278531	DOWN
10.75341676	DOWN
10.73693595	DOWN
10.69510158	DOWN

10.64695948	UP
10.63030475	UP
10.49214884	UP
10.41101326	UP
10.05723991	UP
10.5844774	UP
10.57101147	DOWN
10.56020034	UP
10.42181585	UP
10.34444875	DOWN
10.43868122	DOWN
10.43438981	DOWN
10.29384333	UP
10.35451758	DOWN
10.37273237	UP
10.35736648	DOWN
10.23800435	UP
10.23155783	UP
9.796113352	DOWN
9.825158538	UP
10.14995553	DOWN
10.05572536	DOWN
10.00564641	DOWN
9.988632112	UP
9.991691039	UP
10.02129526	UP
10.00442799	DOWN
9.947538564	UP
9.968393693	UP
9.991276514	DOWN
9.915750261	UP
9.773083754	UP
9.85626561	DOWN
9.773317176	UP
9.779134319	DOWN
9.766947906	DOWN
9.633524756	UP
9.707732164	DOWN
9.490542985	DOWN
9.701566051	UP
9.223153526	UP
9.656616587	UP
9.658224858	DOWN
9.649525791	DOWN
9.642987454	DOWN
9.649247603	UP
9.530239536	UP
9.52984567	UP
9.382641565	DOWN
9.5002268	UP
9.47603187	DOWN
9.268587909	UP
9.456529545	DOWN
9.386401102	UP

9.357436609 DOWN
9.250024558 UP
9.195002907 DOWN
9.326393638 UP
9.305592958 UP
9.104235007 DOWN
9.248931798 DOWN
9.068344393 UP
9.186744521 DOWN
9.185727886 UP
9.133098488 DOWN
9.178147436 UP
9.169298853 DOWN
9.1573451 UP
9.043628297 UP
9.127308619 DOWN
9.04881187 UP
8.818922826 UP
9.062042653 DOWN
9.079517487 DOWN
9.070697991 DOWN
8.952277213 UP
9.01181053 DOWN
8.938092964 UP
8.882347136 UP
9.004728429 UP
8.964226852 DOWN
8.870599731 DOWN
8.912651334 UP
8.879038146 UP
8.82776966 UP
8.690830026 DOWN
8.773167881 DOWN
8.64663304 UP
8.752091018 DOWN
8.700538001 UP
8.743264007 DOWN
8.413395283 DOWN
8.59941881 UP
8.121612073 UP
8.456616489 UP
8.016198396 DOWN
8.68535961 DOWN
8.637186082 DOWN
8.642746908 DOWN
8.634273553 DOWN
8.598731316 DOWN
8.546699913 DOWN
8.179133897 DOWN
8.295621559 UP
8.330629453 UP
8.267072756 DOWN
8.11816872 UP
8.28798633 UP

8.141774073	UP
8.196745037	UP
8.179197352	DOWN
7.903186446	DOWN
8.170202526	UP
8.130946248	UP
8.055535764	UP
7.978372438	UP
7.617010677	UP
7.975416343	DOWN
7.918042785	DOWN
7.820652734	UP
7.716981032	DOWN
7.730159627	UP
7.532947148	DOWN
7.507108632	UP
7.16501789	UP
7.68461024	DOWN
7.657538463	UP
7.320025937	UP
7.583717415	UP
7.565152143	UP
7.514731139	DOWN
7.240664966	UP
7.509745816	DOWN
7.499682573	UP
7.470798729	UP
6.716069832	UP
7.241146169	UP
7.357375838	DOWN
7.354030238	UP
7.341899326	UP
7.218932387	UP
7.214306123	DOWN
7.101218841	UP
7.030683384	DOWN
7.187298324	UP
7.007813103	DOWN
6.92136004	DOWN
6.490867776	UP
6.98248398	UP
7.112048407	DOWN
7.080921633	DOWN
7.018948623	DOWN
6.950452955	DOWN
6.944461811	DOWN
6.87558672	UP
6.894955533	DOWN
6.850276219	DOWN
6.86852751	UP
6.603441844	UP
6.442369446	UP
6.771277994	DOWN
6.73371928	UP

6.477673408	UP
6.721090339	DOWN
6.679950305	DOWN
6.620117498	DOWN
6.357868357	UP
6.593366951	UP
6.579209441	DOWN
6.391853881	DOWN
6.513782265	UP
6.234882309	DOWN
6.479452967	UP
6.463801998	DOWN
6.459764919	DOWN
6.415901153	DOWN
6.390703232	DOWN
6.262228882	UP
6.193672172	UP
6.341194607	UP
6.001901792	UP
6.284781929	DOWN
5.989298196	UP
6.263479859	DOWN
6.251829239	DOWN
6.26562936	UP
6.1511461	DOWN
6.180066884	UP
6.055258834	DOWN
6.103372365	UP
6.053091313	DOWN
5.96389056	UP
6.020485351	UP
5.948204477	UP
5.911821912	UP
5.948344991	UP
5.8878242	DOWN
5.902989334	DOWN
5.881516922	UP
5.814594904	DOWN
5.802375825	DOWN
5.656601931	UP
5.772113324	DOWN
5.689505561	UP
5.663469198	UP
5.49383607	DOWN
5.652607391	DOWN
5.646803624	DOWN
5.590386932	DOWN
5.313081165	UP
5.448110601	UP
5.485731488	UP
5.549124021	DOWN
5.536941565	UP
5.485885566	DOWN
5.46236423	DOWN

5.301880207	DOWN
5.469408409	DOWN
5.433510988	DOWN
5.398233057	UP
5.399106281	UP
5.391471441	UP
5.341873324	DOWN
5.246855557	DOWN
5.23357251	DOWN
5.219001675	UP
4.722047631	UP
5.095954354	DOWN
5.160521636	UP
5.083818316	DOWN
5.100675539	DOWN
4.951141295	DOWN
5.039881373	DOWN
5.059153402	DOWN
4.525192205	UP
4.923220388	UP
4.901219546	DOWN
4.894942338	DOWN
4.872074567	UP
4.287219481	UP
4.697233085	UP
4.871592614	UP
4.850713458	UP
4.849163616	DOWN
4.761224762	DOWN
4.666672022	DOWN
4.734633936	DOWN
4.697401976	UP
4.746498531	DOWN
4.37335095	DOWN
4.701383369	DOWN
4.679515884	DOWN
4.650144871	UP
4.424106371	UP
4.52587299	DOWN
4.422840177	DOWN
4.449374114	DOWN
4.193486227	UP
4.4282015	DOWN
4.401919632	DOWN
4.362651286	UP
4.134339463	DOWN
3.933779783	DOWN
4.101131753	UP
3.342637258	UP
4.073988827	UP
4.014840315	DOWN
3.984572499	DOWN
4.029598596	DOWN
3.835033934	DOWN

3.941928436 DOWN
3.835202397 UP
3.810437962 UP
3.903858123 DOWN
3.828484568 UP
3.870733487 DOWN
3.821936989 DOWN
3.676820724 DOWN
3.656710224 UP
3.669999735 DOWN
3.661020204 DOWN
3.639354707 UP
3.607288537 UP
3.646708519 UP
3.596880955 DOWN
3.458061585 UP
3.118708933 UP
3.518014919 UP
3.477021615 DOWN
3.428137809 DOWN
3.212904031 UP
3.006010947 DOWN
2.973356055 UP
3.195559952 UP
3.142394665 DOWN
3.091986783 DOWN
2.976757302 DOWN
3.018998153 DOWN
2.769891574 UP
2.856719591 DOWN
2.900314613 DOWN
2.776495414 DOWN
2.506028602 DOWN
2.890414437 DOWN
2.922725111 UP
2.396871396 DOWN
2.720987114 UP
2.791321978 UP
2.814370144 DOWN
2.782633082 DOWN
2.768094578 DOWN
2.62415449 UP
2.617104188 UP
2.625588863 UP
2.285633177 UP
2.636073224 UP
2.539489788 DOWN
2.525175992 UP
1.8428782 UP
2.495683952 DOWN
2.179343084 DOWN
2.395876457 DOWN
2.282318157 UP
2.349937661 DOWN

2.235831518	DOWN
1.577470195	UP
1.939576693	DOWN
2.103236936	DOWN
2.075049564	DOWN
1.98662336	DOWN
1.739448915	DOWN
1.712374545	UP
1.601499527	DOWN
1.587327431	DOWN
1.528304683	DOWN
1.451177985	DOWN
1.333632692	DOWN
1.285698992	UP
1.145282443	DOWN
1.14364208	DOWN
1.093655089	DOWN
0.97149209	UP
0.797827553	UP
0.860753765	DOWN
0.846250584	UP
0.745526204	DOWN
0.3819919	UP
0.588360898	DOWN
0.507231618	UP
0.526293359	DOWN
0.476891396	UP
0.471344345	UP
0.21520407	DOWN
-0.259668713	UP
0.032973389	UP
0.175761456	DOWN
0.084314687	UP
-0.00543713	UP
0.053964882	DOWN
-0.073009667	DOWN
-0.666122978	DOWN
-0.57498768	DOWN
-0.648128739	DOWN
-1.20004052	DOWN
-1.554591403	DOWN
-1.991868865	DOWN
-1.81392775	UP
-2.923132563	DOWN
-2.542535658	DOWN
-3.44057375	DOWN
-4.124517407	DOWN
