# Supplementary figures and images for: Identification of Prognostic DNA Methylation Signatures in Lung Adenocarcinoma
Source: Oxid Med Cell Longev. 2022 Jun 29;2022:8802303. doi: 10.1155/2022/8802303 (PMC9259289; doi:10.1155/2022/8802303)

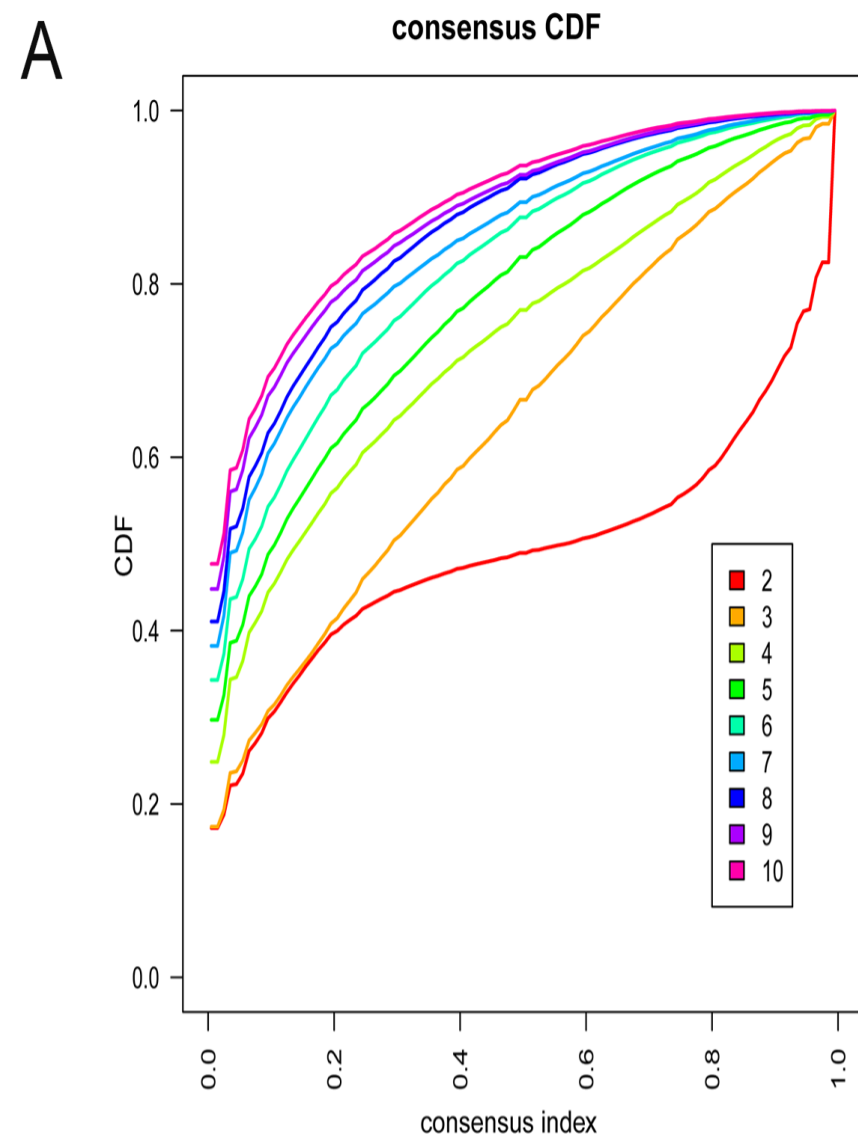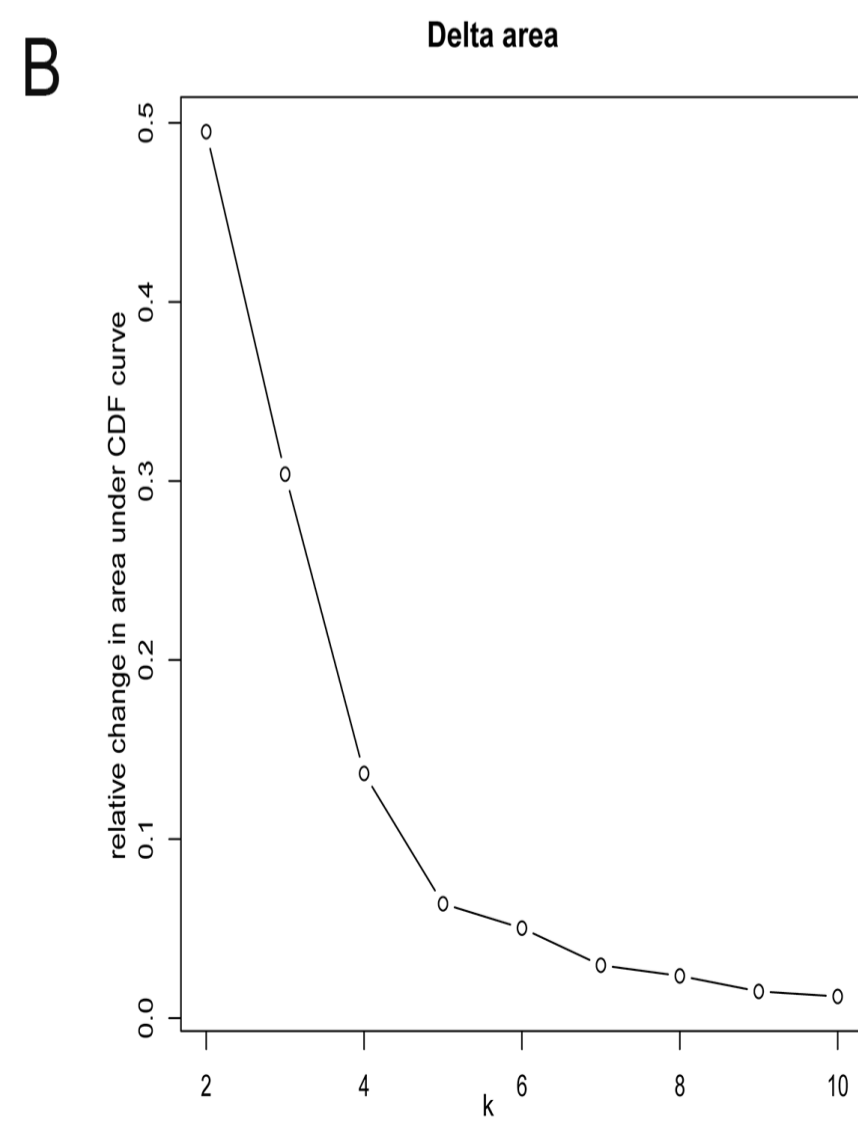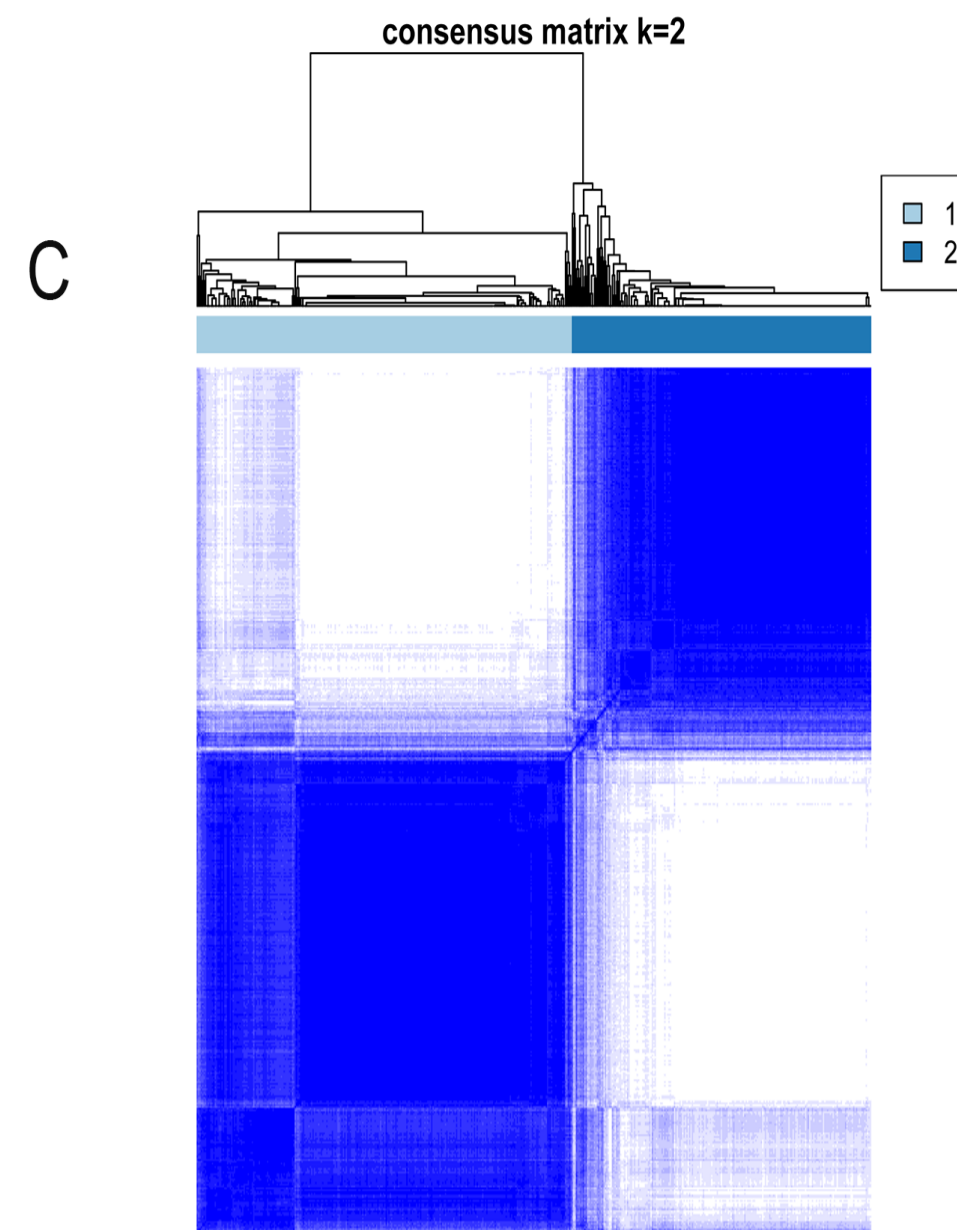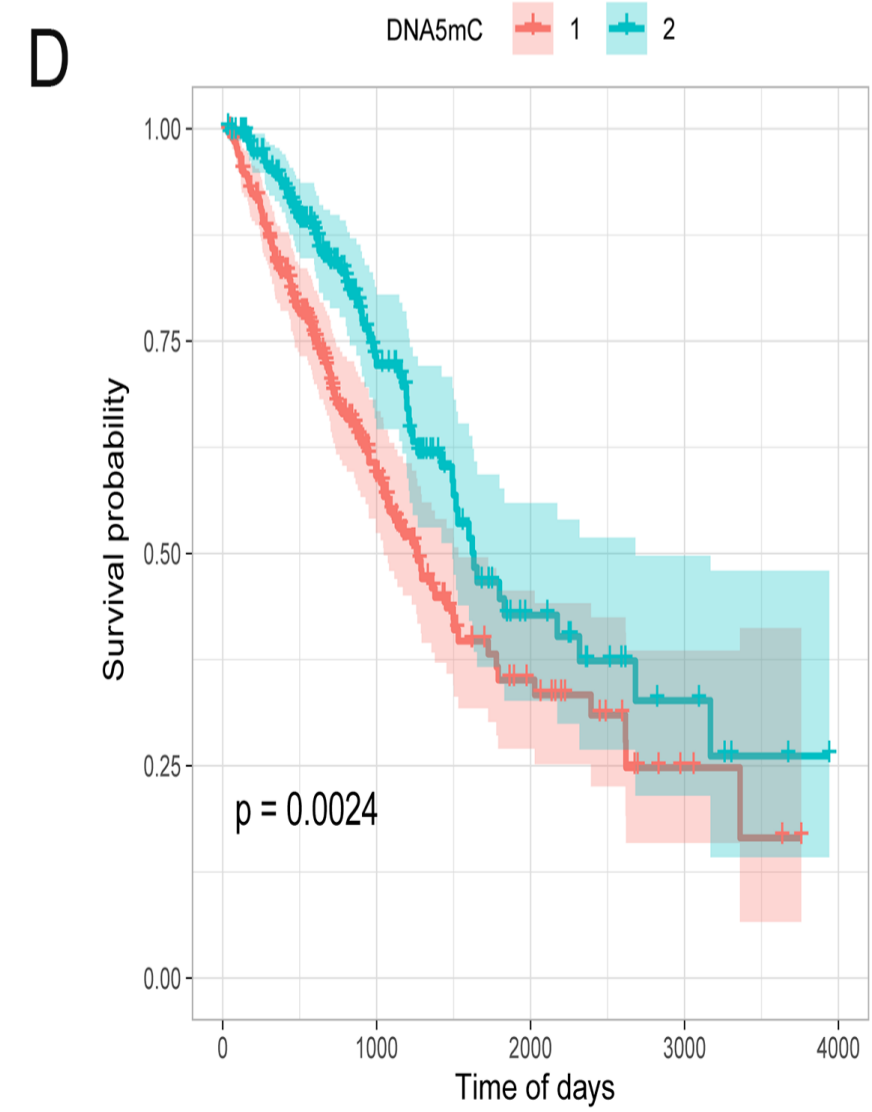

Supplement: Supplementary Materials — Supplement Fig1: consistent clustering of tumor DNA methylation-related gene expression profiles. A-B: the optimal number of clusters is determined, and the CDF delta area curve is observed; C: when the cluster is selected as 2 and it has relatively stable clustering results; D: the prognosis of DNA methylation-2 was significantly better than that of DNA methylation-1. Supplement Fig2: A: the samples had a good aggregation form in the space of the first and second dimensions; B: volcanic map of differential expression analysis between tumor DNA methylation subtypes. Supplement Fig3: consistent clustering of differentially expressed gene expression profiles among tumor DNA methylation subtypes. A-B: the optimal number of clusters is determined, and the CDF delta area curve is observed; C: when the cluster is selected as 2 and it has relatively stable clustering results; D: the prognosis of C1 was significantly better than that of C2. Supplement Fig4: the best gradient grouping of the tumor DNA methylation score (DMS). A: the score value of 4.75 was selected as the critical point; B: the group with low DMS had a good prognosis. Supplement Fig5: A: the relationship of the DNA methylation regulator pattern, ACRG molecular subtype, gene cluster, and DMS group is summarized in the Sankey diagram. B: the results showed that the meth.cluster with good prognostic correlation cluster 3 had a trend of lower DMS. C: dynamic flow diagram of tumor sample grouping and state transition. Supplement Fig6: consistent clustering of gene methylation profiles in tumors. A-B: the optimal number of clusters is determined, and the CDF delta area curve is observed; C: when the cluster is selected as 3 and it has relatively stable clustering results; D: the prognosis of C3 was significantly better than that of method.cluster-1/2. [file 8802303.f1.zip › Supplement Fig1.pdf]

A

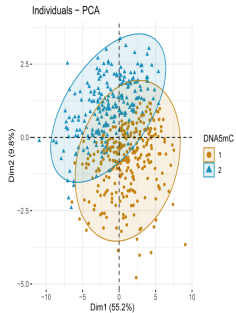

B

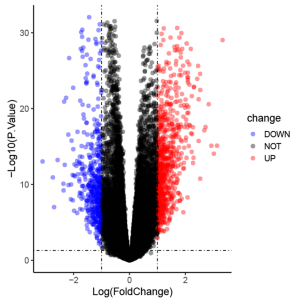

Supplement: Supplementary Materials — Supplement Fig1: consistent clustering of tumor DNA methylation-related gene expression profiles. A-B: the optimal number of clusters is determined, and the CDF delta area curve is observed; C: when the cluster is selected as 2 and it has relatively stable clustering results; D: the prognosis of DNA methylation-2 was significantly better than that of DNA methylation-1. Supplement Fig2: A: the samples had a good aggregation form in the space of the first and second dimensions; B: volcanic map of differential expression analysis between tumor DNA methylation subtypes. Supplement Fig3: consistent clustering of differentially expressed gene expression profiles among tumor DNA methylation subtypes. A-B: the optimal number of clusters is determined, and the CDF delta area curve is observed; C: when the cluster is selected as 2 and it has relatively stable clustering results; D: the prognosis of C1 was significantly better than that of C2. Supplement Fig4: the best gradient grouping of the tumor DNA methylation score (DMS). A: the score value of 4.75 was selected as the critical point; B: the group with low DMS had a good prognosis. Supplement Fig5: A: the relationship of the DNA methylation regulator pattern, ACRG molecular subtype, gene cluster, and DMS group is summarized in the Sankey diagram. B: the results showed that the meth.cluster with good prognostic correlation cluster 3 had a trend of lower DMS. C: dynamic flow diagram of tumor sample grouping and state transition. Supplement Fig6: consistent clustering of gene methylation profiles in tumors. A-B: the optimal number of clusters is determined, and the CDF delta area curve is observed; C: when the cluster is selected as 3 and it has relatively stable clustering results; D: the prognosis of C3 was significantly better than that of method.cluster-1/2. [file 8802303.f1.zip › Supplement Fig2.pdf]

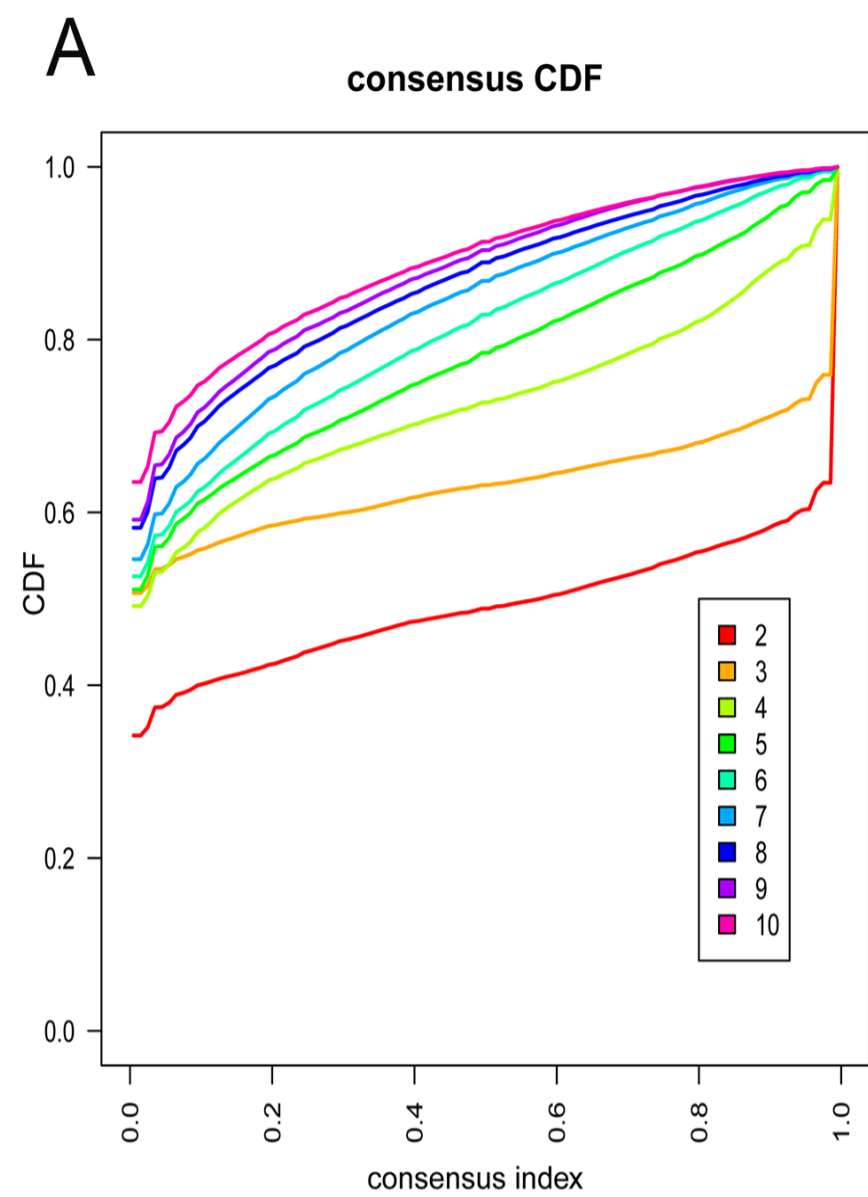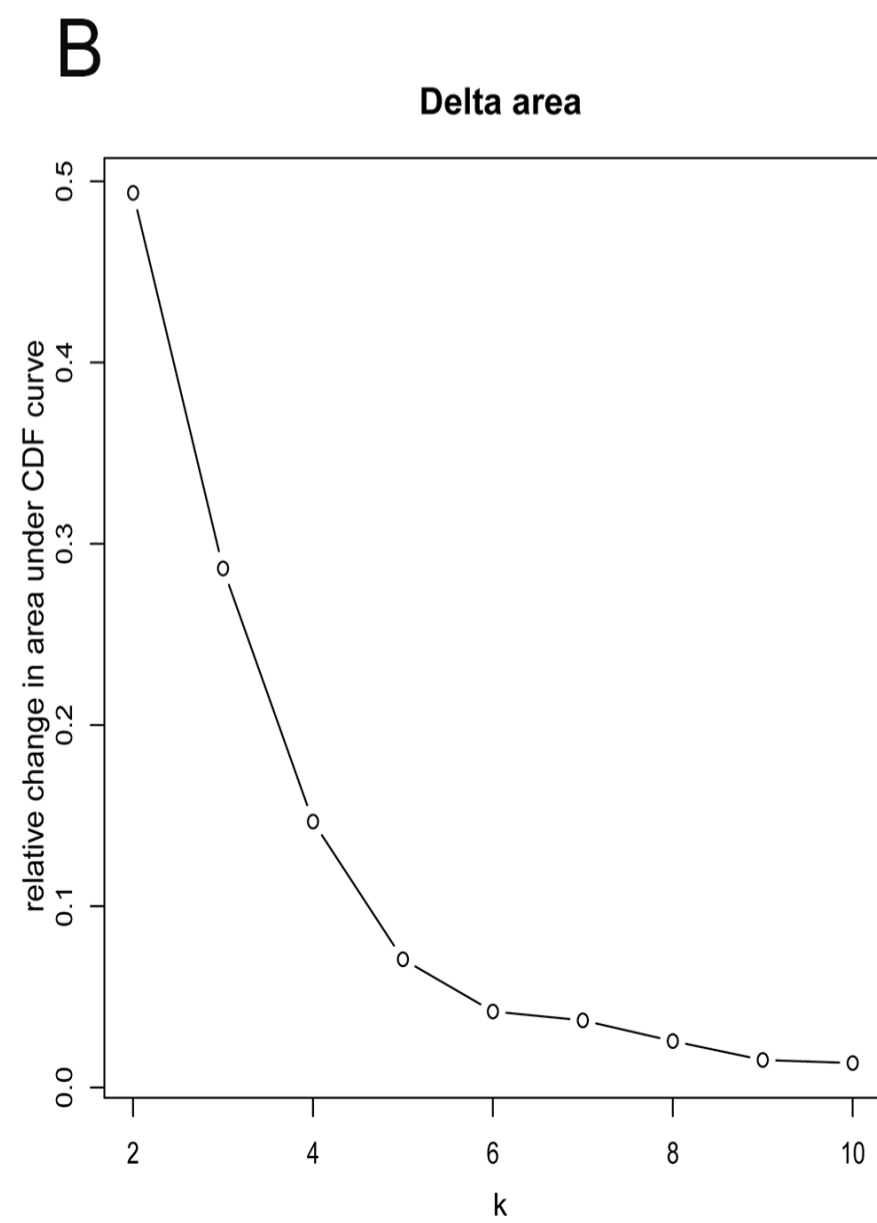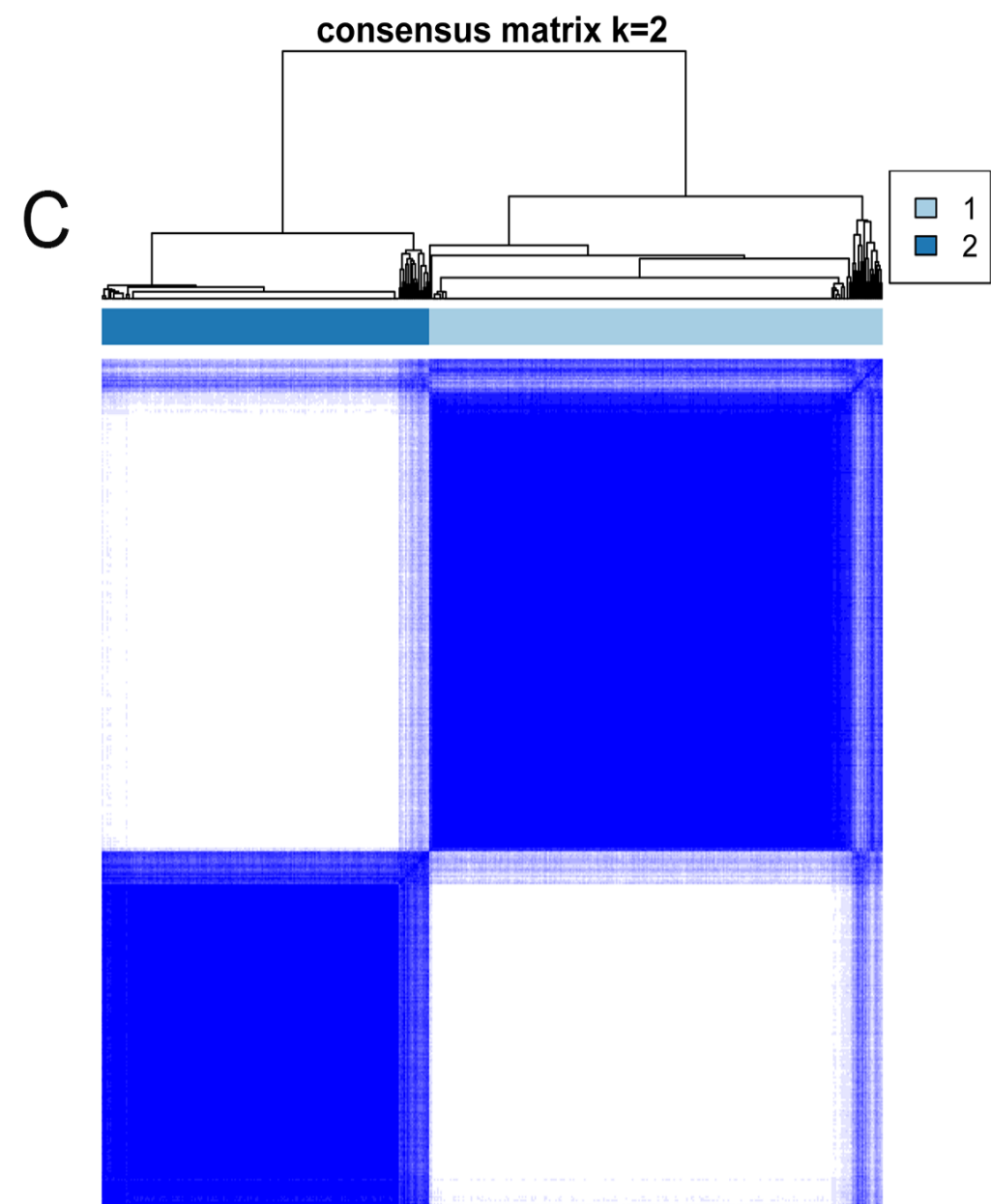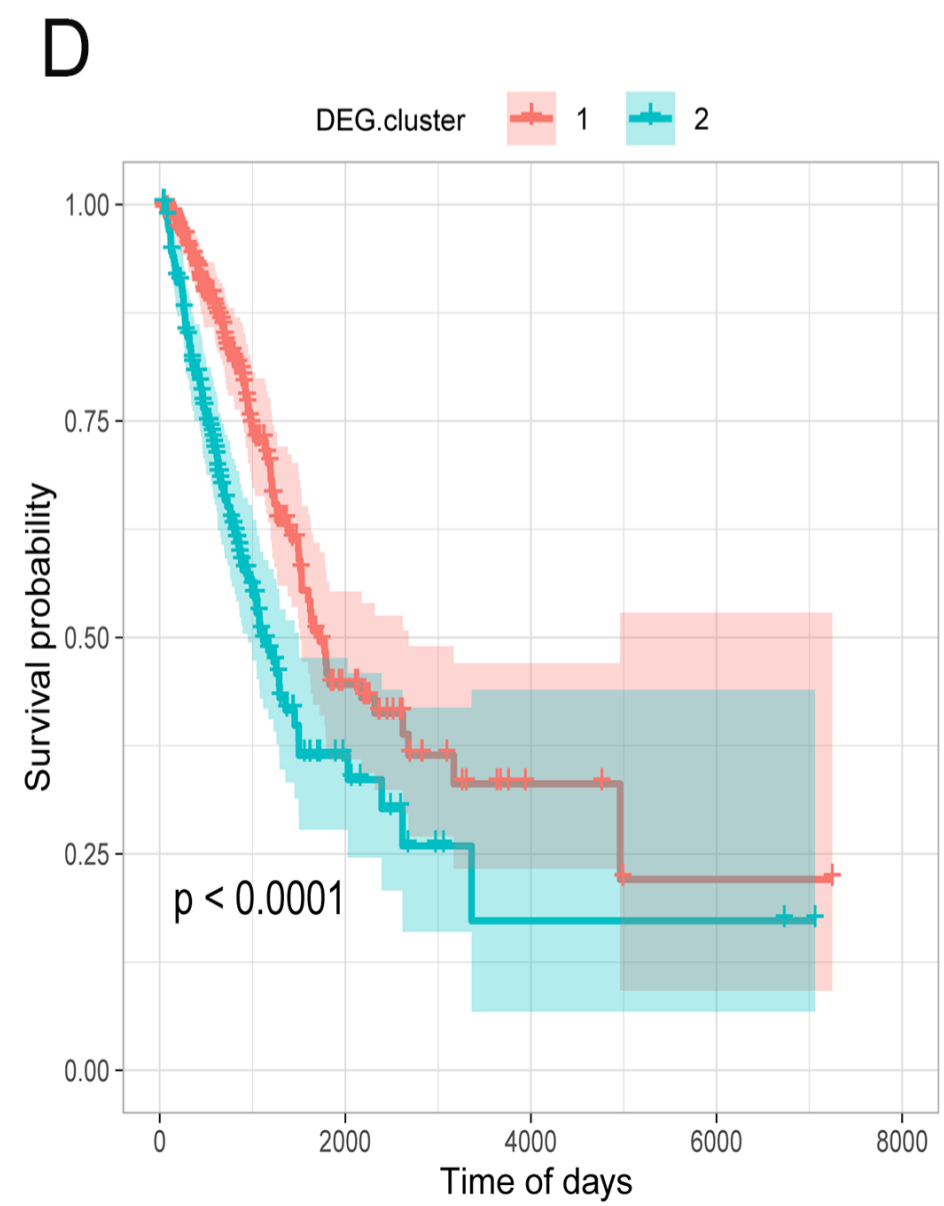

Supplement: Supplementary Materials — Supplement Fig1: consistent clustering of tumor DNA methylation-related gene expression profiles. A-B: the optimal number of clusters is determined, and the CDF delta area curve is observed; C: when the cluster is selected as 2 and it has relatively stable clustering results; D: the prognosis of DNA methylation-2 was significantly better than that of DNA methylation-1. Supplement Fig2: A: the samples had a good aggregation form in the space of the first and second dimensions; B: volcanic map of differential expression analysis between tumor DNA methylation subtypes. Supplement Fig3: consistent clustering of differentially expressed gene expression profiles among tumor DNA methylation subtypes. A-B: the optimal number of clusters is determined, and the CDF delta area curve is observed; C: when the cluster is selected as 2 and it has relatively stable clustering results; D: the prognosis of C1 was significantly better than that of C2. Supplement Fig4: the best gradient grouping of the tumor DNA methylation score (DMS). A: the score value of 4.75 was selected as the critical point; B: the group with low DMS had a good prognosis. Supplement Fig5: A: the relationship of the DNA methylation regulator pattern, ACRG molecular subtype, gene cluster, and DMS group is summarized in the Sankey diagram. B: the results showed that the meth.cluster with good prognostic correlation cluster 3 had a trend of lower DMS. C: dynamic flow diagram of tumor sample grouping and state transition. Supplement Fig6: consistent clustering of gene methylation profiles in tumors. A-B: the optimal number of clusters is determined, and the CDF delta area curve is observed; C: when the cluster is selected as 3 and it has relatively stable clustering results; D: the prognosis of C3 was significantly better than that of method.cluster-1/2. [file 8802303.f1.zip › Supplement Fig3.pdf]

A

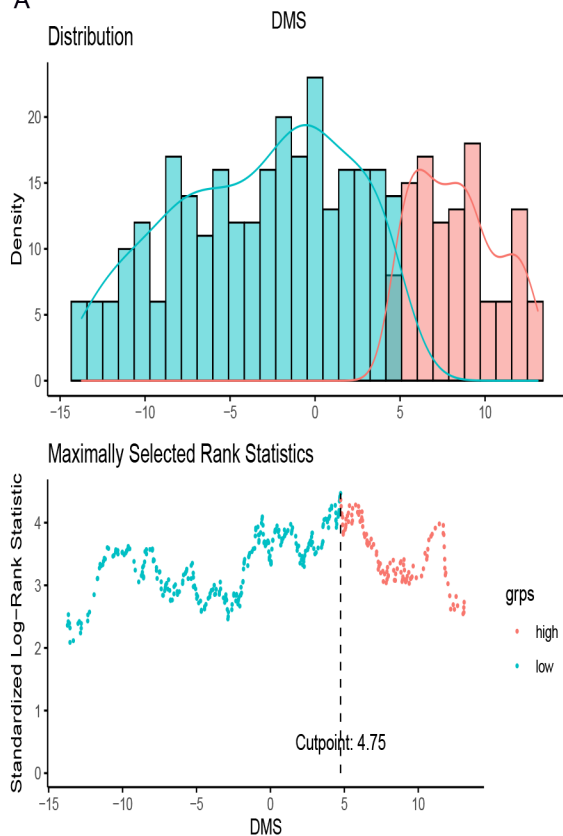

B

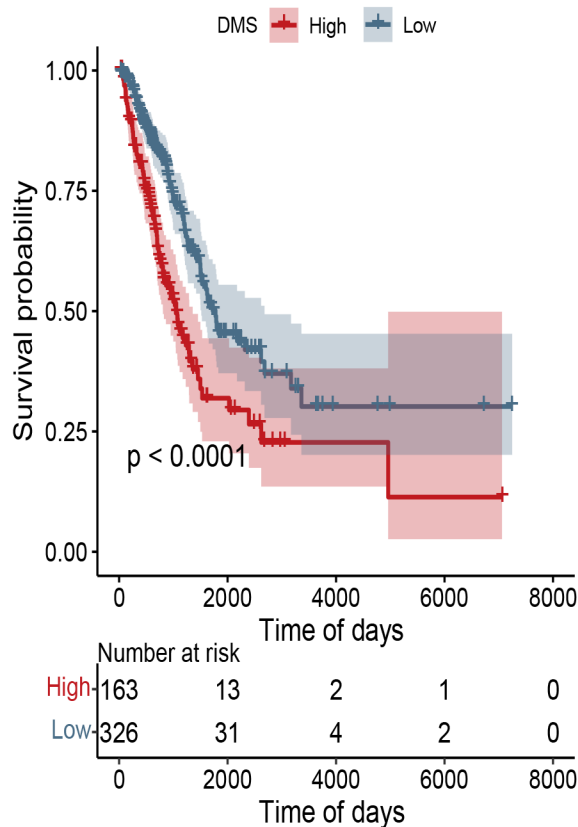

Supplement: Supplementary Materials — Supplement Fig1: consistent clustering of tumor DNA methylation-related gene expression profiles. A-B: the optimal number of clusters is determined, and the CDF delta area curve is observed; C: when the cluster is selected as 2 and it has relatively stable clustering results; D: the prognosis of DNA methylation-2 was significantly better than that of DNA methylation-1. Supplement Fig2: A: the samples had a good aggregation form in the space of the first and second dimensions; B: volcanic map of differential expression analysis between tumor DNA methylation subtypes. Supplement Fig3: consistent clustering of differentially expressed gene expression profiles among tumor DNA methylation subtypes. A-B: the optimal number of clusters is determined, and the CDF delta area curve is observed; C: when the cluster is selected as 2 and it has relatively stable clustering results; D: the prognosis of C1 was significantly better than that of C2. Supplement Fig4: the best gradient grouping of the tumor DNA methylation score (DMS). A: the score value of 4.75 was selected as the critical point; B: the group with low DMS had a good prognosis. Supplement Fig5: A: the relationship of the DNA methylation regulator pattern, ACRG molecular subtype, gene cluster, and DMS group is summarized in the Sankey diagram. B: the results showed that the meth.cluster with good prognostic correlation cluster 3 had a trend of lower DMS. C: dynamic flow diagram of tumor sample grouping and state transition. Supplement Fig6: consistent clustering of gene methylation profiles in tumors. A-B: the optimal number of clusters is determined, and the CDF delta area curve is observed; C: when the cluster is selected as 3 and it has relatively stable clustering results; D: the prognosis of C3 was significantly better than that of method.cluster-1/2. [file 8802303.f1.zip › Supplement Fig4.pdf]

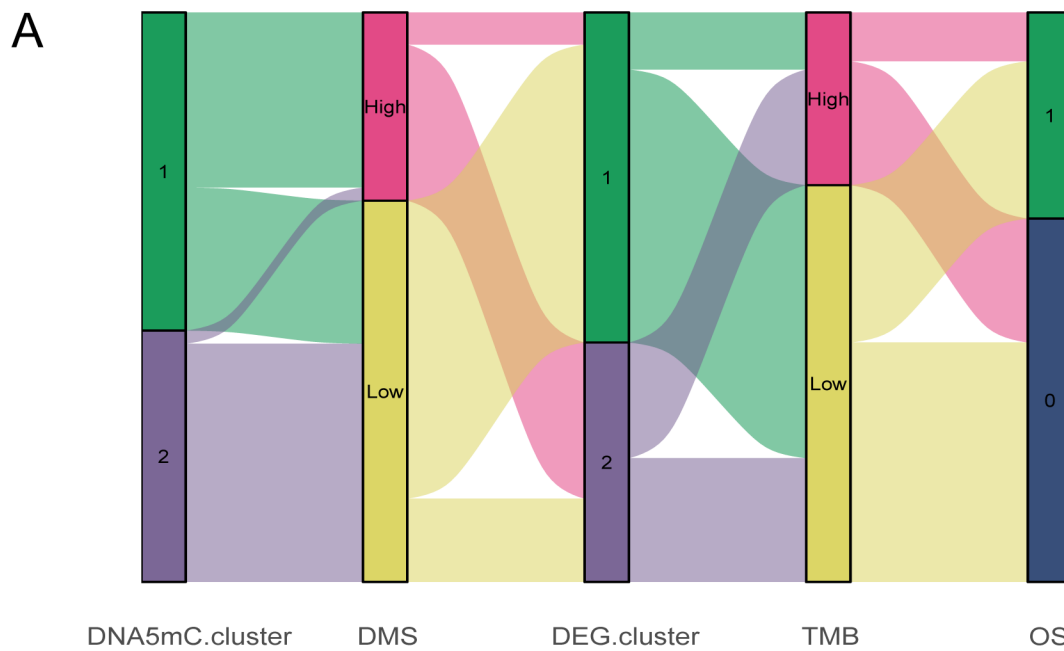

**B**

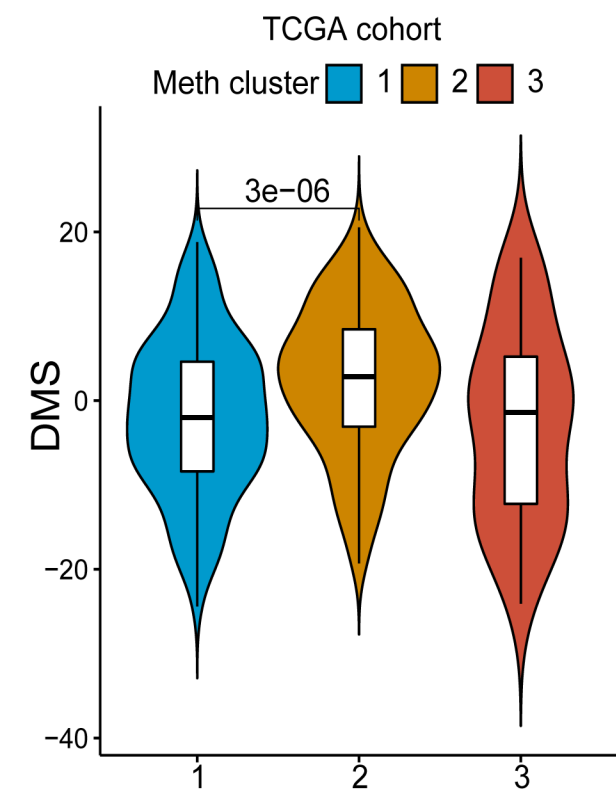

**C**

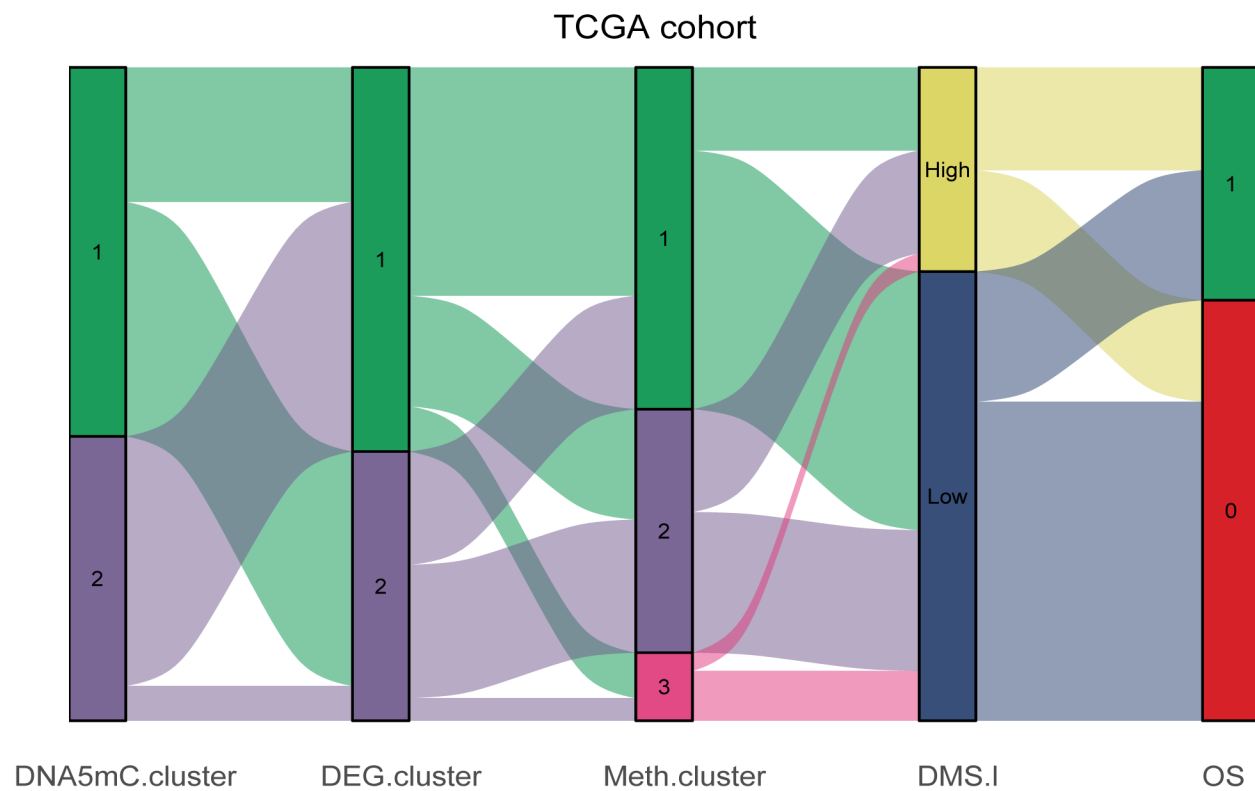

Supplement: Supplementary Materials — Supplement Fig1: consistent clustering of tumor DNA methylation-related gene expression profiles. A-B: the optimal number of clusters is determined, and the CDF delta area curve is observed; C: when the cluster is selected as 2 and it has relatively stable clustering results; D: the prognosis of DNA methylation-2 was significantly better than that of DNA methylation-1. Supplement Fig2: A: the samples had a good aggregation form in the space of the first and second dimensions; B: volcanic map of differential expression analysis between tumor DNA methylation subtypes. Supplement Fig3: consistent clustering of differentially expressed gene expression profiles among tumor DNA methylation subtypes. A-B: the optimal number of clusters is determined, and the CDF delta area curve is observed; C: when the cluster is selected as 2 and it has relatively stable clustering results; D: the prognosis of C1 was significantly better than that of C2. Supplement Fig4: the best gradient grouping of the tumor DNA methylation score (DMS). A: the score value of 4.75 was selected as the critical point; B: the group with low DMS had a good prognosis. Supplement Fig5: A: the relationship of the DNA methylation regulator pattern, ACRG molecular subtype, gene cluster, and DMS group is summarized in the Sankey diagram. B: the results showed that the meth.cluster with good prognostic correlation cluster 3 had a trend of lower DMS. C: dynamic flow diagram of tumor sample grouping and state transition. Supplement Fig6: consistent clustering of gene methylation profiles in tumors. A-B: the optimal number of clusters is determined, and the CDF delta area curve is observed; C: when the cluster is selected as 3 and it has relatively stable clustering results; D: the prognosis of C3 was significantly better than that of method.cluster-1/2. [file 8802303.f1.zip › Supplement Fig5.pdf]

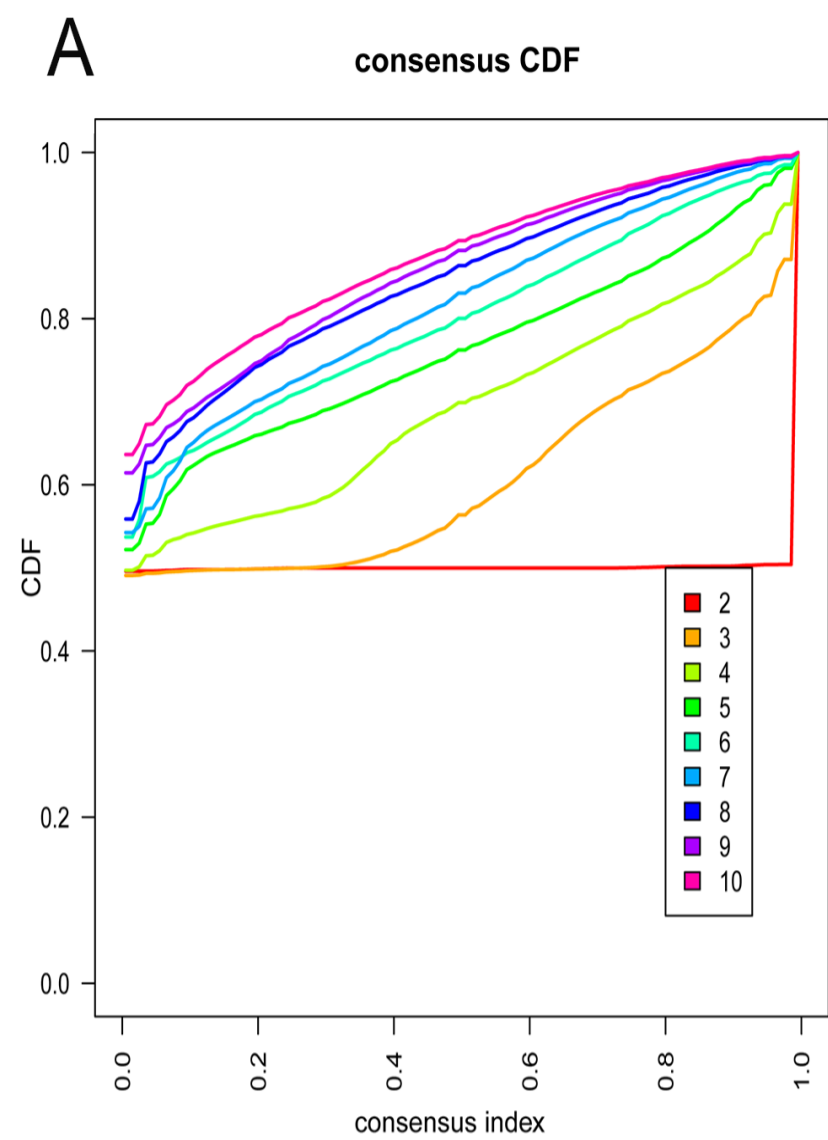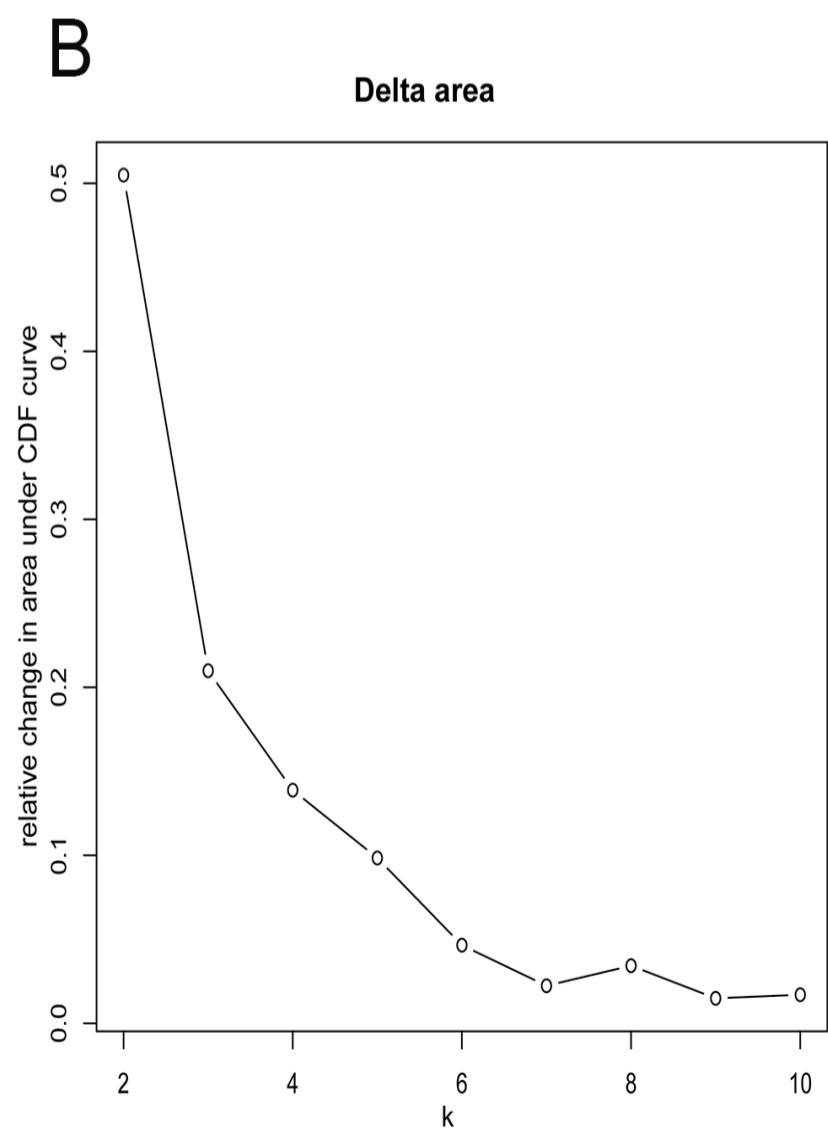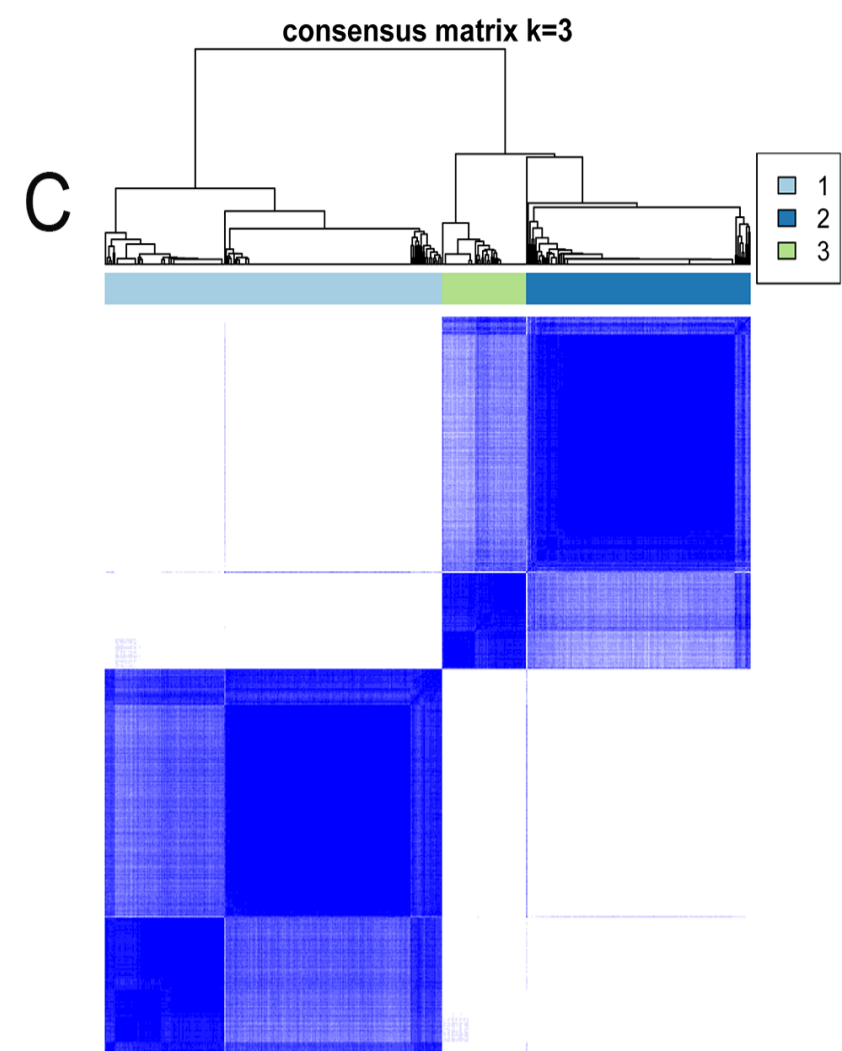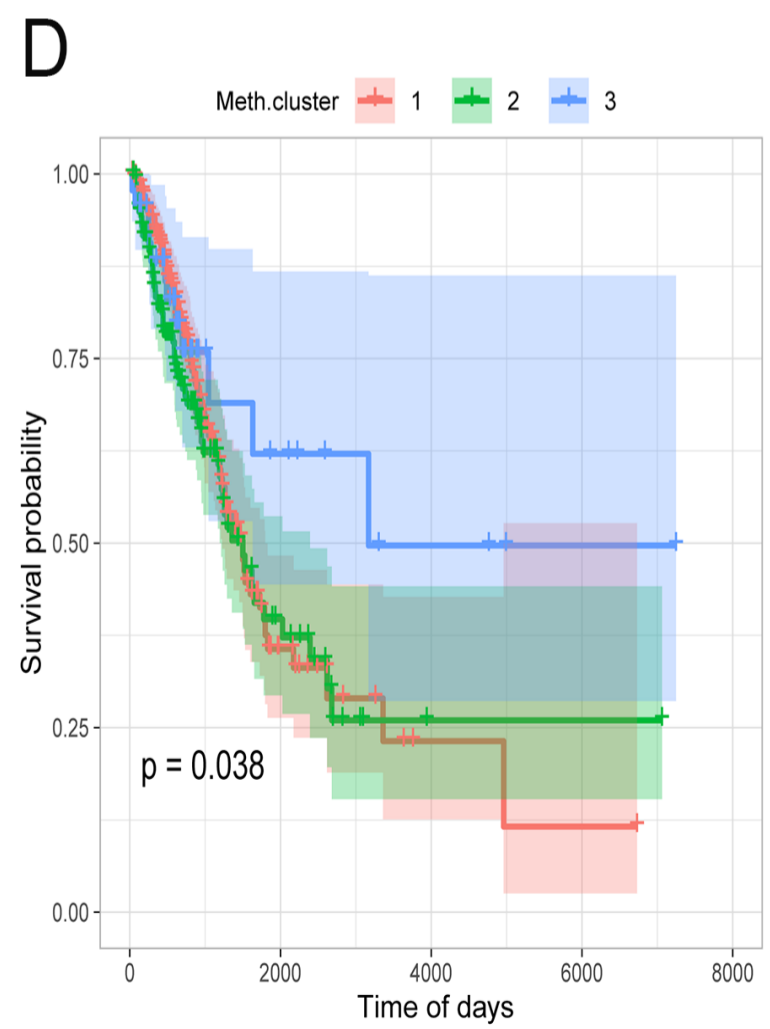

Supplement: Supplementary Materials — Supplement Fig1: consistent clustering of tumor DNA methylation-related gene expression profiles. A-B: the optimal number of clusters is determined, and the CDF delta area curve is observed; C: when the cluster is selected as 2 and it has relatively stable clustering results; D: the prognosis of DNA methylation-2 was significantly better than that of DNA methylation-1. Supplement Fig2: A: the samples had a good aggregation form in the space of the first and second dimensions; B: volcanic map of differential expression analysis between tumor DNA methylation subtypes. Supplement Fig3: consistent clustering of differentially expressed gene expression profiles among tumor DNA methylation subtypes. A-B: the optimal number of clusters is determined, and the CDF delta area curve is observed; C: when the cluster is selected as 2 and it has relatively stable clustering results; D: the prognosis of C1 was significantly better than that of C2. Supplement Fig4: the best gradient grouping of the tumor DNA methylation score (DMS). A: the score value of 4.75 was selected as the critical point; B: the group with low DMS had a good prognosis. Supplement Fig5: A: the relationship of the DNA methylation regulator pattern, ACRG molecular subtype, gene cluster, and DMS group is summarized in the Sankey diagram. B: the results showed that the meth.cluster with good prognostic correlation cluster 3 had a trend of lower DMS. C: dynamic flow diagram of tumor sample grouping and state transition. Supplement Fig6: consistent clustering of gene methylation profiles in tumors. A-B: the optimal number of clusters is determined, and the CDF delta area curve is observed; C: when the cluster is selected as 3 and it has relatively stable clustering results; D: the prognosis of C3 was significantly better than that of method.cluster-1/2. [file 8802303.f1.zip › Supplement Fig6.pdf]
